# Oncofetal TRIM71 drives liver cancer carcinogenesis through remodeling CEBPA-mediated serine/glycine metabolism

**DOI:** 10.7150/thno.99633

**Published:** 2024-08-12

**Authors:** Ying Su, Ziteng Li, Qin Li, Xinyi Guo, Hena Zhang, Yan Li, Zhiqiang Meng, Shenglin Huang, Zhixiang Hu

**Affiliations:** 1 Department of Integrative Oncology, Fudan University Shanghai Cancer Center, and Shanghai Key Laboratory of Medical Epigenetics, International Co-laboratory of Medical Epigenetics and Metabolism, Institutes of Biomedical Sciences, Shanghai Medical College, Fudan University, Shanghai, China.; 2 Department of Oncology, Shanghai Medical College, Fudan University, Shanghai, China.

**Keywords:** RNA-binding proteins, TRIM71, CEBPA, m6A, serine/glycine metabolism

## Abstract

**Rationale:** Tumor cells remodel transcriptome to construct an ecosystem with stemness features, which maintains tumor growth and highly malignant characteristics. However, the core regulatory factors involved in this process still need to be further discovered.

**Methods:** Single cell RNA-sequncing (scRNA-seq) and bulk RNA-sequencing profiles derived from fetal liver, normal liver, liver tumors, and their adjacent samples were collected to analyze the ecosystem of liver cancer. Mouse models were established to identify molecular functions of oncofetal-related oncogenes using hydrodynamic tail vein injection.

**Results:** We found that liver cancer rebuilt oncofetal ecosystem to maintain malignant features. Interestingly, we identified a group of RNA-binding proteins (RBPs) that were highly overexpressed with oncofetal features. Among them, TRIM71 was specifically expressed in liver cancers and was associated with poor outcomes. TRIM71 drove the carcinogenesis of hepatocellular carcinoma (HCC), and knockdown of TRIM71 significantly abolished liver cancer cell proliferation. Mechanistically, TRIM71 formed a protein complex with IGF2BP1, bound to and stabilized the mRNA of CEBPA in an m6A-dependent manner, enhance the serine/glycine metabolic pathway, and ultimately promoted liver cancer progression. Furthermore, we identified that all-trans-retinoic acid (ATRA) combined with e1A binding protein p300 (EP300) inhibitor A-485 repressed TRIM71, attenuated glycine/serine metabolism, and inhibited liver cancer cell proliferation with high TRIM71 levels.

**Conclusions:** We demonstrated the oncofetal status in liver cancer and highlighted the crucial role of TRIM71 and provided potential therapeutic strategies and liver cancer-specific biomarker for liver cancer patients.

## Introduction

The central concept of cancer development is that cancer cells acquire multiple cellular properties known as cancer hallmarks, including deregulating cellular metabolism and undergoing non-mutational epigenetic reprogramming 1. This phenomenon is more prevalent in low-mutant cancers such as liver cancer 2, 3. Reprogrammed liver cancer cells elevate expression of pluripotent stem cell-related genes to maintain continuous regeneration and metastasis. Strikingly, liver cancers form an oncofetal ecosystem through evolution and reprogramming, which further suppresses tumor immunity and ultimately leads to highly malignant characteristics and a dismal prognosis 4. The specific cell types of fetal-associated endothelial cells (ECs) and genes orchestrating the oncofetal reprogramming have been explored 5. However, the oncogenic drivers that promote liver cancer initiation and progression with oncofetal characteristics remain elusive.

Metabolic reprogramming enables tumor cells to adapt to the harsh microenvironment and support their rapid growth and proliferation 6. The metabolic changes in cancer cells involve alterations in glucose metabolism, amino acid metabolism, nucleotide metabolism, and lipid metabolism, which help to generate oncometabolites to meet the biosynthesis of macromolecules and support tumor growth 7. Recent studies have revealed that metabolic reprogramming is not only a consequence of oncogenic mutations but also a driver of cancer development 8, 9. However, in tumors with oncofetal characteristics, the metabolic pathways and regulatory factors that promote cancer development still need to be further elucidated.

RNA-binding proteins (RBPs) are core effectors that manipulate RNA homeostasis and remodel the cancer transcriptome at transcriptional and post-transcriptional levels 10. RBPs participate in controlling various stages of tumor development 11-13. Tumors also rely on abnormally high expression of RBPs such as FMRP to suppress the immune microenvironment and promote tumor growth and metastasis 14. More importantly, a single RBP, such as LIN28B, can initiate the occurrence of liver cancer, indicating that RBPs dysregulation are significant driving factors in cancer development 15. In recent years, studies have found that RBPs change tumor metabolic characteristics by causing transcriptome remodeling 16-19, which further maintains the malignant ability of tumors, indicating that RBPs play a crucial role in the metabolic reshaping process of tumors.

In this study, we systematically analyzed and clarified the oncofetal characteristics in liver cancer featuring initiating potential and prominent RNA processing activity. The identification of core regulators for oncofetal phenotype demonstrated strikingly high presence of RBPs. Among these oncofetal RBPs, we found that TRIM71 is a liver cancer-specific oncogene that drives the initiation and development of liver cancer. TRIM71 binds to and stabilizes the mRNAs of CEBPA, remodels PSPH and PSAT1 transcription, enhances the serine/glycine metabolic pathway, and ultimately promotes liver cancer progression. Furthermore, we identified that all-trans-retinoic acid (ATRA) and e1A binding protein p300 (EP300) inhibitor A-485 could repress TRIM71 expression, dampens glycine/serine/threonine metabolism, and inhibits liver cancer cell proliferation. Targeted inhibition of TRIM71 decreased liver cancer cell viability with high TRIM71 expression, suggesting that TRIM71 may be a core therapeutic target for liver cancer treatment.

## Results

### Identification of fetal-like liver malignant tumor cells

**Figure 1:**
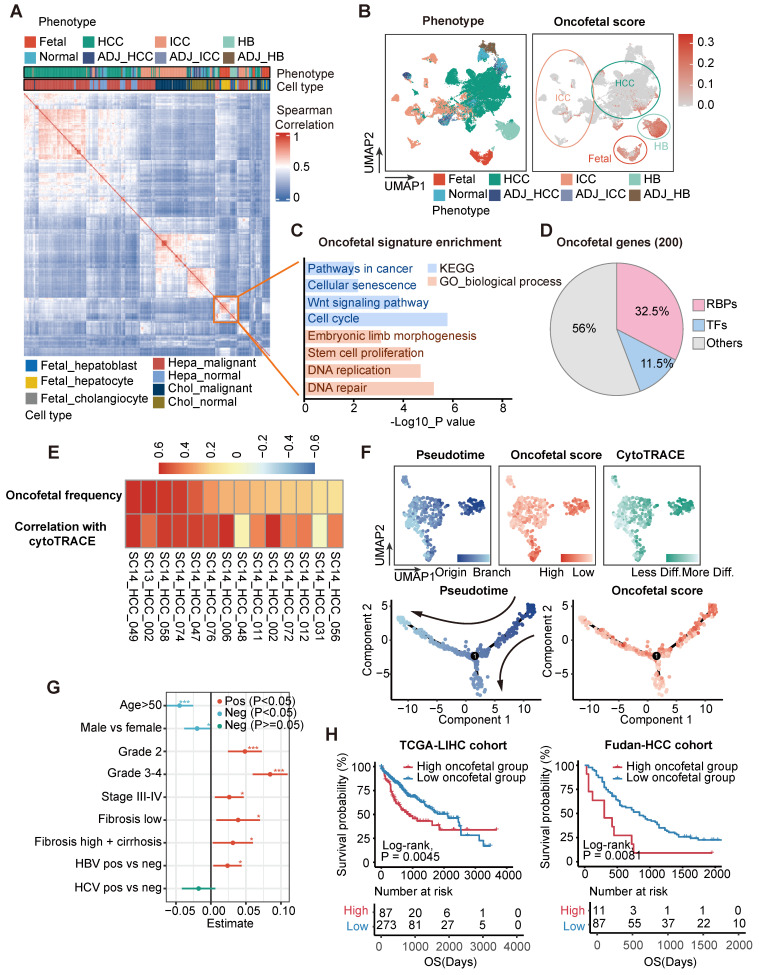
** Deciphering the oncofetal features of liver cancer and enriching them with abundant RNA-binding proteins.** (A) Heatmap demonstrating pair-wise correlations among epithelial pseudobulks. The yellow box represented the module formed by the co-clustering of epithelial cells from fetal liver, HB and HCC which denoted similar transcriptional pattern shared among these cells. (B) UMAP layout of all epithelial cells colored by liver phenotype (Left panel) and oncofetal score (Right panel). Oncofetal scores below 95% percent of normal cells were reset as zero. Color scheme of liver phenotype was the same as that in (A). (C) KEGG and gene ontology analysis of 200 oncofetal related genes. (D) The proportion of RBPs and TFs genes among the 200 oncofetal related genes. (E) Heatmap showing the positive correlation between oncofetal scores and cell developmental potential in the individual HCC samples with higher abundance of fetal-like cells. (F) UMAP layout and cell differentiation trajectory inferred for SC14_HCC_058 displaying the substantial agreement between cells with higher oncofetal scores and originating cells characterized by higher CytoTRACE scores and earlier pseudotime. (G) Correlation between oncofetal scores and clinicopathological variables in HCC samples of GepLiver bulk meta-cohort. Linear regression was performed for each parameter with dataset included as covariate. Lines represented 95% confidence intervals. (H) Kaplan-Meier plots demonstrating the association between oncofetal score and worse survival outcome in TCGA-LIHC (Left panel) and Fudan-HCC cohort (Right panel). Optimal cutpoint defined by the surv_cutpoint function of the survminer R package to delineate the most significant survival relationships for the oncofetal score was employed. For the TCGA-HCC cohort, the cut-off value was set at 1.04, defining the top 24% of HCCs as oncofetal high tumor group (n = 87) and 76% as low group (n = 273). For the Fudan-HCC cohort, the cut-off value was set at 1.87, defining the top 11% of HCCs as oncofetal high tumor group (n = 11) and 89% as low group (n = 87). *P<0.05, ***P < 0.001.

To systematically explore the oncofetal characteristics of liver malignant tumor cells, we retrieved single cell RNA-sequncing (scRNA-seq) profiles derived from fetal liver, normal liver, liver tumors, and their adjacent samples from the GepLiver data resource 20. Interestingly, we identified a highly correlated module consisting of hepatoblasts and hepatocytes from fetal liver as well as malignant tumor cells of HB and hepatocellular carcinoma (HCC) samples (**Figure 1A**), suggesting that these hepatoblastoma (HB) and HCC samples may recapitulate transcriptomic programs activated during hepatocyte differentiation. Subsequently, we identified a feature of this epithelial module, designated as the oncofetal signature, was made up of the top 200 differential genes compared to the rest of epithelial cells (**Table S1**). The expression of this oncofetal signature was then evaluated for all epithelial cells, and epithelial cells with high oncofetal activities were predominantly fetal-derived hepatoblasts and hepatocytes (**Figure S1A**). Consistently, most epithelial cells derived from fetal, HB and HCC scored highest (**Figure 1B, Figure S1B**). We have shown that 92 out of 102 HCC samples contained fetal-like epithelial cells in proportions ranging from 0.4% to 72.6% (**Figure S1C-D**), suggesting that oncofetal reprogramming could be a heterogeneous hallmark in HCC representing typical subpopulations.

We analyzed these 200 oncofetal related genes and found the enrichment of wnt signaling pathway and stem cell proliferation process (**Figure 1C**). Gene set enrichment analysis (GSEA) performed between fetal-like cells and non-fetal-like cells also revealed the significant enrichment of RNA processing pathways involving RNA splicing, metabolism, transport, and stabilization processes (**Figure S1E**). Interestingly, 65 of the 200 oncofetal genes belong to RBPs (**Figure 1D, Table S1**). As RBPs are known to be indispensable coordinators in the maintenance and differentiation from pluripotency, we speculated that RBPs could play an essential role in the recapitulation of the oncofetal phenotype. We further found that the oncofetal score demonstrated a significant positive relationship with the CytoTRACE score in liver tumors with a high abundance of fetal-like cells, representing a less differentiated state (**Figure 1E**). The developmental trajectory inferred for epithelial cells of one of the HCC samples, SC14_HCC_058, revealed two obvious sequential differentiation paths, both originating from cells with higher oncofetal scores (**Figure 1F**). The cell cluster harboring the highest oncofetal scores and CytoTRACE scores, as well as earlier pseudotime, was clearly separated from other cells in the UMAP plot, implying that the fetal-like cell subcluster may represent tumor-initiating cells with transcriptome-wide expressional changes.

We subsequently explored the clinicopathological features of liver cancer with oncofetal features. Transcriptomic profiles of a bulk meta-cohort were acquired from the GepLiver database involving liver phenotypes consistent with single-cell datasets. Linear regression was then performed for each parameter among 724 HCC samples of the meta-cohort, regressing out the effects of different data sources to evaluate potential associations between clinical characteristics and oncofetal scores (**Figure 1G**). HCC with higher oncofetal scores demonstrated higher tumor grade and tumor stage, fibrosis background, as well as hepatitis B virus (HBV) infection. Furthermore, oncofetal reprogramming was correlated with worse prognosis in both the TCGA-LIHC and Fudan-HCC cohorts, providing independent prognostic value (**Figure 1H, Figure S1F**). To further confirm that liver cancer single cells expressing the oncofetal signature are definitely cancer cells, we chose to designate the malignancy status from infer copy number variations (inferCNV) output in a semi-supervised approach. Briefly, we calculated the mean of inferCNV observations as the copy number variations (CNV) score for each cell and then compared the CNV scores of tumor cell clusters with those of normal epithelial and endothelial cells to ascertain the malignancy status. Heatmap for each single-cell dataset was generated to manually confirm the classification of cancer cells. These heatmaps revealed significant more or less expression in malignant epithelial cells than normal reference and control cells denoting copy number amplifications or deletions, further validating the accuracy of our method for distinguishing between tumor and non-tumor epithelial cells (**Figure S2**). Thus, our analysis identified a subpopulation of liver tumor cells sharing transcriptomic changes with fetal hepatoblasts and hepatocytes and revealed that this oncofetal cell cluster harbored tumor-initiating potential as well as prominent RNA processing activities.

### TRIM71 serves as a liver cancer-specific biomarker and RBP with oncofetal phenotype

**Figure 2:**
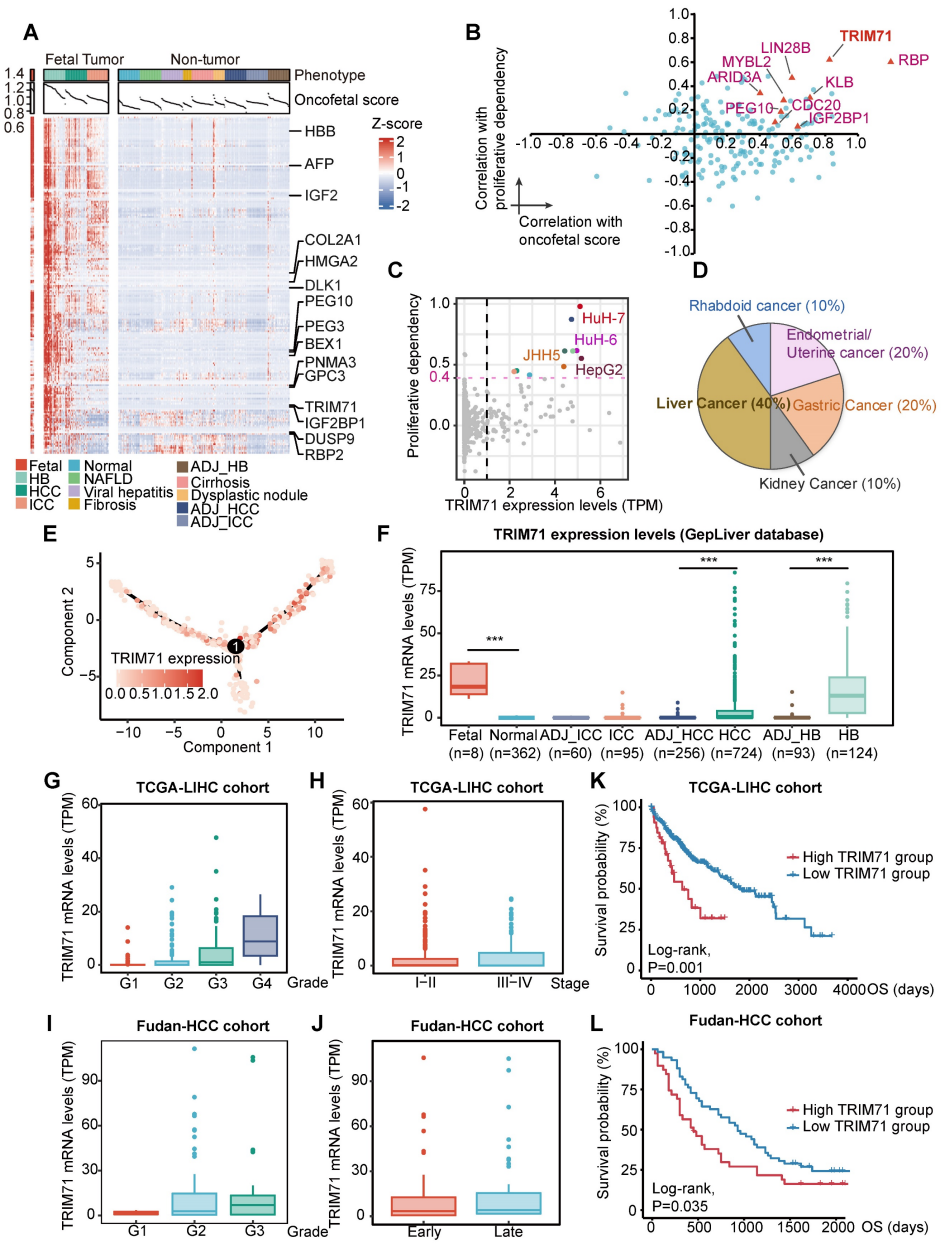
** TRIM71 serves as a liver cancer specific oncogene with oncofetal phenotype.** (A) The expression pattern of oncofetal signature consisting of 200 genes in GepLiver bulk meta-cohort. For each liver phenotype, bulk tissues were downsampled to 50 for optimal figure clarity. Top 15 features most elevated in fetal-like cells versus not group were marked in the heatmap. (B) Core oncofetal genes filtered by the associations of gene expression with both proliferative dependency score (y axis) and oncofetal score (x axis) in liver cancer cell lines. RBPs favoring the oncofetal phenotype as well as essential in liver cancer cell lines were shown as triangles with gene name annotated aside. (C) Scatterplot showing the relationship between TRIM71 expression and proliferative dependency scores in CCLE pan-cancer cell lines. Black dashed lines represented the TRIM71 TPM value as 1 and cell dependency score as -0.4. Liver cancer cell lines with TRIM71 TPM > 1 and dependency score < -0.4 were colored red and annotated aside. (D) Pie chart demonstrated the lineage distribution of top 10 cell lines with highest TRIM71 expression and proliferative dependency scores, suggesting TRIM71 is specifically vital for liver cancer proliferation. (E) Cell differentiation trajectory of SC14_HCC_058 colored by TRIM71 expression presenting the marked overlap between TRIM71-expressing cells and originating cells. (F) TRIM71 expression among liver phenotypes in GepLiver bulk meta-cohort. (G-H) TRIM71 expression grouped by tumor grade (G) and tumor stage (H) in TCGA-LIHC cohort. (I-J) TRIM71 expression grouped by tumor grade (I) and tumor stage (J) in Fudan-HCC cohort. (K-L) Kaplan-Meier survival curve showing TRIM71 as a prognostic factor associated with worse overall survival in TCGA-LIHC cohort (K) and Fudan-HCC cohort (L). Significance was determined with log-rank test. For box plots in (F-J), center line represents the median whereas limits show upper and lower quartiles. Data extend beyond the 1.5 times of the interquartile range from box limits were shown as outlier points. ***P < 0.001.

We further investigated the expression patterns of oncofetal feature genes. Consistent with our findings in scRNA-seq profiles, the expression of most oncofetal genes was consistently elevated in fetal liver, part of HB and HCC tumors as well as a few intrahepatic cholangiocarcinoma (ICC) tumor samples (**Figure 2A**). To identify the core regulators of the oncofetal phenotype among oncofetal feature genes, we estimated the associations of gene expression with both oncofetal scores and gene dependency scores in cancer cell line encyclopedia (CCLE) liver cancer cell lines, indicating contributions to fetal-like phenotype and oncogenic functions. Notably, a large group of RBPs were enriched, further highlighting the vital role of RBPs in oncofetal reprogramming (**Figure 2B**). Among these RBPs, LIN28B and IGF2BP1 have been widely recognized as oncofetal proteins that activate and coordinate RNA life cycle throughout embryonic development and abnormally re-express in cancer 21, 22. Notably, a RBP termed as tripartite motif-containing 71 (TRIM71, also known as LIN-41), which functions as a key regulator of proliferation and differentiation in stem and progenitor cells, ranked highest in both oncofetal contribution and liver cancer dependency. As a conserved target of let-7 miRNA, TRIM71 was essential to lifting let-7 inhibition to pluripotency, and recognized as a key reprogramming factor for induced pluripotent stem cell (iPSC) 23. In light of this, we further explored the cancer dependency score for TRIM71 in all CCLE cancer cell lines and strikingly noticed that among 10 cell lines, the viability of which was most affected by TRIM71 knockdown, four cell lines were derived from liver cancer (**Figure 2C-D**). Such observations indicated that TRIM71 knockdown inhibited cell proliferation in a highly liver-specific manner.

In accordance with the oncofetal expression pattern, TRIM71 was exclusively overexpressed in fetal and liver cancers including HCC and HB but less overexpressed in ICC, with its expression at the single-cell level showing marked overlap with originating cells according to the differentiation trajectory built from the SC14-HCC-058 sample (**Figure 2E-F**). Pan-cancer analysis was found that TRIM71 was high expressed in liver cancer and ovarian cancer (**Figure S3A**). Furthermore, TRIM71 was sequentially upregulated as tumor grade increased, whereas the expression remained little difference between early and late tumor stage, supporting the potential function of TRIM71 in the initiation of less differentiated liver tumor (**Figure 2G-J**). In addition, the expression of TRIM71 demonstrated significant associations with worse survival outcomes in both TCGA-LIHC and Fudan-HCC cohorts (**Figure 2K-L**). Hence, TRIM71 may serve as a liver cancer-specific biomarker and RBP in regulating fetal-like phenotype and maintaining the survival advantage conferred by oncofetal reprogramming, which deserves further exploration.

### TRIM71 drives carcinogenesis of liver cancer

**Figure 3:**
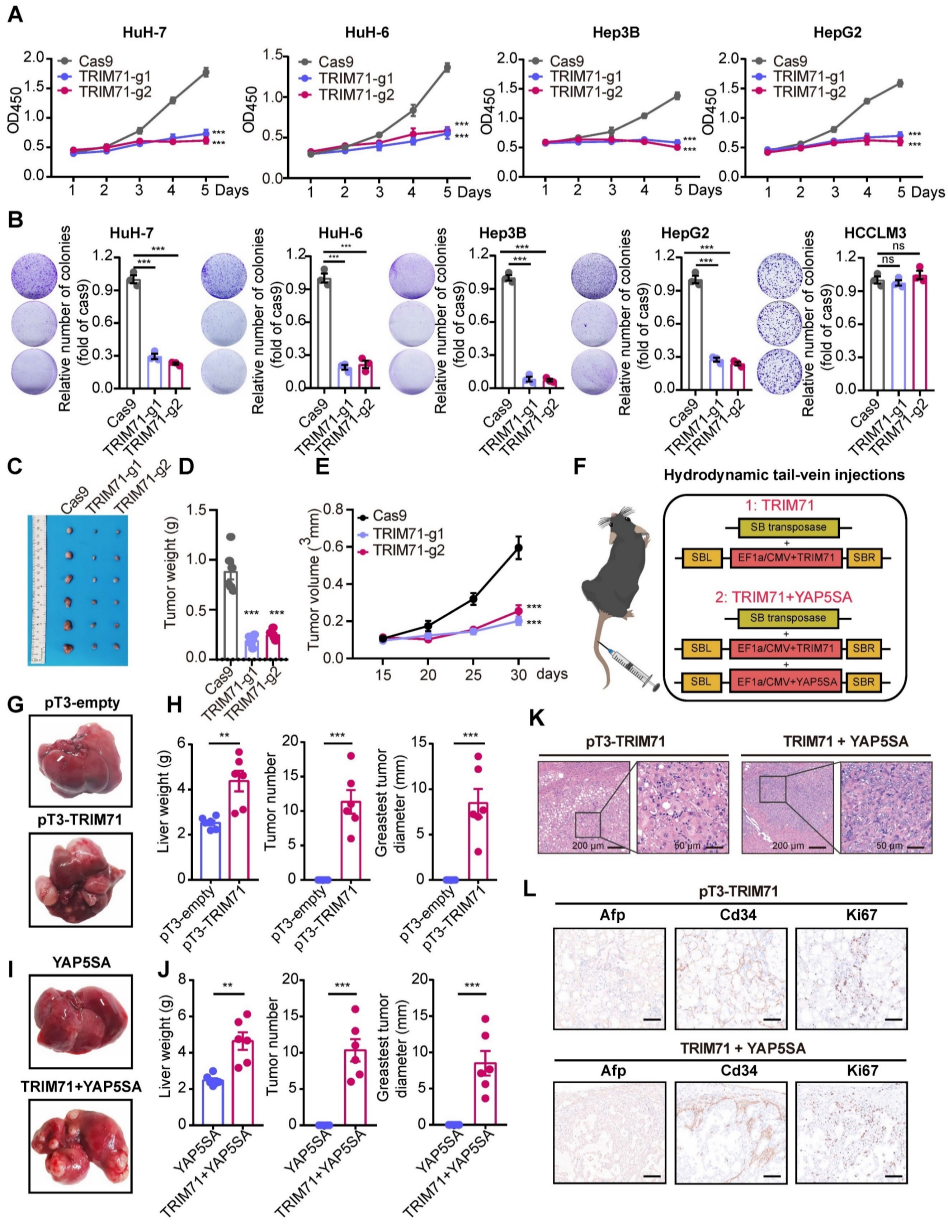
** TRIM71 drives liver cancer initiation and progression.** (A) Effects of TRIM71 knockdown on cell proliferation in HuH-7, HuH-6, Hep3B and HepG2 cells infected with sgTRIM71 specific gRNAs and Cas9 lentiviruses and determined by cell-counting kit 8 (CCK-8) assay. (B) Colony formation assays of HuH-7, HuH-6, Hep3B, HepG2 and HCCLM3 cells infected with sgTRIM71 specific gRNAs and Cas9 lentiviruses. (C) Images of the xenograft mouse models implanted with HuH-7 cells with or without TRIM71 knockdown infected with sgTRIM71 specific gRNAs and Cas9 lentiviruses. (D-E) Effects of TRIM71 knockdown on the tumor weight (D) and tumor volume (E) of HuH-7 xenograft tumors. (F) The experimental scheme for the generation of liver tumor mouse models using hydrodynamic tail-vein injection technology. (G) Representative images of tumors dissected from pT3-empty and pT3-TRIM71 liver tumor mouse models. (H) Liver weight, tumor number and greatest tumor diameter in pT3-empty and pT3-TRIM71 liver tumor mouse models. (I) Representative images of tumors dissected from YAP5SA and TRIM71 + YAP5SA liver tumor mouse models. (J) Liver weight, tumor number and greatest tumor diameter of YAP5SA and TRIM71 + YAP5SA liver tumor mouse models. (K) The HE staining in TRIM71 and TRIM71 + YAP5SA liver tumor mouse models. (L) Immunohistochemistry staining showing Afp, Cd34 and Ki67 protein expression in tumor tissues of TRIM71 and TRIM71 + YAP5SA liver tumor mouse models. Values represent the mean ± SEM. *P<0.05, **P < 0.01, ***P < 0.001.

As TRIM71 is a potential and liver cancer-specific driver analyzed in our previous studies, we aimed to clarify its oncogenic function in liver cancer. Analysis of the Depmap database revealed abundant TRIM71 expression in HepG2, HuH-7, and HuH-6, whereas TRIM71 had negative expression in Li-7 and SNU-449 liver cancer cell lines (**Figure S3B**). Immunoblot analysis revealed similar expression patterns of TRIM71 protein levels (**Figure S3C**). HuH-7, Hep3B, SNU-449, MHCC97H, HCCLM3 and Li-7 are liver cancer cell lines with characteristics of HCC, while HuH-6 and HepG2 are liver cancer cell lines with HB. Next, guide RNAs targeting TRIM71 were constructed and packaged together with the Cas9 plasmid into lentivirus, and infected HuH-7 and Hep3B cells with expression of TRIM71 using clustered regularly interspaced short palindromic repeats/Cas9 (CRISPR/Cas9) method. Western blot analysis showed the efficient knockdown ability (**Figure S3D**). Cell-counting kit 8 (CCK-8) assay (**Figure 3A**) and colony formation assay (**Figure 3B**) showed that TRIM71 knockdown significantly inhibited liver cancer cell proliferation with high TRIM71 expression but not in HCCLM3 with low TRIM71 levels. Additionally, silencing of TRIM71 significantly inhibited the tumorigenic ability (**Figure 3C**) and reduced the tumor weight (**Figure 3D**) and tumor volume (**Figure 3E**) of HuH-7 cells in an in vivo xenograft mouse model.

To determine whether TRIM71 is a driver for liver cancer initiation and progression, we established two mouse models by hydrodynamic tail vein injection to deliver vectors into liver cells to induce malignant transformation. We constructed the sleeping beauty (SB) transposon vector for overexpression of TRIM71 alone or TRIM71 combined with oncogene YAP5SA (**Figure 3F**). We observed that TRIM71 alone could induce visible liver tumors nearly 6 months (**Figure 3G-H**). YAP5SA alone cannot induce liver tumor formation, but the combination of TRIM71 and YAP5SA can significantly promote liver tumor initiation and malignant progression within approximately 3 months (**Figure 3I-J, Figure S3E**). Hematoxylin-eosin (HE) staining showed that these TRIM71-induced mouse liver tumors exhibited some morphological characteristics of HCC (**Figure 3K**). Immunohistochemistry (IHC) assay indicated high abundance of α-fetoprotein (Afp), Cd34, and the cell proliferation marker Ki-67 in the nodules from mice with TRIM71 overexpression (**Figure 3L**). To more comprehensively clarify the types and pathological characteristics of liver tumors induced by TRIM71, we conducted HE staining and IHC staining on mouse liver cancer tissues induced by pT3-TRIM71 and TRIM71 + YAP5SA, as well as on ICC mouse tumor tissues caused by YAP5SA + AKT and HB mouse tumor tissues caused by YAP5SA + CTNNB1. Afp, Arg1, Ck7, Ck19, and β-catenin were chosen to identify pathological characteristics of liver tumors. Among these, HCC is characterized by high expression of Afp and Arg1, HB by nuclear β-catenin staining, and ICC by high expression of Ck7 and Ck19. The HE staining revealed that our collected pT3-TRIM71 samples all had HCC-like characteristics and were distinctly different from classic HB (YAP5SA + CTNNB1) and ICC (YAP5SA + AKT) tumor tissues (**Figure S4A-B**). However, we also noted that the liver cancer tissues induced by TRIM71 + YAP5SA exhibited characteristics of both HCC and mixed HCC-ICC features (**Figure S4C**). The IHC experiments indicated that the liver cancer tissues induced by pT3-TRIM71 alone did not express Ck7 and Ck19, and β-catenin was primarily localized in the cytoplasmic matrix, with tumors expressing AfP and Arg1. These results also suggest that the liver tumors induced by pT3-TRIM71 alone are almost entirely HCC-like (**Figure S5A**). IHC experiments revealed that liver cancer tissues induced by TRIM71 + YAP5SA mainly exhibited HCC-like and mixed HCC-ICC features, likely due to the influence of the YAP gene (**Figure S5B**).

As TRIM71 maintains stemness and proliferation in mouse embryonic stem cells and participates establishment of oncofetal ecosystem in liver cancer, we tried to explore the role of TRIM71 in stemness of liver cancer. We firstly analyzed the correlation between the expression of TRIM71 and tumor stem cell markers in HCC and HB and found significant positive correlation between TRIM71 and CD133, CD24, EPCAM, and LGR5 in HCC and HB tumor samples (**Figure S6A-B**). Moreover, TRIM71 significantly upregulated the mRNA levels of Cd133, Cd24, and Epcam validated by RNA-seq data (**Figure S6C**) and western blot (**Figure S6D**) in TRIM71 induced mice liver tumors via hydrodynamic tail-vein injection. Meanwhile, we overexpressed TRIM71 and assessed its in vitro spheres formation ability in the liver cancer cell line Li-7, which exhibits low expression of TRIM71. The results revealed that TRIM71 overexpression significantly enhanced the spheres formation ability of liver cancer cells in vitro (**Figure S6E**). Taken together, these results suggest that TRIM71 specifically drives liver cancer initiation and progression.

### TRIM71 controls metabolic pathways and CEBPA mRNA levels

**Figure 4:**
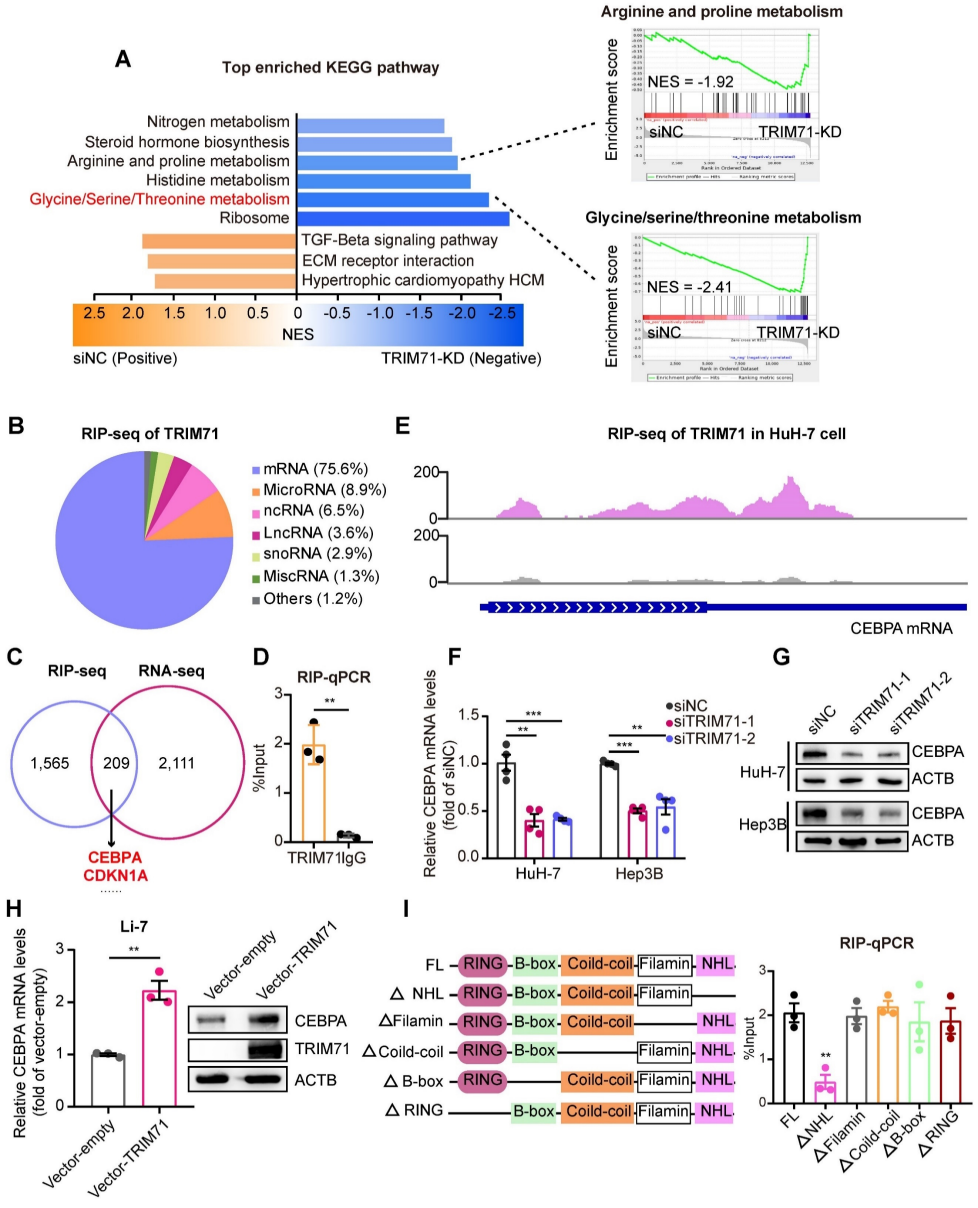
** TRIM71 controls metabolic pathways and CEBPA mRNA levels.** (A) KEGG pathway enrichment analysis of differentially expressed genes in HuH-7 cells transfected with siNC or siRNAs targeting TRIM71 (Left). GSEA showing down-regulated pathways after TRIM71 knockdown including glycine/serine/threonine metabolism pathway and arginine and proline metabolism (Right). (B) Pie chart showing transcript types binding to TRIM71 by RIP-seq analysis of HuH-7 cells. (C) Venn diagram showing the overlap between the differentially expressed transcripts in TRIM71-KD cells and transcripts bound by TRIM71. (D) RIP-qPCR analysis showing the dramatic enrichment of TRIM71 on CEBPA mRNA. (E) The binding peaks of TRIM71 on CEBPA mRNA in HuH-7 cells. (F) The CEBPA mRNA levels in HuH-7 and Hep3B cells transfected with siNC or siTRIM71 siRNAs. (G) Immunoblot analysis of CEBPA protein levels in HuH-7 and Hep3B cells transfected with siNC or siTRIM71 siRNAs. (H) The CEBPA mRNA levels and protein levels in Li-7 cells with infected with control or TRIM71 overexpression lentiviruses. (I) Schematic description of TRIM71 mutants (left). RIP-qPCR analysis showing enrichment of NHL domain of TRIM71 on CEBPA mRNA (right). Values represent the mean ± SEM. **P < 0.01, ***P < 0.001.

To explore the mechanism underlying the tumor-promoting effects of TRIM71, we transfected with siRNAs targeting TRIM71 and performed RNA-seq (**Table S2**). The differentially expressed genes in HuH-7 cells with TRIM71 knockdown (TRIM71-KD) are involved in cellular metabolism such as glycine/serine/threonine metabolism and arginine and proline metabolism (**Figure 4A**). As TRIM71 is an RNA-binding protein that exerts its molecular functions, we conducted RNA immunoprecipitation sequencing (RIP-seq) to identify the target transcripts bound by TRIM71. The results indicated that TRIM71 mainly binds to mRNA (75.6%), followed by microRNA (8.9%) and ncRNA (6.5%) (**Figure 4B, Table S3**). Through integrative analysis of TRIM71-KD RNA-seq data and TRIM71 RIP-seq data, we identified 209 transcripts whose expression level was regulated and bound by TRIM71, including a well-known target CDKN1A and the top enriched target CEBPA (**Figure 4C**).

CCAAT/enhancer binding protein alpha (CEBPA) is a transcription factor (TF) that plays a crucial role in regulating the proliferation of various cells, including hematopoietic cells 24. Accumulating evidence has suggested that CEBPA has a pro-tumorigenic role in liver cancers 25. By analyzing data from the Depmap database, we found a positive correlation between CEBPA mRNA expression and proliferative ability in liver cancer cell lines (**Figure S7A**). The similar positive correlation between CEBPA and TRIM71 on mRNA levels (**Figure S7B**) and proliferative ability (**Figure S7C**) was also confirmed. Then we explored the function role of CEBPA in liver cancer cells. CEPBA knockdown (**Figure S7D**) significantly inhibited colony formation ability in highly expressed CEBPA liver cancer cells (**Figure S7E**). Xenograft tumor mouse experiments (**Figure S7F**) revealed that CEBPA knockdown in HuH-7 cells dramatically decreased tumor volume (**Figure S7G**) and tumor weight (**Figure S7H**). These observations indicated that CEBPA is critical for liver cancer cell proliferation in vitro and in vivo.

Our RIP-qPCR data further verified the interaction between CEBPA mRNA with TRIM71 (**Figure 4D**), and binding sites of TRIM71 were enriched in the coding sequence (CDS) and 3' untranslated region (3'UTR) of CEBPA mRNA (**Figure 4E**). Inhibition of TRIM71 reduced mRNA (**Figure 4F**) and protein levels (**Figure 4G**) of CEBPA. Ectopic expression of TRIM71 also enhanced CEBPA mRNA and protein expression in Li-7 cells (**Figure 4H**). To further investigate which domain of TRIM71 is crucial for the interaction between TRIM71 and CEBPA mRNA, we constructed TRIM71 mutant vectors and then transfected mutants into HuH-7 cells. RIP-qPCR experiments revealed that TRIM71 deletion of the NHL domain significantly inhibits the binding of TRIM71 to CEBPA mRNA (**Figure 4I**), suggesting that TRIM71 regulates CEBPA expression via the NHL domain. These results indicate that TRIM71 regulates metabolic pathways and CEBPA mRNA levels in liver cancer.

### TRIM71/IGF2BP1 protein complex regulates CEBPA mRNA levels through m6A-dependent RNA stability

**Figure 5:**
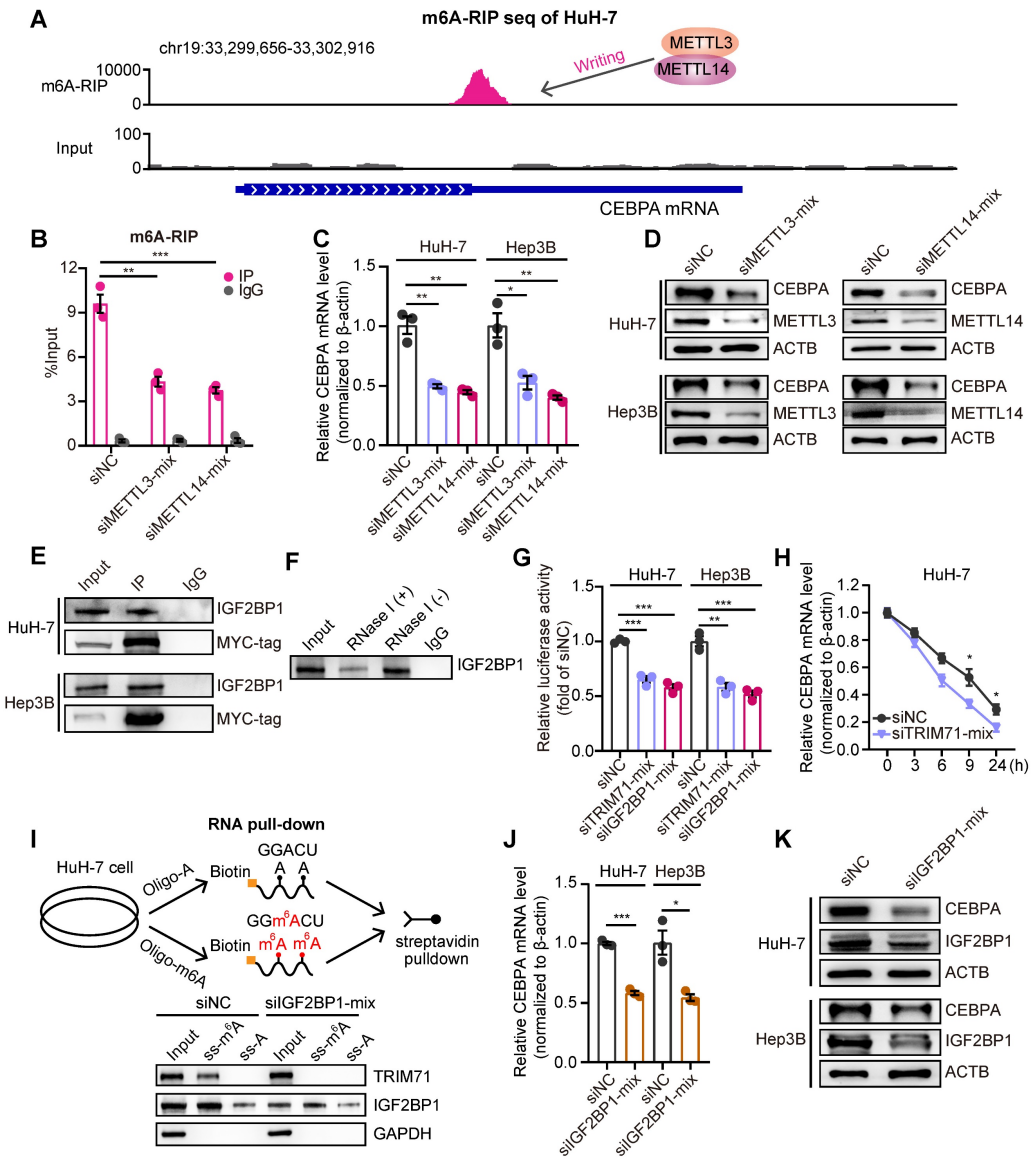
** TRIM71-IGF2BP1 protein complex stabilizes CEBPA mRNA in a m6A-dependent manner.** (A) m6A-RIP-seq data analysis showing the m6A modification signal were presented nearby stop codon region of CEBPA mRNA. (B) m6A-RIP-qPCR analysis showing the enrichment of m6A modification signal on CEBPA mRNA in HuH-7 cells transfected with siNC, siMETTL3 or siMETTL14 mixed siRNAs. (C) The CEBPA mRNA levels in HuH-7 and Hep3B cells transfected with siNC, siMETTL3 or siMETTL14 mixed siRNAs. (D) Immunoblot of CEBPA protein levels in HuH-7 and Hep3B cells transfected with siNC, siMETTL3 or siMETTL14 mixed siRNAs. (E) Co-IP assay showing the interaction between TRIM71 and IGF2BP1. (F) Co-IP assay showing the interaction between TRIM71 and IGF2BP1 with or without RNase I treatment in HuH-7 cells. (G) The relative luciferase activity in HuH-7 and Hep3B cells transfected with siNC, siTRIM71 or siIGF2BP1 mixed siRNAs. (H) The CEBPA mRNA levels in HuH-7 cells transfected with siNC or siTRIM71 mixed siRNAs at the indicated time point. (I) Schematic depicting RNA pull-down in HuH-7 cells using oligonucleotides containing with or without m6A modification in adenosine (upper). Immunoblot of TRIM71 and IGF2BP1 protein level in HuH-7 cells transfected with siNC or siIGF2BP1 mixed siRNAs (lower). (J) The CEBPA mRNA levels in HuH-7 and Hep3B cells transfected with siNC or siIGF2BP1 mixed siRNAs. (K) Immunoblot of CEBPA protein level in HuH-7 and Hep3B cells transfected with siNC or siIGF2BP1 mixed siRNAs. Values represent the mean ± SEM. *P<0.05, **P < 0.01, ***P < 0.001.

There is mounting evidence that N6-methyladenosine (m6A) modification is a critical post-transcriptional regulatory mechanism that modulates gene expression, and that dysregulation of m6A modification is associated with the pathogenesis and drug response in multiple types of tumors 26. m6A deposition on transcripts displays a regional bias, with a notable enrichment of m6A modification primarily occurring near the stop codon and within the 3'UTR in mature mRNA 27. Moreover, the RIP-seq analysis revealed that TRIM71 exhibits binding sites on the CEBPA mRNA near the stop codon and 3'UTR (**Figure 4E**). Hence, we hypothesized that TRIM71 regulates CEPBA expression through an m6A-dependent mechanism. To test this hypothesis, we performed m6A methylated RNA immunoprecipitation sequencing (MeRIP-seq) with HuH-7 cells and found significant enrichment of m6A signal nearby the stop codons of the CEBPA mRNA (**Figure 5A**). In order to further elucidate the regulatory role of m6A modification on the expression of CEBPA, we employed siRNA-mediated knockdown of the methyltransferases METTL3 and METTL14 (two well-known m6A writers) in HuH-7 cells to assess the abundance of m6A signal on CEBPA mRNA. The results showed a significant reduction in m6A enrichment on CEBPA mRNA upon silencing of METTL3 or METTL14 (**Figure 5B**), accompanied by downregulation of CEBPA mRNA (**Figure 5C**) and protein levels (**Figure 5D**).

The m6A “readers” exert their functions by recognizing and interacting with m6A-modified transcripts, leading to the modulation of downstream signaling pathways 28. The insulin-like growth factor mRNA-binding proteins (IGF2BPs; including IGF2BP1/2/3), a class of m6A readers, were reported to have a regulatory role in promoting the stability of mRNA targets in an m6A-dependent manner 29. We performed immunoprecipitation-mass spectrometry (IP-mass) to uncover the binding cofactors with TRIM71 in HuH-7 liver cancer cells. 107 proteins showed potential binding with TRIM71, including a high enrichment of IGF2BP1. Co-immunoprecipitation (co-IP) assays verified the interaction between TRIM71 and IGF2BP1 (**Figure 5E**). Therefore, we hypothesized that TRIM71-mediated regulation of CEBPA expression is contingent upon the recognition of m6A modifications by IGF2BP1. We further noticed that the interaction between TRIM71 and IGF2BP1 depends on CEBPA mRNAs (**Figure 5F**), suggesting their interaction may not be straightforward. We cloned the 3'UTR sequence and stop coding sequence with m6A modification of CEBPA mRNAs into pGL3 vectors. The luciferase reporter assay showed that the activity of the reporter containing m6A modification sequences of CEBPA was dramatically suppressed upon knockdown of TRIM71 or IGF2BP1 in HuH-7 and Hep3B cells (**Figure 5G**). Moreover, the inhibition of TRIM71 resulted in decreased mRNA stability of CEBPA (**Figure 5H**). To further confirm whether TRIM71 recognizes m6A modification of RNA is dependent on IGF2BP1, we constructed oligonucleotides with or without m6A modification and performed RNA pull-down assays. The results showed that both TRIM71 and IGF2BP1 interacted with m6A-modified oligonucleotides, whereas knockdown of IGF2BP1 suppressed TRIM71 binding to oligonucleotides with m6A modification (**Figure 5I**). In addition, silencing of IGF2BP1 distinctly reduced mRNA levels (**Figure 5J**) and protein levels (**Figure 5K**) of CEBPA in HuH-7 and Hep3B cells.

We previously confirmed that the NHL domain of TRIM71 is crucial for binding CEBPA mRNA. To determine whether the NHL domain regulates CEBPA expression and mediates the oncogenic function of TRIM71, we first infected liver cancer Li-7 cells with a TRIM71 lentiviruses lacking the NHL domain. The results showed that overexpression of the full-length TRIM71 significantly enhanced both RNA and protein levels of CEBPA, whereas overexpression of TRIM71 lacking the NHL domain did not increase CEBPA RNA and protein levels (**Figure S8A-B**), indicating that the NHL domain is essential for binding and regulating the expression of CEBPA. Subsequently, we transfected HuH-7 cells with plasmids overexpressing full-length TRIM71 and TRIM71 lacking the NHL domain. Co-IP experiments demonstrated that the full-length TRIM71 protein could bind to IGF2BP1, while deletion of the NHL domain significantly inhibited the binding of TRIM71 to IGF2BP1 (**Figure S8C**). This suggests that the NHL domain regulates the binding of TRIM71 to IGF2BP1, and this interaction may be mediated by CEBPA mRNA, consistent with the results shown in **Figure 5F**. Finally, we assessed whether the NHL domain mediates TRIM71's oncogenic function. Colony formation results indicated that overexpression of full-length TRIM71 significantly promoted the clonogenic capacity of liver cancer cells Li-7 and HCCLM3 with no or less expression of TRIM71. TRIM71 lacking the NHL domain also possessed oncogenic potential, but this function was significantly suppressed compared to the full-length TRIM71 (**Figure S8D**). These results might be due to other structural domains of TRIM71, such as the RING domain that possesses ubiquitin ligase activity, also contributing to TRIM71's oncogenic function.

We also transfected TRIM71 specific siRNAs in liver cancer cells and found that knockdown of TRIM71 had no significant effect on IGF2BP1 protein levels (**Figure S9A**). Moreover, the mRNA levels (**Figure S9B**) and protein levels (**Figure S9C**) of CEBPA were not altered when both knockdown of TRIM71 and IGF2BP1, compared with inhibition of IGF2BP1 alone, suggesting the m6A mediated RNA stability exerted by TRIM71 was dependent on IGF2BP1. We also tested the reported TRIM71 target CDKN1A/p21, a classic tumor suppressor, and found that knockdown of TRIM71 elevated CDKN1A mRNA levels (**Figure S9D**) and protein levels (**Figure S9E**). To further investigate the mRNA regulatory relationship between TRIM71 and CDKN1A in normal liver and liver cancer, we analyzed m6A-RIP seq of HuH-7 cells and found CDKN1A mRNA has no significant m6A modification (**Figure S9F**). We also performed TRIM71 overexpression experiments in hiHep normal liver cells 30 and Li-7 liver cancer cells. Our experimental findings demonstrated that overexpression of TRIM71 significantly inhibited the mRNA levels (**Figure S9G**) and RNA stability of CDKN1A (**Figure S9H**). These results suggest that TRIM71 promotes the degradation of CDKN1A mRNA lacking m6A modification. In tumors, TRIM71 stabilizes m6A-modified CEBPA mRNA dependent on IGF2BP1, and ultimately facilitates liver cancer progression.

### TRIM71-CEBPA controls serine/glycine metabolism in liver cancer cells

**Figure 6:**
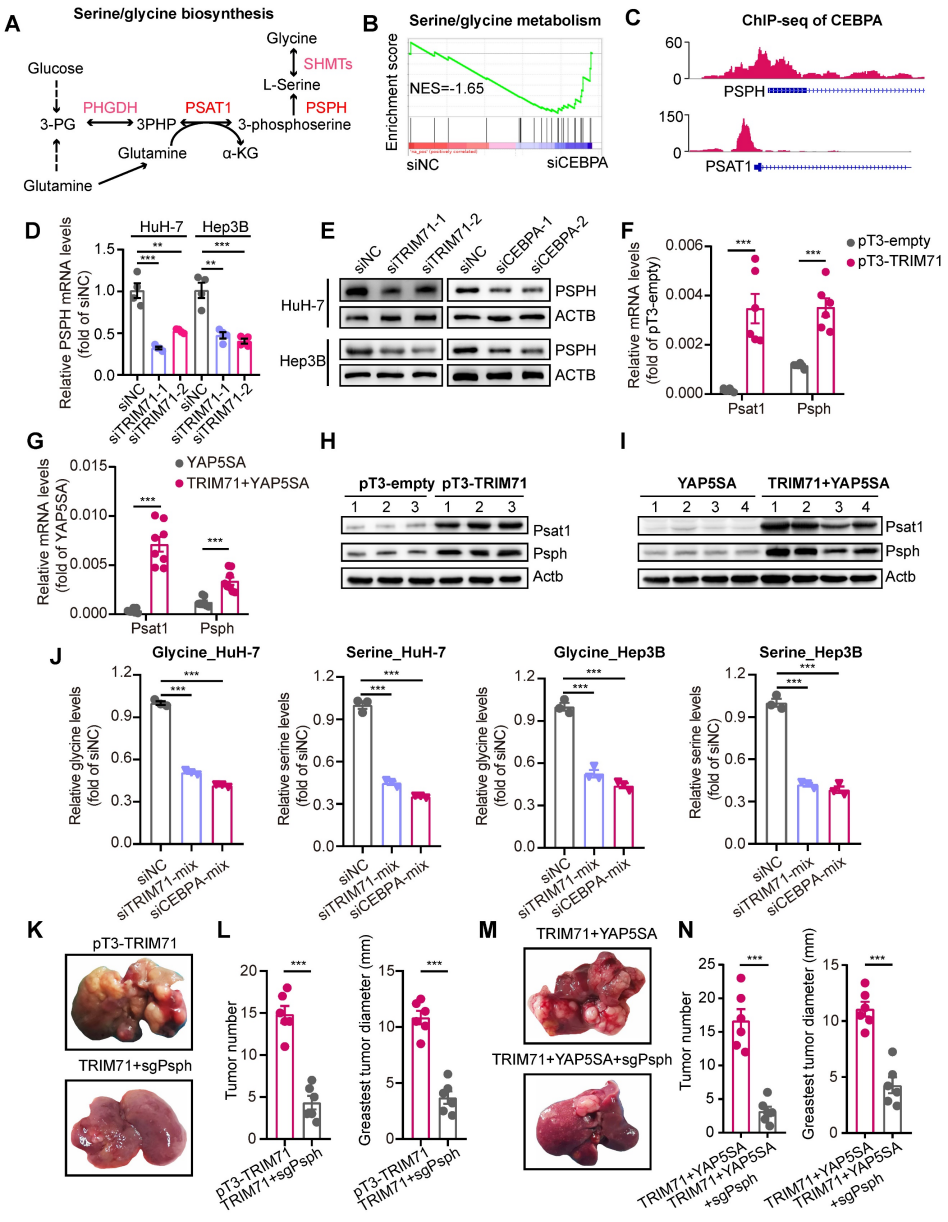
** “TRIM71-CEBPA” axis controls serine and glycine biosynthesis in liver cancer cells.** (A) Schematic diagram depicting serine and glycine biosynthesis pathways. (B) GSEA results showing the enrichment of serine/glycine biosynthesis pathway after CEBPA knockdown. (C) The binding peaks of CEBPA in PSPH and PSAT1 promoter regions. (D) The PSPH mRNA levels in HuH-7 and Hep3B cells transfected with siNC or siTRIM71 mixed siRNAs. (E) Immunoblot analysis of PSPH protein levels in HuH-7 and Hep3B cells transfected with siNC, siTRIM71 or siCEBPA mixed siRNAs. (F) The Psph and Psat1 mRNA levels in mouse liver cancer tissues of pT3-empty or pT3-TRIM71 models. (G) The Psph and Psat1 mRNA levels in mouse liver cancer tissues of YAP5SA or YAP5SA + TRIM71 models. (H) Immunoblot showing the Psph and Psat1 protein levels in mouse liver cancer tissues of pT3-empty or pT3-TRIM71 models. (I) Immunoblot showing the Psph and Psat1 protein levels in mouse liver cancer tissues of YAP5SA or YAP5SA + TRIM71 models. (J) The intracellular serine and glycine levels in HuH-7 and Hep3B cells transfected with siNC, siTRIM71 or siCEBPA mixed siRNAs. (K) Representative images of tumors dissected from liver tumor mouse models of pT3-TRIM71 or pT3-TRIM71combined with knocking down of Psph. (L) Tumor number and greatest tumor diameter in liver tumor mouse models of pT3-TRIM71 or pT3-TRIM71 combined with knocking down of Psph. (M) Representative images of tumors dissected from liver tumor mouse models of TRIM71 + YAP5SA or TRIM71 + YAP5SA combined with knocking down of Psph. (N) Tumor number and greatest tumor diameter in liver tumor mouse models of TRIM71 + YAP5SA or TRIM71 + YAP5SA combined with knocking down of Psph. Values represent the mean ± SEM. **P < 0.01, ***P < 0.001.

Studies have demonstrated that tumor cells exhibit an enhanced capacity for de novo serine synthesis, which generates precursor molecules for proteins, nucleic acids, and lipids, thereby facilitating diverse biological processes crucial for tumor cell growth and proliferation 31. Key enzymes involved in the serine/glycine biosynthesis pathway, including PSPH, have been identified as overexpressed in cancers, particularly in liver cancer 32, 33 (**Figure 6A**). As CEBPA is a downstream effector of TRIM71, we hypothesized the involvement of CEBPA in regulating the serine/glycine biosynthesis pathway. Firstly, we performed RNA-seq in control and CEBPA knockdown HuH-7 cells and found a significant enrichment of the serine/glycine metabolism pathway (**Figure 6B**; NES = -1.65) We also performed chromatin immunoprecipitation sequencing (ChIP-seq) of CEBPA and found significant enrichment in promoter regions of PSPH and PSAT1 (**Figure 6C**). Moreover, knockdown of TRIM71 significantly reduced mRNA and protein levels of PSPH (**Figure 6D-E**), and similar results were obtained with PSPH protein levels when CEBPA was knocked down (**Figure 6E**). Conversely, PSPH and PSAT1 mRNA levels (**Figure 6F-G**) and protein levels (**Figure 6H-I**) were dramatically upregulated in TRIM71 and YAP5SA + TRIM71 mice liver tumor tissues compared to the control group, and inhibition of TRIM71 or CEBPA decreased cellular serine and glycine levels in HuH-7 and Hep3B liver cancer cells (**Figure 6J**). To further elucidate the dependency of TRIM71 on the glycine/serine metabolism pathway in promoting liver cancer initiation and progression, we conducted knockdown experiments targeting the Psph gene (**Figure S10A**) in the previously two established TRIM71 mice models using hydrodynamic tail vein injection. The results revealed that Psph knockdown significantly suppressed the ability of TRIM71 alone (**Figure 6K-L**) or in combination with YAP5SA (**Figure 6M-N**) to induce liver cancer formation.

We also designed specific siRNA targeting PSPH and transfected it into liver cancer cells to further clarify its molecular functions in liver cancer. The results showed that knockdown of PSPH (**Figure S10B**) significantly inhibited the proliferative capacity of liver cancer cells in vitro (**Figure S10C-D**). In pT3-TRIM71 or TRIM71 + YAP5SA mouse models of liver cancer induced by TRIM71, we found that TRIM71 alone or in combination with YAP5SA significantly increased the oncofetal levels (**Figure S10E**), further suggesting that TRIM71 is a key regulatory factor in maintaining the oncofetal landscape of liver cancer. Furthermore, we also discovered that mRNA expression of PSPH and PSAT1 was significantly positively correlated with the oncofetal score in patients with HB and HCC (**Figure S10F-G**). At the same time, the expression of PSPH and PSAT1 mRNA in patients with HB and HCC was also significantly positively correlated with the expression of TRIM71 (**Figure S10H-I**). In summary, this data collectively indicates that TRIM71 is a key regulatory RNA-binding protein in the construction of oncofetal ecosystem of liver cancer. TRIM71 activates the glycine/serine metabolism pathway by remodeling the transcription of PSPH and PSAT1, thereby controlling the oncofetal characteristics of liver cancer and tumor initiation and progression.

### All-trans-retinoic acid combined with A-485 hamper serine and glycine biosynthesis in TRIM71 high-expressed liver cancer cells

**Figure 7:**
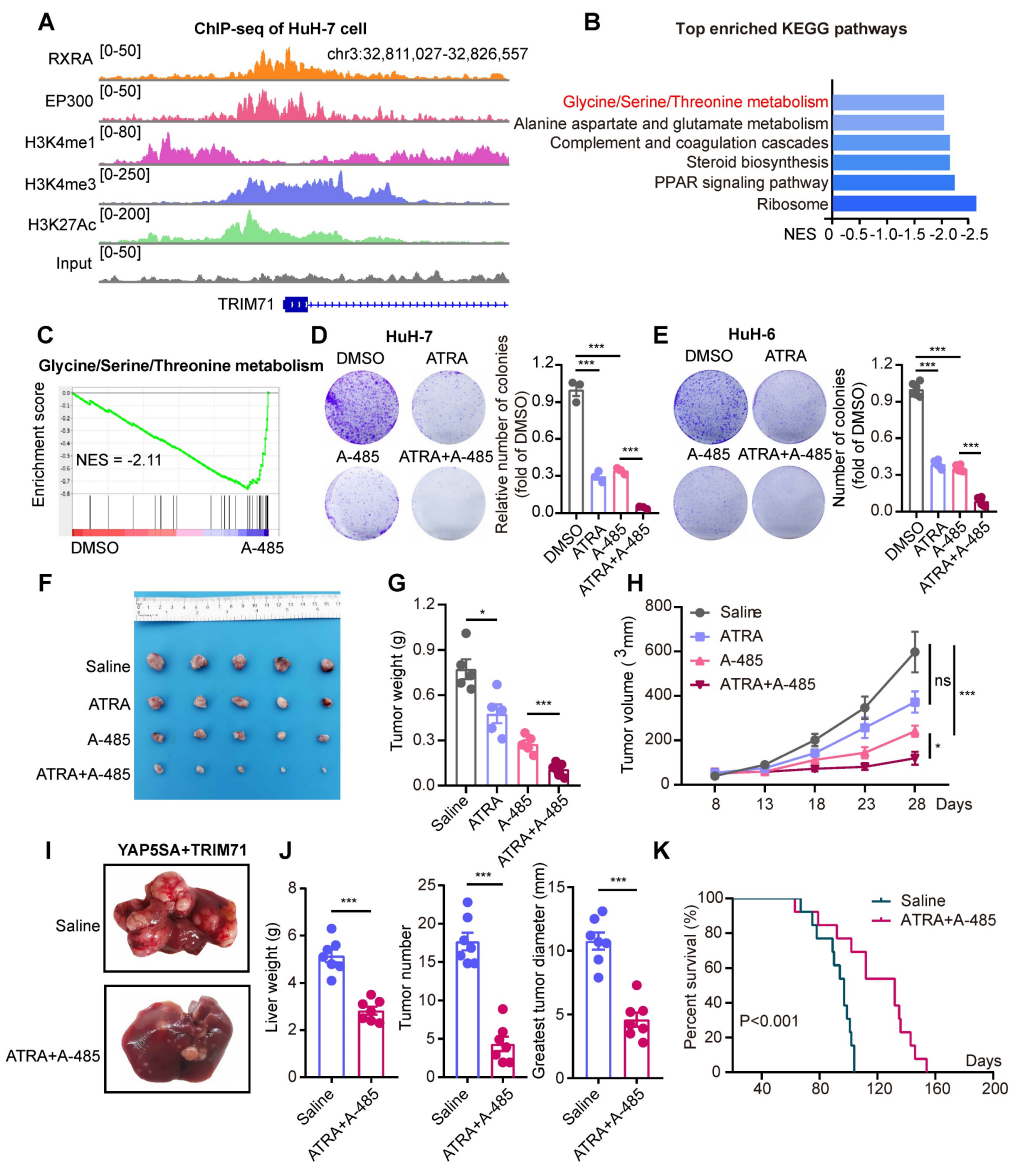
** ATRA combined with A-485 hamper serine and glycine biosynthesis in TRIM71/CEBPA high-expressed liver cancer cells.** (A) The ChIP-seq peaks of RXRA, EP300, H3K4me1, H3K4me3 and H3k27Ac in TRIM71 promoter regions of HuH-7 cells. (B) Top enriched KEGG pathway analysis of differentially expressed genes in HuH-7 cells treated with DMSO or A-485. (C) GSEA analysis displaying down-regulated pathways including Glycine/Serine/Threonine metabolism in HuH-7 cells treated with A-485. (D-E) The colony formation assay in HuH-7 (D) and HuH-6 (E) cells treated with DMSO, ATRA (2 μM/L), A-485 (1 μM/L), ATRA (2 μM/L) + A-485 (1 μM/L). (F) Images of the xenograft mouse models implanted with HuH-6 cells and treated with saline, ATRA (20 mg/kg), A-485 (10 mg/kg), ATRA (20 mg/kg) + A-485 (10 mg/kg). (G-H) Effects of saline, ATRA, A-485, ATRA + A-485 on the tumor weight (G) and tumor volume (H) of HuH-6 xenograft tumors. (I) Images of liver tumor mouse models (YAP5SA + TRIM71) using hydrodynamic tail-vein injection and treated with saline or ATRA (20 mg/kg) + A-485 (10 mg/kg). (J) Liver weight, tumor number and greatest tumor diameter in YAP5SA + TRIM71 liver tumor mouse models treated with saline or ATRA (20 mg/kg) + A-485 (10 mg/kg). (K) Survival curve of YAP5SA + TRIM71 liver tumor mouse models treated with saline or ATRA (20 mg/kg) + A-485 (10 mg/kg). Values represent the mean ± SEM. ns: no significance. *P<0.05, ***P < 0.001.

To elucidate the molecular mechanisms underlying the overexpression of TRIM71 in liver cancer cells, we analyzed potential regulators binding on TRIM71 promoter regions using ENCODE database, and unexpectedly discovered the potential binding of retinoid X receptor alpha (RXRA) factor and EP300. ATRA, a metabolic product of vitamin A, binds to retinoic acid receptors (RARs), which form heterodimers with RXRA to function as transcription factors and regulate gene expression 34. RARs play a vital role in development and are also crucial in promoting the differentiation of embryonic stem cells (ESCs) 35. Meanwhile, EP300 encoded p300 served as transcriptional co-activators in many cancers and control glycine/serine metabolism 36, 37. As TRIM71 is vital for maintaining the dedifferentiation of stem cells and regulate glycine/serine metabolism, we hypothesized the potential regulation of RXRA and EP300 on TRIM71 in liver cancers. To verify the potential regulation to TRIM71 transcription, we performed ChIP-seq of RXRA, p300 and active histone modification marks (H3K4me1, H3K4me3, and H3K27Ac) at the TRIM71 gene locus in HuH-7 cells. Interestingly, we found the enrichment of RXRA and p300 in TRIM71 promoter regions (**Figure 7A**). Treated with ATRA or p300 specific inhibitor A-485 significantly repressed glycine/serine metabolism pathway in HuH-7 cells (**Figure 7B-C, Figure S11A-B**), and similar inhibition of this pathway was found in HuH-6 cells treated with A-485 (**Figure S11C**).

ATRA has been reported as a crucial and clinical therapeutic strategy for the treatment of acute promyelocytic leukemia (APL) by inducing differentiation of myeloid cells, as well as for its anti-tumor functions in other tumors through enhancing sensitivity of cancer treatment 34, 38. ATRA has also been implicated with anti-proliferative ability with other drugs in liver cancer 39, and it alone in liver cancer has a limited anti-tumor effect in vivo 40. As ATRA and A-485 could both inhibit TRIM71 expression and glycine/serine metabolism pathway, we hypothesized ATRA combined with A-485 could attenuate liver cancer initiation and progression with TRIM71 high expression. We firstly tested sensitivity of A-485 in liver cancer cell lines and found cells with higher proliferative ability like HuH-6 and Huh-7 are relatively sensitive to A-485 (**Figure S11D**).

Importantly, ATRA or A-485 decreased mRNA levels of TRIM71, CEBPA, PSPH, PSAT1 (**Figure S11E**) and protein levels of TRIM71 and CEBPA (**Figure S11F**), suggesting the transcriptional regulation of RXRA and EP300 to TRIM71 in liver cancer. ATRA or A-485 alone could decrease colony number formation of TRIM71 high expressed HuH-7 (**Figure 7D**) and HuH-6 (**Figure 7E**) cells in vitro, and the combination of ATRA and A-485 almost completely abolished the growth of HuH-7 and HuH-6 cells. The anti-tumor ability of ATRA alone was limited using subcutaneous xenograft mouse models, which might be the short half-life of ATRA. Importantly, ATRA combined with A-485 could enhance the inhibition of liver cancer cell proliferation with high TRIM71 (**Figure 7F-H**). To test the anti-tumor function targeting liver cancer initiation, we constructed YAP5SA + TRIM71 mouse model and treated with ATRA combined with A-458. Surprisingly, ATRA combined with A-458 significantly decreased liver cancer formation (**Figure 7I-J**), and prolonged survival time (**Figure 7K**). Taken together, these findings suggest that ATRA combined with A-458 may serve as a potential therapeutic strategy for TRIM71 high-expressed liver cancer patients.

## Discussion

**Figure 8:**
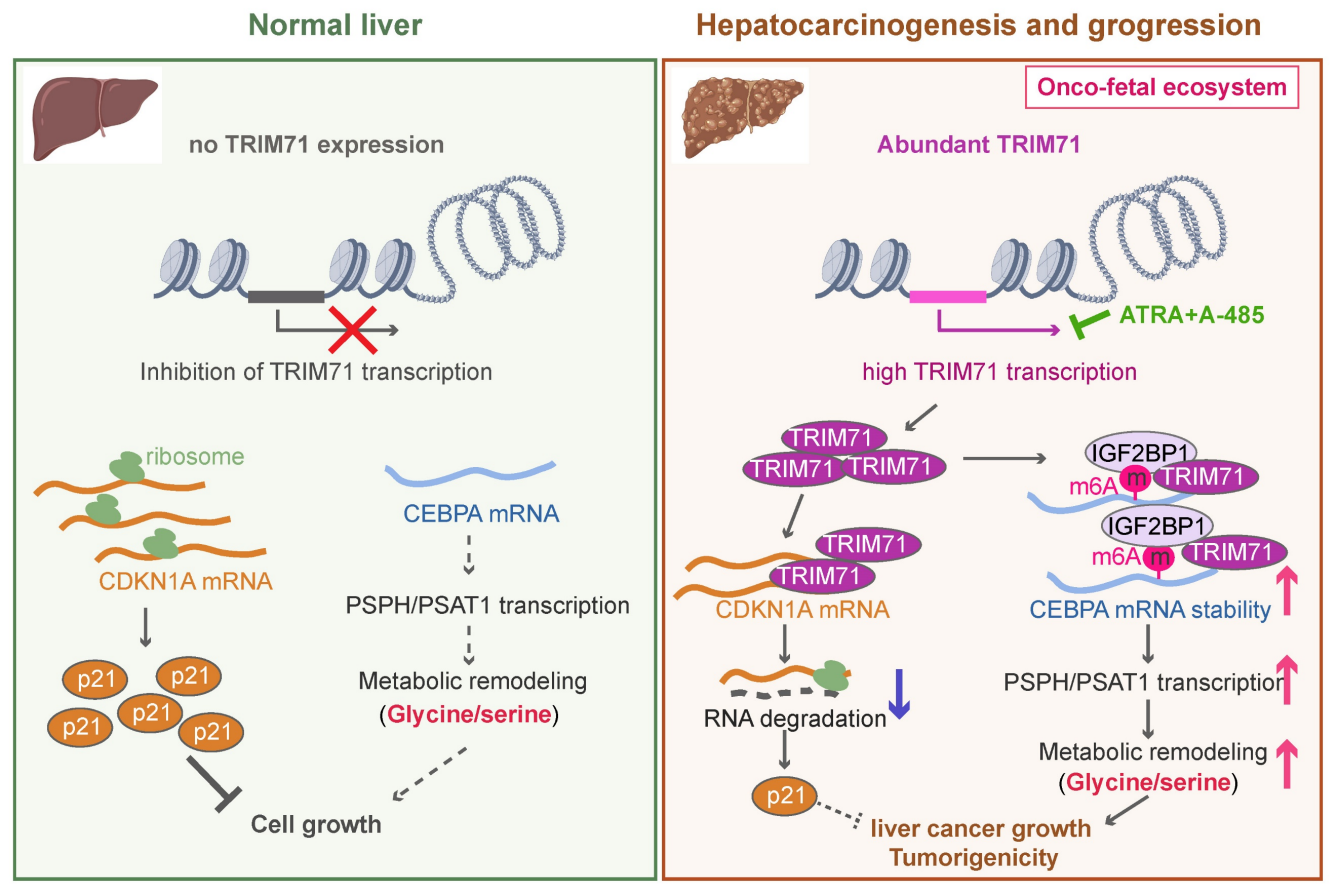
** The working model of TRIM71 in manipulating liver cancer initiation and progression.** In normal liver, the transcription of TRIM71 is inhibited, which accompanied by the low CEBPA mRNA levels and high CDKN1A mRNA levels. CDKN1A translates abundant p21 proteins and glycine/serine metabolism is inhibited, resulted in restricted cell growth (Left). In liver cancer, TRIM71 is specifically and highly expressed, and established the oncofetal ecosystem. TRIM71 degradates CDKN1A mRNA to decrease p21 protein. More importantly, TRIM71 forms protein complex with IGF2BP1, which binds to and stabilize CEBPA mRNA levels through m6A dependent manner. High expression of CEBPA remodels glycine/serine metabolism through enhancing PSPH/PSAT1 transcription, and ultimately promotes liver cancer growth and tumorigenicity. Targeting inhibition of TRIM71 using ATRA combined with A-485 may offer potentially therapeutic strategies for liver cancer patients with high TRIM71 levels (Right).

In this study, we demonstrate the existence of an oncofetal status in liver cancer and the importance of TRIM71 in liver cancer initiation and progression. Our data show that liver cancer establishes an oncofetal ecosystem that facilitates more aggressive ability. We have identified TRIM71 as a potential regulator for the oncofetal ecosystem and a liver cancer-specific driver in manipulating cancer initiation and progression. TRIM71 promotes CDKN1A mRNA degradation with no m6A modification. Importantly, it forms a protein complex with IGF2BP1, which binds to and stabilizes CEBPA mRNA levels through an m6A-dependent manner, enhancing CEBPA mediated PSPH/PSAT1 transcription and remodeling serine/glycine metabolism, and ultimately accelerates liver cancer growth and tumorigenicity. Targeting the inhibition of TRIM71 using retinoic acid combined with A-485 may offer potentially therapeutic strategies for liver cancer patients with high TRIM71 levels (**Figure 8**).

Tumors often reshape their epigenome and other layers to overexpress oncogenes related to tumor stem cells, which maintain the low differentiation of tumors, and further strengthen their highly malignant characteristics. Recent studies have reported that pediatric renal cell carcinoma exhibits features of aberrant fetal cells with faulty wilms tumor 1 (WT1) expression during adult homeostasis, re-emphasizing the link between developmental anomalies and tumors 41. Importantly, Sharma et al. found the fetal-like reprogramming of the tumor microenvironment in HCC, including the re-emergence of fetal-associated endothelial cells (PLVAP/VEGFR2) and fetal-like (FOLR2) tumor-associated macrophages 4. Encouraged by these outstanding results, we further systematically investigated the characteristics of epithelial cells (tumor cells) of liver cancers, including HCC, ICC, and HB. Surprisingly, these tumor cells also exhibited an oncofetal status, which is more enriched in HCC and HB. HCC patients with a high oncofetal score displayed worse prognosis, suggesting that oncofetal features may promote the progression of HCC.

To further identify the core factors that potentially maintain the oncofetal status of liver tumor cells, we analyzed the overexpressed genes in these tumor cells. Surprisingly, nearly 30% of up-regulated genes belong to the RBP family, including TRIM71, LIN28B, and IGF2BP1. Recent evidence has revealed that LIN28B is a key oncofetal RBP and is sufficient to drive hepatocarcinogenesis 15, suggesting the importance of RBPs in building the oncofetal system of liver cancer cells. In addition to the classic oncofetal gene of LIN28B, we also found a more specific RBP, TRIM71, which is one of the top enriched genes in oncofetal liver cancer cells. Overexpression of TRIM71 promotes reprogramming and is important for overcoming the let-7 barrier to reprogramming human iPSC 23. Recent evidence has revealed that TRIM71 is involved in liver cancer progression 42. However, the detailed roles and molecular functions of TRIM71 need to be clarified in liver cancer. Through analyzing the GEPIA database we found that TRIM71 is overexpressed in HCC, acute myeloid leukemia (AML), testicular germ cell tumors (TGCT), and ovarian cancer (OC). We also found a positive correlation between TRIM71 mRNA levels and its proliferative ability. Moreover, among nearly 900 tumor cell lines from the Depmap database, four of the top 10 cell lines associated with the proliferative ability of TRIM71 were liver cancer cell lines, and the cell line with the most significant involvement of TRIM71 in tumor cell proliferation was HuH-7 liver cancer cells, suggesting that TRIM71 is potentially a liver cancer-specific oncogene. We further identified that TRIM71 controls liver cancer cell proliferation and drives hepatocarcinogenesis in vitro and in vivo, which strengthens the importance of TRIM71 in liver cancer initiation and progression. As a significant part of the potential regulatory factors are RBPs, we will further clarify how these RBPs remodel the transcriptome and maintain the oncofetal system in liver cancers.

In the initial study, TRIM71 was identified as a regulator of the microRNA world, acting as a suppressor of phenotypes caused by a microRNA let-7 loss-of-function mutation and a regulator of temporal cell fates in C. elegans larvae 43. Further studies confirmed that TRIM71 also binds to mRNA and long non-coding RNAs, promoting mRNA degradation like CDKN1A/p21 through cooperation with nonsense-mediated decay (NMD) factors and maintaining embryonic stem cell characteristics 44, 45. However, Foster et al. identified that TRIM71 is capable of positive and negative regulation of target RNAs in addition to its function as an RNA suppressor molecule 46. In our work, we performed IP-mass and co-IP assays and identified the protein complex formed by TRIM71 with IGF2BP1, which stabilized CEBPA mRNA levels through an m6A-dependent manner in liver cancers. We also found elevated expression of CDKN1A when TRIM71 was knocked down, suggesting the existence of degradation of TRIM71 to CDKN1A mRNA with no m6A modification. Recently, studies have shown that TRIM71, as well as IGF2BP1, are located in p-bodies, strengthening the potential binding between them to dynamically regulate RNA stability with/without m6A modification 47.

Studies on the serine/glycine metabolic pathway have revealed its critical role in generating biomass, energy, and intracellular reductants required for cancer cell growth and proliferation, thus supports tumorigenesis and progression 1, 31. In liver cancer, Wang et al. found that serine levels are abnormally elevated in HCC tissues. Liver cancer cells use protein arginine methyltransferase PRMT1 to PHGDH and enhance its catalytic activity, thereby enhancing serine synthesis, reducing oxidative stress, and ultimately promoting HCC growth and malignant progression 48, 49. Targeting glycine/serine metabolic pathway exerts promising therapeutic potential in liver cancer using small molecules 37. Recently, Li et al. found that TRIM71 and the transcription factor CEBPA also belong to metabolism-related genes, and are crucial genes essential for the survival of liver cancer cells 50. In this study, we have discovered for the first time that TRIM71, as an RNA-binding protein, remodels the serine/glycine metabolic pathway in liver cancer, thereby controlling its malignant progression. This new regulatory mechanism provides a fresh perspective and critical regulatory targets for understanding how TRIM71 remodels liver cancer metabolism. Interestingly, in tumor tissues from mice models of hydrodynamic tail-vein injection induced by TRIM71, oncofetal-like ecological characteristics are activated, along with the upregulation of key serine/glycine regulatory genes Psph and Psat1. In large-scale samples of human HCC and HB tumors, we also observed a strong positive correlation between PSPH and PSAT1 and oncofetal scores, suggesting that serine/glycine metabolism may play a crucial role in the construction of the oncofetal ecological landscape in liver cancer, and that this process is closely regulated by TRIM71. Moving forward, we will continue to clarify the metabolic remodeling within the oncofetal ecological landscape and explore new potential regulatory mechanisms.

Our study highlights the potential inhibition of ATRA to TRIM71 transcription in liver cancer. Treatment with ATRA significantly decreased TRIM71 expression, leading to the inhibition of colony formation ability in liver cancer cells with high TRIM71 expression but with little effect on TRIM71-negative liver cancer cells, indicating that clinical ATRA treatment should consider the oncofetal characteristics and high TRIM71 expression of liver cancer patients. We further found limited anti-tumor ability of ATRA alone in vivo, consistent with previous experimental results 40. This may be the short half-life of ATRA 51. Interestingly, both ATRA and EP300 inhibitor A-485 could decrease TRIM71 and CEBPA expression and dampen glycine/serine metabolism, and ATRA could enhance anti-tumor ability of A-485 in liver cancer. ATRA may disrupt the onco-fetal ecosystem and change tumor characteristics such as tumor aging, and on this basis, synergistic effect with A-485 is produced to further improve the therapeutic effect. We will continue to explore the therapeutic range and potential of ATRA combined with A-485 drugs in patients with liver cancer. Furthermore, small molecule cocktails therapy containing ATRA has been proved to have excellent potential for the treatment of liver cancer 39. We will further explore the therapeutic effect of the cocktail therapy including ATRA combined with A-485 in the treatment of liver cancer patients with high expression of TRIM71.

In summary, we have described the oncofetal characteristics of liver cancers and identified TRIM71 as a liver cancer-specific driver and biomarker. TRIM71 forms a protein complex with IGF2BP1, enhances mRNA stability of CEBPA with m6A-dependent manner, remodels PSPH and PSAT1 transcription, strengthens serine/glycine metabolism, and ultimately promotes liver cancer progression. Based on our preclinical results, we suggest that ATRA combined with A-485 exert anti-tumor ability through repressing TRIM71/CEBPA expression and decrease serine/glycine metabolism in liver cancer. Our findings not only expand our understanding of oncofetal characteristics and related possible regulators but also provide a potential therapeutic option for liver cancer patients with high TRIM71 expression.

## Supplementary Material

Supplementary materials and methods, figures and tables.

## Abbreviations

scRNA-seq: single cell RNA-sequncing
RBPs: RNA-binding proteins
HCC: hepatocellular carcinoma
ATRA: all-trans-retinoic acid
EP300: e1A binding protein p300
ECs: endothelial cells
HB: hepatoblastoma
GSEA: gene set enrichment analysis
HBV: hepatitis B virus
inferCNV: infer copy number variations
CNV: copy number variations
ICC: intrahepatic cholangiocarcinoma
CCLE: cancer cell line encyclopedia
TRIM71: tripartite motif-containing 71
iPSC: induced pluripotent stem cell
CRISPR/Cas9: clustered regularly interspaced short palindromic repeats/Cas9
CCK-8: cell-counting kit 8
SB: sleeping beauty
HE: hematoxylin-eosin
IHC: immunohistochemistry
Afp: α-fetoprotein
TRIM71-KD: TRIM71 knockdown
RIP-seq: RNA immunoprecipitation sequencing
CEBPA: CCAAT/enhancer binding protein alpha
TF: transcription factor
CDS: coding sequence
3'UTR: 3' untranslated region
m6A: N6-methyladenosine
MeRIP-seq: m6A methylated RNA immunoprecipitation sequencing
IP-mass: immunoprecipitation-mass spectrometry
ChIP-seq: chromatin immunoprecipitation sequencing
co-IP: co-immunoprecipitation
RXRA: retinoid X receptor alpha
RARs: retinoic acid receptors
ESCs: embryonic stem cells
APL: acute promyelocytic leukemia
WT1: wilms tumor 1
AML: acute myeloid leukemia
TGCT: testicular germ cell tumors
OC: ovarian cancer
NMD: nonsense-mediated decay
