# Supplementary material for: Oncofetal TRIM71 drives liver cancer carcinogenesis through remodeling CEBPA-mediated serine/glycine metabolism
Source: Theranostics. 2024 Aug 12;14(13):4948–66. doi: 10.7150/thno.99633 (PMC11388079; doi:10.7150/thno.99633)
Supplement: Supplementary file 1 — Supplementary materials and methods, figures and tables. [file thnov14p4948s1.pdf]

Oncofetal TRIM71 drives liver cancer carcinogenesis through remodeling CEBPA-mediated serine/glycine metabolism

Ying Su[1, 2, *], Ziteng Li[2, *], Qin Li[2, *], Xinyi Guo[1, 2], Hena Zhang[1, 2], Yan Li[1, 2], Zhiqiang Meng[1, 2, †], Shenglin Huang[1, 2, †], Zhixiang Hu[1, 2, †]

[1]Department of Integrative Oncology, Fudan University Shanghai Cancer Center, and Shanghai Key Laboratory of Medical Epigenetics, International Co-laboratory of Medical Epigenetics and Metabolism, Institutes of Biomedical Sciences, Shanghai Medical College, Fudan University, Shanghai, China.

[2]Department of Oncology, Shanghai Medical College, Fudan University, Shanghai, China.

* These authors contributed equally to this work.

† **Correspondence**: Zhixiang Hu, Ph.D., Email: zhixiang_hu@fudan.edu.cn; Shenglin Huang, Ph.D., Email: slhuang@fudan.edu.cn; or Zhiqiang Meng, M.D., Email: mengshca@fudan.edu.cn. Department of Integrative Oncology, Fudan University Shanghai Cancer Center, and Shanghai Key Laboratory of Medical Epigenetics, International Co-laboratory of Medical Epigenetics and Metabolism, Institutes of Biomedical Sciences, Shanghai Medical College, Fudan University, 305 Rm., 7# Bldg., 270 Dong An Rd., Shanghai, 200032, China;

Materials and Methods

Cell culture and chemical reagents

HuH-7, HuH-6, Hep3B, HepG2, SNU-449, MHCC97H, HCCLM3, Li-7, HEK-293T cells were purchased from Shanghai Cell Bank Type Culture Collection (Shanghai, China) and cultured in DMEM (BasalMedia, L110KJ, Shanghai, China) culture media supplemented with 10% FBS (ExCell Bio, FSP500, Shanghai, China), 1% penicillin-streptomycin (BasalMedia, S110JV). All cell lines were grown in incubator at 37 ℃ and 5% CO_2 and were authenticated by short tandem repeats (STR) sequencing. Routine mycoplasma testing was carried out to ensure cells free of contamination before proceeding with further experiments. Retinoic acid was purchased from MedChemExpress (HY-14649, Shanghai, China) and used at a concentration of 2 μM for cells treatment. For in vitro spheres formation assay, 6,000 Li-7 liver cancer cells infected with PCDH-empty or PCDH-TRIM71 lentiviruses were seeded in flat-bottom ultralow attachment 6-well plates (Corning, NY, USA) and cultured in DMEM/F12 (11320033, ThermoFisher, IL, USA) supplemented with B-27 supplement (17504044, 1 : 50; Thermo Fisher Scientific) and 20 ng/mL of EGF (315-09, PeproTech) and FGF (100-17A, PeproTech). Hihep liver cells were obtained from professor Lijian Hui (Chinese Academy of Sciences Center for Excellence in Molecular Cell Science, Shanghai, China) and cultured in DMEM/F12 (11320033, ThermoFisher) culture media supplemented with 1% FBS, 1% penicillin-streptomycin, Galactose (G0050000, 2 g/L, sigma), Ornithine (A430329-0200, 0.1 g/L, sangon), L-Proline (A600923-0100, 0.3 g/L, sangon), Nicotinamide (A510659-0250, 0.61 g/L, sigma), ZnCl2 (208086, 0.544 mg/L, sigma), ZnSO4·7H2O (A602906-0500, 0.75 mg/L, sangon), CuSO4·5H2O (A600063-0500, 0.2 mg/L, sangon), MnSO4 (A600248-0500, 0.025 mg/L, sangon), ITS-A Supplement (51300044, ThermoFisher), TGF-α (100-16A, 40 ng/mL, PeproTech), EGF (315-09, 40 ng/mL, PeproTech), Dexamethasone (A429708-0500, 10 μM, sangon).

Antibodies

The antibodies against TRIM71 (sc-393352, WB) were purchased from Santa Cruz (USA); CEBPA (8178, ChIP-seq, WB), Myc-tag (2276S, WB, co-IP, IHC), Mouse Anti-rabbit IgG (Conformation Specific) (L27A9) mAb (HRP Conjugate; 5127s, WB) were purchased from Cell Signaling Technology (Danvers, MA, USA); AFP (14550-

1-AP, IHC) , MKI67 (GB111141-50, IHC), CD34 (GB113798-50, IHC), CK7 (GB115695, IHC), CK19 (GB15198, IHC), ARG1 (GB11285, IHC), CTNNB1 (GB15016, IHC) were from Servicebio (Shanghai, China); IGF2BP1 (22803-1-AP, WB, co-IP), PSPH (14513-1-AP, WB), PSAT1 (10501-1-AP, WB), ACTB (20536-1-AP, WB), METTL3 (15073-1-AP, WB), METTL14 (26158-1-AP, WB), GAPDH (60004-1-Ig, WB). HRP-conjugated Affinipure Goat Anti-Mouse IgG(H + L) (SA00001-1, WB), HRP-conjugated Affinipure Goat Anti-Rabbit IgG(H + L) (SA00001-2, WB), CD133 (18470-1-AP, WB), CD24 (10600-1-AP, WB), EPCAM (21050-1-AP), GPC3 (30021-1-AP, IHC) were from Proteintech (Wuhan, China), IGF2BP1 (A22612, WB, co-IP), CEBPA (A25033, WB) were from ABclonal (Wuhan, China), m6A antibody was from Sigma (ABE572).

Plasmid construction

The open reading frame (ORF) of TRIM71 with a Myc-tag and a 6x His-tag at the C terminus were amplified and subcloned into pCDH-puro vector (SBI, Palo Alto, CA, USA) using ClonExpress Ultra One Step Cloning Kit (Vazyme, C115-02, Nanjing, China). The pT3 vector, pCMV(CAT)T7-SB100 and pX330-U6-Chimeric_BB-CBh-hSpCas9 were kindly provided by professor Yongzhong Liu (Shanghai Jiaotong University, Shanghai, China). The fragments of the truncated TRIM71, including ΔNHL, ΔFilamin, ΔCoild-coil, ΔB-Box and ΔRING, were amplified based on pCDH-TRIM71-Myc-6xHis and inserted into pCDH vector. The CDS and 3'UTR regions of CEBPA containing m6A modification were amplified from HuH-7 genomic DNA and subclone into pGL3 basic vector. The CDS of YAP5SA with a Myc-tag at the N terminus were produced from plasmid encoding YAP5SA kindly provided by professor Faxing Yu (Fudan University, Shanghai, China), and constructed into pT3 vector. The sgRNA against TRIM71 and CEBPA were inserted into lentiGuide-Puro vector (a generous gift from Dr. Feng Zhang). The sgRNA sequences targeting Psph were inserted into pX330 vector. The primer sequences for all vectors' construction are provided in Table S4.

siRNA transfection

All the siRNAs were ordered from Ribobio (Guangzhou, China) and the sequences used in the study are listed in Table S4. Liver cancer cells were seeded in 6-well plates to allow 30% confluency in the next day. Negative control siNC and siRNAs

against target genes (TRIM71, CEBPA, METTL3, METTL14, IGF2BP1, PSPH) were transfected into cells using RNAimax (Thermo, 13778150). siRNA was utilized at a working concentration of 50 nM. Cells were harvested at 48 h post-transfection and then performed qPCR analysis or immunoblot to evaluate knockdown efficiency of target genes.

RNA isolation, cDNA synthesis, qRT-PCR

Total RNA was isolated from cultured cells and tumor tissues with TRIzol reagents (Thermo, 15596018, USA) following the manufacturer's protocols. RNA concentration was determined using a NanoDrop spectrometer (Thermo). To obtain complementary DNA, 0.5 μg total RNA was used for reverse transcription with RT Master mix (Accurate Biology, AG11706, China) according to the manufacturer's guidelines. Quantitative real-time PCR (qRT-PCR) was performed using SYBR Green Pro Taq HS Premix (Accurate Biology, AG11701) on ABI 7900HT Fast Real-Time PCR System (Thermo). β-actin was set as loading control for gene expression evaluation. Primers used in the qRT-PCR analysis are listed in Table S4.

RIP-seq and RIP-qPCR

RIP experiments were performed with HuH-7 cells stably expressing pCDH-puro-TRIM71-Myc. Cells were seeded in a 10 cm dish and collected when cells reached 80% confluence, washed two times with cold PBS and centrifuged at 1,000 g for 3 min at 4 °C. The cell pellets were resuspended in 1 mL RIP lysis buffer (20 mM Tris pH 7.4, 150 mM NaCl, 0.2% NP40, 1 mM EDTA, 20% glycerol) supplemented with protease inhibitor cocktail and RNase inhibitors, and lysed on ice for 30 min. The lysate was cleared by centrifuging at 17, 000g for 10 min at 4 °C. A part of the volume of the supernatant was set aside as input and mixed with 800 μL Trizol reagent. The remaining cell lysate were incubated with protein G magnetic beads pre-conjugated with anti-Myc-tag antibody or corresponding control IgG at 4 °C overnight. After incubation, the RNA-protein-bead complexes were washed with cold NT2 buffer (20 mM Tris–HCl pH 7.5, 150 mM NaCl, 1 mM $MgCl_2$, 0.05% NP40) four times at 4 °C for 5 min, and mixed with 800 μL Trizol reagent. RNA from the input and immunoprecipitation samples was extracted with Trizol reagent referring to manufacturer's protocol and concentration of RNA were determined by NanoDrop spectrometer. For RIP-seq assay, libraries were prepared using NEB Next Ultra

Directional RNA Library Prep kit (New England Biolabs, Beverly, MA, USA) following the manufacturer's protocol and sequenced on an Illumina Novaseq 6000 sequencer. For RIP-qPCR assay, equal volumes of RNA samples were subjected to reverse transcription to synthesize cDNA, followed by appropriate dilution for qPCR detection of the enrichment signal of TRIM71 on CEBPA mRNA. The RIP relative enrichment ratio was calculated by comparing the signal values of the Myc-tag group and IgG group to input sample. Primers used in the RIP-qPCR assay are listed in Table S4.

MeRIP-seq and MeRIP-qPCR

Total RNA from HuH-7 cells was extracted using TRIzol reagent referring to manufacturer's protocol. The obtained RNA was subjected to DNase I treatment at 37 °C for 15 min to eliminate potential DNA contamination. Then, the total RNA was fragmented to approximately 200 nucleotides in length via metal-ion-induced fragmentation. Protein-G magnetic beads were washed three times with IP buffer (20 mM Tris–HCl pH 7.5, 150 mM NaCl, 0.1% NP40) and incubated with anti-m6A antibody (Sigma, ABE572) in 200 µL IP buffer at room temperature for 1 h. The antibody-conjugated protein G beads were washed three times with 500 µL IP buffer and incubated with the supernatant containing fragmented total RNA supplemented with RNase Inhibitor at 4 °C for overnight. After incubation, the RNA-protein-bead complexes were washed in 500 µL IP buffer two times at 4 °C for 5 min and then washed twice in high-salt IP buffer (20 mM Tris–HCl pH 7.5, 500 mM NaCl, 0.1% NP40) at 4 °C for 5 min. Immunoprecipitated RNA fragments was eluted from beads using RNeasy Mini Kit (Qiagen, Valencia, CA, USA). For MeRIP-seq, libraries were prepared with RNA fragments from input and IP samples by smart-seq method and sequenced an Illumina NovaSeq 6000 sequencer with the paired-end 150 bp read option. For m6A RIP-qPCR, an equivalent volume of RNA samples was employed for reverse transcription to generate cDNA. Next, appropriate dilution of the cDNA was carried out for subsequent qPCR analysis. The relative abundance of m6A on CEBPA mRNA in HuH-7 cells expressing siNC, siMETTL3 or siMETTL14, was determined by normalizing to input. Primers used in the MeRIP-qPCR experiment are listed in Table S4.

RNA-sequencing (RNA-seq)

For RNA-seq, total RNA extraction was carried out using TRIzol reagent referring to the manufacturer's protocol. Before RNA library construction, rRNAs were removed using the RiboMinus Eukaryote kit (Qiagen, Valencia, CA, USA). RNA libraries were obtained using the NEB Next Ultra Directional RNA Library Prep kit (New England Biolabs, Beverly, MA, USA). Briefly, RNA samples were fragmented, cDNA was synthesized, and cDNA ends were repaired. cDNA fragments were then ligated to adaptor sequences, treated with uracil DNA glycosylase, purified and subjected to quality control using a Bioanalyzer 2100 (Agilent, Santa Clara, CA, USA) and sequenced using a HiSeq 3000 (Illumina, San Diego, CA, USA). Sequencing reads were aligned to the human reference genome (hg38) and normalized into FPKM (fragments per kilo base of transcript per million mapped reads) values.

RNA pulldown

Biotinylated RNA oligonucleotides, incorporating adenosine or m6A, were synthesized (Hippobio, China) and subjected to denaturation at 99 °C for 10 minutes, followed by a gradual cooling to room temperature to facilitate the formation of stem-loop structures prior to utilization. Then biotin-labelled RNA oligonucleotides were fixed onto streptavidin magnetic beads in NT2 buffer. HuH-7 cells expression siNC or siIGF2BP1 were seeded in a 10 cm dish and harvested at 80% confluence to conduct RNA pull-down assay. Cells were lysed with lysis buffer (20 mM Tris pH 7.4, 150 mM NaCl, 0.2% NP40, 1 mM EDTA, 20% glycerol) containing protease inhibitor cocktail and RNase inhibitors and incubated on ice for 30 min. A part of the volume of the supernatant was taken as input control, and the remaining samples were subdivided into two equal portions and incubated with biotin-conjugated RNA oligonucleotides with adenosine or m6A modification overnight at 4 °C. Next, protein-RNA-bead complexes were washed with cold NT2 buffer four times at 4 °C for 5 min, followed by the addition of a suitable volume of SDS loading buffer and denaturation of the protein at 95 °C for 5 min. Analysis of the interaction between the target proteins and m6A-modified RNA oligonucleotides was carried out using immunoblot.

ChIP-seq

Approximately 1×10^7 HuH-7 cells were crosslinked in adherent conditions with 1% formaldehyde for 10 min at room temperature and the reaction was stopped by adding

0.125 M glycine for 5 min at room temperature. Fixed cells were washed two times with cold PBS and harvested by centrifugation at 1,000 g for 3 min at 4 °C. Cell pellets were resuspended in ChIP lysis buffer A (Cell Signaling Technology, 14282S) containing DTT and protease inhibitor cocktail on ice for 10 min, and then resuspended with ChIP lysis buffer B (Cell Signaling Technology, 14282S) containing DTT. Chromatin was digested with Micrococcal Nuclease (Cell Signaling Technology, 10011S) at 37 °C for 20 min and then the reaction was terminated with EDTA on ice for 2 min. Cells were pelleted and resuspended in ChIP buffer (Cell Signaling Technology, 14231S) with protease inhibitor cocktail, and sonicated to yield DNA fragments to length of approximately 300 bp. The crosslinked DNA-protein-containing supernatants were obtained by centrifugation at 10,000 g for 10 min at 4 °C. A 1% volume of supernatant was set as input and the remainder was divided equally and incubated with protein G magnetic beads that had been preincubated with indicated antibodies overnight at 4 °C. The DNA-protein-bead complexes were washed four times with ChIP wash buffer (20 mM Tris-HCl pH 7.4, 500 mM NaCl, 1 mM EDTA, 1% NP40, 0.05% SDS) and de-crosslinked, and then treated with RNase A and proteinase K to digest RNA and protein. DNA fragments were extraction using MinElute Reaction Cleanup Kit (Qiagen, 28606) and DNA concentration was determined by Qubit (Thermo). For ChIP-seq, libraries were prepared with KAPA Hyper-Prep Kits (Roche, KK8502) following the manufacturer's protocol and sequenced on an Illumina Novaseq 6000 sequencer. Sequenced reads were aligned to hg38 using bowtie2.

Co-immunoprecipitation (Co-IP) and immunoprecipitation mass (IP-mass)

For Co-IP and IP-mass, cultured cells were collected and washed with cold PBS, lysed with IP lysis buffer (50 mM Tris-HCl pH 7.5, 150 mM NaCl, 0.2% NP40, 20% glycerol) containing 1 × protease inhibitor cocktail (MedChemExpress, HY-K0010, China) on ice for 30 min. The lysate was subsequently clarified by centrifugation at 12,000g for 10 min at 4 °C. A 5% of the volume of the lysate was taken as an input control, while the remaining lysate was incubated with Protein G magnetic beads (Thermo, 10004D) pre-coated with primary antibody, and rotated at 4 °C overnight. The Protein G magnetic beads bound to the proteins were captured using a magnetic stand, followed by three washes using NT2 buffer (20 mM Tris–HCl pH 7.5, 150 mM NaCl, 1 mM $MgCl_2$, 0.05% NP40) and boiled at 95 °C for 5 min with SDS loading

buffer for Western blotting analysis. The eluted proteins were also subjected to SDS-PAGE for IP-mass analysis (Shanghai Applied Protein Technology, Shanghai, China). Protein identification was retrieved by Mascot version 2.4.01 (Matrix Science, London, UK), in the human RefSeq protein database (National Center for Biotechnology Information).

Cell proliferation and colony formation assays

For the cell proliferation assay, 1,000 to 3,000 cells were seeded per well in a 96-well plate. At the designated time points, the cell culture medium was replaced with a mixture of Cell Counting Kit-8 (CCK-8) reagent (MedChemExpress, HY-K0301) and DMEM in a 1 : 9 ratio, followed by incubation at 37 °C for 1.5 to 2 h. Cell proliferation capacity was evaluated by measuring the absorbance at 450 nm. For the colony formation assays, 2,000 to 5,000 cells were seeded per well in a 6-well plate. After 7 to 10 days, cells were collected, washed 2 times with 1 x PBS, fixed and stained with 1% crystal violet (C0775, Sigma-Aldrich, Missouri, USA) dissolved in methanol for 20 min. Megascopic cell colonies were counted by ImageJ. The impact of target gene silencing or drug administration on cell colony formation capacity was assessed by referencing the control group.

Xenograft in nude mice

For xenograft tumor in nude mice experiment, the stable HuH-7 cells expressing lentiGuide-Puro, lentiGuide-Puro-sgTRIM71 or lentiGuide-Puro-sgCEBPA were first harvested and suspended in DMEM. The experimental animals (male BALB/c-nu/nu, 6-week-old) were randomly divided into groups, with each group containing 6 mice. Each mouse received a subcutaneous injection of 2×10^6 cells in 200 μL of DMEM mixed with Matrigel (Corning, 356234, USA) into the lower back region. For ATRA and A-485 treatment in liver cancer, 2×10^6 HuH-6 liver cancer cells in 200 μL of DMEM mixed with Matrigel were injected into lower back region of nude mice and divided into 4 groups. Control mice were treated with saline, ATRA group were treated with ATRA (20 mg/kg) through intraperitoneal injection, A-485 group were treated with A-485 (10 mg/kg) through intraperitoneal injection, and ATRA + A-485 group were treated with ATRA (20 mg/kg) combined with A-485 (10 mg/kg) through intraperitoneal injection. Tumor length (L) and width (W) were assessed using a caliper and monitored at intervals of 3-4 days. Calculation of the tumor volume (V)

was performed using the formula: $V = (L \times W^2)/2$. The mice were euthanized, and the xenograft tumors were harvested and weighed. All animal experiments were performed in accordance with guidelines approved by the Animal Care and Use Committee of the Fudan University Shanghai Cancer Center (FUSCC).

Hydrodynamic tail vein injection

Male C57BL/6J mice (6-8 weeks old) were purchased from the Animal Center of Fudan University Shanghai Cancer Center. Mice were fixed into mouse tail injection fixator imaging apparatus (GEGD-Q9G, Mukexi, China), and tail were swabbed to fill blood vessels using 75% ethanol. Plasmids suspended in normal saline solution in a volume equal to 10% of the body weight was injected in 5 to 7 s via the tail vein. The amount of injected plasmids was 20 μg of transposon plasmids together with 3 μg of SB100 transposase plasmids (10 μg of pT3-TRIM71 plasmids + 10 μg of pT3-YAP5SA plasmids + 3 μg of SB100; 10 μg of pT3-TRIM71 plasmids + 3 μg of SB100). Animals were monitored for up to 8 months and were euthanized at specific time points after injection or when symptoms of tumorigenesis such as abdominal enlargement were evident. Livers were pictured and weighted, and tissues were then fixed or frozen for further processing. All animal experiments were performed in accordance with guidelines approved by the Animal Care and Use Committee of the Fudan University Shanghai Cancer Center (FUSCC).

Immunohistochemistry (IHC)

Mouse liver tissues were collected, washed two times with 1 x PBS and immersed in 4% paraformaldehyde for 24 h at room temperature to fix tissue samples. The fixed samples were embedded in paraffin, followed by sectioning at a thickness of 4 μm onto slides and subsequent baking. Tissue sections were deparaffinized with xylene for three times, rehydrated in gradient of ethanol, and then washed with distilled water to remove ethanol. The sections were treated with 10 mM sodium citrate buffer (pH 6.0) and subjected to microwave heating for 20 min for antigen retrieval. Endogenous peroxidase was inactivate using 3% hydrogen peroxide solution for 25 min at room temperature. Then, the slides were blocked with 3% BSA solution at room temperature for 30 min to remove non-specific antigens and incubated with indicated primary antibody in a humidified chamber at 4 °C overnight. Next, the sections were washed and incubated with HRP labeled secondary antibody corresponding to the

species of the primary antibody in a humidified chamber at room temperature for 50 min. IHC staining was developed using DAB as chromogen and hematoxylin as the counterstain to detect the objective signals.

Luciferase assays

HuH-7 and Hep3B cells were seeded in 24-well plates and co-transfected with 500 ng pGL-luc-CEBPA vectors (firefly luciferase, Fluc), 50 ng pRL-CMV-Renilla (Renilla luciferase, Rluc, Promega) reporter vectors and siNC, or siTRIM71 or siIGF2BP1 using RNAimax (Thermo, 13778150). After 48 h of transfection, cells were collected, washed and lysed, and then the Fluc and Rluc luciferase activities were detected by the Dual Luciferase Reporter Gene Assay Kit (YEASEN, 11402ES60). To determine the relative luciferase activity, the Fluc signal values was divided by the Rluc signal values and then normalized to the respective negative control for each assay.

Public data analysis using TCGA, DepMap and GepLiver database

DepMap database (https://depmap.org/portal/) was utilized to analyze TRIM71 mRNA expression, expressional correlation, association between target genes expression and cell proliferation, and proliferative correlation for all selected genes in liver cancer cells. The correlation between mRNA expression of target genes and survival time was determined using the TCGA liver hepatocellular carcinoma databases (https://portal.gdc.cancer.gov/). The expression pattern of all selected genes in fetal liver, normal liver, and liver diseases was assessed through analysis using the GepLiver database (http://gepliver.org/).

Human single cell RNA-seq processing

The integrated liver single cell RNA-seq Seurat object was download from the GepLiver data resource established by our research team previously. Briefly, GepLiver database curated raw reads of single cell RNA-seq datasets from public database and reanalyzed by cellranger using GRCh38 as human genome reference. We removed cells expressed fewer than 300 genes or with a higher mitochondrial gene percent as well as genes expressed in less than 3 cells for quality control. Doublets were also predicted with DoubletFinder R package (v2.0.3) and excluded from analysis. Subsequently, Seurat objects were merged into one followed by two-round Harmony integration and cell type annotation. Epithelial cells were annotated

with canonical cell markers, ALB, TTR and HNF4A for hepatocytes as well as KRT19, EPCAM, TM4SF4 and FXYD2 for cholangiocytes. For epithelial cells from fetal liver, hepatoblasts were additionally identified based on the expression of SPINK1, HNF4A and AFP. Malignancy status according to inferred copy number variation as well as tissue origin were both added to the epithelial annotation, characterizing 7 major cell types including Fetal_Hepatoblast, Fetal_Hepatocyte, Fetal_Cholangiocyte, Hepa_Malignant, Hepa_Normal, Chol_Malignant and Chol_Normal. Ultimately, single-cell data derived from 63562 epithelial cells of 263 samples involving fetal liver, normal liver, HCC, ICC, HB and tumor adjacent tissues were extracted for downstream analysis.

Public RNA-seq datasets processing for human bulk tissue and cancer cell lines

Human bulk transcriptomic profiles from 1,708 liver samples across 33 datasets, encompassing fetal and normal liver phenotype, liver malignant tumor as well as tumor adjacent samples, were retrieved from GepLiver database. The reprocessed expression matrices were directly used for downstream analysis without any batch correction procedures. RNA-seq raw reads of cancer cell lines were retrieved from Cancer Cell Line Encyclopedia and reprocessed through the standardized pipeline of ASJA program and annotated with GENCODE v29. Counts were normalized to TPM values and log2-transformed for use.

Pair-wise transcriptomic correlation analysis

For each liver sample with epithelial cells over 50, single cell transcriptomic profiles were further merged by epithelial cell type into pseudobulks. Top 5,000 variable genes chosen by the coefficient of variation (CV) were used to compute Pearson correlation coefficient for all pair-wise combinations of pseudobulks. Subsequently, correlation heatmap was plotted performing consensus clustering on coefficient matrices with clustering pseudobulks demonstrating similar expression pattern.

Oncofetal signature generation and scoring

Differential analysis was performed between cells derived from oncofetal module and other epithelial cells using Seurat “FindMarkers” function with top 200 features with highest log2FC and significant adjust p-value composed oncofetal signature. Oncofetal activity was evaluated with UCell R package in single cells RNA-seq

whereas using ssgsea algorithm for expression profile of bulk tissues and cell lines. Both methods scored the oncofetal signature based on relative rankings of involved features at the single sample level unaffected by sample composition.

To confirm that liver cancer single cells expressing the oncofetal signature are definitely cancer cells, inferCNV to deduce CNVs from scRNA-seq datasets curated by our GepLiver project. InferCNV was utilized to facilitate the comparison of gene expression levels across specific genomic regions against reference cells, thereby identifying somatic chromosomal copy number aberrations. Epithelial cells from tumor samples exhibiting distinct copy number gains or losses were identified as tumor cells. To mitigate batch effects, each dataset was analyzed independently. To avoid the potential algorithmic errors, semi-supervised approach was chosed to designate the malignancy status from inferCNV output. Briefly, the mean of inferCNV observations as the CNV score for each cell was calculated and then compared the CNV scores of tumor cell clusters with those of normal epithelial and endothelial cells to ascertain the malignancy status.

Cell developmental potential

Differentiation states were evaluated for liver epithelial cells of each HCC sample using CytoTRACE R package (version 0.3.3). CytoTRACE is a computational algorithm which estimates gene counts per cell as transcriptomic diversity for measuring single cell relative developmental potential. The CytoTRACE scores range from 0 (relatively more differentiated) to 1(relatively less differentiated). Furthermore, we inferred cell developmental trajectory using Monocle R package (version 2.22.0) for sample SC14_HCC_058 which harbored high abundance of fetal-like cells as well as strong correlation between oncofetal score and cytotrace score. Genes with mean expression > 0.1 and dispersion_empirical >=1 * dispersion_fit were regarded as variable features for cell ordering. Differentiation trajectories were subsequently built with default parameters. The spectrum of Pseudotime started from 0 which denoted differentiation origin.

Pathway enrichment analysis

Pathway enrichment analysis was performed with clusterProfiler R package (v4.4.4). HALLMARK, KEGG and GO-BP gene sets were obtained from msigdbr package

(v7.5.1). Over-represented pathways were enriched for oncofetal signature using enricher function. To explore the transcriptome-wide expression changes in oncofetal phenotype, we also exploited GSEA function for gene set enrichment analysis comparing fetal-like cells and not group among HCC epithelial cells.

Filtering for core oncofetal genes with tumorigenic function

Chronos gene dependency score was obtained from Depmap project (https://depmap.org/portal/download/all/, Public 23Q2) to suggest the tumorigenic function of oncofetal genes in CCLE cancer cell lines. The dependency score reflects the scale of effect CRISPR knockout of one gene have on the viability or proliferation of the specific cell line. Genes with scores of 0 represents non-essential for the given cell line. Two kinds of associations were assessed for CCLE liver cancer cell lines to filter for core oncofetal genes with tumorigenic function. Pearson correlation was evaluated for gene expression with both oncofetal scores and gene dependency scores. Genes with the expression positively associated with oncofetal scores indicated favorable contribution to fetal-like phenotype whereas those with the expression negatively correlated to dependency scores suggested a dose-dependent effect on cell viability.

Correlation between oncofetal scores and clinicopathological variables

We assessed the association between oncofetal scores and clinical characteristics in the GepLiver bulk meta-cohort by fitting a linear model for each parameter independently with different source dataset as the covariate to regress out the confounding factor.

Statistical analysis

Statistical analyses were conducted utilizing R software or GraphPad Prism software (Version 9.4.0). All statistical data were represented as the mean ± SEM from at least three independent experiments unless otherwise stated. Statistically significant differences of date were determined using unpaired Student’s t test and presented as box plots and line graphs. The correlation was analyzed by calculating Pearson correlation coefficients (r), and the statistical significance was evaluated using a two-tailed t-test with $r = 0$. The survival distributions among experimental groups were compared using the log-rank test and showed as the Kaplan-Meier survival curves. A significance level of $P < 0.05$ was used to define statistical significance (ns, not significant; *, $p < 0.05$; **, $p < 0.01$; ***, $p < 0.001$).

Supplementary Figures

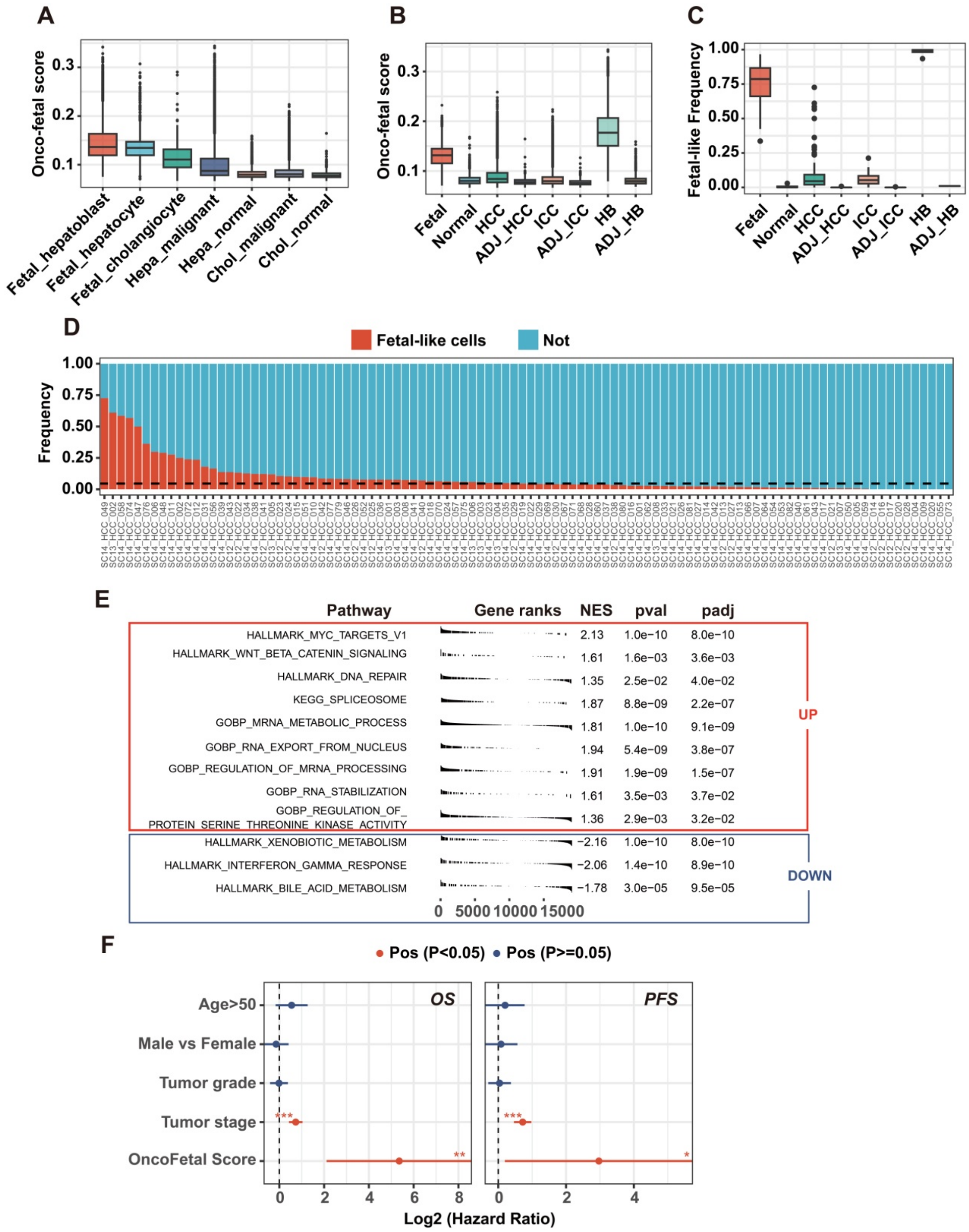

Figure S1. Evaluating oncofetal signature in single cell and bulk liver datasets. (A) Box plot showing oncofetal scores of epithelial cells grouped by cell type. (B) Box plot displaying oncofetal scores of epithelial cells grouped by liver cancer phenotype. (C) Box plot displaying frequency of fetal-like epithelial cells grouped by liver phenotype. Fetal-like cells were defined as epithelial cells with oncofetal score above 95 percent of all normal epithelial cells. For box plots in (A)-(C), center line

represents the median whereas limits show upper and lower quartiles. Data extend beyond the 1.5 times of the interquartile range from box limits were shown as outlier points. (D) Stacked bar plot demonstrating the abundance of fetal-like cells in each HCC sample with over 50 epithelial cells. Black dashed line represents the median frequency of fetal-like cells in all HCC samples. (E) Gene set enrichment analysis performed between fetal-like cells and not group among HCC samples. NES are normalized scores indicating the magnitude of enrichment. NES above 0 denotes upregulated pathways whereas score below 0 denotes downregulated in fetal-like cells. Adjusted p values were computed using Benjamini-Hochberg method. (F) Multivariate Cox proportional hazards regression analysis demonstrating oncofetal signature as negative prognostic factor independent of age, sex, tumor grade and tumor stage.

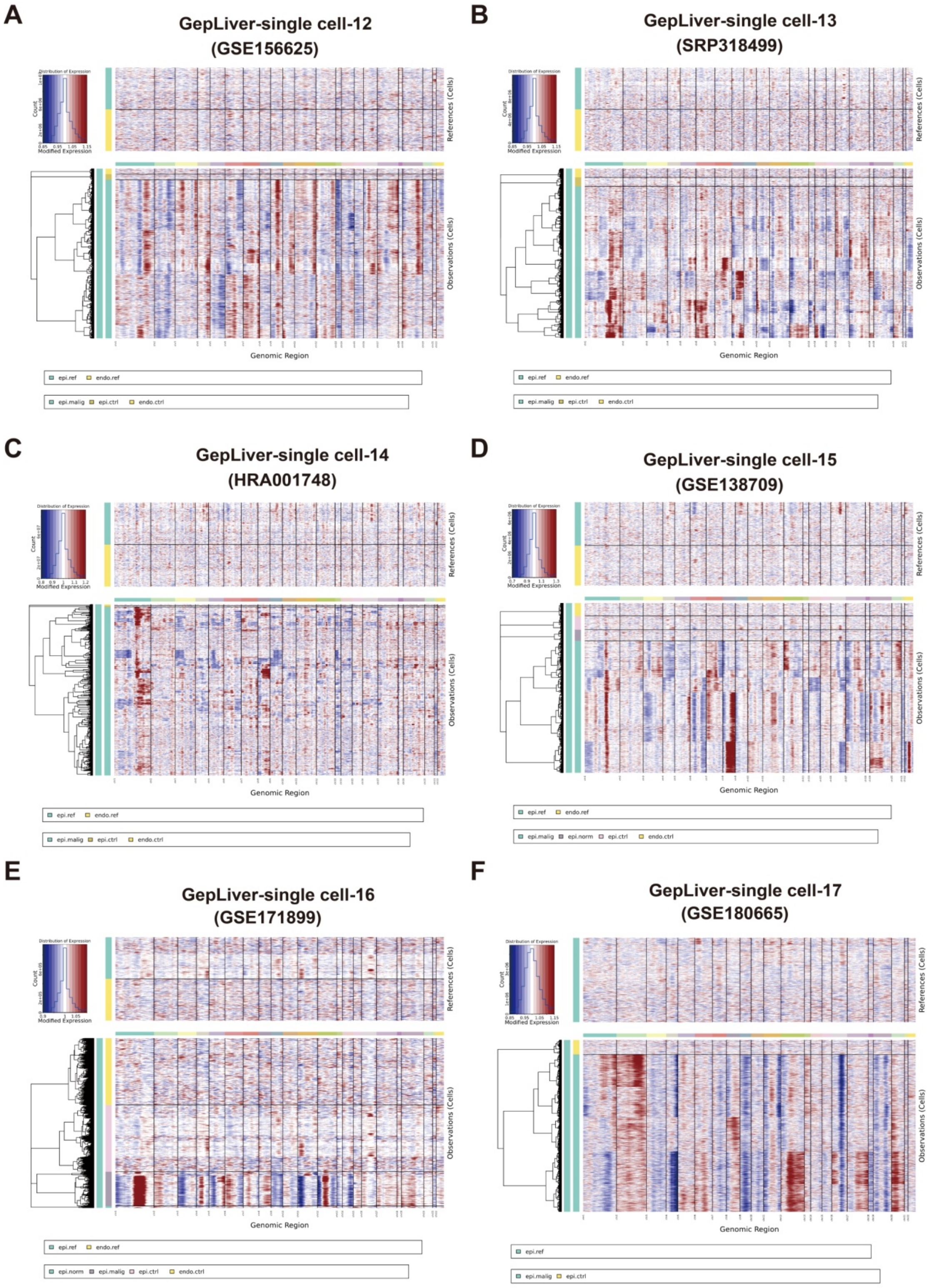

Figure S2. Heatmaps of relative expression intensities across genomic regions demonstrated copy number variations of cancer cells. Heatmaps (A-F) were plotted separately for six single cell datasets included containing HCC, HB or ICC tumor tissues. These heatmaps were generated by inferCNV based on the relative gene expression matrices compared to the reference normal cells. The upper panel

exhibited the baseline gene expression of reference cells (epi.ref and endo.ref) of the corresponding dataset whereas the lower panel delineated relative expression intensities for malignant epithelial cells assigned (epi.malig) as well as normal cells as controls (epi.ctrl and endo.ctrl). Malignant epithelial cells demonstrated significant more or less expression in chromosomal regions in contrast to normal reference and control cells, which denotes copy number amplifications or deletions.

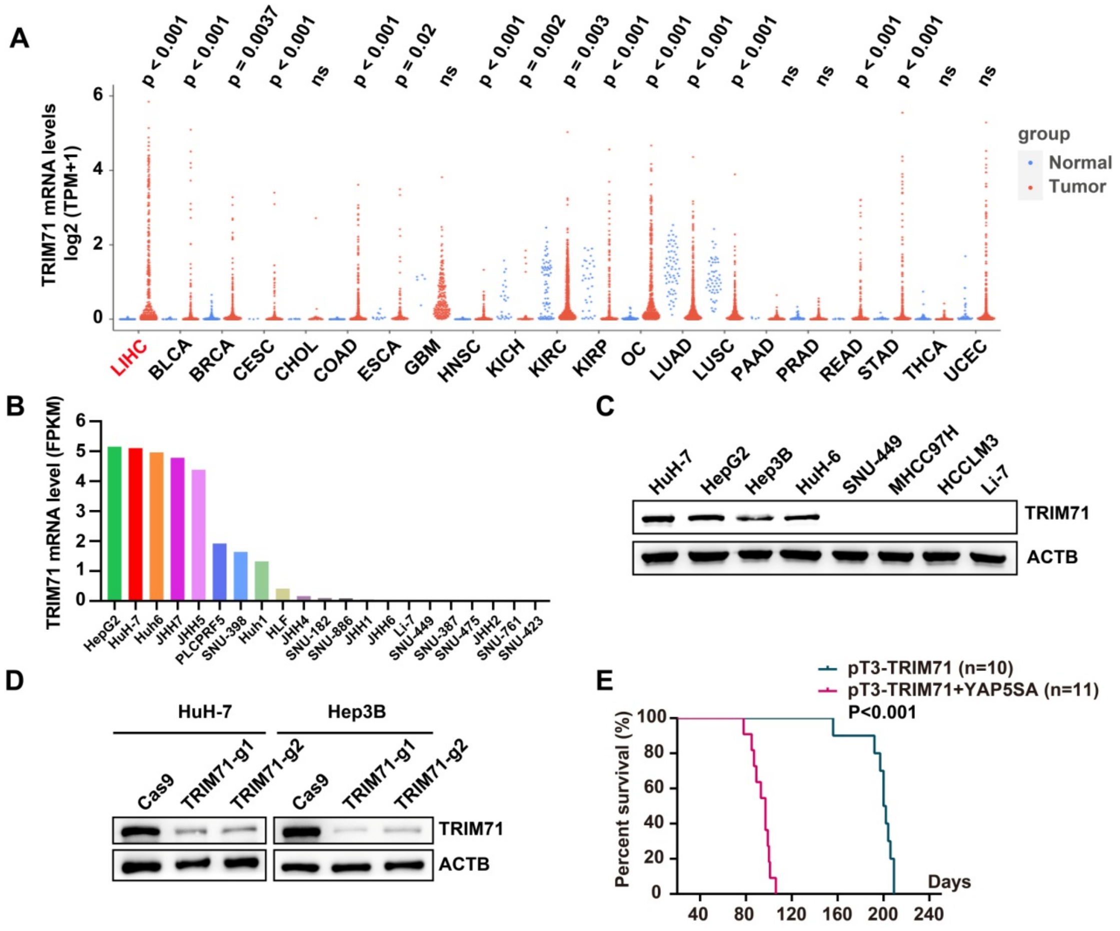

Figure S3. TRIM71 specifically facilitates liver cancer cell proliferation in vitro. (A) The mRNA levels of TRIM71 in pan-cancer tissues and normal tissues from TCGA dataset. (B) The mRNA expression of TRIM71 in multiple liver cancer cell lines via analysis from Depmap database. (C) Immunoblot analysis of TRIM71 protein levels in various liver cancer cell lines. (D) Determination of TRIM71 knockdown efficiency via immunoblot in HuH-7 and Hep3B cells infected with specific sgRNAs targeting TRIM71 and Cas9 lentiviruses. (E) K-M survival curve analysis in mice with overexpression of TRIM71 alone or in combination with overexpression of YAP5SA using the hydrodynamic tail-vein injection.

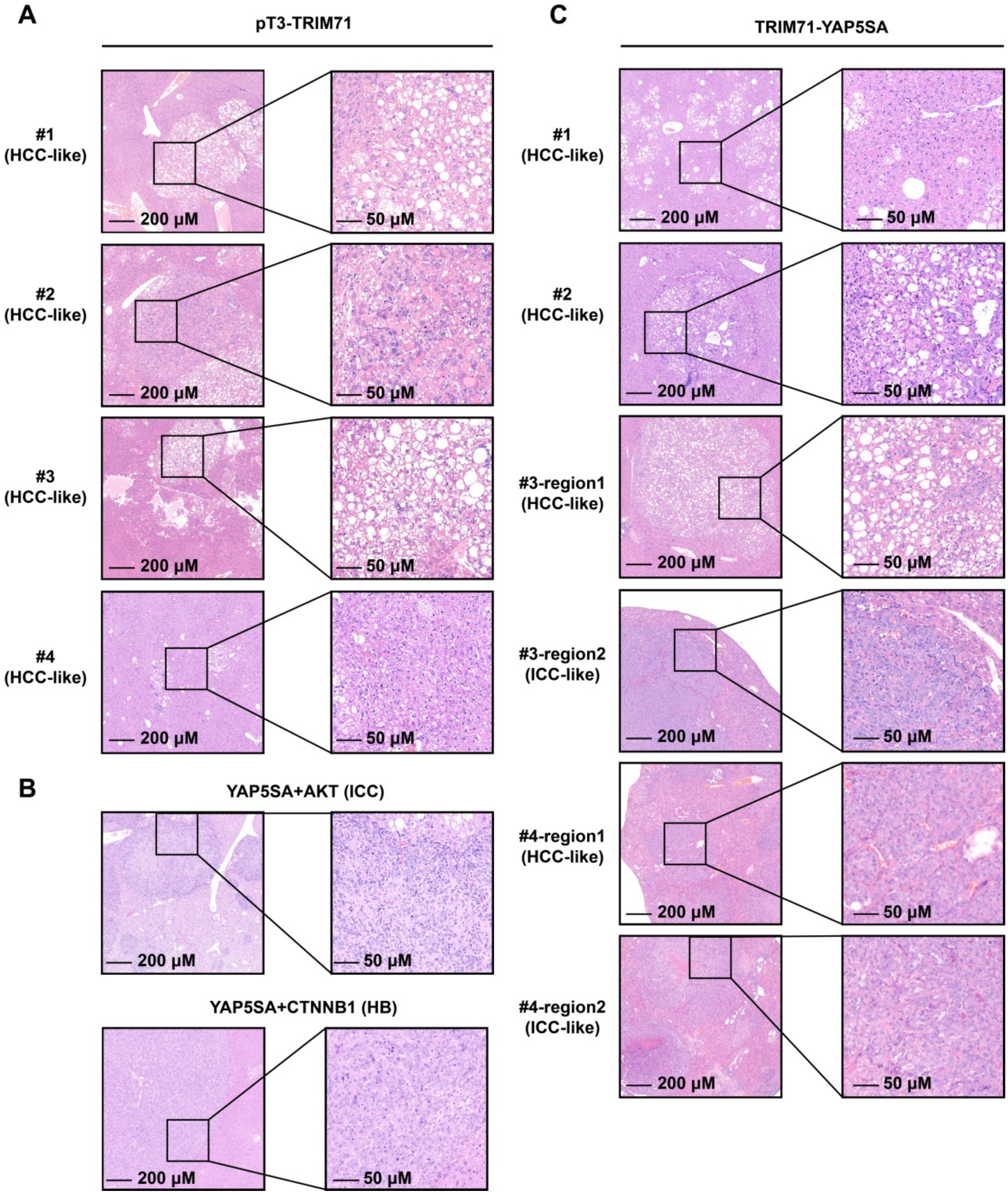

Figure S4. The HE staining of TRIM71 induced liver cancer tumors in mice. (A-B) HE staining revealed that the liver tumor cells induced solely by TRIM71 exhibit enlarged nuclei, increased nuclear-cytoplasmic ratio, deepened nuclear staining, prominent nucleoli, and marked cellular pleomorphism. Additionally, the tumor cells contain uniformly sized round lipid droplets within the cytoplasm, and there are abundant sinusoid-like spaces between the tumor cells, and the surrounding hepatocytes show fatty degeneration (A). These morphological features are typical of HCC and are distinctly different from the morphologies induced by YAP5SA + AKT in ICC and YAP5SA + CTNNB1 in HB (B). (C) liver cancer tissues induced by

TRIM71 + YAP5SA exhibited characteristics of both HCC and mixed HCC-ICC features.

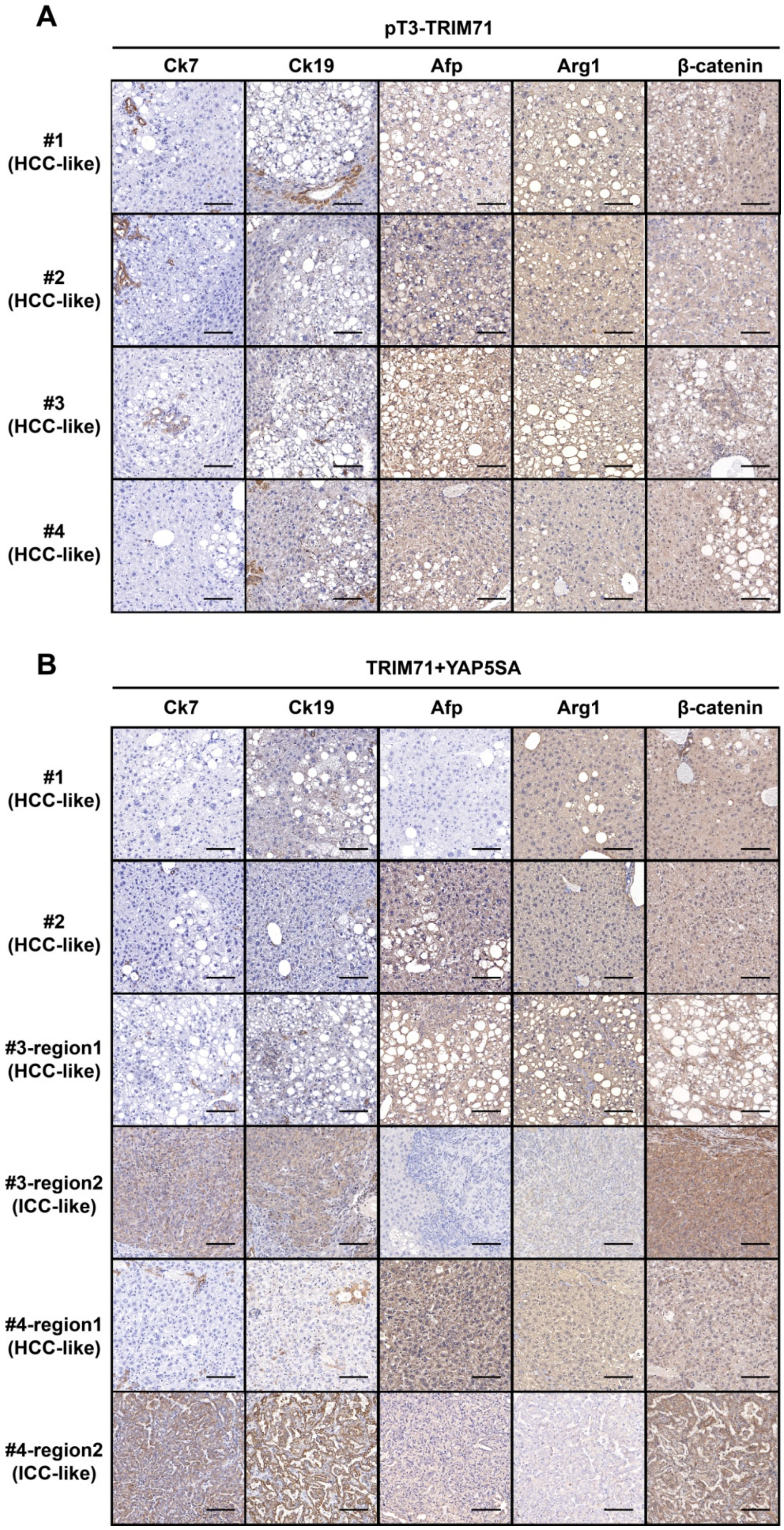

Figure S5. The IHC staining of TRIM71 induced liver cancer tumors in mice. (A) Ck7, Ck19, Afp, Arg1, β-catenin were selected to identify the subtype of liver cancer.

Liver tumors of pT3-TRIM71 showed Afp, Arg1 and β-catenin expression but with no Ck7 or Ck19 expression. (B) Liver tumor tissues 1 and 2 with TRIM71 + YAP5SA expression exhibit Arg1 and β-catenin, but do not express Ck7 and Ck19. Conversely, liver tumor tissues 3 and 4 with TRIM71 + YAP5SA show characteristics of mixed HCC-ICC tumors, with some tumor tissues exhibiting features of HCC while other parts display ICC traits. Scare bar = 50 μM.

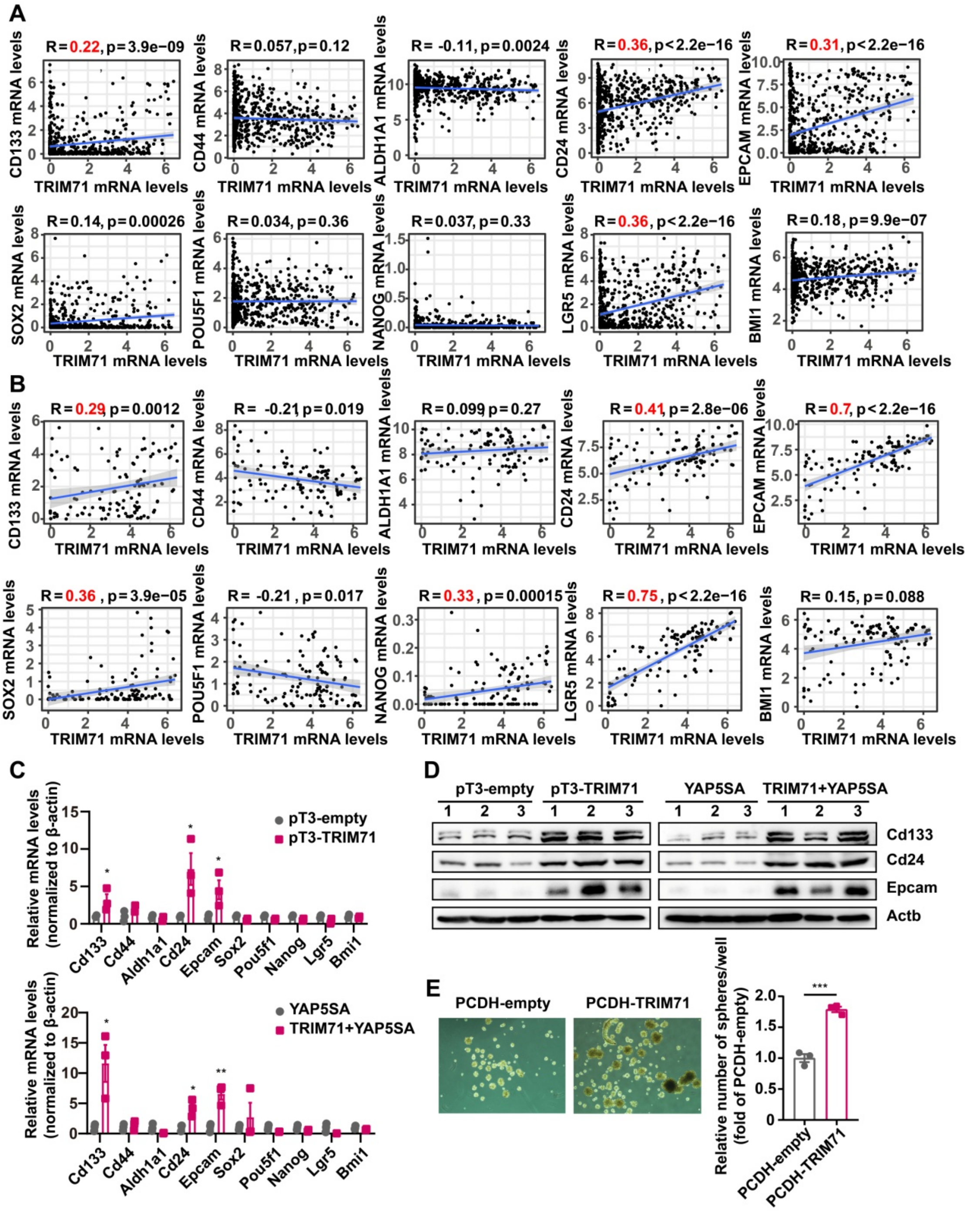

Figure S6. TRIM71 participates stemness in liver cancer. (A-B) The mRNA expressional correlation of TRIM71 with CD133, CD44, ALDH1A1, CD24, EPCAM, SOX2, POU5F1, NANOG, LGR5, BMI1 in HCC (A) and HB (B) tissues. (C) The relative mRNA levels of Cd133, Cd44, Aldh1a1, Cd24, Epcam, Sox2, Pou5f1, Nanog, Lgr5, Bmi1 in pT3-empty, pT3-TRIM71, YAP5SA, TRIM71 + YAP5SA liver or tumor tissues. (D) Immunoblot analysis of Cd133, Cd24, Epcam protein levels in pT3-empty, pT3-TRIM71, YAP5SA, TRIM71 + YAP5SA liver or tumor tissues. (E) The in vitro spheres formation ability of liver cancer Li-7 cells infected with PCDH-

empty or PCDH-TRIM71 lentiviruses. β-Actin served as loading control. Values represent the mean ± SEM. *P<0.05, **P<0.01, ***P < 0.001.

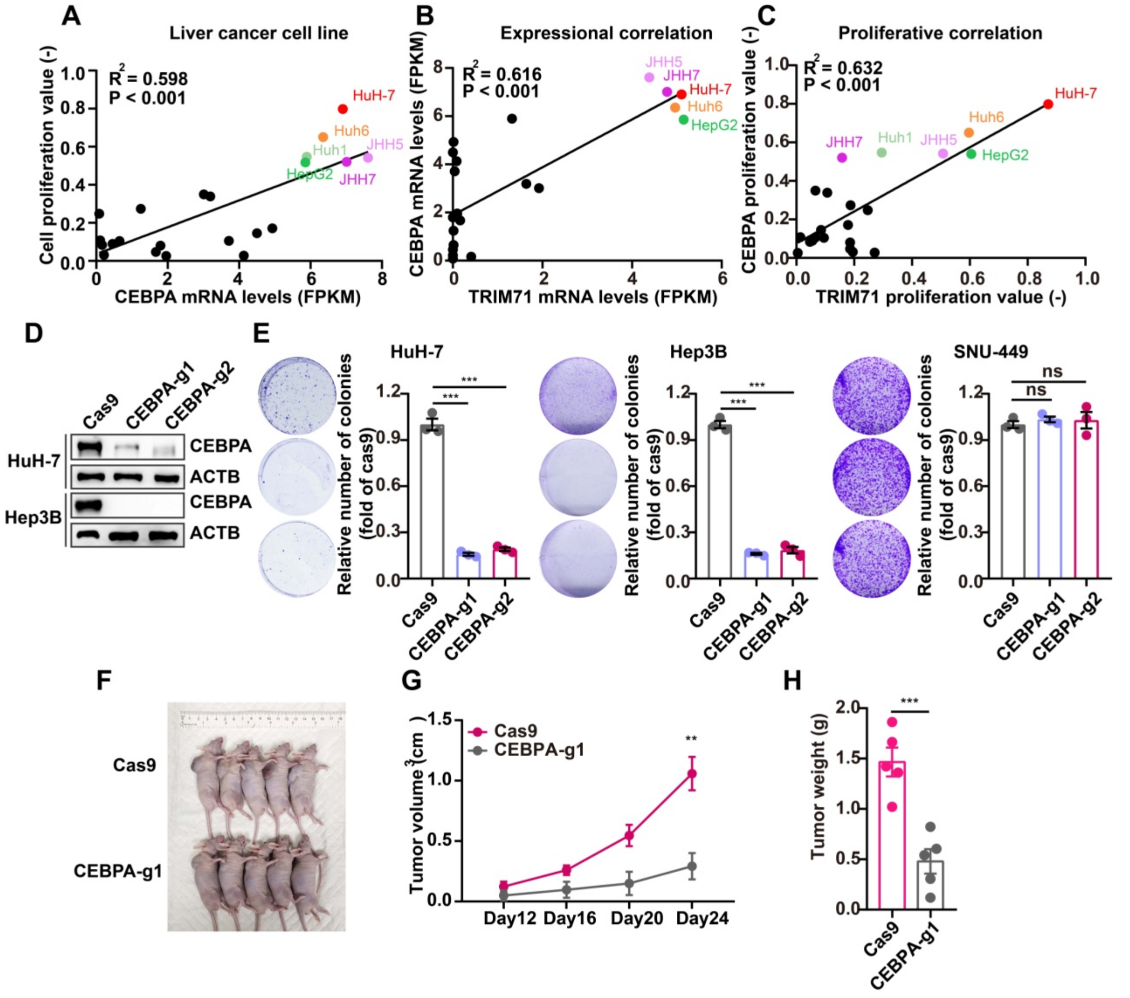

Figure S7. CEBPA is critical for liver cancer cell proliferation. (A) Correlation analysis between CEBPA mRNA levels and cell proliferation of liver cancer cell lines. (B) Correlation analysis of mRNA levels between CEBPA and TRIM71 in liver cancer cell lines. (C) Correlation analysis between CEBPA dependent cell proliferation and TRIM71 dependent cell proliferation in liver cancer cell lines. (D) Assessment knockdown efficiency of CEBPA in HuH-7 and Hep3B cells by immunoblot analysis. (E) Effects of CEBPA knockdown on cell proliferation and colony formation in HuH-7, Hep3B and SNU-449 cells infected with ctrl or sgCEBPA lentiviruses. (F) Images of the xenograft mouse models implanted with HuH-7 cells with or without CEBPA knockdown. (G-H) Evaluation of the tumor volume (G) and tumor weight (H) of HuH-7 xenograft tumors with or without CEBPA knockdown. Values represent the mean ± SEM. **P < 0.01, ***P < 0.001. ns: no significance.

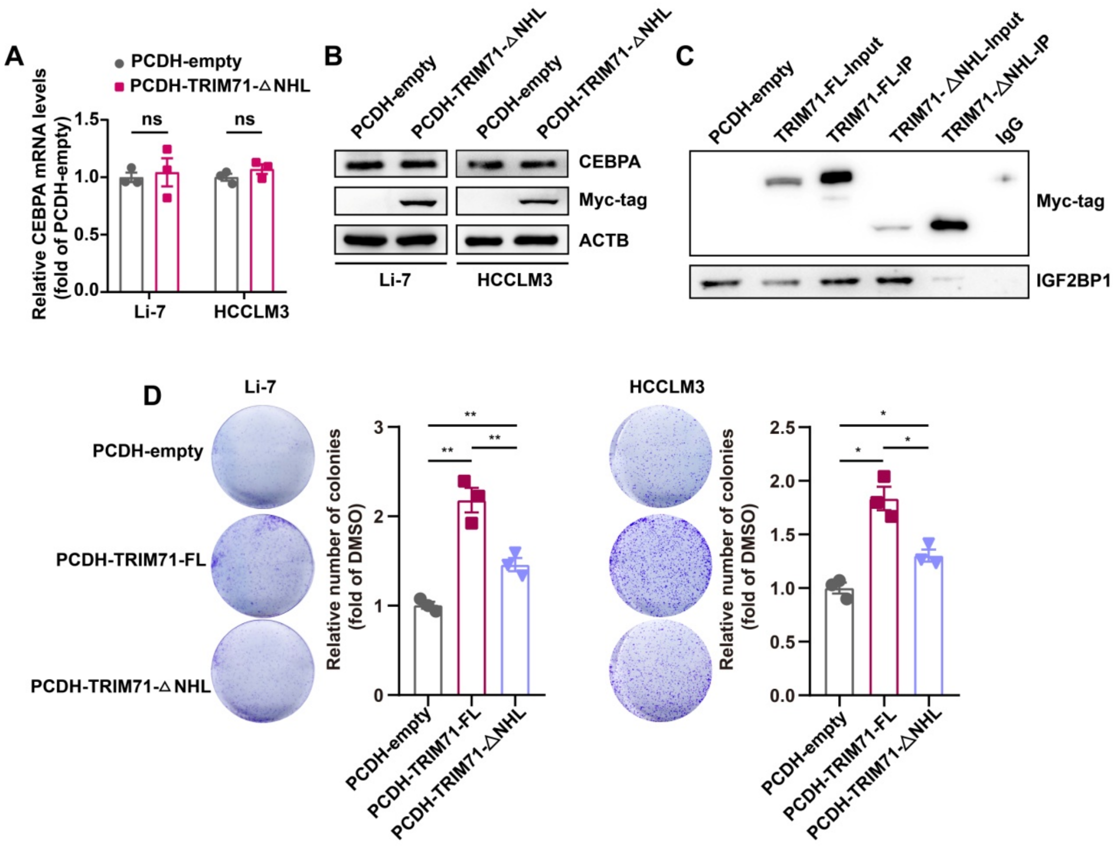

Figure S8. The NHL domain of TRIM71 in regulation of mRNA expression of CEBPA. (A-B) The mRNA levels (A) and protein levels (B) of CEBPA in Li-7 and HCCLM3 liver cancer cells infected with PCDH-empty or PCDH-TRIM71-NHL deletion lentiviruses. (C) Immunoblot analysis of myc-tag and IGF2BP1 protein levels in HuH-7 liver cancer cells transfected with PCDH-empty, PCDH-TRIM71-FL and PCDH-TRIM71-NHL deletion plasmids and performed with protein immunoprecipitation experiments. (D) The colony formation assay of Li-7 and HCCLM3 liver cancer cells infected with PCDH-empty, PCDH-TRIM71-FL, or PCDH- TRIM71-NHL deletion lentiviruses. Values represent the mean ± SEM. *P <0.05, **P < 0.01.

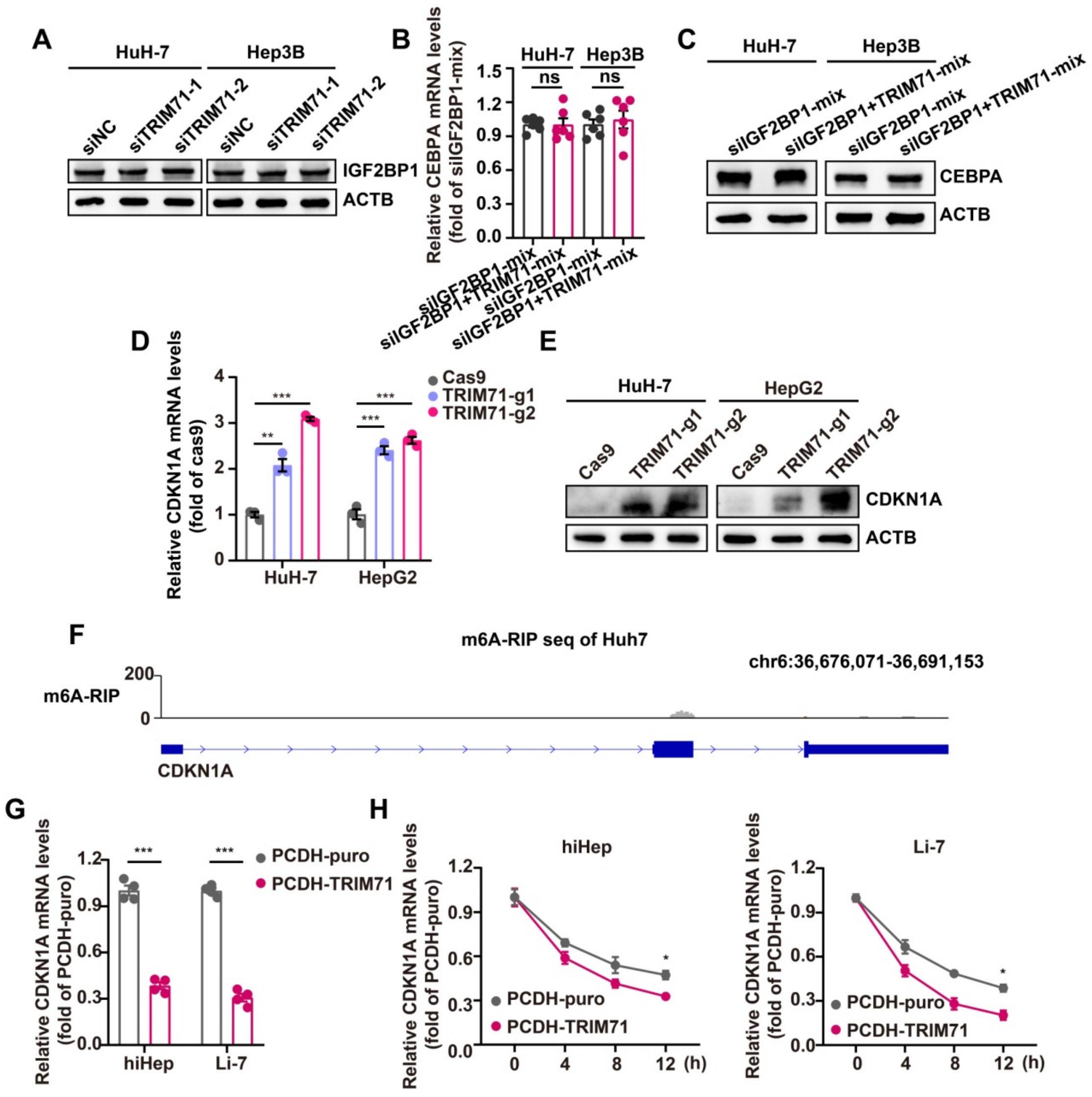

Figure S9. TRIM71 regulates mRNA stability of CEBPA and CDKN1A. (A) Assessment IGF2BP1 protein levels in HuH-7 and Hep3B cells with or without TRIM71 knockdown by immunoblot analysis. β-actin (ACTB) is used as a loading control. (B) RT-qPCR analysis of CEBPA mRNA expression in IGF2BP1 knockdown HuH-7 and Hep3B cells that were transfected with the control or TRIM71 siRNA. (C) Immunoblot analysis of CEBPA protein levels in IG2BP1 knockdown HuH-7 and Hep3B cells that were transfected with the control or TRIM71 siRNA. (D) The mRNA levels of CDKN1A in HuH-7 and HepG2 liver cancer cells infected with Cas9 and TRIM71 gRNAs. (E) The protein levels of CDKN1A in HuH-7 and HepG2 liver cancer cells infected with Cas9 and TRIM71 gRNAs. (F) m6A-RIP seq reveals no m6A modification signal in CDKN1A mRNA of HuH-7 liver cancer cells. (G-H) The mRNA levels (G) and RNA stability (H) of CDKN1A in hiHep normal liver cells

and Li-7 liver cancer cells infected with control or TRIM71 overexpression lentiviruses. The Values represent the mean ± SEM. **P < 0.01, ***P < 0.001.

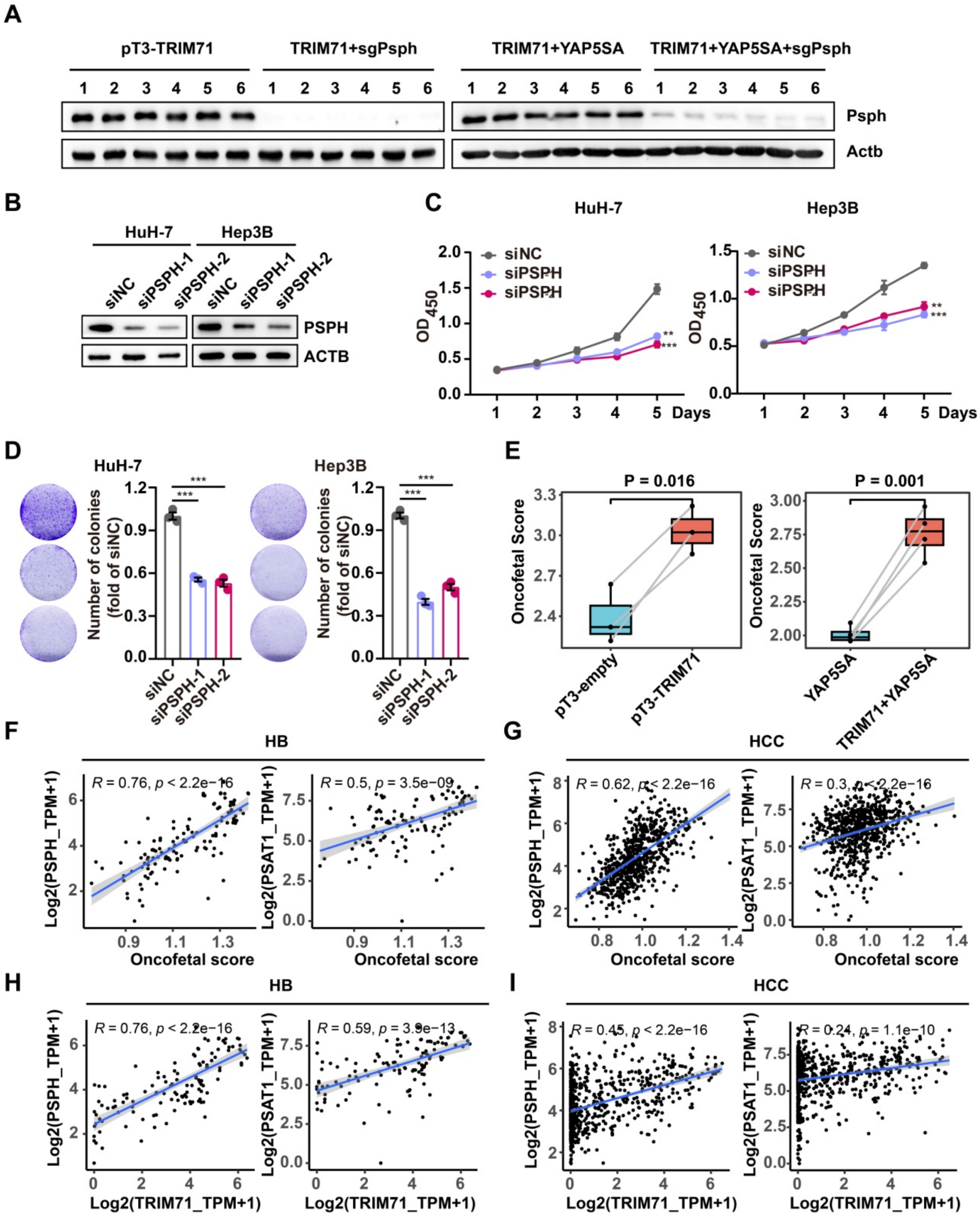

Figure S10. PSPH promotes liver cancer cell growth and exhibits higher oncofetal features. (A) Immunoblot confirming the knockdown efficiency of Psph protein levels in pT3-TRIM71, TRIM71 + sgPsph, TRIM71 + YAP5SA or TRIM71 + YAP5SA + sgPsph mice tumors using western blot assay. (B) Immunoblot confirming the knockdown efficiency of siRNA against PSPH in HuH-7 and Hep3B cells. (C-D) CCK-8 assay (C) and colony formation assay (D) in HuH-7 and Hep3B liver cancer cells transfected with siNC or PSPH siRNAs. (E) The difference of oncofetal score in control and TRIM71 induced liver cancer mice models. (F-G) The

correlation of between PSPH or PSAT1 mRNA levels with oncofetal score in HB (F) and HCC (G) tissues from Gepliver database. (H-I) The expressional correlation of between PSPH or PSAT1 mRNA levels with TRIM71 mRNA levels in HB (H) and HCC (I) tissues from Gepliver database. The Values represent the mean ± SEM. **P < 0.01, ***P < 0.001.

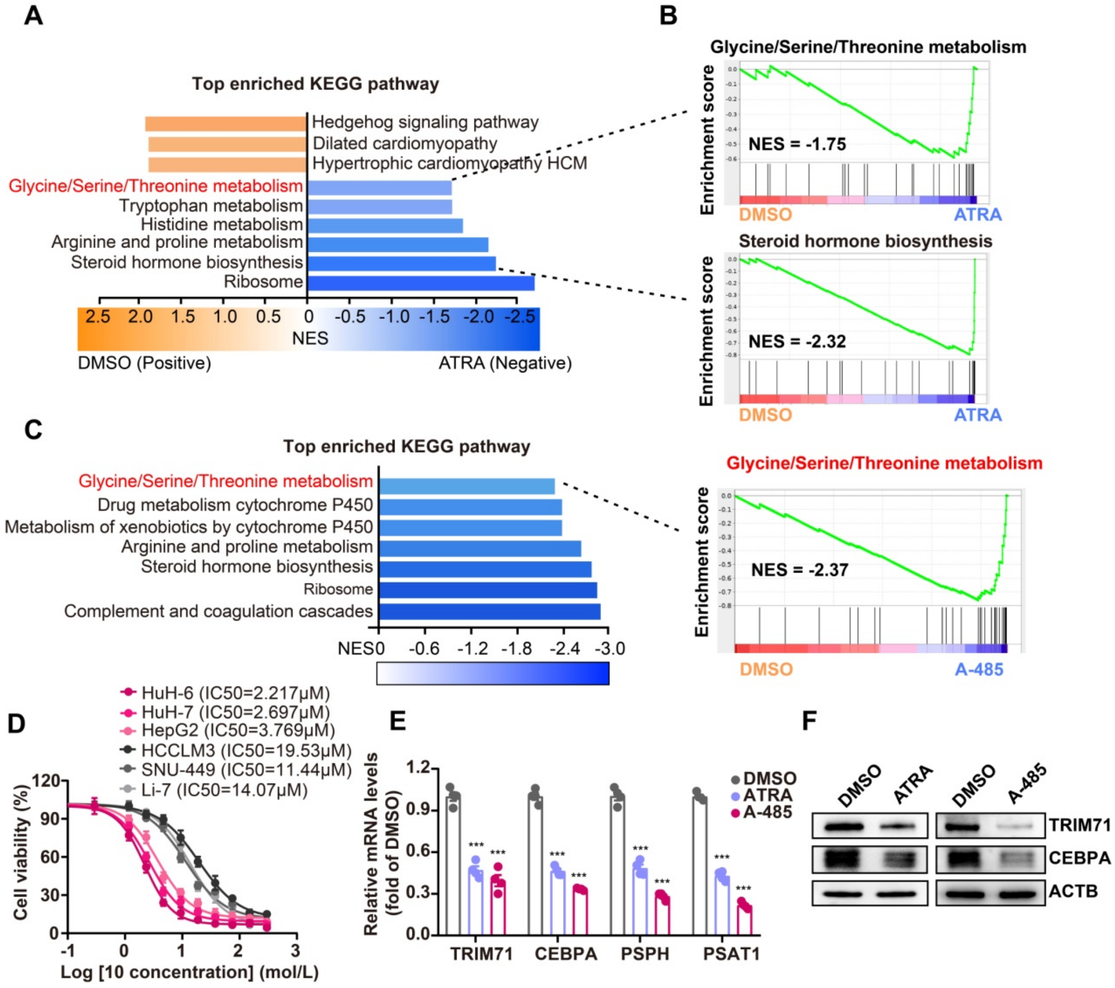

Figure S11. ATRA combined with A-485 exert anti-tumor ability in high TRIM71 liver cancer patients. (A) Top enriched KEGG pathway analysis of differentially expressed genes in HuH-7 cells treated with DMSO or ATRA. (B) GSEA analysis displaying down-regulated pathways including Glycine/Serine/Threonine metabolism in HuH-7 cells treated with DMSO or ATRA. (C) Top enriched KEGG pathway analysis including Glycine/Serine/Threonine metabolism differentially expressed genes in HuH-6 cells treated with DMSO or A-485. (D) The IC50 value of A-485 in liver cancer cell lines. (E) Relative mRNA levels of TRIM71, CEBPA, PSPH, PSAT1 in HuH-7 cells treated with DMSO, ATRA or A-485. (F) Protein levels of TRIM71 and CEBPA in HuH-7 cells treated with DMSO, ATRA or A-485. Values represent the mean ± SEM. ***P < 0.001.

Table S1. Oncofetal genes in liver cancer

Gene	RBPs	TFs
DLK1		
AFP	Yes	
PEG3		Yes
BEX1		
COL2A1		
GPC3		
HBB	Yes	
DUSP9	Yes	
IGF2BP1	Yes	
RBP2		
PEG10	Yes	
RHBG		
HMGA2		Yes
TRIM71	Yes	
PNMA3		
IGF2		
SULT1E1		
NKD1		
TOP2A	Yes	
ARID3A		Yes
FKBP10	Yes	
TSPAN18		
IGSF1		
CACNB4		
CDCA7		
GREB1		
SOAT2		
NOTUM		
ASPM	Yes	
ROBO1	Yes	
LIN28B	Yes	Yes
MYH7B		
SMLR1		
CENPF	Yes	
GNG4		
AURKB		
SLC29A4		
PLPPR1		
LGR5		
SERPINI1		
ZNF711		Yes
SLC2A1	Yes	
IGF2BP2	Yes	
SALL4		Yes
FIGN		
SOBP		
GPAM		
HIF3A		Yes
TBX3		Yes

PLAG1		Yes
KLB	Yes	
MDK		
MBNL3	Yes	
MYH10	Yes	
PTK7	Yes	
MKI67	Yes	
TSPAN5		
MSI1	Yes	
RRM2	Yes	
CDK1	Yes	
MAP7D2		
C21orf58		
FRAS1		
LEF1		Yes
HELLS	Yes	
IQCA1		
QPRT	Yes	
FBLN1		
VSNL1		
SLC16A10		
DKK1		
FAM3B		
BCL11A		Yes
LINGO1		
ELP1		
SP5		Yes
TNFRSF19		
CENPE	Yes	
ORC6	Yes	
BMP4		
CCNF		
NT5DC2	Yes	
CST1		
WRN		
UBE2C	Yes	
BCAM		
AXIN2		
CCDC88A	Yes	Yes
SMARCA1	Yes	Yes
SPTLC3		
KIZ	Yes	
RNF43		
HJURP		
ASPSCR1		
DLGAP5	Yes	
BRCA2		
ANK1		
ESCO2		
TPX2	Yes	
MAP4K4	Yes	

GTSE1	Yes	
PBK		
PLK1	Yes	
ADAMTS6		
CCNB1	Yes	
HMGCR		
MEX3A	Yes	
ITPR2	Yes	
GLUD1	Yes	
CHTF18	Yes	
PARPBP		
CCNA2		
KNTC1	Yes	
RFC3	Yes	
PHYHIPL		
HIC2		Yes
ERP27		
SERPINE2		
NRXN3		
DGKD		
KITLG		
DAB1		
PLCG1	Yes	
PGC		
H2AFY2		
PIF1		
MFAP2		
GDF11		
LRP1	Yes	
SGO2		
TBCK		
BUB1B	Yes	
SOX12		Yes
CDC25C		
SEMA4G		
SLC25A36		
SPC25		
TMEM245	Yes	
MDFI		
ZNF334		Yes
TRIM24	Yes	
APCDD1		
UTRN	Yes	
KIF18B	Yes	
MYBL2		Yes
CLCN5		
DEPDC1		
ETV4		Yes
CDCA3		
PCLAF		
PFKM	Yes	

CKAP2	Yes	
PDE4D		
ARHGAP11A		
ZNRF3		
ATAD5		
XRCC2		
RAD51AP1	Yes	
NSD2		Yes
EXTL3		
FOXM1		Yes
ZRANB3		
BUB1	Yes	
CDC20	Yes	
KIF20B		
LSS	Yes	
CDCA2		
AURKA	Yes	
CENPO		
NYNRIN	Yes	
TRIB2		
FOXQ1		Yes
PTCH1		
PRLR		
MMS22L		
MEST		
RMND1		
BIRC5		
KIF15	Yes	
RERE		Yes
SLF2		
KIF18A		
SLC22A23		
NCAPG	Yes	
PGAP1		
CCND2		
USP51		
TET1		
PFAS	Yes	
DNMT3A		
CENPK	Yes	
BRIP1		
RACGAP1	Yes	
HMMR		
CEP126		
ATP8B2		
SYNE3		
DIAPH3	Yes	
PRC1	Yes	
TYW5		

Table S2. RNA-seq of TRIM71 in HuH-7 cells.

GeneID	siNC	siTRIM71	Fold change
KRTAP20-2	0.1	2.77903944	27.79039435
SLC16A14	0.169019737	4.26110013	25.21066605
MAB21L2	0.20962398	4.41026388	21.03892826
INSL4	0.1	1.56628402	15.66284023
CRLF1	0.228350444	3.35222927	14.68019594
TUBA1A	4.587029937	67.0917831	14.62641056
GPRC5A	0.163882596	2.33268948	14.23390608
KLRC2	0.497770747	6.61658241	13.2924292
COL7A1	1.96507294	24.8928249	12.66763407
SGK2	0.128636679	1.62629426	12.64253918
SNCG	0.687987993	8.06316903	11.71992698
COTL1	6.399453616	73.1937021	11.43749239
BTC	0.1	1.09836079	10.9836079
GIPR	0.493220075	5.40300428	10.95455062
LOC283278	0.183392528	1.9944636	10.87538094
CDKN2B	2.126706353	22.774599	10.70885924
KLRC3	0.988435516	10.398489	10.52014906
ANXA1	23.23694445	240.098301	10.3326107
SPP1	3.296808438	34.0063724	10.31493733
ACSL5	1.105544683	11.2235999	10.15209979
EMP1	0.1	0.98912771	9.891277099
PLEK2	0.490919255	4.36955737	8.900765927
KLRK1	0.176412536	1.56883521	8.892991654
KLRC4-KLRI	0.162362363	1.43497897	8.838125706
DUSP13	0.564267172	4.94396361	8.761742404
IGFBP3	3.330469948	28.9713084	8.698864996
SLCO2A1	0.28688773	2.4865845	8.667448079
LUM	4.116713815	35.2460601	8.56169791
C10orf107	0.156317032	1.33017117	8.509444904
TXNIP	0.180012864	1.51569801	8.419942749
CD52	0.1	0.83527696	8.352769618
MFSD14A	0.162437189	1.35474929	8.340142404
IBSP	0.13815824	1.13356028	8.204796745
CGB8	0.1	0.81763221	8.176322095
AKAP12	1.519495109	12.3836447	8.149841787
ADAM28	0.193819523	1.56808952	8.090462202
KIAA0319	0.126410859	1.01536437	8.032255968
HIST1H2BL	0.1	0.80258253	8.025825274
C15orf65	0.261462128	2.07507907	7.936442217
BHLHE40	5.040794326	39.2501671	7.786504373
CTGF	46.09852141	352.02086	7.636272263
C1QTNF9B-A	0.1	0.75976344	7.59763443
CLDN4	0.332096298	2.50455129	7.541641695
VCAN	1.429981861	10.6355125	7.437515643

SBK3	0.1	0.71807037	7.180703728
EREG	0.493537772	3.53852512	7.169714906
NHLRC1	0.1	0.69656961	6.965696062
IL12A	0.142071906	0.982245	6.913717366
NKG7	0.1	0.67797195	6.77971946
AGTR1	0.4269374	2.89068457	6.770745726
MAP1A	2.840989648	19.1289141	6.73318683
SIX1	0.190375527	1.26473907	6.643391015
GRK4	0.232844114	1.53155674	6.577605556
LTC4S	0.1	0.65669395	6.566939516
CPA4	0.250858474	1.64408535	6.553836199
PLK3	2.100662553	13.481802	6.417880853
LOC1053719[illegible]	0.1	0.64048743	6.404874319
AKR1B10	0.650031803	4.12493059	6.345736588
MYOF	1.644246617	10.3764166	6.310742257
B3GALT2	0.185554905	1.16149764	6.2595901
UBE2E1	0.725195363	4.4701014	6.163996117
SERPINE1	34.71788523	210.617071	6.066529394
GLIPR1	0.441851734	2.67961258	6.064506201
ARL14	0.28839336	1.74608164	6.054514013
HIST1H4H	3.18418568	19.1054209	6.000096368
UCN2	0.386173954	2.3146726	5.993859959
MFNG	0.1	0.59830889	5.983088851
C6orf52	1.065837095	6.36895228	5.975540081
HIST1H3E	0.888438445	5.26671891	5.928062819
EVA1A	0.132727634	0.78629625	5.924133707
CGB5	0.1	0.5822271	5.822270983
ARSI	0.540898164	3.13162031	5.789667114
NSUN3	0.523064531	3.02024633	5.774137129
HIST1H2AL	0.487509108	2.80867987	5.761286971
MNS1	0.160019536	0.91284943	5.704612446
CAV1	3.380071368	19.2670964	5.700204032
LYVE1	0.148567808	0.84262973	5.671684477
WBSCR27	0.367915976	2.06636293	5.61639901
CBR3	0.427570649	2.3897114	5.589044545
VSIG2	0.154156789	0.85710945	5.559985106
C19orf33	112.9296118	621.730377	5.505468121
ZBED8	0.266043789	1.46012771	5.488298451
ESAM	7.960760822	43.580423	5.474404267
LBH	0.119640815	0.64915541	5.425869195
SEMA3C	0.264270708	1.42253624	5.382875213
MEIOB	0.196671593	1.05728734	5.375902666
TGFB2	0.834145572	4.45656628	5.342672103
NTN4	0.486258502	2.59450809	5.335655987
NFIC	0.160198077	0.84952006	5.30293543
SOCS3	0.477079256	2.52258772	5.28756531

HIST1H4A	0.1	0.5277821	5.277821034
SPRY4	1.095741674	5.76404983	5.260409429
SIX4	0.129024587	0.67483007	5.23024401
PLEKHH2	0.271860609	1.42143194	5.228532148
ERMAP	0.5730207	2.99310347	5.223377559
PCDHB5	0.993401674	5.16486983	5.19917569
CHAC2	0.528539484	2.72104611	5.148236212
CD9	1.591843938	8.1744237	5.135191653
FOS	0.634515961	3.24631953	5.116214134
TM4SF19	0.501292088	2.55439477	5.09562155
CYP2C18	0.171563568	0.8711844	5.077910228
ARHGDIB	1.060470828	5.38002116	5.073238246
ZSWIM4	0.467094046	2.35629193	5.044577095
RND1	14.42969115	72.4833012	5.023205305
IL11	1.836023421	9.20107324	5.011413872
EDN1	1.545470482	7.72837343	5.000660655
CLEC2B	0.576622259	2.85896367	4.958122287
CLDN7	0.371970056	1.84264248	4.953738741
CYP2C9	0.1	0.49464537	4.946453715
EPB41L1	0.4999671	2.46608457	4.932493712
C15orf57	2.250137342	11.0681549	4.918879698
FSCN1	8.37268192	41.0410117	4.901776044
FBXL2	0.808280536	3.95460194	4.892610626
HIST4H4	0.1	0.48624986	4.862498603
GNG11	0.22727413	1.10084869	4.843704351
NOXRED1	0.158515432	0.76466	4.823883663
PGM2L1	0.773450166	3.70487843	4.790067399
ZNF117	0.204279726	0.97800874	4.787595717
ZNF596	0.113738348	0.54414218	4.784157572
HIST1H4K	0.271496498	1.29875663	4.783695698
F2R	0.577423279	2.7509034	4.764102008
SNX16	0.363497223	1.72695832	4.750953276
PRSS23	14.11156788	66.8717755	4.738791326
BBOX1	0.156082919	0.73702979	4.722040026
TIGIT	0.118720247	0.55798935	4.700035297
THBS1	2.011590296	9.42554351	4.685617907
CASP7	1.157582925	5.3887694	4.655190815
SERPINE2	1.189778793	5.51558705	4.635808842
CD68	3.469130013	16.0477906	4.625883293
GABARAPL1	7.502045633	34.621787	4.614979524
UBASH3B	0.465201445	2.13059644	4.579943734
KLF4	0.690007723	3.15755251	4.576111841
MLLT11	3.278963349	14.9958565	4.573352895
SCN1B	2.122771494	9.68111885	4.56060338
HACD1	4.605621073	20.8390858	4.524706986
LIF	0.52828755	2.37201984	4.490016541

C6orf99	0.906156162	4.05534586	4.475327798
RGS5	0.130666658	0.5823083	4.456441336
ABLIM3	0.319588494	1.40919527	4.409405519
SLC44A5	0.428847649	1.87621997	4.375026823
ULBP2	0.401182368	1.75413122	4.37240358
SLC22A14	0.120297479	0.52563676	4.369474409
KLF2	3.9635457	17.3012147	4.365085215
TAC3	0.37273028	1.62510277	4.359996657
SYT1	0.641152506	2.79403406	4.357830675
TCTEX1D4	0.1	0.43414161	4.341416114
DUSP5	11.98739372	52.0118997	4.338883077
PLK2	1.532741788	6.58348304	4.295232954
C8orf4	1.320130977	5.66093498	4.288161613
ME3	0.76263878	3.26859282	4.285899047
ARHGAP25	1.031193388	4.41576369	4.28218775
MICALL2	4.905780697	20.9952092	4.279687676
ERICH5	0.609178634	2.59824748	4.265165155
TRAM1L1	0.189851643	0.80656857	4.248414983
DEFB133	0.42639656	1.81112841	4.247521161
C1S	1.050277286	4.45908594	4.245627319
OLFM2	8.128482409	34.4912233	4.243254963
SERPINI1	2.620607767	11.1118694	4.240187935
MSANTD3-TI	1.247349618	5.28681407	4.238438035
CCDC159	2.744703469	11.6195212	4.233434075
ADRB1	0.14242471	0.60042435	4.215731573
SPOCK2	2.073603513	8.72215236	4.206277769
ERICH2	1.032018553	4.34022875	4.205572404
OXCT1	0.407132008	1.71013432	4.200441851
SATB1	0.338077491	1.41522027	4.186082505
C17orf97	0.1	0.41728917	4.172891729
WDR78	0.510201083	2.12640649	4.167781222
C4orf32	1.433079688	5.96206231	4.160314575
CYP2S1	0.817226588	3.39509599	4.154412054
UGDH	11.06385967	45.9062917	4.149211314
ITGA2	0.437702838	1.81004679	4.13533254
PNMA1	4.533213668	18.6848098	4.121758016
LOC1053713(	0.1	0.41136328	4.113632812
AXL	4.580897529	18.8145198	4.107168887
KRTAP20-1	0.1	0.41020457	4.102045662
TAF13	2.204324445	9.01744045	4.090795468
RIN2	0.433635581	1.76578939	4.072058346
GLCCI1	1.61964356	6.59507796	4.071931702
TNFRSF10A	0.620369373	2.52491341	4.070016214
CLCF1	5.538170705	22.504449	4.063516675
C8orf48	0.14198462	0.57689125	4.063054526
SLC10A4	0.443964647	1.80304943	4.061245513

SPANXB1	0.358890236	1.45722765	4.060371417
DNAAF3	0.126190578	0.51191098	4.056649781
TMSB4X	113.2189809	459.289063	4.056643676
AQP3	1.851243084	7.50911675	4.05625648
VPREB3	0.203777368	0.82540145	4.050505979
FRMD3	0.361821068	1.45829466	4.030430465
SUMF2	10.88362949	43.8587118	4.029787289
KIF3C	1.195480851	4.81749691	4.029756652
UGT2B11	1.157661328	4.65903436	4.024522754
ELF1	1.044503211	4.20089249	4.021904807
CAPN8	0.275100256	1.10538489	4.018116527
RAET1G	0.111351862	0.44715302	4.015676129
GPSM3	0.191984485	0.76975986	4.009489923
CLPSL2	0.1	0.39912583	3.991258317
PTHLH	0.304409967	1.21283177	3.984205172
PFKP	0.437276446	1.73923725	3.977431831
HIST1H4B	0.1	0.39717076	3.971707609
YY1	0.887939275	3.52550683	3.970436867
TTC39A	1.498746195	5.93713682	3.961402428
EHD2	0.1	0.39600994	3.960099376
TEX15	0.292996466	1.15934303	3.956849873
NEK11	0.279402365	1.10321477	3.948480422
CYTH3	1.974680446	7.77959333	3.939672034
MAP1LC3B2	0.532757965	2.09863981	3.939199307
EEF1A2	5.87756064	23.1469473	3.938189453
LTBP1	2.507671009	9.8390216	3.923569545
FOSL2	1.265224292	4.95666664	3.917618932
RHOBTB1	0.490692226	1.91506919	3.902790978
PPP1R1C	0.783198302	3.05154942	3.89626664
LOC1053788€	0.143488367	0.55597405	3.874697713
MAP3K7CL	0.396145172	1.53491966	3.874639318
GPC1	1.961151087	7.59767634	3.87409027
FBN1	0.203821736	0.789426	3.873119796
UCN3	0.527533523	2.04249226	3.87177718
PIGB	0.818458701	3.16632342	3.868641656
C9orf43	0.324435343	1.252086	3.859277434
LMCD1	13.79737055	53.2390322	3.858636108
ZFP36	4.431461204	17.0852935	3.855453704
ELK3	0.680074614	2.62183083	3.855210552
QPCT	0.95556914	3.68201573	3.8532175
CALCOCO1	4.06139241	15.5514415	3.829091091
IL15	2.353953972	9.00504924	3.825499288
ITGAV	9.051517327	34.3269477	3.792397056
RAB27B	0.178264484	0.67442032	3.783256769
SLC10A7	0.487600739	1.84349783	3.780752743
LGR5	0.639817864	2.4123056	3.77030049

CYB5R4	1.153707454	4.34926415	3.769815421
ZNF229	0.188417637	0.70838045	3.759629174
LPXN	0.370375774	1.3902342	3.753577588
DKK3	9.338441775	34.9251831	3.739936908
COL12A1	0.425071693	1.5888694	3.737885689
IQCG	0.35594334	1.32996372	3.736447831
OSBPL10	0.530565675	1.98102769	3.733802958
FRRS1	0.189608502	0.7067876	3.727615562
LOC728715	0.943191114	3.50740328	3.718655984
RASSF2	0.142798562	0.53004391	3.71182943
LOC1053713[illegible]	0.172880865	0.64127531	3.709348145
G3BP2	4.806927613	17.8216279	3.707488295
S100A3	1.90977605	7.07203677	3.703071244
PKIB	1.529538442	5.65430911	3.696742074
GABRR1	0.620954118	2.29462838	3.695326782
CCDC74A	0.199091012	0.7349236	3.691395166
GOLM1	24.4586117	89.8882496	3.675116588
AGR2	2.826996529	10.3754174	3.670120316
RAB30	0.299345481	1.09756233	3.666540483
SPERT	0.528395486	1.93589819	3.663729624
CNR1	0.268059352	0.98104829	3.659817444
IKBKG	0.77186543	2.82386798	3.658497809
QRFP	0.230278455	0.8398978	3.647313855
C11orf70	0.431445461	1.57213095	3.643869489
HPS3	0.438174978	1.59024407	3.629244368
DCAF12L2	0.123358892	0.44696602	3.623297909
ZNF680	0.250478613	0.90349048	3.607056404
SLC25A53	0.14876286	0.53458539	3.593540662
TLDC1	1.705134671	6.12547765	3.592371765
LMBRD1	2.914533945	10.4653754	3.590754325
COL1A1	1.144777382	4.10947383	3.589758058
TTC5	1.038477104	3.71927374	3.581469183
DMTF1	0.273093785	0.97719318	3.578232956
BBS9	0.463803807	1.65956217	3.578155561
PANX2	2.168746745	7.747376	3.572282482
ZNF543	0.149612988	0.53349208	3.565813938
CSNK1G3	0.751968366	2.68020437	3.564251494
RGS20	1.138266683	4.05632398	3.563597217
SRD5A1	1.279454897	4.55945088	3.563588595
NLRP1	2.879691498	10.2591581	3.562589291
SMPX	0.1	0.35576172	3.557617204
MUC13	0.1421742	0.50534546	3.554410443
CD44	1.921716462	6.81853735	3.548149522
ALPK1	0.130871986	0.46415319	3.546619876
PRKD1	0.333439222	1.18102435	3.541947887
C3orf52	1.785515194	6.31812629	3.538545237

KIF3A	0.453909111	1.60434797	3.534513689
SUSD1	5.495243844	19.3272599	3.517088685
SPACA6	5.357799419	18.748474	3.499286279
AK9	0.302169314	1.05682635	3.497464175
TNFRSF11B	0.229389941	0.80093538	3.491588946
ELMO1	0.132881257	0.46391213	3.491178053
LOC10272389	0.194711014	0.67927436	3.488628332
KLF7	0.445555377	1.55302865	3.485601851
RASSF6	0.128395585	0.44734786	3.484137433
PRH1-TAS2R	0.135399277	0.47116022	3.479783834
SFR1	1.88386097	6.53898611	3.471055568
TRIM68	0.49845308	1.72996212	3.470661909
HIST1H2BF	0.1	0.34672084	3.46720841
LCP1	6.267086462	21.7238774	3.466343976
ARFGEF3	0.365161565	1.26554957	3.465725016
TGFB1	87.58034363	302.59809	3.455091381
SMOX	52.08093547	179.763327	3.451614791
TSPAN12	0.431359315	1.4855804	3.443951118
TIMP2	7.524695711	25.8750714	3.438686743
PHACTR2	0.805854162	2.77069612	3.438210344
MAFK	2.973940429	10.2223736	3.437316189
ARHGAP21	1.347371365	4.63073524	3.436866301
JRKL	0.185027675	0.63490737	3.431418398
HIST2H2AA3	0.782132137	2.68271442	3.430001522
FLRT2	0.52313704	1.79402104	3.429351971
LOC10798399	0.139294343	0.47766653	3.429188287
ITGB5	35.63877289	121.958127	3.422063031
ASMTL	0.479605468	1.63993915	3.419350398
ASPH	14.1622718	48.4095178	3.418202849
ARHGAP11B	0.189542419	0.64766204	3.416976772
MAPK13	0.779015907	2.65698868	3.410698877
CNGB3	0.197761289	0.67352521	3.405748502
FHL2	4.662940401	15.8777715	3.405098524
MEF2C	0.392281809	1.33571162	3.404979762
TMEM107	0.373467388	1.26483911	3.386745791
RBM24	0.460161722	1.55544256	3.380208489
LGALS1	45.4691219	153.626244	3.378693877
CBLB	1.477994558	4.9917998	3.377414196
BCAR3	1.282109379	4.31999013	3.369439613
PDP1	0.239242569	0.80564424	3.367478632
LRP10	14.13089538	47.5794799	3.367053442
SSFA2	2.240276293	7.53528799	3.363552975
KIAA0586	1.096066666	3.67794286	3.355583168
SSBP2	0.637254515	2.13417331	3.34901245
LAT2	0.377086993	1.26209908	3.346970593
PLSCR4	0.223789199	0.74896277	3.346733325

DZANK1	0.235968108	0.78905014	3.343884686
STAC3	0.622159635	2.0770205	3.33840446
SNCAIP	0.431857228	1.43557016	3.324177692
IL31RA	0.533534363	1.77194227	3.321139913
WRB-SH3BG	0.357244747	1.18637319	3.320897523
CTTNBP2NL	0.336665167	1.11594624	3.314706575
GAD1	0.187327043	0.61885194	3.303591044
JKAMP	1.462408764	4.80749405	3.287380495
ANKRD44	0.295690642	0.97201913	3.287284052
ACO2	5.004630876	16.4228726	3.281535241
JUNB	31.95925193	104.729472	3.27696882
DDX60L	0.280658207	0.91931457	3.275566316
CDK6	0.807955629	2.64601564	3.274951677
KAT2B	0.85509652	2.79403406	3.267507226
FAM161A	0.236398563	0.77172974	3.264528059
VLDLR	0.514479683	1.67937834	3.264226744
GAP43	0.118116908	0.38493275	3.258913206
RCAN2	0.135523558	0.44142469	3.257180476
CEACAM1	1.359395424	4.42620332	3.256008697
TLE6	0.160198077	0.52078319	3.250870426
C12orf60	0.661515737	2.14907162	3.248708233
CFAP53	0.198395073	0.64449197	3.248528095
CCDC191	0.341604175	1.10859349	3.245257434
ATG4A	1.898511659	6.15879142	3.244010324
KATNAL1	0.555401719	1.801391	3.243401922
NOG	0.1	0.32429167	3.242916736
MAFF	1.527885683	4.93987051	3.233141437
GNG3	0.1	0.32311243	3.231124325
HLTF	0.858447499	2.77054723	3.227392751
PDE5A	1.003393199	3.23457964	3.223641182
CHN1	1.615939316	5.20896962	3.223493335
RGS2	4.731929855	15.2276427	3.218061794
BICD2	1.689657064	5.41607273	3.205427215
EMSY	0.403001715	1.291361	3.204356092
HECA	0.629225805	2.01569669	3.203455222
NR2F1	5.767112812	18.4693858	3.202535896
HABP4	18.73816782	60.0028826	3.202174468
KISS1R	0.39887882	1.27515034	3.196836436
NEDD9	0.441614406	1.41120029	3.19554858
ETV1	0.412017857	1.31512731	3.19191824
LOC10099663	0.128900235	0.41143515	3.191888255
NELL2	0.394946204	1.25968784	3.18951753
ACRC	0.214618805	0.68419582	3.187958412
RCAN3	1.365695936	4.35198804	3.186644936
ZCCHC11	0.66212741	2.10848224	3.184405619
HEY1	0.284368035	0.90545524	3.184096408

SPHK1	4.380816427	13.9441571	3.183004199
PCDHB3	0.607889796	1.93423804	3.181889311
CCDC68	1.186422475	3.77321789	3.180332445
CFI	0.943569058	3.00071164	3.180171724
TIMP4	6.382883944	20.272035	3.175999303
CD55	0.662513221	2.10238431	3.173346956
FGF18	0.191891665	0.60850385	3.171080158
MYC	13.95598923	44.2443648	3.170277944
TMEM156	0.462110869	1.46376442	3.16756111
PCED1B	2.544178894	8.05873681	3.167519715
FOSB	0.24470017	0.7743006	3.164283063
KITLG	0.836146275	2.64358626	3.161631326
NCK1	0.672347908	2.12416313	3.159321393
LYPD6	0.351539343	1.10841055	3.153019929
MMP24	0.478297606	1.50784048	3.152515212
KCTD1	0.276944798	0.87302508	3.152343295
RHOH	0.180303916	0.56776879	3.148954299
AMIGO2	8.319861101	26.1629423	3.14463691
OXTR	0.483989726	1.5194651	3.139457355
VIM	281.1118861	880.801967	3.133278993
EPS8	2.330882909	7.29914732	3.131494634
HYPM	0.1	0.31175641	3.117564104
CCSAP	0.660729579	2.05974352	3.117377491
FLNC	31.32519951	97.6219608	3.116403481
IL15RA	0.652157892	2.02978557	3.11241433
GPR157	0.187143197	0.5822271	3.111131525
FAM171A1	3.074936213	9.56574571	3.110876144
CEP290	0.678057114	2.10226464	3.100424126
INTS2	0.218112374	0.67620196	3.100245767
ARRDC3	1.967550599	6.09988188	3.10024143
ZNF671	0.493546723	1.52973507	3.099473662
RANBP6	0.336429183	1.04108361	3.0945104
LOC730183	0.621561514	1.92285157	3.093582101
SMIM14	2.792639752	8.63444116	3.091856425
A4GALT	0.348504954	1.07708724	3.090593783
VNN2	1.566097393	4.83581241	3.087810779
ANKRA2	0.486686371	1.49973107	3.081514416
PPP1R3B	0.801601151	2.46849415	3.079454351
UGT2B15	0.314901806	0.96843553	3.075357168
PRDX4	61.66257513	189.29687	3.069882652
USP50	0.405293335	1.24321079	3.06743459
TUFT1	2.981327798	9.12790937	3.061692638
NIPSNAP3A	2.330609684	7.11635999	3.053432771
C8orf88	2.117893265	6.44726266	3.044186772
NABP1	2.158478712	6.56955485	3.043604187
IGFBP7	5.423530791	16.4974299	3.041824687

ZNF211	0.893493784	2.71627526	3.040060609
DACH1	0.13876122	0.4217413	3.039331201
GRIA3	1.575869147	4.78736277	3.037918963
STX12	3.215343331	9.74767433	3.03161228
GABRA2	0.308082147	0.93382371	3.031086752
PLEKHB2	5.209420097	15.7843606	3.029965004
BTBD16	0.22389747	0.67736034	3.025314827
PCMTD1	0.605446662	1.82972764	3.022112019
TBC1D4	0.723120706	2.18446552	3.020886421
SYTL5	0.991960398	2.99049272	3.014729954
FBXL14	0.406333653	1.21929776	3.000730441
DMRTA1	0.680197535	2.03561181	2.992677431
LYPD1	2.515384493	7.52710902	2.992428808
FOSL1	12.35970058	36.9561247	2.990050161
COPG2	2.146830339	6.41005815	2.98582428
TGFB3	0.609133328	1.81805606	2.984660298
BMPR2	0.923932529	2.75576079	2.982642893
LOC1005075(	5.343349318	15.910189	2.977568579
KLHL21	5.513531358	16.3936072	2.973340708
BRINP2	0.214357919	0.6370695	2.971989582
ZNF419	0.818458701	2.43164751	2.971008196
SLC38A5	1.440305795	4.27865629	2.970658248
TMEM206	1.428495992	4.23934821	2.967700461
OXR1	0.263706053	0.7821798	2.966104832
MAPRE2	3.526597617	10.4439553	2.961481986
LOC1002895(	4.065671178	12.0236556	2.95736057
NEK1	0.40882004	1.20723605	2.952976694
RASSF10	0.167857407	0.49526811	2.950528796
PLEKHA1	2.597643245	7.66368357	2.950244835
CAV2	7.448042177	21.9359921	2.945202455
TTLL7	0.441351685	1.29936612	2.944060627
MGARP	0.23601888	0.69423055	2.941419574
TMC7	1.813745177	5.33493476	2.941391563
CIDEC	8.071052489	23.7070411	2.937292393
POLD4	59.74044059	174.967602	2.928796637
KLHDC1	0.1	0.29268064	2.926806423
ZFC3H1	0.931175818	2.72334775	2.924633244
ARNTL	0.160168246	0.46800284	2.921945216
WRN	0.670540733	1.95555885	2.916390835
TMCO3	7.562615645	22.0521455	2.91594159
S100A6	529.7771028	1544.77295	2.915892249
SLC15A1	0.546538051	1.59279127	2.914328235
EID1	10.29087797	29.9837816	2.913627167
CCPG1	1.470230165	4.27535609	2.90795019
TCTEX1D2	28.89019558	84.0032539	2.907673424
GABPB1	2.922798022	8.49265115	2.905657895

TFPI	8.353547772	24.2338391	2.901023587
ZSCAN29	0.529077935	1.53297262	2.897441991
FAM183A	1.096330857	3.17591245	2.896855847
ZNF616	0.205849954	0.5960618	2.895612958
TUBB3	70.38501267	203.797374	2.895465477
CD200R1	0.260325071	0.75372336	2.895316045
BHLHE41	0.437276446	1.26474429	2.892322008
CD72	0.320992367	0.9275348	2.889585229
GJA1	15.5983263	45.0601825	2.888783174
ZNF605	0.354959419	1.0253526	2.88864738
GAREM1	0.410579987	1.18547353	2.887314454
GCNT3	3.433299167	9.91165037	2.886917185
GRAMD3	0.319678636	0.92261537	2.886071389
ANXA8	1.195844048	3.4512172	2.886009431
RUSC2	3.309990871	9.54946074	2.885041414
RASA1	0.573794796	1.65521036	2.884673013
MAP7D3	0.976195971	2.81430691	2.882932317
DPY19L1	2.049395971	5.90807628	2.882837853
TMEM150C	0.132155593	0.38095858	2.882651995
BNIP3L	4.193572404	12.0881657	2.882546073
LOC10272447	0.132087611	0.38036459	2.879638664
PCDHB13	0.134661582	0.38771186	2.879157161
AGA	3.015685109	8.67598358	2.876952756
PPL	1.865890421	5.36465288	2.875116793
GPR19	0.166762933	0.4791705	2.873363359
MXRA8	7.006798774	20.1285253	2.872713485
STK39	0.826478645	2.37376235	2.872139967
AOX1	0.469131092	1.34710535	2.871490227
CDKN1A	0.914367186	2.62485846	2.870683129
RHOB	67.43031935	193.407077	2.868250949
RASSF9	0.503713359	1.44423837	2.867182983
CAT	5.722251709	16.4055658	2.866977316
CDK20	0.3257801	0.93293772	2.863703815
SLC9A6	0.780257356	2.2307709	2.859019378
HIST1H2BD	35.23387862	100.718438	2.858568005
CSAD	1.615333248	4.61369816	2.856189686
SERF1B	0.259594533	0.74144824	2.856178193
KLK14	0.460754093	1.31580177	2.855757089
ITGB1	11.06540631	31.5020804	2.846897755
CEP135	0.311716688	0.88619414	2.842947364
ABCA3	0.136666019	0.38833147	2.841463237
GOLPH3L	1.478461916	4.19839808	2.83970661
THAP2	0.413583472	1.17405126	2.83872868
PARP4	2.694069235	7.64587471	2.838039429
ZNF274	2.174408735	6.16941003	2.837281662
ATP1B1	11.11054288	31.5170955	2.836683662

PTGR1	227.2784993	644.02667	2.833645383
PPP2R3C	3.337853879	9.45612424	2.832995265
MORC4	4.480213581	12.6659707	2.827090819
GPR158	0.21844918	0.61747317	2.826621618
CCDC53	7.157883456	20.22051	2.824928638
WWP1	0.955462087	2.6977214	2.823472993
ZSCAN30	0.602888028	1.69891682	2.817964098
GPSM2	0.522121706	1.47128226	2.817891398
TCP11L1	0.628818542	1.77190484	2.81783173
WBP4	3.221921372	9.0723342	2.815814897
C17orf67	0.250869185	0.70637901	2.815726467
LURAP1L	3.23704709	9.11173094	2.814828048
KLHL18	2.515030665	7.07015089	2.811158919
CTAGE4	0.423662088	1.19079549	2.810719973
MIA2	0.126615414	0.35580657	2.810136293
AGBL2	0.14641419	0.41091604	2.806531561
LYRM5	0.451329631	1.26649842	2.806149505
BACH2	0.478707717	1.34212815	2.80364845
ACOT6	0.173498499	0.48531463	2.79722668
USP53	0.757605346	2.11901311	2.796988064
USP15	2.03731397	5.69803013	2.796834563
UBE2E3	16.47135506	46.0427838	2.795324585
SETD7	2.03894994	5.69430621	2.79276411
CSGALNACT	0.470558048	1.31415266	2.792753558
PTN	0.213053557	0.59390112	2.78756728
ARL13B	0.389311624	1.08453187	2.785768025
SLC38A2	27.48402985	76.5395117	2.784872236
PFKM	5.954032467	16.5731293	2.783513428
CCDC40	0.196980448	0.54789235	2.781455517
AMPD3	0.398594841	1.1080088	2.779787109
PTPN1	6.593660486	18.3276969	2.77959367
LIPT1	0.568383907	1.57959294	2.779095111
PERP	12.94190651	35.9072116	2.774491654
USP54	0.625237428	1.73421971	2.77369785
PHTF1	3.19276137	8.83597932	2.76750383
DPP4	5.159146686	14.2725308	2.766451826
DDHD2	3.15547105	8.72868025	2.766205144
SLC41A3	4.493639523	12.4297401	2.766074148
NPIPB3	0.301136699	0.83226092	2.76373129
TCTN1	2.690283103	7.43199513	2.76253273
CNPY3-GNM	0.9385326	2.59106264	2.760759334
EGFR	2.60427761	7.18529387	2.759035304
SYTL2	0.523193877	1.44282879	2.757732563
LVRN	0.41530713	1.14459928	2.756030885
ANGPT1	0.517058119	1.42399883	2.754040177
AKR1C2	67.51039378	185.889825	2.753499339

SVIP	5.249243171	14.4460015	2.752016049
AREG	6.427898206	17.6846606	2.751235318
UCHL1	503.7641585	1385.21582	2.749730804
NPTN	19.69206	54.1220181	2.7484183
PKIA	0.215610112	0.59244329	2.74775279
ATF1	0.685282526	1.87823884	2.740824065
GIP	0.52693221	1.44291091	2.738323602
CHMP3	5.197185383	14.2253865	2.737132784
PLXND1	11.63807397	31.8494396	2.736658975
HSD17B3	2.587619444	7.07347431	2.73358369
C1orf61	0.1	0.27334961	2.733496105
DYSF	0.948353051	2.5919987	2.73315797
EFCAB7	0.430597704	1.17665555	2.732609904
C14orf105	0.62889648	1.7174263	2.730856909
LOC10798426	0.1	0.27264436	2.726443639
TCTE3	0.324297144	0.88391597	2.725636009
FAM72A	0.352957334	0.96198094	2.725487884
KRTAP6-3	0.1	0.27222396	2.722239637
KLF3	1.581253277	4.30392101	2.721841637
ZNF83	1.122645783	3.05553505	2.721726743
QTRT2	1.819481408	4.94982682	2.720460234
LMLN	0.312153202	0.84888776	2.719458775
SP4	0.50345413	1.36906085	2.719335814
CITED2	28.19707912	76.6535519	2.718492633
KIAA0226L	0.801846939	2.17967253	2.718314963
TSPAN7	2.587553852	7.03168077	2.717501228
KIAA1217	0.666175311	1.80951243	2.716270622
ATP8B3	0.215880436	0.58600097	2.714470016
WDR60	0.608770011	1.65134115	2.712586238
TSC22D2	0.950106665	2.57239058	2.707475571
LOC728392	4.32894512	11.7180233	2.706900396
IL4I1	0.427888194	1.15781432	2.70588051
CCDC66	0.276430573	0.74745399	2.703948357
ERO1A	2.60020239	7.0289525	2.703232844
MOK	1.990020838	5.37947686	2.7032264
PCDHB9	0.223841306	0.60496129	2.702634723
AGMO	0.238238543	0.64353491	2.701220798
STX11	0.144120465	0.38912599	2.70000506
GINM1	5.84799353	15.7886868	2.699846838
KIF20A	2.618641738	7.06874424	2.699393405
RNF103-CHM	4.149717181	11.1870772	2.695864979
SAT1	58.19048571	156.726906	2.693342453
BBC3	5.139931262	13.8289505	2.690493284
ZC2HC1A	0.232656288	0.62499531	2.686346093
NEK7	1.745444833	4.68814706	2.68593253
CRISPLD2	1.603957727	4.30505215	2.684018464

ZNF146	4.239659565	11.3773877	2.683561617
BAGE2	0.1	0.26830771	2.683077128
TMEM133	0.623360002	1.67191484	2.682101571
HERC1	0.637744392	1.70993624	2.681225055
NCSTN	7.28879399	19.5130135	2.677125118
DKK1	14.26000502	38.1572023	2.675819697
CPQ	4.152869225	11.1116238	2.675649826
FUBP1	1.766577388	4.7176855	2.670522975
PHEX	0.222474687	0.59390112	2.669522241
CADM1	7.771219113	20.7095671	2.664905834
RAPSN	0.562347325	1.49837384	2.664498913
LOC653513	0.197291282	0.52503991	2.661242321
GBA	8.39676553	22.3201584	2.658185269
EMC6	17.35509427	46.1297513	2.657994859
HOXA10	0.643105595	1.7092993	2.657882802
ATP10D	0.220492654	0.58590822	2.65726867
SENP8	0.21662494	0.57553665	2.656834659
ERLIN2	2.82003904	7.48294589	2.653490176
MANEA	0.233321494	0.61890678	2.652592222
FILIP1L	1.752846219	4.64599504	2.650543438
TNS1	0.917764216	2.43255934	2.650527547
UBE3D	1.62672474	4.31116465	2.650211524
SPRY2	3.261032568	8.64047697	2.649613824
CHML	1.173824824	3.10991047	2.649382096
ZFYVE28	0.30485831	0.80702775	2.647222413
RGS3	2.34787892	6.20550457	2.643025804
C14orf178	0.1	0.26422595	2.642259468
C9orf135	0.358614527	0.94736391	2.641733224
TSPAN13	34.18939218	90.2095337	2.638524054
LOC10537919	0.242177424	0.63831753	2.635743308
ARRDC2	0.412194178	1.0862002	2.635166274
ATP2C2	0.182422095	0.48065638	2.634858344
PARM1	0.313599626	0.82579137	2.633266447
PPP1R3C	0.335949321	0.88340819	2.629587663
KCTD9	1.579561767	4.15343721	2.629487052
ZNF608	0.344607743	0.90558006	2.627857548
CLEC18A	0.186255875	0.4893901	2.627514983
XRCC4	1.798175111	4.72443423	2.627349358
MYO5A	0.512180785	1.34530957	2.626630301
CFHR1	0.334039168	0.87706603	2.625638276
GNAT2	0.1	0.26251526	2.625152599
GIN1	0.560957122	1.47236733	2.624741312
SLC15A4	1.164351245	3.05403468	2.622949643
DSG3	0.395291574	1.03648138	2.622067975
PIK3CD	1.78126011	4.66810448	2.620675358
SH3RF1	1.241551047	3.25348129	2.6204974

MAP1B	3.888669423	10.1857062	2.619329423
YIPF5	3.141641622	8.22649721	2.618534574
CSF1	3.727673439	9.75934818	2.618080243
CCP110	0.273514782	0.71535032	2.615399102
GMFG	0.192124067	0.50246571	2.615318941
OGFR	16.90807411	44.2018601	2.614245702
BBX	0.435635561	1.13814259	2.612602585
AKR1C1	140.505675	366.832796	2.610804127
TNFRSF1A	38.06405492	99.3746586	2.610721817
TARSL2	1.321496101	3.44904712	2.609956338
GPR153	0.826367076	2.1563022	2.609375738
TES	22.53336935	58.7888753	2.608969584
HCAR1	0.140785865	0.367274	2.608742039
TMPPE	0.14586484	0.38051992	2.608715836
RAB5A	3.633459245	9.47692812	2.608238453
LOC10798484	0.1	0.26074237	2.607423661
GXYLT1	1.478109125	3.85401787	2.607397387
DCBLD1	13.61454882	35.4964766	2.607245901
NOLC1	7.614114514	19.849795	2.606973528
CCNE2	0.339564986	0.8849471	2.606120001
PIK3R1	0.326674784	0.8512486	2.605798316
CCDC153	0.397305388	1.03517106	2.605479547
SPA17	4.132977872	10.765377	2.604750701
ZNFX1	2.916814272	7.58430064	2.60020006
ZNF845	0.237352399	0.61697162	2.599390707
BLVRB	72.29922421	187.740423	2.596714209
EOMES	0.1	0.25942481	2.594248057
TAX1BP1	12.65944037	32.8389366	2.59402751
HCN4	1.091111194	2.83004052	2.593723293
P2RY1	0.153988226	0.39875638	2.589525098
ZNF468	0.779903988	2.01833225	2.587924004
CEP19	0.513554225	1.32856223	2.586995027
MBL2	0.130054337	0.33633902	2.586142314
STPG1	0.803275244	2.07631582	2.584812407
RP2	0.924042687	2.38463112	2.580650395
CD47	0.327122186	0.84417762	2.580618673
BTN2A1	2.748668133	7.092747	2.580430469
LOC10798399	0.64378544	1.66114467	2.580276851
GPX8	0.853262601	2.20051402	2.57894114
KIDINS220	0.743304075	1.91596728	2.577635918
LYG2	0.129832806	0.33459697	2.577137297
SNX6	2.485026295	6.40339707	2.576792481
RASEF	0.480499899	1.23736558	2.575163036
ISCA1	13.15683965	33.8222084	2.570693971
PVRIG	0.559342448	1.43783054	2.570572897
ATP2C1	4.960649993	12.7352912	2.567262598

HEATR5B	0.536111728	1.37455119	2.563926723
MEX3D	5.85145097	14.9898192	2.56172687
ADGRG6	6.806767077	17.4362062	2.561598774
MAP2	0.858721463	2.19928063	2.561110591
LMO7	4.891121622	12.5203592	2.559813513
BBS1	3.270598754	8.36368396	2.557233273
RASA4	0.164976554	0.42171412	2.556206372
ZNF720	0.428279165	1.09435436	2.555236053
NINJ2	6.951101443	17.7525307	2.553916214
ZNF664	3.006318309	7.66909185	2.5509913
CPT1A	0.724086627	1.84696015	2.550744736
NODAL	0.129103433	0.32886137	2.547270573
CDC42BPA	1.459089915	3.71648019	2.547122116
CARD6	1.305504359	3.32115677	2.543964517
PCDHB8	0.323938621	0.82396322	2.543578216
LNPEP	0.24939762	0.63400987	2.54216489
DCAF6	6.813824743	17.3172539	2.541488017
ULBP3	0.756530486	1.92215583	2.540751318
CCDC103	0.911718243	2.3163641	2.54065784
SDCBP	10.73578733	27.2744985	2.540521499
LDLR	8.014308678	20.3395682	2.537906767
ANKDD1B	0.377505936	0.95797086	2.537631239
GCLC	5.083518215	12.8986841	2.537353762
IGF2R	4.824848187	12.2394594	2.536755347
DNAJC2	12.10698976	30.6989636	2.53563968
HIST1H2AD	1.885581183	4.78043948	2.535260495
LEPR	0.442859405	1.12267831	2.535067094
ANK3	0.192156339	0.48712182	2.535028653
KLHL20	0.28839336	0.73099796	2.534725355
ISM1	0.217109877	0.55022746	2.534327172
TTC26	0.505272098	1.28035115	2.533983489
TVP23B	2.842910854	7.20106838	2.532991273
DHFRL1	0.366206013	0.92701066	2.531391156
RIBC1	0.31430576	0.79549647	2.530963706
ZW10	1.994605815	5.04822582	2.530939089
NEDD4	1.06431249	2.6936358	2.530869296
KIAA0556	1.354223618	3.42648378	2.530220071
HIC1	0.119230206	0.30162806	2.529795639
AASS	1.094077945	2.76775927	2.529764247
HMMR	1.394986926	3.52824932	2.529234684
TIGD6	0.46416752	1.17389239	2.529027429
CD46	3.720708223	9.40915519	2.528861343
ASAP2	1.629159607	4.11757507	2.527422762
ARFIP1	0.714807088	1.80535222	2.525649578
U2AF1L5	1.664087334	4.19985062	2.523816226
SLC22A1	0.256267079	0.64615167	2.521399436

C20orf194	1.005066624	2.5338195	2.521046306
NID2	0.68067114	1.71526781	2.519965534
NEIL3	0.706592109	1.77835909	2.516811426
CAP2	1.077068255	2.71038439	2.516446263
IL6R	0.452963722	1.13975212	2.516210592
ETV3	0.52259954	1.3146222	2.515544124
STON1	0.196997664	0.49550669	2.515292219
CNTRL	0.617012152	1.55103689	2.513786625
CDKN1B	2.482190354	6.23917336	2.513575703
CD247	0.1	0.2511253	2.511253015
LCTL	0.461114739	1.15792999	2.511153712
ZNF165	0.457416587	1.14840795	2.510639055
HARBI1	0.475330821	1.19314747	2.510141179
ZNF827	0.501908293	1.25922616	2.508876977
TDG	4.741190055	11.8895904	2.507722794
LOC10537894	0.205582192	0.51513463	2.505735678
KCNE3	0.121179055	0.30355653	2.505024728
ZNF256	0.566998156	1.42012396	2.504635937
MAP4K3	1.248600433	3.12285051	2.501080755
ZNF43	0.283511011	0.70835727	2.498517656
PIGN	0.641420894	1.60146708	2.496749163
CTSO	0.720965839	1.80004371	2.496711508
PPTC7	0.164791633	0.41136328	2.49626315
PIGK	1.569842876	3.91841302	2.496054271
GBP2	2.832159546	7.06913249	2.496021985
IQGAP1	7.989742766	19.93485	2.495055292
BEST3	0.655094054	1.63161639	2.490659738
PBLD	0.308465411	0.76786889	2.489319273
C1QTNF9B	0.540677167	1.34571902	2.488951085
TUBB4A	0.123789091	0.30785651	2.486943764
ZNF367	1.739032404	4.32469475	2.486839657
TOR4A	5.754706102	14.309641	2.486598049
PPM1J	0.277099651	0.68801321	2.482908963
SLC6A13	0.357135791	0.88635084	2.481831451
PITRM1	18.26448943	45.3293108	2.481827425
CACNG4	1.710667108	4.24462241	2.48126733
NOTCH3	0.498664018	1.23700678	2.480641734
RASA4B	0.312542127	0.77530211	2.480632351
C14orf28	0.496932362	1.23172361	2.478654448
C2CD3	0.907072145	2.24817808	2.478499741
RAB22A	1.72809668	4.27565969	2.474201667
LOC10537675	0.1	0.24734717	2.473471689
ZNF34	0.545712525	1.34945351	2.472828544
NPFFR2	0.395280935	0.97720299	2.472173342
TMEFF1	0.368627258	0.91082432	2.470854517
HCFC2	0.327803978	0.80992032	2.470745848

CD164	28.58208546	70.6164157	2.470653018
TESMIN	0.20048512	0.49509365	2.46947828
DCDC5	0.112756831	0.27833992	2.468497205
ECT2	5.294213247	13.0685333	2.468456151
INVS	1.749263762	4.31793324	2.468428907
FBXO34	1.432557681	3.53588105	2.468229443
FANCF	1.054036728	2.60076066	2.467428873
MYLK2	0.186945593	0.46085021	2.465156854
ADSS	5.006727435	12.3316931	2.463024661
IFT52	4.041481278	9.95326064	2.462775392
SGMS1	1.814690803	4.46741249	2.461803675
GFM2	3.652070914	8.98546753	2.460375974
EXOC1	2.07012857	5.09059678	2.45907276
GPAT3	0.434418171	1.06649753	2.455002124
DENR	10.80561532	26.5250529	2.454747102
GALK2	0.714720132	1.75429871	2.454525394
OTUD3	0.701170569	1.72020234	2.453329361
SRGAP1	0.496011482	1.21668125	2.452929613
RSRP1	2.137746801	5.24359585	2.452861046
ME1	11.19303093	27.4440168	2.451884302
ZNF227	1.528683233	3.74765302	2.451556307
TBCK	0.549618665	1.34730838	2.451351212
HSPA13	1.257246496	3.08020459	2.449960769
HCLS1	0.338311129	0.82878286	2.449765275
NAAA	4.991418616	12.2263198	2.44946793
SLC35B3	4.528118032	11.0890336	2.448927677
AGO4	0.590080206	1.44471768	2.448341208
YEATS4	2.219754198	5.43413215	2.448078329
TGFBR2	8.536817613	20.8985711	2.448051729
LOC10050554	0.314413886	0.76908402	2.446087947
C1orf228	0.786761994	1.92417129	2.445684084
GPATCH11	0.830506588	2.02841456	2.442382266
STX2	2.594921654	6.337024	2.442086832
ZNF678	0.213185291	0.52030514	2.440624007
NDUFS2	17.40806856	42.4808252	2.440295147
ZNF33B	0.439793434	1.07315108	2.440125284
VPS37A	1.313689501	3.20404823	2.438969201
SEMA3A	0.570746338	1.39166801	2.438330165
MORF4L2	26.57470862	64.7502003	2.436534724
SLC2A1	4.847602503	11.8074753	2.435735051
DAB2	7.568316517	18.4311279	2.435300884
PPARG	7.981559281	19.4289594	2.434231044
ENPEP	0.355236229	0.86461234	2.433908101
MGLL	8.563185997	20.8360667	2.433214307
KRCC1	2.584359897	6.28774941	2.433000689
ENOX2	0.807519762	1.96455102	2.432820978

AGTPBP1	1.496528473	3.63724607	2.430455643
WNK1	1.735771189	4.21624126	2.429030557
AKR1C3	1.290387456	3.13361818	2.428431996
JMJD1C	0.556048734	1.34975086	2.427396692
MAP4K5	1.053872526	2.55813057	2.42736243
KMT2E	0.63856133	1.54964821	2.426780541
CEP128	0.142435831	0.34564299	2.42665757
PSMD10	6.001266402	14.5576218	2.425758309
STAM2	0.781533881	1.89576939	2.425703398
COL28A1	0.401362446	0.97255224	2.42312716
JUN	12.35887725	29.9188457	2.420838484
S100A11	495.0787354	1197.71993	2.41925141
CENPQ	0.948375632	2.29414678	2.419027543
F2RL1	3.616659715	8.74763822	2.41870646
PLAC8	3.678591969	8.89178253	2.417170104
NIPBL	0.527424726	1.27437781	2.416226895
CXCL8	18.50876601	44.7137845	2.415816616
ATXN7L3B	3.727416418	9.00325859	2.415415286
FAM220A	2.814664076	6.7845301	2.410422672
TRNP1	30.89218341	74.4631077	2.410419061
SP110	0.928436285	2.23746832	2.40993201
ATL1	0.143117727	0.34488095	2.409770994
ANKRD1	45.98599912	110.785044	2.409103764
PP2D1	0.527533523	1.2704761	2.408332452
INTU	0.469109718	1.12895591	2.406592462
SBF2	0.306238569	0.73670796	2.405666791
CDK8	1.378100215	3.31484732	2.405374649
ZFAND4	0.288500601	0.69292672	2.401820701
IVNS1ABP	1.66543517	3.9998049	2.401657518
RPS6KC1	1.265691711	3.03848823	2.400654289
LDHB	1.047356467	2.51427091	2.400587564
AP3S1	3.38108394	8.11527133	2.400198123
CCDC74B	0.600080397	1.43996108	2.399613588
ITGA6	4.069044739	9.7593975	2.398449298
MAT2A	5.714542031	13.7053494	2.398328562
SVEP1	0.129508957	0.31056417	2.39801303
CCDC102B	0.126595011	0.30332651	2.396038399
RMDN2	0.186941146	0.44726665	2.39255327
KCNAB3	0.148782451	0.35574117	2.391015652
CEP97	0.41541439	0.99239453	2.388926708
C6orf141	1.532623824	3.66017493	2.388175672
ATP2A2	8.102152401	19.3467477	2.38785285
KMT5A	3.184886521	7.60486942	2.387799179
HEATR4	0.130106501	0.31044376	2.386074168
GATA2	5.742950376	13.7008857	2.38568764
S100A14	0.1	0.23849864	2.384986444

PCDH9	0.228528114	0.54488503	2.38432383
IL9	0.305447581	0.72828234	2.384312038
MLYCD	2.810504102	6.69739397	2.382986727
SNAI1	1.982748562	4.72475257	2.382930776
ZNHIT6	1.87777707	4.47404708	2.38262952
DR1	2.597254083	6.18306418	2.380615827
PLPPR2	3.217289218	7.65301803	2.378716213
PCDHB2	1.456887215	3.46550707	2.37870649
SOX6	0.78965314	1.87820907	2.378524156
HAVCR1	1.655374023	3.93589737	2.377648386
E2F5	0.660182332	1.56837317	2.375666684
SERINC1	12.21540812	29.0080236	2.37470769
ZC3H8	2.072010773	4.92040622	2.374701081
BAZ2B	0.651906911	1.5466813	2.372549314
PTPRF	16.8832709	40.0403913	2.371601542
FYCO1	1.592811544	3.77736152	2.371505613
NIPAL2	0.524247102	1.2429445	2.370913436
CCZ1B	5.82047305	13.7946192	2.370016854
NAA20	28.51403101	67.5254616	2.368148563
ADHFE1	0.196110438	0.46389697	2.365488395
C14orf142	2.216581777	5.24211549	2.364954698
GAS8	6.283548313	14.8539578	2.36394424
BROX	2.872327948	6.78902864	2.363598017
CRIP2	11.38559271	26.8763581	2.360558538
P4HA3	0.179221088	0.42302294	2.360341308
USP16	3.541905049	8.3574826	2.359600973
CTTNBP2	0.27605537	0.65121657	2.359007075
EEPD1	4.672337393	11.0213583	2.358853268
IFNAR2	3.948089995	9.31044003	2.358213729
ZMYM6	0.925983132	2.18262121	2.357085279
CRIM1	2.786729815	6.56471596	2.355705934
ZNF354A	0.607606691	1.43055972	2.354417324
ZNF134	1.009417461	2.37642428	2.35425319
RGS19	2.770988459	6.52225041	2.353763108
SBK2	0.1	0.23531883	2.353188286
DOCK7	4.241991439	9.97484536	2.351453439
TCF3	7.747666521	18.2116095	2.350592841
ANKRD37	0.21799759	0.51240024	2.350485796
RNF144A	0.168753975	0.39661336	2.350245999
PPIP5K1	0.159511038	0.37484446	2.349959383
EPC2	0.596369109	1.4011033	2.349389468
PIK3C2G	0.190154084	0.44659625	2.348601942
FHL3	12.99670223	30.5120288	2.347674686
SCPEP1	3.311608477	7.77160772	2.346777337
CTNNA1	37.75343797	88.5456307	2.345366024
LRP2BP	0.195290787	0.45801823	2.345314096

GAB2	0.987858047	2.31523563	2.343692636
MTRF1	0.686810718	1.60887407	2.342529075
ARHGEF35	0.811634215	1.90104219	2.342240088
CRISP3	0.15442381	0.36153957	2.341216509
TBC1D10B	12.15701747	28.4455083	2.339842677
FAM129B	20.27096549	47.4289839	2.339749624
ZSWIM6	0.88328057	2.06641397	2.339476314
KPNA7	0.64570571	1.51055942	2.339392995
FAM3C	6.484435682	15.1636468	2.338468224
GCSAM	0.373830174	0.87407109	2.338150191
TRAF3	1.703559494	3.98040563	2.33652282
TNFRSF21	20.7866591	48.5570706	2.335972817
SLCO1A2	0.495142933	1.15648035	2.335649516
PIBF1	0.795652254	1.85776183	2.334891681
FAM162B	0.920402165	2.14806876	2.33383714
NFATC1	0.196537086	0.45848327	2.332807927
PRAME	1.662698342	3.87823911	2.332497132
IRS1	1.927686234	4.49612707	2.332395695
ZNF436	0.356729456	0.83198059	2.332245294
COG6	0.700515034	1.63373351	2.332189083
GABRG1	0.153812758	0.3586031	2.331426218
SLC35G2	0.950397876	2.21575422	2.331396436
ADGRE5	14.09523396	32.8505666	2.330615208
NIPAL3	1.068206832	2.4891623	2.330225031
GRIPAP1	12.21679607	28.4663652	2.330100711
CFAP36	7.281141716	16.9563654	2.328805845
CDK14	0.606013887	1.41078007	2.327966572
CCNDBP1	2.927577874	6.81418661	2.327585091
SMNDC1	4.736609647	11.0217223	2.326922227
GLDN	0.108759163	0.25306588	2.326846519
STK10	3.751259878	8.72785117	2.326645303
SVBP	15.78903916	36.721566	2.325763186
SPAG9	2.254641394	5.24367836	2.325726111
EPHA5	0.164830071	0.38322546	2.324972937
CELSR1	0.906915316	2.10843623	2.324843554
RPE	1.327067382	3.08519262	2.32481987
FOXJ3	1.880910381	4.3717464	2.324271504
DNM1	2.222718889	5.16530321	2.323867059
SUN3	4.134133905	9.60317246	2.322898261
HIST1H2AE	4.620939592	10.7278064	2.321563878
ADPRM	1.699730177	3.94383919	2.320273678
GALNT5	2.137278901	4.95747004	2.319524158
LSMEM1	0.971575766	2.2533111	2.31923354
UGT2B7	0.259594533	0.60200297	2.319012526
SCTR	0.153044788	0.35491253	2.319010907
CYR61	54.46102714	126.229177	2.317789128

SLC31A2	0.728765841	1.68898285	2.317593334
ALKBH8	0.488112556	1.13037951	2.315817325
ZCCHC3	8.043538762	18.614794	2.314254274
ATAD2B	0.337727891	0.78156179	2.314176042
COL4A3BP	1.450911513	3.3570701	2.313766254
LOXL2	2.968177649	6.86636763	2.31332772
ARHGAP42	0.426993034	0.98773941	2.313244791
PPP1R18	1.112049348	2.57212602	2.312960322
KLHDC8B	3.618351413	8.36840229	2.312766598
STXBP5	0.936414607	2.16510571	2.312122961
ATF7IP	0.975590127	2.25240101	2.308757485
FAM122B	1.329389911	3.0690706	2.308630878
PLIN3	34.86541825	80.4835479	2.308406205
KCNAB2	1.046477331	2.41554927	2.308267176
FGF22	0.785432777	1.81112841	2.305898693
AFAP1	0.53463904	1.23251237	2.305316825
HTATIP2	48.65342199	112.097815	2.304006797
EFEMP1	4.338302589	9.99235744	2.303287343
RAD50	1.820892097	4.19275356	2.302582106
DNM1L	8.370302533	19.2709621	2.302301745
BHLHA15	0.271982324	0.62594174	2.301405944
TANC1	0.791280165	1.81805606	2.297613589
AGAP5	0.500394132	1.14952971	2.297248585
TPM4	23.7784283	54.6210767	2.29708524
DYX1C1	0.49599944	1.13801016	2.294377918
UHRF2	0.700833593	1.60791165	2.294284498
CLIP4	0.466826346	1.07044359	2.29302309
TCTN2	1.6010655	3.67033525	2.292432912
PAPSS2	3.45178303	7.90962911	2.291461845
ANKRD52	13.53105255	31.0040941	2.291329071
PLSCR1	1.542925639	3.53204761	2.28918849
MXD1	1.937397052	4.43463525	2.288965623
TNFAIP8	2.205960582	5.04917285	2.288877187
IL17RD	0.255926799	0.58572735	2.288651884
MOSPD2	1.82159183	4.16639616	2.287228178
EYA4	0.609253106	1.39334591	2.286973839
LOC10272442	1.789226044	4.09179135	2.286905761
PPM1N	0.549701928	1.25694814	2.286599479
ESYT2	4.788012373	10.9458598	2.286096801
MAP3K2	0.686432827	1.56886285	2.285530053
TFRC	16.6022702	37.9341498	2.284877269
TSGA10	0.323384299	0.73877548	2.284512531
YWHAG	12.78634701	29.2060797	2.284161352
WDR1	101.2101402	230.995134	2.282331921
ANKIB1	1.55782129	3.55401908	2.281403588
EID3	0.264748332	0.60381936	2.280729599

KDM3A	1.299602107	2.96349968	2.280313076
LATS1	0.826000322	1.88320225	2.279904986
POP1	2.569332713	5.85725292	2.279678647
SVIL	0.609498805	1.38926765	2.27936075
FAM13A	0.92201989	2.10130268	2.279020988
C7orf25	1.789641568	4.07786095	2.278590877
PTGER4	0.970836725	2.21186106	2.278303866
MKRN3	0.301321976	0.68634835	2.277790557
CCDC88A	0.910453336	2.0730376	2.276928998
UBE2A	15.68152526	35.6892843	2.275880928
SNAPC1	1.448597837	3.29548077	2.274945253
CCDC17	0.370164222	0.84188784	2.274363088
TMEM243	5.763340012	13.0961562	2.272320602
POLK	0.342612603	0.77834263	2.271786353
GOLGA6L9	0.268746394	0.61037506	2.271193476
TMEM2	15.86610974	36.0271386	2.270697683
RIT1	2.392265127	5.43068933	2.270103455
LOC10192724	0.354637233	0.80454711	2.268648162
TCEANC	0.321703324	0.72956611	2.267822742
CTNNB1	24.47502569	55.4908457	2.267243614
MAP10	0.155645976	0.35282667	2.2668538
MTURN	0.841381661	1.90648541	2.265898466
SNX25	0.644691517	1.45977905	2.264306282
LRRC37B	0.68429139	1.54922642	2.263986434
LGALS3	23.31620688	52.7381942	2.261868512
FPGT	0.575701213	1.30199342	2.261578392
ZNF823	0.957024467	2.16432203	2.261511701
MCIDAS	0.1	0.22605746	2.260574639
EML6	0.283824552	0.64133602	2.259621349
MED11	8.611883909	19.4586837	2.259515331
PLEKHA7	0.620438535	1.40074415	2.257667873
CGA	0.1	0.22569901	2.256990067
GLRB	0.771111295	1.73886137	2.255007001
COMMD10	0.646372231	1.45715495	2.25435883
SKIL	2.591725231	5.83774123	2.252453757
DEPDC7	0.800263032	1.80254703	2.252443204
MAK16	2.410164351	5.42824514	2.252230281
RBMS2	2.925872648	6.58216417	2.249641374
FAM72B	0.230934062	0.51947864	2.249467382
FAM127B	25.10663934	56.4708169	2.249238385
ZNF251	1.715158237	3.85742631	2.24902066
HTATSF1	7.81041214	17.5378144	2.24544033
HIBADH	7.793495257	17.4939409	2.244684878
PLAGL1	1.994170203	4.47600053	2.244542881
STK36	1.711545814	3.84055792	2.243911841
ZNF184	0.387809234	0.87013454	2.243717943

WDR41	2.142931614	4.80388296	2.241734138
CTBS	0.922254089	2.06716311	2.241424713
MAGI3	0.722334057	1.61878524	2.241047927
GSG2	0.924801891	2.07235538	2.240864128
KLF10	2.262214365	5.06736848	2.240003671
EFL1	3.609324057	8.08308726	2.239501671
E2F3	0.716450647	1.60442698	2.239410327
PLA2R1	0.131949142	0.29540831	2.238804301
FAM229B	2.963882362	6.63380707	2.238215375
TEX9	0.291755402	0.65290094	2.23783667
INIP	1.693432027	3.78948029	2.237751638
CLIP3	0.66212741	1.48137971	2.237303104
HIST1H4E	0.422067694	0.9442172	2.23712266
DST	1.961401579	4.38757135	2.236957182
TTC14	1.342857009	3.0032348	2.236451667
CDRT15	0.421215663	0.94198382	2.236345668
GSK3B	3.203944878	7.1587942	2.234368714
STXBP6	2.165448684	4.83629973	2.233393829
ITPRIPL2	0.754488908	1.68466627	2.232857567
FNBP1L	2.070058875	4.62198924	2.232781541
SEC16B	0.688389823	1.5368546	2.232535327
TOB1	2.053129016	4.57966996	2.230580702
ADAM10	5.828473602	12.9977069	2.230036161
HIST1H3H	2.410308	5.37085433	2.228285485
ZFAND5	13.07376267	29.1174486	2.227166681
MRFAP1	143.2015777	318.904221	2.226960247
RNF146	1.283732713	2.85567735	2.224510853
WDFY3	0.557866907	1.2409116	2.224386468
PKD2	0.566170634	1.25928192	2.224209178
VMP1	13.82665535	30.7422522	2.223404827
OSBPL3	3.638868884	8.08478982	2.221786517
ZNF443	0.186011467	0.41313448	2.221016192
MKNK2	17.51896952	38.8922079	2.220005456
ARL6IP1	30.11466353	66.8411112	2.219553645
HIST1H2BE	0.1	0.22194248	2.219424846
TOP1	5.010109157	11.1096037	2.217437451
HAUS3	3.665295981	8.11665419	2.214460777
TMEM44	1.434979021	3.17716433	2.214084168
RFX2	1.251595646	2.7703091	2.213421811
ITFG1	3.760441426	8.32197203	2.213030622
PDGFRA	0.645484663	1.4282732	2.212714384
VCL	6.712829986	14.844379	2.211344401
SLC35E2	0.620865436	1.37260758	2.21079722
ANKS1B	0.88590658	1.95777203	2.209907993
BPTF	0.832250569	1.8391797	2.209886982
SMURF1	4.376967318	9.67258026	2.209881765

BMPER	0.112067136	0.24761076	2.209485928
PITX1	11.23309493	24.8083209	2.208502737
PLS1	1.404339561	3.10111255	2.208235552
TMEM116	2.362960214	5.2135907	2.206381074
ZNF396	0.193519528	0.42684969	2.205718946
FGD6	0.485645548	1.0708922	2.205090115
VEZT	3.573263994	7.87655857	2.204303567
SYBU	0.536760865	1.18297161	2.203908083
HMX3	0.269580336	0.59390112	2.203058035
ZNF84	0.98571972	2.17109068	2.202543618
ZNF559-ZNF1	0.164084898	0.36130532	2.201941362
MADCAM1	0.139498868	0.30707214	2.201251866
ZNF528	0.61673824	1.3568744	2.200081516
PKM	659.0258957	1449.24887	2.199077271
IRAK2	1.395048215	3.06648206	2.198119055
ZNF595	1.270474538	2.79210422	2.197686086
ZBTB3	0.508270076	1.11666574	2.196992883
CLCN3	1.73322978	3.80690012	2.196419752
SBDS	15.79753925	34.6701246	2.194653485
MRFAP1L1	21.68399767	47.580589	2.194272002
LOC10798597	0.168715065	0.37017817	2.194102642
DENND6A	0.658336238	1.44428318	2.193838197
PEX6	4.872779893	10.6847332	2.192738724
GLIPR1L1	0.180709599	0.39618309	2.192374348
ZNF547	0.248382794	0.54451101	2.19222517
PLCD3	8.675319261	19.0151459	2.191866993
MEPE	0.265121741	0.58091551	2.191127395
TEX2	0.886930594	1.94293548	2.190628548
SLC25A12	0.16074013	0.3520897	2.190428134
NR0B2	1.150837707	2.52062068	2.190248606
SLC5A12	0.755511833	1.6546115	2.190053718
ZNF177	0.17442962	0.38180823	2.188895638
ZHX2	2.156727629	4.71950112	2.188269421
CDKN2A	34.25070006	74.9222007	2.187464799
TFPI2	0.373243602	0.81623747	2.186875977
FAM127A	68.81329776	150.450967	2.186364721
PRKD3	1.674831653	3.66041214	2.185540337
TMF1	0.40564465	0.88590809	2.183951118
DAPK1	2.280294767	4.97999906	2.183927765
CCDC186	0.385892978	0.84264323	2.183618987
CLDN2	0.754864165	1.64826721	2.18352822
RABL2B	2.877287102	6.28036589	2.182738692
ZNF407	0.400620344	0.87441234	2.182645866
LRRFIP2	6.706866289	14.634453	2.182010554
ZBTB5	2.924997967	6.38215193	2.181933799
ZNF493	0.271531109	0.59244584	2.181870943

CDS2	3.070662122	6.69900238	2.181614947
CASC4	2.344692135	5.11330605	2.180800617
CAMK2N1	9.938544725	21.6699709	2.180396781
CTSC	11.07532976	24.1431485	2.179903355
ZNF425	0.552495107	1.20373097	2.178717877
LONRF3	0.616868886	1.34332885	2.177657012
PIK3C2B	1.534533969	3.34160341	2.177601462
SCRN3	1.346485105	2.93169334	2.177293558
TM4SF1	121.4128249	264.330505	2.177121775
MBIP	2.356990009	5.13012749	2.176558862
MYEOV	0.173026977	0.37649749	2.175946784
ELOVL1	67.5303979	146.903452	2.17536779
SUCO	5.005217274	10.8865301	2.175036463
IBTK	3.115758749	6.77371857	2.174018952
PLPP2	0.275842897	0.59933726	2.172748555
CFAP126	1.418091857	3.08081629	2.172508273
PHF7	0.50968206	1.10675839	2.171468205
TMX3	1.750486632	3.80029266	2.170992103
KIF9	2.18937282	4.75229363	2.17061872
BBS4	3.361178867	7.28966122	2.168781106
RNF103	3.171056748	6.87653799	2.168531987
FSD1L	0.455962457	0.98863196	2.168231064
PPP1R11	5.31724502	11.5282606	2.168089029
DSCR3	4.67517418	10.1346549	2.167759863
SH3BP5	0.392401013	0.85048588	2.16738962
JOSD2	16.44727278	35.6226148	2.165867574
POU6F2	0.563659202	1.22054721	2.165399236
DPCD	29.88501378	64.6967038	2.164854407
LRP11	4.915837807	10.6415537	2.164748736
KIAA0430	1.328277366	2.87529152	2.164677045
C6orf62	15.66767249	33.9054917	2.164041388
CAPN2	27.93110855	60.429541	2.163521038
FAM46C	0.333499078	0.72133107	2.162917732
FAM219A	6.782486589	14.6694677	2.162845078
ARPP21	0.187003444	0.40407605	2.160794694
PODN	4.462274734	9.6363456	2.159514188
IFT81	0.809618852	1.74796772	2.159000765
DNAJB4	0.943463551	2.03669881	2.158746687
SMIM10	3.182917512	6.86876057	2.158007723
HSPA4L	0.518584902	1.11883982	2.157486295
NES	44.19595215	95.3290066	2.15696239
44258	1.567891101	3.37901227	2.155131993
MTAP	2.381858115	5.13291468	2.155004383
PRDM10	0.671244494	1.44631931	2.154683313
SLC4A4	0.376204551	0.81020837	2.153637889
P4HTM	1.934854777	4.16608482	2.153177008

NRP1	10.34219084	22.2652241	2.15285373
SPEF1	0.559053007	1.20301472	2.151879532
GNG12	3.636981454	7.82606878	2.151803325
UBE2D1	4.124451149	8.87090267	2.150808034
MAGEB2	9.691554129	20.8427709	2.150611829
TTYH3	4.619868089	9.93455264	2.150397469
TATDN3	1.3798148	2.96674676	2.150105043
SYCP2L	0.487921791	1.04904758	2.150032239
PTTG1IP	73.16224153	157.264057	2.149524857
CADM4	23.17305685	49.8052922	2.149275883
CENPF	2.776984377	5.96812676	2.149139481
KIAA1841	3.043938699	6.54110155	2.148893983
BRPF1	2.266437203	4.87015427	2.148815006
RALB	9.784652228	21.023298	2.148599408
TNFRSF10B	7.115090253	15.2830723	2.147980099
LIPA	4.230812495	9.0848176	2.147298565
ERMN	0.195380613	0.4194644	2.146909037
ERC1	1.141821158	2.45019884	2.145869184
FRYL	0.454127437	0.97444173	2.145745121
PLCG2	0.225505419	0.48381001	2.145447395
TRIM36	0.565208891	1.21182854	2.144036579
TAF1B	1.485112911	3.18268291	2.143057872
RLF	0.618804724	1.32560013	2.14219458
MESDC2	8.579732154	18.3742259	2.141585022
PXMP2	24.14284131	51.7038424	2.141580674
HOXC6	0.976464296	2.0911013	2.141503084
FAM32A	33.555348	71.8323869	2.140713514
POLR3G	1.86131296	3.98201637	2.139358858
CSRNP2	3.482123249	7.44817344	2.138974673
GPATCH8	1.047266334	2.23983305	2.138742528
ZEB1	0.494528414	1.05688985	2.137167088
KIF16B	0.657884242	1.40566904	2.136651022
HECW1	0.437878535	0.93552947	2.136504515
ADGRV1	0.473982096	1.01222118	2.135568386
TBX19	0.487234474	1.03984729	2.134182498
ZNF202	1.272419343	2.7139813	2.132929927
IKZF5	0.64331692	1.36936433	2.128599892
KDM2A	27.66617975	58.8879266	2.128516734
AMN1	0.527692045	1.12274342	2.12764894
UBR3	1.436354628	3.05537264	2.127171509
DUSP4	3.959772016	8.42090847	2.126614471
MAX	7.413766405	15.7636447	2.126266709
MET	3.928952148	8.35285085	2.125974187
CHST11	0.215777975	0.45866058	2.125613532
FOXP4	13.81556863	29.3643515	2.125453703
NOMO2	1.884321662	4.00385116	2.124823613

ABHD17C	0.1	0.21238343	2.123834339
BSCL2	20.14404072	42.7731982	2.12336734
ADAM23	2.21264588	4.69801479	2.123256519
ZFYVE16	0.181819084	0.38595677	2.122751703
TRIM5	2.738112019	5.8118673	2.122582004
PDZD11	14.02301277	29.7625321	2.122406402
APBB2	1.139157839	2.41706985	2.12180417
L3MBTL3	0.727722779	1.54393385	2.121596157
HCST	0.18159914	0.38518807	2.121089718
CYB5R1	15.43447991	32.7082962	2.119170608
PRDM8	0.144013843	0.30510384	2.118573015
MICU2	7.850809195	16.631494	2.118443285
BIRC7	0.321892399	0.68163356	2.117582039
AP2A1	18.81059747	39.8159939	2.116678854
FAIM	5.206695057	11.0180929	2.116139458
NOX1	0.196021764	0.41462029	2.115174771
POLN	3.344317654	7.07274318	2.114853884
APIP	7.93198627	16.7724647	2.11453526
ZMYM5	0.863644783	1.82610137	2.114412555
YWHAH	51.76932187	109.385086	2.112932563
ZNF200	0.65636977	1.38680546	2.112841766
UBR1	0.640652227	1.35286932	2.111706262
ENC1	5.062824068	10.6904652	2.111561655
TBC1D19	0.641246005	1.35321723	2.110293426
MEIS2	3.383816417	7.13745305	2.109290863
DDX43	1.541181252	3.24807008	2.107519848
ARMCX6	2.075575875	4.37428564	2.107504571
MARCKS	25.32288924	53.3667202	2.107449891
GPD2	3.41290614	7.19143383	2.107129095
CD2AP	2.164042107	4.55962052	2.106992516
RUFY2	0.478344623	1.00696915	2.105112304
NPAS2	1.588034974	3.34138691	2.104101589
STK38L	1.600476616	3.36718791	2.103865735
RCHY1	1.757326191	3.69647106	2.103463252
ANXA6	9.413429127	19.7968495	2.103043347
LOC10798554	0.200089365	0.42066026	2.102361922
FAM13B	0.918678028	1.93009829	2.100951834
KIFAP3	1.800346426	3.78188493	2.100642897
ROPN1L	0.235412105	0.49438647	2.100089438
ZNF25	0.226163259	0.47479829	2.099360821
DOCK1	1.160164669	2.43492844	2.098778307
NIPAL1	0.214503509	0.45016491	2.098636561
RABL2A	1.217929089	2.55553492	2.098262486
SCLT1	0.668529772	1.40229388	2.097578812
NOTCH2NL	0.442091061	0.92695969	2.096761902
COQ9	10.66759592	22.3669576	2.096719615

LCOR	0.640426089	1.34262043	2.096448678
BCKDHB	0.840579685	1.7616999	2.095815458
PPP1R21	1.970556981	4.12934723	2.095522875
ZNF426	0.586348908	1.22830484	2.094836068
PDE8A	5.201550882	10.8959589	2.094751962
NINL	3.293015785	6.89664323	2.094324375
C1QTNF3	0.544295161	1.13982581	2.094131814
DUSP6	6.233563405	13.0526625	2.093932736
CLIP1	3.152558239	6.6012125	2.09392246
SLC37A3	2.227938235	4.66494865	2.093841103
NCDN	6.523272878	13.6583257	2.093784207
PWWP2A	0.657174745	1.37549422	2.093041806
LZTFL1	1.161652503	2.43124513	2.092919461
HYI	41.43878472	86.7196941	2.092718083
LTBR	35.08573925	73.4227579	2.092666691
CHMP1B	7.480402225	15.6494601	2.092061308
WSB1	8.220053998	17.1893925	2.091153236
BOD1L1	1.031910916	2.15784324	2.091113881
KCTD18	0.876496428	1.83186238	2.089982712
GTF3C3	3.450274191	7.21088154	2.089944492
FNBP1	5.133723839	10.728988	2.089903611
ZSCAN32	1.079493494	2.25603242	2.089899042
RFX3	0.184530438	0.38558516	2.089547726
TRMT13	0.375562032	0.78469408	2.08938607
SNX3	35.31855977	73.7903285	2.089279091
GNA12	6.591764147	13.7663995	2.088424164
ZNF830	3.067707048	6.4064944	2.088365773
SYNE2	0.231828477	0.48394919	2.087531262
MTHFD2L	0.475679209	0.99283631	2.087197193
MYH11	0.274905676	0.57375235	2.087088042
FAM127C	4.698771803	9.8062139	2.086973855
GANAB	39.11916677	81.6253322	2.086581566
PRSS3	0.736259496	1.53615232	2.086427853
PTMA	157.2298205	328.043024	2.086391903
PPP1R14B	138.9266756	289.670777	2.085062321
SAMHD1	1.587761066	3.31002385	2.084711558
ZSCAN18	0.369452674	0.77007999	2.084380608
HERC2	1.21473278	2.53155695	2.084044323
GOLGA8A	0.701628255	1.46196572	2.08367567
CSTF2T	1.621436814	3.37820563	2.083464245
ZNF37A	0.572647552	1.19279395	2.08294604
CLGN	5.607334734	11.6789186	2.082793184
NCAM2	0.198360912	0.4130765	2.082449075
AKAP9	1.013187822	2.1097928	2.082331386
DOCK9	0.71988769	1.49862687	2.081750931
C19orf68	1.471886809	3.06377469	2.081528737

DBN1	120.7805594	251.392885	2.08140189
TOP2B	7.528971174	15.6694236	2.081217107
SRGAP3	0.262926434	0.54670491	2.079307513
MYCBP	3.105902359	6.44885345	2.076322019
IER5L	41.27510676	85.6961997	2.07621994
KANSL2	4.169359714	8.65564207	2.076012304
TBC1D9	5.843413336	12.128428	2.075572499
TMX4	3.369896236	6.99309371	2.075165887
TAF1	0.274513511	0.56964051	2.07509098
HERC3	0.296773361	0.6157905	2.074952066
NEURL1B	0.326811059	0.67801193	2.074629709
RGMB	0.477235461	0.98994272	2.074327662
NR1D2	1.163693842	2.41361147	2.074094902
FANCM	0.364251975	0.75542801	2.073916039
PALMD	2.360720319	4.89421277	2.073186194
STRIP2	0.736622106	1.52689386	2.072831984
CPSF6	6.09880989	12.6400542	2.072544379
PLXNA2	1.866631161	3.86817306	2.072274987
DHX57	1.594510026	3.30420654	2.072239423
HBEGF	2.056718311	4.26171393	2.072094126
RGL4	0.562951378	1.16641556	2.071965013
EPCAM	6.920130224	14.3338394	2.071325102
FRK	0.981056214	2.03181338	2.071046845
HIST1H4C	1.033996311	2.14019157	2.06982515
COL26A1	0.29367963	0.60768108	2.069197247
PCYOX1L	1.021340535	2.1125467	2.068405812
TAF15	29.58205609	61.1828328	2.068241389
SH3YL1	0.207896493	0.42995989	2.068144035
GNMT	0.382108551	0.79017633	2.067936788
HKR1	1.333549166	2.75735935	2.067684805
CCDC71L	0.141168463	0.29184442	2.067348534
AFTPH	1.984299057	4.10023982	2.066341669
GVQW2	0.256699234	0.53030589	2.065864724
MIB1	1.419915347	2.93327952	2.065812957
HIST1H2AG	0.1	0.20651603	2.065160257
ARMCX1	1.092452876	2.25549432	2.064614745
SNX13	0.845145636	1.74480561	2.064502889
FAM188A	1.403618886	2.89762566	2.064396318
ABAT	0.393441376	0.81220436	2.064359294
CRYAB	1.721629656	3.55367989	2.06413724
MTCP1	0.414016001	0.85451912	2.063976081
CEP55	6.024577205	12.4318588	2.063523853
FRZB	0.187667524	0.38724719	2.063474706
SPATA17	0.206836358	0.42671788	2.063069958
MYCBP2	0.734445761	1.51512963	2.062956466
SPIRE1	3.57092137	7.3620561	2.061668497

EIF1B	37.47369627	77.2563357	2.061615037
SPINK5	1.182113682	2.43592576	2.060652709
CNBD1	0.224405246	0.4623291	2.060241957
MIS18BP1	0.817341694	1.68343226	2.059643201
CEP41	1.653780481	3.40406603	2.058354217
MIA3	2.389008731	4.91532247	2.05747363
NIPA1	1.690567976	3.47742604	2.056957244
SIRT5	1.424430644	2.92875394	2.056087425
FAM214B	6.971765798	14.3339233	2.055996104
INSIG2	1.38340754	2.84336065	2.055331182
HIST1H2AC	29.12059967	59.8484674	2.055193508
CCDC96	1.199712331	2.46521977	2.054842401
CTHRC1	1.523997504	3.12758686	2.052225714
ARHGAP26	0.470255486	0.9644603	2.050928321
TPBG	0.240250486	0.49213909	2.048441582
44445	1.040826861	2.13202086	2.048391464
FAM83D	3.824065161	7.83072178	2.047747999
RSU1	10.07547858	20.6257942	2.047127986
PODXL	0.3130523	0.64073259	2.046726989
TPR	3.24555103	6.64046581	2.046021074
DCLRE1B	0.500671596	1.02422617	2.045704567
C10orf131	0.203247892	0.41574393	2.045501827
ATP8	924.5307452	1891.01263	2.045375597
WDR3	1.728531097	3.53417338	2.044610816
ASH1L	0.827032121	1.69035827	2.043884661
ZNF737	0.302582921	0.61837545	2.043656172
ATR	0.818845385	1.67339496	2.043603089
MYO6	2.80658244	5.73180079	2.042270595
ANKRD36	0.150155591	0.30659558	2.041852589
ABI1	5.413122641	11.0507625	2.041476467
C16orf45	0.43484382	0.88768533	2.041388867
ZNF107	0.507130332	1.03482265	2.040545762
DDX59	0.431546021	0.88054659	2.040446558
LOC285500	0.501351929	1.02284239	2.040168462
MTMR3	1.797536892	3.66716598	2.0401061
MFN1	2.612530982	5.32791726	2.039369982
FOPNL	4.735102026	9.65249862	2.038498551
YWHAQ	48.55468441	98.9651129	2.038219671
DCAF17	0.932781348	1.90090984	2.037894355
IER5	5.984336282	12.1947992	2.037786421
COMMD3-BM	3.952596945	8.05361578	2.037550474
KIAA1109	0.249393443	0.5079128	2.03659245
ZNF75A	2.912044478	5.93043229	2.036518446
ACAP2	1.306317215	2.65975374	2.036070342
SZRD1	12.86742701	26.1858482	2.035049294
KLRF1	0.144346063	0.29373623	2.034944547

AP2B1	21.68374013	44.1207664	2.034739676
CREM	2.883869076	5.86525034	2.033812973
NRIP1	1.081789509	2.19977718	2.033461372
POLR3D	8.975143798	18.2491381	2.03329757
FLNB	22.21994179	45.1711153	2.032908804
FASTKD5	4.743883162	9.64179711	2.032469346
ZIK1	0.257373257	0.52309057	2.032420078
C1GALT1C1	5.520514307	11.2160438	2.031702691
REEP3	1.448006491	2.94188836	2.031681748
ARMCX3	1.187386724	2.41237833	2.03167029
ZNF222	1.374373976	2.79091156	2.030678413
TRIB1	5.34820299	10.8570041	2.030028423
ZNF621	1.066146131	2.16352041	2.029290686
RAB3GAP1	3.357357048	6.81251529	2.029130411
AGO2	0.838889194	1.70193851	2.028800136
KMT2C	0.540672614	1.09674758	2.028487383
TMTC3	1.726681067	3.50230198	2.028343302
JAG2	0.932353182	1.8908931	2.028086709
SSUH2	0.781734219	1.58529041	2.027914819
SDAD1	4.743193899	9.61699305	2.027535297
INTS12	3.208784558	6.50426496	2.027018282
RBM23	0.361593266	0.73295304	2.027009641
CDR2L	5.42240545	10.9910226	2.02696437
MBD1	5.046913057	10.2269748	2.026382202
NBN	1.706418296	3.4574194	2.026126544
PDGFC	2.275977069	4.61115673	2.026011944
CUZD1	0.233193857	0.47240882	2.025820156
EML1	0.811905983	1.64354559	2.024305303
NEU4	5.250985971	10.6294496	2.024276904
ZNF721	1.044546568	2.11236287	2.022277354
ZNF284	0.509095419	1.0293595	2.021938252
PTPN21	0.950609218	1.92059454	2.020382829
KLHDC10	2.928972984	5.91698609	2.020157278
EIF4G3	2.437748859	4.9245494	2.020121713
TRDMT1	0.386810708	0.78131886	2.019899785
ABCC5	5.881881927	11.8806381	2.019870206
TTC37	1.228196807	2.4807004	2.01979063
DDX5	31.97223901	64.5184923	2.017953522
RNF44	5.830369856	11.7614658	2.017276107
PTPRM	3.369558691	6.79670854	2.017091603
CACNB3	1.436212504	2.89588373	2.016333737
ELF5	0.124769384	0.25149525	2.015680812
BOC	0.505069293	1.01800113	2.015567255
SLC25A24	1.875139188	3.77813986	2.014858353
MEMO1	5.367799228	10.8112789	2.014098977
FHDC1	0.825043658	1.66166309	2.014030503

AHDC1	1.401562594	2.82226017	2.013652607
PPP2R2C	0.168397657	0.33904903	2.013383328
FBXO36	0.637254515	1.28295001	2.01324586
RSPRY1	2.444168777	4.92004103	2.012971067
LRRC8A	12.08939877	24.3274103	2.012292816
HNRNPH1	28.41363533	57.1730189	2.012168391
JAK2	0.201445942	0.40530902	2.011998956
KLHL9	1.173291319	2.3603029	2.011693824
FBXO11	2.247716257	4.52148616	2.011591165
MECOM	0.882972887	1.77609483	2.011494189
PAIP1	12.75872325	25.6549317	2.010775783
CREBRF	0.374291085	0.75241066	2.010228635
SGPL1	2.651006698	5.32836574	2.009940504
WDR37	1.429795225	2.87246592	2.009005117
BAHD1	2.342304878	4.70526367	2.008817774
RAB6B	2.458084555	4.93614553	2.008126822
TOMM34	17.53702229	35.2122296	2.007879618
SMURF2	0.590352076	1.18478308	2.006909306
BMI1	3.002740723	6.02309844	2.005866972
SUPT3H	0.886893652	1.77889163	2.005755287
TNRC6B	0.5649544	1.1330423	2.005546458
ARHGAP32	0.301621141	0.6046915	2.004804754
SENP7	0.450853537	0.9033915	2.003736082
PPP1CB	6.285430846	12.5899333	2.003034258
COG5	3.518004503	7.04658944	2.00300751
SESTD1	1.523611338	3.04976396	2.001667935
CPE	0.1	0.20016046	2.001604623
ITGA5	18.7843215	37.5934406	2.00132012
RLIM	0.55122505	1.10310858	2.001194583
LRRC63	0.320533639	0.64136894	2.000941141
SAA4	34.06094858	0.73813511	0.021671009
SAA2-SAA4	18.09865833	0.53684102	0.029661923
CPA2	8.270712092	0.33930067	0.041024359
HPR	7.480977291	0.31354662	0.041912522
ESPN	5.314812738	0.25868157	0.048671812
RBP2	2.041300473	0.1	0.048988378
OXT	7.57197049	0.42643065	0.056316999
LOC10537009	1.808209348	0.10746272	0.059430461
HP	11.08477706	0.72276566	0.065203446
IHH	22.13365815	1.45684555	0.065820369
APOA1	4639.077751	305.919229	0.065943975
KISS1	2.565802587	0.17429269	0.067929112
CDKN1C	45.71095976	3.12328906	0.068326919
F12	52.75853899	3.61736587	0.068564557
SHBG	2.493409805	0.22064817	0.088492543
PCOLCE	12.42932785	1.11505827	0.089711872

AQP10	2.002304751	0.18597242	0.092879176
RAB17	35.58114349	3.31427098	0.093146837
TFF3	9.143104703	0.85490959	0.093503205
AVP	1.596566183	0.14934417	0.093540857
SLC5A9	1.704827678	0.17608911	0.103288511
PPP1R1A	2.582678308	0.27353865	0.105912784
SLC19A3	13.93887908	1.48614976	0.106619029
PGLYRP2	0.917561219	0.1	0.108984554
ITIH1	2.737748734	0.29962189	0.109440975
MRGPRF	1.942974864	0.21366782	0.109969419
ACTN3	2.125625301	0.23746675	0.111716184
SMPD5	1.654927044	0.1886052	0.113965871
AGXT	0.85982178	0.1	0.116303172
FTCD	4.682205104	0.5517781	0.117845778
PDZK1IP1	0.846988101	0.1	0.118065413
SLC13A5	2.241177314	0.27740796	0.123777784
PLEKHF1	1.932975508	0.24079623	0.124572832
SLC38A3	10.9494521	1.37769573	0.125823257
FAM3B	0.784967604	0.1	0.127393792
CYGB	2.309152952	0.29546746	0.127954909
SCGN	54.41925916	6.97222424	0.128120528
APOD	1.872939901	0.24082738	0.128582547
SPATA25	0.775491074	0.1	0.128950549
TFR2	4.984911829	0.64805397	0.130003095
SARDH	14.36217108	1.88791204	0.131450324
SOAT2	9.863593051	1.3233621	0.134166332
KHK	5.539176484	0.76409992	0.137944678
CLDN3	0.719487349	0.1	0.13898785
C2orf54	0.869391021	0.12100791	0.139186983
SMIM1	11.0876897	1.55461556	0.140210955
ITGAL	2.80390946	0.40549999	0.144619501
GJB1	13.01340204	1.94631701	0.149562505
DUOXA2	0.668139467	0.1	0.14966935
CD7	16.95120133	2.54032599	0.149861119
FOXE3	1.172610605	0.18033061	0.153785589
KRT222	0.834815542	0.13057348	0.156409973
ANPEP	65.63331919	10.2875391	0.15674263
KRT4	0.637254515	0.1	0.156923172
KNG1	4.588789007	0.72131749	0.157191252
HIST1H1E	1.492070935	0.23514645	0.157597365
GCGR	0.630338135	0.1	0.15864501
HCAR3	3.253384832	0.52197692	0.160441185
APOC3	153.4120774	24.7813419	0.161534491
TMEM82	7.106818177	1.14831978	0.161580014
LOC1053710€	0.61888684	0.1	0.161580427
FAM19A4	0.761614649	0.12312336	0.161660962

MROH6	1.015034836	0.16526605	0.162818105
C12orf57	171.3957893	28.1898682	0.16447235
SLC30A10	6.475557304	1.07730265	0.166364469
ENTPD8	1.564911145	0.26786386	0.171168736
OCA2	1.501882537	0.25851529	0.172127504
CPN1	12.30418301	2.12392581	0.17261819
CHST13	159.0887891	27.8713072	0.175193408
SLC39A5	17.58782321	3.08145091	0.175203655
REEP6	324.1654281	58.3219855	0.179914267
CLDN19	0.555070385	0.1	0.180157333
HIST1H2BB	1.226863299	0.2230742	0.181824822
ASGR1	97.0176425	17.6993653	0.182434502
MMEL1	1.631624352	0.30073786	0.184318078
DDC	19.16345128	3.55945527	0.18574187
SULT2A1	22.12905744	4.13109819	0.186682067
FABP1	214.5013111	40.4886345	0.188757049
DUSP15	1.689552748	0.31929482	0.188981861
PCP2	0.839543037	0.15874306	0.189082692
TFF2	0.528741245	0.1	0.189128427
MYL4	1.930445032	0.36655769	0.189882481
LCN10	0.521386536	0.1	0.191796284
GCKR	7.199908004	1.38432343	0.192269599
RARRES1	0.661261266	0.12724447	0.19242692
BAIAP2L2	1.425759082	0.27484955	0.192774185
INPP5J	7.439202164	1.43759902	0.193246398
PLEKHG6	10.54781634	2.04769246	0.194134255
CA5A	0.710148344	0.13807967	0.194437784
RTN4R	20.85331043	4.08044671	0.19567381
PLK5	0.508291197	0.1	0.196737619
SECTM1	7.336220326	1.44820722	0.197405089
TM7SF2	157.9226247	31.410123	0.198895649
SLC47A1	6.968960368	1.39417459	0.200054888
MST1	8.20238818	1.65815397	0.202155023
NUDT8	24.20734327	4.90608887	0.202669447
SEZ6L2	1.58488871	0.32369264	0.204236825
SLC34A3	2.08608411	0.42830669	0.205316117
PNMA6A	7.02912101	1.45057458	0.206366426
CITED4	11.80931859	2.44203246	0.2067886
AFM	0.738410098	0.15312467	0.207370766
LBX2	5.744502501	1.19597068	0.208193952
C1QL1	3.702112441	0.77190309	0.208503416
C1orf54	0.478844058	0.1	0.208836256
CGREF1	6.485534367	1.36428452	0.210358074
MLXIPL	1.631829797	0.3461484	0.21212286
LCN12	3.62012493	0.77145545	0.213101887
TMEM52	9.225475442	1.98259848	0.214904749

IRX3	0.987063496	0.21342476	0.216221919
SEMA3F	3.493093449	0.76851209	0.220009026
PRAP1	6.038149006	1.32884125	0.220074273
DAB1	0.547087382	0.12056007	0.220367119
TMPRSS6	1.202431463	0.26607696	0.221282433
LYPD6B	1.33363429	0.29597899	0.22193415
ITPKA	57.82188128	12.8376806	0.222021151
EMILIN3	4.129338673	0.91778256	0.222258971
ATAD3C	0.571959784	0.12749157	0.222903029
CLIC3	5.245933259	1.17053443	0.22313178
44264	7.645501188	1.71711667	0.224591773
ASS1	5.783327122	1.30556775	0.225746827
HSD17B2	5.535501904	1.2523926	0.226247344
PGF	111.9889459	25.4150301	0.22694231
PRODH	6.907157501	1.57591932	0.228157432
CRACR2B	7.316195644	1.68856534	0.230798276
DUSP26	1.058401223	0.24437937	0.23089483
PNMA3	23.36019629	5.40732866	0.231476165
JSRP1	0.72346352	0.16809369	0.232345765
KANK4	1.430138986	0.33244103	0.232453654
TMC4	0.61345347	0.14272653	0.232660719
FOXH1	9.305190398	2.16542527	0.232711549
ANO9	1.76834038	0.41157111	0.232744279
CST7	0.42639656	0.1	0.234523468
PTP4A3	3.279123091	0.7693373	0.234616779
WNT11	2.235869194	0.52598264	0.2352475
C22orf24	0.885753028	0.20840255	0.235282909
UPK3A	0.854519009	0.20142444	0.235716743
NRAP	7.229420805	1.71003736	0.236538639
CPB2	21.79187922	5.20625523	0.238908044
GDPD3	1.814027839	0.43817383	0.241547467
AIFM3	1.224717666	0.29654377	0.242132356
ANGPTL6	0.848013739	0.2054153	0.242231103
CLDN14	1.194151189	0.2912023	0.243857145
GAMT	490.9602039	120.103399	0.244629602
CLDN5	0.577090454	0.14168564	0.245517219
GC	20.44946831	5.02253118	0.245606932
ITPRIPL1	1.447803557	0.35600859	0.245895649
GGT5	0.745086	0.18350253	0.246283688
SLC22A31	3.374568741	0.83154279	0.246414537
GLUD1	126.1943452	31.1678278	0.246982761
LRG1	2.930848371	0.72581157	0.247645555
HCAR2	1.128978987	0.27981349	0.247846503
CXCL6	1.819926902	0.45171476	0.248204893
SERPINF2	164.0240342	40.7730389	0.248579661
TNFRSF14	0.880331108	0.22121104	0.251281633

RSPH9	0.807985542	0.20310006	0.251365958
LOC10798497	2.970689188	0.75028873	0.252563861
GALNT1	23.62074678	5.97362788	0.252897503
C15orf62	1.319561274	0.3344294	0.253439845
SARM1	3.881374525	0.98589838	0.254007536
RBP1	2.631274468	0.67075971	0.254918185
RHBDL1	3.991651297	1.01808621	0.255053894
RNF208	81.66640953	20.9338225	0.256333328
PER1	4.689652717	1.20393037	0.256720581
ALDOC	35.9247002	9.23094373	0.256952561
SLC46A1	7.861722273	2.02014333	0.256959386
VTN	1002.71554	258.623154	0.257922755
VWCE	11.77495389	3.04027609	0.258198556
GCHFR	199.3438134	51.5826462	0.258762212
TRPM2	0.61597455	0.15954376	0.259010306
PDZK1	15.32361219	3.97290603	0.259266939
VAX2	4.719616267	1.2282785	0.260249654
TBX1	2.290106791	0.5975359	0.260920538
FADS6	0.524543778	0.13709439	0.261359295
FOLH1	0.80461653	0.21081821	0.26201078
GRB7	0.441166946	0.11568914	0.262234378
AGPAT3	8.050190413	2.11379353	0.262576836
ATP6V1B1	2.245021747	0.59321228	0.264234537
PRR5	75.25089256	20.1267631	0.267462118
TMEM151A	2.091664071	0.5612607	0.268332143
ALDH3A1	1.273650901	0.3423848	0.268821541
TF	249.757309	67.142812	0.26883222
TMEM86B	109.6618746	29.4852835	0.268874516
SH3BP1	8.183636553	2.20038356	0.268876012
ACY3	5.777891245	1.55708907	0.269490893
CERS4	36.8425655	9.9536147	0.270166167
LCAT	31.66548834	8.56212397	0.270392924
GOLT1A	17.01347794	4.60551932	0.270698286
BIN1	57.11747072	15.5016567	0.271399565
GHRL	1.19155571	0.32441028	0.272257756
RPS6KL1	5.697685304	1.56014095	0.273820134
APOA5	0.608031466	0.16658366	0.2739721
NEURL3	0.560620336	0.15366209	0.274092974
AFP	7186.212193	1977.51491	0.275181814
GAL3ST3	0.593723954	0.16390962	0.27607041
ZASP	31.88794313	8.82699971	0.276813079
C2CD4C	0.974500388	0.26979827	0.276858047
B3GNT7	1.205336324	0.33412957	0.27720858
ADM2	1.253380592	0.3486688	0.2781827
MIXL1	1.296251433	0.36130532	0.278730897
FGA	447.4837252	125.010978	0.279364302

LRRC71	0.599993691	0.16897917	0.28163491
ABCA7	9.949142628	2.80574369	0.28200859
ACOT4	1.505318524	0.42743815	0.283951965
C15orf48	0.545926885	0.15566105	0.28513168
FAM20A	3.630088295	1.03774939	0.285874422
CTSH	16.23909604	4.65436704	0.286614909
FBLN5	8.677188731	2.49451843	0.287480025
PIPOX	0.653737448	0.18796846	0.287528976
ANKRD53	0.581365051	0.16729462	0.287761735
MVB12B	11.60961078	3.34297227	0.287948695
CCNE1	15.64989258	4.52041507	0.288846396
CXCL16	11.1571041	3.24441897	0.290794003
APOA4	11.28556541	3.29723172	0.292163627
PLAU	2.992025338	0.87597684	0.292770528
KCND1	1.296188072	0.38095858	0.293906874
CX3CL1	14.48151644	4.27261603	0.29503927
ASB9	1.719757585	0.50824767	0.295534485
BST2	0.338311129	0.1	0.295585901
C2orf48	1.280999542	0.37889054	0.295777265
ANXA9	11.15024145	3.29898459	0.29586665
MSANTD1	3.758523737	1.112792	0.296071563
FOXA3	111.2943975	32.9830877	0.296358922
PI3	1.305428452	0.38724719	0.296643748
CPT1B	4.155594072	1.23900266	0.298152956
SYTL1	2.676086007	0.80026377	0.299042621
44444	10.49055446	3.14183834	0.299492114
NUPR1	1.497922111	0.44898014	0.299735302
CDX2	3.670185572	1.10112245	0.300018195
URAD	0.332604445	0.1	0.300657437
RCN3	13.9326036	4.20333888	0.30169084
UBA7	1.071209566	0.32415627	0.302607709
ENHO	7.308580065	2.21589441	0.303190824
ELOVL3	0.402163883	0.12200124	0.303361991
ZBTB16	0.833734792	0.25433857	0.305059327
BARX1	0.796400758	0.24317134	0.305337904
UBD	1.725059239	0.52864631	0.306451105
EBF4	16.2419974	4.98356402	0.306831968
MT1M	14.03564951	4.31948711	0.307751138
DUSP9	317.8909019	98.2789805	0.309159463
NRROS	0.860654788	0.26615499	0.309247093
CHAC1	1.50721292	0.46624843	0.309344767
LOC10537437	1.001183972	0.30997518	0.309608616
MVD	896.5643245	278.325876	0.310436037
PHGDH	126.6936478	39.4664237	0.311510675
LCN15	2.330804838	0.72651552	0.311701567
FAM83H	7.198751832	2.24391254	0.311708557

EXOC3L4	27.0738364	8.44982361	0.312102928
RAB26	2.686569055	0.83884857	0.312237857
CHST3	9.346007094	2.92604484	0.313079672
TMEM97	104.9982749	32.9151012	0.313482305
LTK	1.906272227	0.59838692	0.31390423
NUCKS1	46.03617533	14.4526719	0.313941631
NXPH2	0.977445753	0.30720696	0.314295658
PFKFB4	3.3442551	1.05336055	0.314976135
TMEM249	1.581652471	0.49833528	0.315072549
OGDHL	0.400766709	0.12627941	0.31509455
ACP5	15.02346097	4.78314026	0.318378053
OPLAH	18.56523936	5.91314858	0.318506455
KRT23	0.539527676	0.17200676	0.318809894
MIOX	1.109263413	0.35408979	0.319211639
RND2	6.475109697	2.06746798	0.319294664
ATP2B2	3.064956018	0.97875721	0.319338094
HIST1H3A	3.853908415	1.23102305	0.319421977
S1PR2	2.861074475	0.91402733	0.319469953
CCL20	23.31679667	7.47085838	0.320406722
TMEM89	2.376616009	0.76306226	0.321070908
CCDC154	1.291474071	0.41473517	0.321133173
PRSS50	1.046477331	0.33628054	0.321345268
AMIGO1	0.633269415	0.20354281	0.321415821
CMTM1	1.243309986	0.39968916	0.321471847
AFF3	2.221109451	0.71414783	0.321527528
HUS1B	0.632541756	0.20340152	0.321562205
SMCO4	28.55224531	9.18993921	0.321863976
KIF5C	2.464522136	0.79374723	0.322069428
ACSS2	63.86120351	20.5930675	0.32246601
RBM14-RBM	11.61002889	3.74807489	0.322830798
GP1BB	14.86552624	4.80649621	0.323331723
PINLYP	0.439296065	0.14235128	0.324044064
RNF215	4.3010396	1.39714573	0.324839077
DBP	2.915913782	0.94942266	0.32560039
HSD17B8	1.027425483	0.33457077	0.325639937
TNFSF12	1.407594836	0.45907372	0.326140523
HOXA3	3.524959743	1.15224287	0.326881142
GPX2	8.451110759	2.76454695	0.327122319
PIGZ	3.614712458	1.19003715	0.329220418
CCDC78	1.648200407	0.54351991	0.329765667
AMHR2	0.638656537	0.21074109	0.329975621
NOXA1	2.63416422	0.87016306	0.330337438
PARP10	1.798407993	0.59465972	0.330658963
RAB37	1.455751624	0.48324781	0.331957596
TSPO	21.59667161	7.19895654	0.33333639
HABP2	34.87915349	11.6277491	0.333372457

SLC51B	6.041806321	2.01864824	0.334113365
HGFAC	1.266401204	0.42314187	0.334129396
HSPA12B	0.601414792	0.20110225	0.334381949
APOE	5896.950882	1974.13399	0.334771992
KCNE5	1.053122516	0.35345725	0.33562785
VWA5B2	0.711008054	0.2388053	0.335868634
CHDH	2.350003069	0.79054306	0.336400861
STAT5A	3.700374329	1.24502912	0.336460318
TRPV4	7.118954316	2.39674261	0.336670598
SERPINC1	1.544651518	0.52075339	0.337133252
SLC25A18	11.24952206	3.79334377	0.337200438
FZD5	12.05882389	4.067868	0.337335386
SPINK1	2483.52729	837.862861	0.337368091
TNNC1	0.97226667	0.32864216	0.338016481
EBP	1517.791534	513.127948	0.338075379
PSPH	37.1856624	12.5743506	0.338150507
ACOX2	1.649924645	0.55906517	0.338842853
C3	24.05503984	8.16217557	0.339312494
MFAP4	0.533420012	0.18114989	0.339600859
TNFAIP3	0.689415148	0.23434778	0.339922583
CCDC183	10.11215743	3.4384953	0.340035776
ANGPTL3	73.79107461	25.1215565	0.340441668
CAMK2N2	8.179754855	2.7912527	0.341239163
TRIM17	1.613444448	0.5508072	0.341385911
DHCR7	288.3002077	98.4329292	0.341425107
FAM213B	4.027142497	1.37502958	0.341440509
NTAN1	19.52313904	6.67078926	0.341686306
FADS1	26.52660151	9.06484178	0.341726466
LPGAT1	4.409223913	1.5074855	0.341893614
ADAM33	0.422653271	0.14451327	0.341919205
OAF	7.370825205	2.52149483	0.342091253
ZBTB47	1.808736669	0.62014327	0.342859896
ZNF853	1.893912044	0.64997388	0.343191165
CITED1	0.539571876	0.18534993	0.343512954
GMDS	4.368052048	1.50266413	0.344012413
PPFIA3	2.82694621	0.97252394	0.344019257
LRRC45	38.84679565	13.3753279	0.344309684
SLC43A3	5.750999051	1.98279903	0.34477471
YDJC	71.13302824	24.5547456	0.345194718
ARHGEF16	1.571649245	0.54339633	0.345749111
TMEM176A	65.53110058	22.7264137	0.346803479
RBP5	6.931555915	2.40803367	0.347401608
ISYNA1	10.88727145	3.78246148	0.347420517
SERPINA1	666.876053	232.149263	0.34811456
ESPNL	1.163036588	0.40583269	0.348942323
C17orf96	1.227037628	0.42824864	0.349010196

GATSL3	2.158592363	0.75406881	0.349333587
METTL7B	11.6695227	4.08013468	0.349640237
AGXT2	1.460096461	0.51053142	0.349655953
IFI6	5.747419557	2.01208652	0.350085199
FRAT1	12.32897471	4.32198829	0.35055537
CRB2	0.63582303	0.22366856	0.351778017
CDK18	1.212285778	0.42832991	0.353324209
FOXD2	1.372632407	0.48578168	0.353905151
LSP1	0.431253652	0.15262399	0.353907702
F2	85.40331686	30.2255948	0.353915936
NEK8	6.090883992	2.15741746	0.354204326
RASSF5	0.734480443	0.26047711	0.354641321
SLC7A10	16.85033762	5.97802165	0.354771625
ST20-MTHFS	11.35338676	4.02839691	0.354818963
HOXC13	0.530745377	0.18855589	0.355266202
VPS25	51.8757435	18.4351919	0.355372099
METRN	8.276398685	2.94140546	0.355396782
SCAMP5	5.428703016	1.92965021	0.355453264
TLX2	1.736229597	0.61763225	0.355731897
SERPINA4	6.838813605	2.44992697	0.358238595
SLC52A1	0.401663405	0.14392959	0.358333841
LOC10106017	0.895956332	0.32128419	0.358593578
NLGN2	1.929394411	0.69370189	0.35954385
FMO1	0.680029556	0.24479977	0.359984009
PCSK4	4.620057285	1.6761316	0.36279455
TMEM198	1.662476187	0.60644855	0.364786308
OLR1	0.389553705	0.14216612	0.364946141
ZFPM1	11.62543544	4.24484053	0.365133896
GLTPD2	209.2457873	76.4801363	0.365503828
CCDC69	23.33724568	8.54287573	0.366061867
BEX1	0.945147474	0.3461484	0.366237452
ARHGAP27	0.795002555	0.29117335	0.366254604
MMP23B	2.700231816	0.98902202	0.366273006
PCYT2	135.5312064	49.6838057	0.366585726
SLC29A4	70.92108988	26.0018678	0.366630967
LOC1079872(	0.935934736	0.3434652	0.36697559
HIST1H1B	0.637934584	0.23429109	0.367265071
IGLV1-51	0.271982324	0.1	0.367670953
DEF6	2.532621055	0.93171573	0.36788596
DHRS4L2	19.95112795	7.34106087	0.367952173
NKX3-2	1.942152475	0.71542593	0.368367538
RRAGD	5.074244916	1.87165698	0.368854284
AMH	2.89782344	1.07257893	0.370132604
NOC4L	53.31561773	19.7439573	0.370322208
MST1L	1.80372292	0.66817674	0.370443119
C16orf59	32.24484848	11.96018	0.370917543

APOM	19.80474478	7.34924836	0.371085235
PRCD	0.348669922	0.12996892	0.372756316
ARPIN	2.373808837	0.88488258	0.372769097
KCNH3	0.635175268	0.23686084	0.372906271
HIST1H3J	0.8620472	0.32194553	0.37346624
C14orf159	0.676358483	0.25260351	0.37347578
RAB11FIP4	2.801964294	1.04679266	0.373592433
CDH4	0.594029985	0.22216716	0.373999908
RHOV	2.09398626	0.78445137	0.374621066
JPH3	0.372989179	0.13975392	0.374686259
ORM2	30.7426041	11.5386096	0.37532961
RAC3	109.4802729	41.1799476	0.376140344
FGFR4	172.1741083	64.8426731	0.376611058
SMARCD3	75.35514898	28.4155198	0.377087965
RHBG	1.646464053	0.62118431	0.377283858
AMBP	4972.923673	1879.57171	0.377961101
NOXO1	2.011768356	0.76039156	0.377971727
ORM1	70.21651689	26.5908784	0.37869834
DENND6B	4.102320843	1.55881457	0.379983582
WFIKKN1	2.125736353	0.80799256	0.38010008
STARD10	175.7552016	66.8865126	0.380566333
RHPN1	4.279102813	1.63014368	0.380954548
MICB	1.777198848	0.67772459	0.381344265
MPZL2	2.158317631	0.82308782	0.381356204
GYLTL1B	1.519843506	0.58022616	0.38176704
NAT14	102.9018607	39.3139932	0.382053278
ONECUT1	4.206279308	1.60873688	0.382460784
NPDC1	3.795031219	1.45389676	0.38310535
DNAJC17	60.60895618	23.2304189	0.383283599
ACAD11	2.88338075	1.10589378	0.383540668
ZNF296	12.34913195	4.74896112	0.384558294
TLE2	2.823304343	1.08661343	0.384872935
POLR2L	438.0973569	168.620755	0.384893341
KCTD14	1.496801442	0.57627368	0.385003422
NFKBIE	15.24803781	5.87514127	0.385304741
PTRHD1	31.51623956	12.1556775	0.38569568
TMEM189-UI	58.95809174	22.7686398	0.38618346
GSDMD	54.41520427	21.0352653	0.386569628
ENO3	14.64504672	5.68077402	0.387897296
CDA	0.542675335	0.21051038	0.387912188
DPP7	127.4688798	49.612478	0.389212474
LOC10272375	0.402038608	0.15655115	0.38939333
SLC22A5	1.304844175	0.50852177	0.38971839
CELSR3	0.313159165	0.12217229	0.390128412
SNAP25	1.240576196	0.48504042	0.390979954
DLL3	0.482142847	0.18903899	0.392080885

CD160	1.661426779	0.65221352	0.3925623
SAMD11	15.47948899	6.08157967	0.392879873
HLX	0.845885602	0.33275551	0.39338122
DAO	0.404394341	0.15910304	0.393435384
TRIM71	6.363206122	2.5125236	0.394851832
GAR1	20.08103395	7.929054	0.394852875
ZMYND15	1.240787978	0.49003662	0.394939855
SYCP2	0.31920478	0.12611767	0.395099555
DEPDC4	0.628567564	0.24844677	0.395258662
CNFN	0.675447964	0.26759868	0.396179557
LRRC3	0.940190434	0.37267092	0.396378121
RGS14	12.43498037	4.93348992	0.396742879
PRDM16	2.194879953	0.87229308	0.397421773
FGG	537.5933968	213.695661	0.397504252
TSPAN18	0.370301693	0.14723507	0.39760841
TMEM238	2.667784421	1.06154044	0.397910877
PSMB3	681.4831632	271.189173	0.397939653
GJB2	0.308088296	0.12272707	0.398350316
PRR22	5.987800023	2.38642159	0.398547309
FBXO22	5.060224824	2.01695374	0.398589749
ASNS	15.65735901	6.24394559	0.39878664
B4GAT1	102.7786814	40.998449	0.398900321
SYP	2.545358201	1.01569634	0.399038665
SNAI3	1.801149148	0.7193226	0.399368704
RUSC1-AS1	85.44695237	34.2177518	0.400456083
HOXA2	0.919479328	0.36850665	0.400777528
TMSB15A	0.646934777	0.25929424	0.400804294
C19orf35	2.769339652	1.11136345	0.40130991
GPAM	0.853407473	0.34381024	0.402867621
EFEMP2	21.70656844	8.76982762	0.404017228
HMGCR	23.9323969	9.67118395	0.404104277
UBE2V1	66.85029943	27.0214018	0.40420764
MTFP1	81.8539103	33.0974646	0.404347996
PARP12	7.280896298	2.95067863	0.405263104
CCDC28B	4.463278926	1.80900806	0.40530921
FBXO44	22.591869	9.15918799	0.405419666
LOC388780	0.535315435	0.2170325	0.405429185
SLC27A5	0.356700889	0.14485833	0.406105898
LOC10798687	10.67444594	4.33925052	0.406508267
MTMR4	1.87014539	0.76063346	0.406724239
MYO1A	1.743047092	0.70904571	0.406785169
FASN	165.8591495	67.4856659	0.406885397
PTPRCAP	18.7747774	7.64443498	0.407165146
E2F2	2.559481974	1.04270652	0.407389671
MSI1	5.671013292	2.31095046	0.407502212
AMN	31.8708512	12.9875075	0.407504255

SLC25A10	54.21581624	22.1027402	0.407680668
SNRNP35	17.24789412	7.03608172	0.407938596
CRB3	10.7862641	4.40094439	0.40801378
CLCN2	4.440030148	1.81160439	0.408016236
SPTSSB	18.85345872	7.69304048	0.408043988
IFI35	5.636730133	2.3066712	0.409221507
PLXNC1	6.637084175	2.72119902	0.409999173
SYCP3	1.190594495	0.48822009	0.410064122
KLF14	0.592241299	0.24336481	0.410921719
GLYCTK	38.71460276	15.9436982	0.411826471
SERPINA6	27.40311603	11.2991749	0.412331754
NEU1	89.12730171	36.7525193	0.412359834
TPRN	61.47892901	25.3825564	0.41286595
C16orf74	3.818303801	1.57675336	0.412946022
GALK1	84.1390803	34.7877538	0.413455361
MMP17	17.07153812	7.07439192	0.414396868
DTX4	2.226674949	0.92382196	0.41488856
ISOC2	244.080592	101.384177	0.415371727
P2RY11	4.245583021	1.76352494	0.415378744
BCAS4	19.12656403	7.94737603	0.415515093
SLC29A2	84.44846155	35.1228479	0.415908677
AS3MT	25.32395794	10.5498827	0.416596914
NRARP	0.725396233	0.30238451	0.416854264
FAM117B	1.099034683	0.45834995	0.417047756
DCST2	0.393401983	0.16408979	0.417104628
INS-IGF2	156.6062603	65.3416054	0.417234951
DENND1C	2.404343507	1.00321358	0.41725052
ABHD8	28.2763002	11.8003522	0.417323064
DLC1	7.884551419	3.29163543	0.4174791
AZU1	1.621050638	0.67783204	0.418143653
NKX6-3	5.319620454	2.22534147	0.418327113
IDUA	13.48846423	5.64652841	0.418619074
HDHD3	63.62855351	26.6378836	0.418646695
HUNK	0.461710149	0.1933764	0.418826412
ADRA2C	2.890540562	1.21074781	0.418865532
B4GALNT2	1.407092108	0.58945772	0.418919075
ADAM32	0.656153905	0.27493764	0.419013955
BCL2L13	11.76403422	4.93730534	0.419694915
AIF1L	0.238172996	0.1	0.419862879
CNNM1	12.53027804	5.26179986	0.419926824
C12orf42	0.606759267	0.25497282	0.420220719
PIDD1	9.523560135	4.00227912	0.420250312
CEBPA	262.2526363	110.282752	0.420521042
FN3K	30.39133187	12.79674	0.421065456
WDR66	3.640866186	1.53440828	0.42144045
THAP7	98.83890151	41.6561726	0.421455236

LOC10798464	8.21135915	3.46097675	0.421486466
PPP1R27	0.707857424	0.29916764	0.422638271
RTKN	24.08143554	10.1804341	0.422750299
SULT1E1	0.236426427	0.1	0.422964561
CFB	3.312278901	1.40239865	0.423393891
CENPM	58.56704572	24.7996388	0.423440154
PARD6A	5.111864942	2.16561452	0.423644706
TRMU	35.01252714	14.8419705	0.423904577
CIB2	19.01430977	8.06177032	0.423984379
LAPTM5	0.235512858	0.1	0.424605268
DGAT2	46.06525449	19.5973809	0.425426519
TMEM184C	2.377252113	1.01150219	0.42549218
IGF2	159.7654951	67.9811592	0.42550589
HRASLS2	0.855723156	0.36412439	0.425516574
EPO	0.328232181	0.13988344	0.426172241
LINC00083	0.234512024	0.1	0.426417368
KIF12	71.81365211	30.6242509	0.426440516
CPLX1	11.90152348	5.08226222	0.427026189
TSSC4	53.91838205	23.0766007	0.427991342
ITGB4	0.915885703	0.39202224	0.428025285
MEIS3	0.400814127	0.17168241	0.42833422
SALL1	0.516057751	0.22117643	0.428588518
GATM	14.30197225	6.13033818	0.42863586
DRICH1	0.752183319	0.32298217	0.429392892
SDC1	60.84663224	26.131184	0.429459825
SDHAF2	128.3923235	55.1466782	0.429516942
MVP	18.59312696	7.98874074	0.429660958
SH2D3A	1.503319493	0.64704994	0.430414125
NR6A1	7.355970372	3.1732972	0.431390699
PGAP3	7.224651961	3.11882005	0.43169139
SYCE2	0.617490105	0.26658361	0.431721265
FAM69B	62.58590925	27.0427981	0.432090839
H1FX	86.67284323	37.551798	0.433259099
SPATA2L	36.11613134	15.6516489	0.433370031
FAM229A	11.56450408	5.03265201	0.435180962
TST	138.2320578	60.1896529	0.435424704
SLC37A1	0.403332135	0.17572446	0.435681761
ADCK5	21.96000239	9.57161765	0.435865966
PAK6	0.459996206	0.20065461	0.436209277
TNFAIP8L1	19.98977678	8.73028733	0.43673761
RCN1	36.31834265	15.8701294	0.436972843
PLIN4	0.471969781	0.20625247	0.437003541
HIST1H2AB	2.009111957	0.87843787	0.43722694
MRPL23	124.683459	54.562664	0.437609483
WASF3	0.979123545	0.4291993	0.438350506
KCNJ16	0.86268543	0.37867395	0.438947892

RNF166	13.60258077	5.97173686	0.43901499
FITM1	1.552676217	0.68174954	0.439080301
FAM151A	7.696109241	3.37980164	0.439157181
HIST1H2AH	0.478647153	0.21028063	0.439322837
TCAP	0.478253958	0.21016611	0.439444576
BORCS6	22.46174554	9.88311297	0.439997548
LRFN3	13.83513065	6.08997	0.440181604
MGMT	21.58416921	9.50332262	0.440291332
LSM2	46.02325424	20.2731669	0.440498337
C10orf11	2.036368416	0.89760831	0.44078876
PGM1	22.29062776	9.82560438	0.440795319
DERL1	11.64212507	5.13450866	0.441028474
SDSL	9.3456458	4.12470106	0.44135003
CERS1	5.474850068	2.41709745	0.441491076
ST6GALNAC	0.262036727	0.11573101	0.441659518
SKP2	18.2723717	8.0731022	0.441820161
HNF1B	9.133709329	4.03775989	0.4420723
BEX2	6.087045479	2.69113731	0.442108954
FAM169A	1.635355436	0.72340873	0.442355658
NAT8	9.039554629	4.00545525	0.443103163
NGEF	1.015222516	0.44985697	0.443111697
ADAMTSL5	8.897284122	3.94330103	0.443202776
CRYBB3	0.540506176	0.23996037	0.443954912
CXCL1	17.0600253	7.57613824	0.444087163
EPPK1	0.756707136	0.33607607	0.44412964
CCND1	200.6163123	89.1225628	0.444243849
CRIP1	636.1612171	283.006564	0.444866107
SLC25A45	5.954846696	2.65078149	0.44514689
ZNF709	0.248707312	0.11082772	0.445615028
DLK1	0.605482632	0.26987078	0.445711836
MUL1	8.217828494	3.66335274	0.445781114
SYT7	1.190878504	0.53099047	0.445881311
TCEA3	16.67536217	7.44797066	0.446645211
PRICKLE4	0.80078095	0.35781279	0.446829793
RGN	7.577763999	3.39033997	0.447406382
DGAT1	41.08439068	18.389898	0.447612772
HEXDC	27.58678642	12.3597813	0.44803266
LOC10192983	0.802471636	0.35967725	0.448211797
GYG2	13.6565484	6.12852723	0.448761067
PRR5-ARHGA	0.605680557	0.27182548	0.448793474
DNAJC4	13.59105783	6.11255562	0.449748334
TGM1	0.646540874	0.29101542	0.450111396
IGSF23	1.268647902	0.57127821	0.450304777
SLC31A1	19.29892167	8.69288722	0.450433831
RECQL4	49.65053078	22.3680295	0.450509373
C19orf66	2.420790139	1.0908489	0.450616881

NPPB	26.19302016	11.8033339	0.450628977
VMO1	1.685702693	0.75976344	0.450710227
POMK	0.511464795	0.23073316	0.451122277
PIM2	14.28504229	6.45472564	0.45185205
RSC1A1	1.383338331	0.6263307	0.452767544
ABCB9	1.578030568	0.71553903	0.453438001
CMTM4	2.194213732	0.99530151	0.453602809
CXCL3	0.623811564	0.28307155	0.453777332
CAPN12	99.42915128	45.136594	0.45395735
SAT2	120.3406902	54.6384803	0.454031635
BIK	4.877788056	2.21511991	0.454123853
CCDC39	0.462305263	0.21010894	0.454480955
ATP6V0E2	16.95660009	7.71030774	0.454708355
WDR18	173.1698785	78.7679682	0.454859522
SCUBE3	0.474751606	0.21596768	0.454906685
STAP2	9.70622087	4.41563905	0.454928763
JAML	7.746963027	3.52457438	0.454962076
SHF	11.31916369	5.14988217	0.4549702
FAM124A	0.432757517	0.19691531	0.455024591
MCRIP2	144.135574	65.619295	0.455260927
TMEM245	11.95802745	5.45291978	0.456004956
MESP2	0.363301315	0.16573108	0.456180786
ENPP2	0.849052112	0.38733363	0.456195356
LRRC75B	19.74048617	9.01716031	0.456785118
C7orf50	188.8950104	86.3054476	0.456896386
GSTA1	0.95206681	0.43502093	0.456922694
SLC2A4	0.697650863	0.31898531	0.457227719
MAPK11	4.370410942	1.99836007	0.457247636
TONSL	26.17225362	11.9778462	0.457654369
FZD9	1.603486803	0.73418423	0.457867334
FGF12	0.83748494	0.38352516	0.45794873
WNT10B	1.086122719	0.49767166	0.458209418
PLCD1	3.792079436	1.73912827	0.458621266
CARMIL2	4.203641845	1.92792512	0.458632107
SLC6A12	0.693967788	0.31851691	0.458979392
SLC44A1	4.300808426	1.97555678	0.459345449
PKLR	0.937926365	0.43120428	0.45974215
RHOD	368.4844674	169.441302	0.459832957
USP2	1.823877467	0.83890678	0.459957861
UPB1	1.389945177	0.63956242	0.460134996
TREX2	0.583743109	0.26906767	0.460935065
ELMO3	4.522725367	2.08496345	0.460997139
ECT2L	0.370844346	0.17099484	0.461095998
BAHCC1	1.003686335	0.46291869	0.461218487
SLC25A40	1.6071169	0.74153701	0.461408256
METTL21B	1.147457131	0.52958963	0.461533263

LINGO3	0.755551402	0.34903747	0.461963895
SCT	0.415102562	0.19177332	0.461990218
TMEM92	0.714338015	0.3300469	0.462031829
CLDN22	1.06400284	0.491765	0.462183916
COL2A1	1.882821398	0.87066896	0.462427804
EMX1	0.356102452	0.16477518	0.462718455
YY2	0.29933887	0.13870484	0.463370622
KRT17	0.215711106	0.1	0.463582993
LPAR2	1.175009734	0.54492528	0.463762355
HMBS	29.27142403	13.5828255	0.464030226
TLN2	1.919814785	0.89099332	0.464103793
DHRS4	43.84467712	20.3542806	0.46423607
TNK2	12.83587889	5.96252346	0.464520078
CKB	782.3515465	363.477607	0.464596266
FBLN1	85.6333982	39.7892374	0.464646251
WDR86	11.78282717	5.47859753	0.4649646
NRG4	0.819009282	0.3808568	0.46502135
TAP2	0.871164964	0.40520702	0.46513237
SELO	76.36089968	35.5244235	0.465217456
PLEKHA6	0.612865113	0.28558275	0.465979774
COL6A1	33.23531529	15.4924607	0.466144538
THEM5	1.901162171	0.88688017	0.466493697
ASGR2	163.772066	76.4130971	0.466581994
HYAL1	0.58745063	0.27423534	0.466822784
IFITM3	30.45488012	14.2347404	0.46740425
GSAP	0.664366961	0.31133458	0.468618404
MRPS17	209.3725523	98.133642	0.468703471
TLCD1	22.42089538	10.5119528	0.468846252
FADS2	187.978448	88.1818383	0.469106109
SLC19A1	77.46080409	36.3652039	0.469465871
TCEAL3	1.71892694	0.80726641	0.469633928
GPAT2	1.193869554	0.56081547	0.469746019
LOC10013035	0.995501886	0.46820811	0.470323679
TP53I13	227.8364485	107.265394	0.470799974
TAZ	20.00201871	9.42108307	0.471006612
FAM107B	13.53679955	6.37620946	0.47102784
PCYT1B	0.942431346	0.44413756	0.471267812
SLC17A9	111.7062246	52.7252579	0.471999283
TMEM176B	42.17228839	19.9084054	0.47207316
ZDHHC19	1.097203683	0.51822587	0.4723151
SYNGR3	2.675206797	1.26383429	0.472424896
CACNB1	1.764831018	0.83438206	0.472782973
NOTCH1	2.428968881	1.14881544	0.472964247
ACTR5	8.034635868	3.80180786	0.473177369
LGALS3BP	1.550587191	0.73372322	0.47319056
GUCA1B	0.902331633	0.42714966	0.473384337

PROM1	24.01842728	11.3713192	0.473441458
SCARF2	12.16589198	5.76397147	0.473781247
TMEM185A	2.53694108	1.2019908	0.473795315
PKDCC	29.03483974	13.7589235	0.473876337
ZNRF1	31.20834682	14.7895554	0.473897433
LGALS4	1.938597252	0.91898426	0.474045996
FBXO27	0.305621542	0.14491531	0.474165888
CCS	398.9068944	189.213268	0.474329399
C14orf1	83.7927409	39.7459564	0.474336512
CBLC	0.625950893	0.29694919	0.474396937
ZNF684	1.042454234	0.4945778	0.474435984
CYSRT1	5.202053867	2.47209629	0.475215435
APOH	14.70875996	6.99010575	0.475234199
PDK2	1.203813822	0.57238573	0.475476954
LIN7B	31.19211585	14.831605	0.475492111
FBXO6	8.973867079	4.267088	0.475501583
WNK4	1.323047741	0.62922851	0.475590179
RNF207	3.261966679	1.55180299	0.475726192
IL17RB	4.317730711	2.05428877	0.475779733
TTC38	17.94467129	8.5393921	0.47587342
PRR36	0.382183481	0.18218567	0.476696878
UNC119	41.98274798	20.0199807	0.476862084
FAHD2B	3.674535631	1.7534802	0.477197766
PROC	30.32996292	14.4799922	0.477415428
HMOX1	37.67132332	18.0151916	0.478220303
DOK3	2.917196472	1.39553549	0.478382413
BOLA2B	1.950543331	0.93453264	0.479114011
SEMA7A	0.961640595	0.46074351	0.479122361
TMEM54	84.44609355	40.4613365	0.479138049
GLYATL1	0.647526698	0.31049596	0.479510662
FRG1	10.81006317	5.18580832	0.479720446
NDRG1	12.31875565	5.9108504	0.479825282
FCGRT	68.57086006	32.9133505	0.479990341
LSR	7.9618873	3.82572297	0.480504537
TM4SF5	219.0152795	105.365816	0.481088884
TRPV3	1.073262965	0.51651547	0.481257146
AGMAT	20.77288602	10.0228087	0.482494763
PTGES2	167.4359972	80.8166654	0.482671986
LGALS2	48.27687413	23.3071791	0.482781446
PPP2R3B	1.226108496	0.59196705	0.482801525
LOC730098	4.934589262	2.38522763	0.483369032
PYGL	29.30297321	14.172017	0.483637512
PAQR6	0.727973477	0.35210083	0.483672609
HMHA1	26.58568612	12.8672178	0.483990436
RPL24	2814.029794	1362.46878	0.48416999
APOC1	1519.414086	735.718039	0.484211674

CFAP45	1.486685331	0.72058195	0.484690293
SDC4	96.89175704	46.9736551	0.484805483
CDC25C	17.08917645	8.28752874	0.48495776
ARHGAP1	16.60316853	8.05469694	0.485130108
HOOK2	5.709696743	2.77217966	0.485521349
CD3D	1.13587647	0.55254706	0.48644996
CXADR	12.76857564	6.21666562	0.486872286
NUDT18	17.09873287	8.32786591	0.487045793
CCR6	3.084009486	1.50274166	0.487268819
TEF	3.027768237	1.47559777	0.487354929
THAP3	14.06374535	6.85634752	0.487519316
C2	9.006206146	4.3914863	0.487606682
PRR7	2.46465994	1.2023479	0.487835209
MRPS33	65.77396315	32.0976426	0.487999218
CCR10	1.515259684	0.7396099	0.488107686
LOXL4	31.36894433	15.3231733	0.488482277
DUOXA1	0.33281029	0.16258995	0.488536421
EMILIN1	0.562491825	0.2751104	0.489092262
JAM3	2.185449779	1.06892413	0.489109446
MMAB	59.81852184	29.2584218	0.489119772
LOC10050650	0.415990945	0.20353607	0.489280054
IFT20	70.01150865	34.2581132	0.489321168
KCTD17	13.77388058	6.74301629	0.489550948
SCNN1D	0.524201496	0.25681128	0.489909484
ATP7B	2.096669571	1.02723973	0.489938778
TMEM191C	6.742307356	3.30623832	0.490371937
NUTM2B	0.597923307	0.29335959	0.490630799
AEBP1	2.239551022	1.09962397	0.491001971
AK4	11.42126852	5.61067463	0.49124794
AP1M2	216.9205723	106.703203	0.491899877
CA11	0.635960965	0.31286108	0.491950129
FZD4	10.37977006	5.1074921	0.492062163
SLC12A2	5.166451258	2.54316328	0.492245674
CIAPIN1	27.56128935	13.5921926	0.493162438
LOC10272518	0.452553776	0.22321714	0.49323894
TOMM40L	8.588104574	4.23645161	0.49329297
TSPYL2	13.3838698	6.60266006	0.493329669
DYRK1B	3.940862605	1.94489003	0.493518861
THEM6	9.673212281	4.7743169	0.493560645
PMP22	0.99246248	0.48989328	0.493613905
LPIN3	2.34897759	1.15979566	0.493744882
STAR	1.23719112	0.61100329	0.493863304
NAT6	11.66767053	5.76291011	0.493921224
AMOT	0.629128902	0.31076575	0.493961972
TMPRSS9	4.854181978	2.39823518	0.494055473
HPX	33.80270731	16.7031117	0.494135322

TMEM59L	1.26244754	0.62422464	0.494455905
PAGE1	0.966045082	0.47835222	0.495165522
MTHFS	18.27442294	9.04923778	0.495185966
FAM212B	2.088354557	1.03413643	0.495191981
GSTO2	1.301578629	0.64462313	0.495262536
TEN1	36.2701079	17.9767438	0.495635244
SEPN1	16.62280871	8.24286986	0.495877081
EMID1	7.732667409	3.83584117	0.49605666
EPHX1	65.85180696	32.7056304	0.496655019
NTHL1	68.35916047	33.951676	0.496666076
CCDC24	16.83711069	8.36446662	0.496787529
ENTPD2	0.302269549	0.15049498	0.497883379
PAH	4.968555931	2.47426248	0.497984225
RTN4RL2	4.612211909	2.29826047	0.498298975
BRAT1	66.89140581	33.3346848	0.498340324
C1orf229	0.341978672	0.17047606	0.498499098
UBE2J2	76.16113768	37.9864706	0.498764485
CCDC106	66.04913744	32.9703218	0.499178688
PEX11G	1.467880615	0.73274331	0.49918454
LOC10050555	0.434304847	0.21683916	0.499278712
IL17RC	27.87616078	13.9220064	0.49942338
HIST2H3D	0.897064686	0.4482165	0.49964792

Table S3. RIP-seq of TRIM71 in HuH-7 cells.

GeneID	GeneType	Input	TRIM71-RIP	Fold change	AccID
HIST1H4C	mRNA	32.0238	1552.149662	47.03122397	NM_003542.3
MIR1244-1	Precursor_m	1.933325	43.99199914	15.33822356	NR_036052.1
HIST1H4D	mRNA	35.37406	509.0522516	14.02241809	NM_003539.3
MIR1469	Precursor_m	0	12.23999976	13.23999976	NR_031715.1
HIST1H4K	mRNA	0.928433	23.56372835	12.73766198	NM_003541.2
MIR6741	miRNA	0	11.41428549	12.41428549	NR_106799.1
MIR15A	miRNA	0	10.3966263	11.3966263	NR_029485.1
MIR4635	Precursor_m	0	9.102531468	10.10253147	NR_039778.1
HIST4H4	mRNA	2.79206	37.00223229	10.02152825	NM_175054.2
MIR6837	miRNA	0	8.988749825	9.988749825	NR_106896.1
SNORD37	snoRNA	0	8.716363466	9.716363466	NR_002602.1
CDKN1A	mRNA	0.497762	13.38005166	9.601024264	NM_078467.2
MIR1244-2	Precursor_m	0	8.459999835	9.459999835	NR_036262.1
H2AFZ	mRNA	56.91775	534.7309505	9.249857787	NM_002106.3
SNORD36B	snoRNA	0	8.102535053	9.102535053	NR_000017.1
MIR6859-3	miRNA	2.416657	29.60999942	8.95905057	NR_107063.1
RPL27	mRNA	46.14594	406.1825375	8.636641277	NM_000988.3
RNA5SP398	ncRNA	0	7.413401917	8.413401917	RNA5SP398
MIR16-2	Precursor_m	0	7.102222084	8.102222084	NR_029525.1
MIR6819	Precursor_m	0	7.073114616	8.073114616	NR_106877.1
RNR1	rRNA	1.205795	16.73377326	8.039628454	RNR1
RPS21	mRNA	46.06294	364.9977201	7.776771316	NM_001024.3
MIR6835	miRNA	0	6.741562368	7.741562368	NR_106893.1
SNORA21	snoRNA	1.235584	16.22030044	7.702820251	NR_002576.1
HIST2H4B	mRNA	2.074907	22.51727229	7.648124245	NM_001034077.4
SNORD56	snoRNA	2.314544	24.30760516	7.635319477	NR_002739.1
RPL27AP6	ncRNA	1.62384	18.75913007	7.530614268	NM_001089587.2
UQCRQ	mRNA	8.087033	67.41845925	7.529241075	NM_014402.4
MIR6728	miRNA	0	6.463820099	7.463820099	NR_106786.1
C8orf59	mRNA	14.2898	108.4767087	7.160115879	NR_120681.1
HIST1H1E	mRNA	72.01329	517.7519899	7.10489829	NM_005321.2
MIR574	Precursor_m	0	5.992499883	6.992499883	NR_030300.1
MIR1228	Precursor_m	0	5.910410844	6.910410844	NR_031597.1
MIR579	Precursor_m	0	5.870203967	6.870203967	NR_030305.1
MIR885	miRNA	0	5.830540427	6.830540427	NR_030614.1
MIR4792	Precursor_m	0	5.830540427	6.830540427	NR_039955.1
TRI-TAT2-1	tRNA	0	5.830540427	6.830540427	TRNAI13
PSME2	mRNA	13.77033	98.98147681	6.769076821	NM_002818.2
MIR3605	miRNA	0	5.752799888	6.752799888	NR_037400.1
SNORD23	snoRNA	1.493933	15.68945424	6.692021406	NR_003048.1
MIR5187	miRNA	0	5.677105152	6.677105152	NR_049819.1
ALG5	mRNA	12.86865	89.42292699	6.519952002	NM_013338.4
MIR7113	Precursor_m	13.9265	95.06745577	6.436035442	NR_106963.1
MIR320E	miRNA	0	5.427169705	6.427169705	NR_036157.1

SNRPGP10	ncRNA	0.849264	10.77720909	6.36859146	SNRPGP10
MIR107	miRNA	0	5.326666563	6.326666563	NR_029524.1
MIR5188	miRNA	0	5.090973352	6.090973352	NR_049820.1
MIR1306	Precursor_m	0	5.075999901	6.075999901	NR_031706.1
MIR6515	miRNA	0	5.046315691	6.046315691	NR_106770.1
RNU7-171P	ncRNA	0	5.046315691	6.046315691	RNU7-171P
RPL12P16	ncRNA	1.831733	15.80187867	5.933425409	RPL12P16
HIST2H3A	mRNA	1.620638	14.46710031	5.902037146	NM_001005464.2
CRELD2	mRNA	1.832706	15.63841977	5.873683672	NR_104295.1
SNORD78	snoRNA	6.086394	39.94999922	5.778679064	NR_003944.1
MIR6820	Precursor_m	0	4.639354748	5.639354748	NR_106878.1
RPS16	mRNA	75.95017	428.8882447	5.58657985	NM_001020.4
MIR6790	miRNA	0	4.565714197	5.565714197	NR_106848.1
RPS15A	mRNA	64.76462	363.1256283	5.536801461	NM_001030009.1
HIST1H2AJ	mRNA	5.240677	33.41603579	5.514792238	NM_021066.2
MIR193A	Precursor_m	1.867416	14.70886335	5.47840311	NR_029710.1
MIR4449	miRNA	0	4.358181733	5.358181733	NR_039651.1
RPL36AP37	ncRNA	7.900608	46.67235486	5.356078362	RPL36AP37
CEBPA	mRNA	41.28616	223.136917	5.30047958	NM_004364.4
MIR6856	miRNA	0	4.293134245	5.293134245	NR_106915.1
MIR4728	miRNA	0	4.293134245	5.293134245	NR_039881.2
MIR3124	Precursor_m	0	4.293134245	5.293134245	NR_036070.1
HIST1H4L	mRNA	12.18951	68.74911954	5.288226843	NM_003546.2
MIR1282	miRNA	0	4.271881105	5.271881105	NR_031695.1
HCFC1R1	mRNA	1.308121	11.16214904	5.269286644	NM_017885.3
HIST2H3D	mRNA	9.349341	53.18507555	5.235606288	NM_001123375.2
MRPL2	mRNA	7.306425	42.37650256	5.222042103	NM_015950.4
SNORD9	snoRNA	0	4.188931957	5.188931957	NR_003029.2
GIPC1	mRNA	8.782423	49.66445946	5.179131824	NM_202494.2
HIST1H4A	mRNA	6.626317	38.27467667	5.149888088	NM_003538.3
MAGED2	mRNA	1.944765	14.13979035	5.141255716	NM_201222.2
HIST3H2BB	mRNA	2.544975	17.18203506	5.128960504	NM_175055.2
RPL21P16	ncRNA	1.129434	9.88453589	5.111468955	NM_001139505.1
MIR1269A	Precursor_m	0	4.109142777	5.109142777	NR_031673.1
RUSC1	mRNA	8.050763	45.0012842	5.082586589	NR_103478.1
SNORD38A	snoRNA	0	4.051267527	5.051267527	NR_001456.1
RNU6-45P	ncRNA	0	4.03233637	5.03233637	NR_046496.1
MIR19A	miRNA	2.004057	14.03121924	5.003640311	NR_029489.1
TRP-AGG2-3	tRNA	0	3.994999922	4.994999922	TRP1
EFNA4	mRNA	2.876148	18.19202828	4.951314357	NM_182690.2
TRV-CAC1-6	tRNA	0	3.940273896	4.940273896	TRV-CAC1-6
HIST1H1A	mRNA	92.16095	457.2407849	4.918807641	NM_005325.3
MANF	mRNA	15.69948	81.09476477	4.916005856	NM_006010.5
MIR6853	miRNA	0	3.887026951	4.887026951	NR_106912.1
TRI-AAT8-1	tRNA	0	3.887026951	4.887026951	TRNAI5
TRNAI-AAU	tRNA	0	3.887026951	4.887026951	TRNAI-AAU

HIST1H2AK	mRNA	4.286939	24.69952126	4.860945574	NM_003510.2
HIST1H2BI	mRNA	8.649087	45.74594876	4.844598201	NM_003525.2
MIR4512	Precursor_m	0	3.735584343	4.735584343	NR_039737.1
TK1	mRNA	5.945606	31.13540864	4.626724985	NM_003258.4
MIR3190	miRNA	0	3.59549993	4.59549993	NR_036158.1
MIR4435-1	Precursor_m	0	3.59549993	4.59549993	NR_039634.1
MIR1248	Precursor_m	9.301848	46.1309425	4.574998774	NR_031650.1
H2AFX	mRNA	30.82526	143.6395455	4.544803449	NM_002105.2
RPL27A	mRNA	8.521451	42.03470301	4.519763013	NM_032650.1
MIR3177	miRNA	0	3.50780481	4.50780481	NR_036138.1
RPL36	mRNA	36.41636	166.5848012	4.478917803	NM_033643.2
MIRLET7F2	miRNA	0	3.465542101	4.465542101	NR_029484.1
RPL12	mRNA	171.0553	766.6041669	4.461379258	NM_000976.3
ZMAT5	mRNA	4.136156	21.85841196	4.450490411	NM_019103.2
MIRLET7A1	miRNA	2.054158	12.58424975	4.44778862	NR_029476.1
SPON2	mRNA	4.106415	21.5630167	4.418562948	NM_012445.3
MRPL54	mRNA	2.637763	15.00529666	4.399763148	NM_172251.2
ATP5H	mRNA	66.5785	295.5015376	4.387512773	NM_006356.2
DAZAP2	mRNA	40.89349	182.0657582	4.369790155	NM_014764.3
HMGN2	mRNA	46.73757	207.0745008	4.358715401	NM_005517.3
HIST1H2AI	mRNA	10.86207	50.29100115	4.323949274	NM_003509.2
HIST2H2AA4	mRNA	1.230956	8.618426798	4.311347253	NM_001040874.1
GTF2A2	mRNA	12.02434	54.84931157	4.288072218	NM_004492.2
RPS7P10	ncRNA	0.248237	4.345015021	4.282052686	RPS7P10
SNORD24	snoRNA	13.14661	59.44559884	4.272796824	NR_002447.1
MIR3618	Precursor_m	0	3.2686363	4.2686363	NR_037412.1
FAM222A	mRNA	3.140114	16.67212706	4.268511844	NM_032829.2
RNU6-758P	ncRNA	0	3.231910049	4.231910049	RNU6-758P
MIR2276	Precursor_m	0	3.231910049	4.231910049	NR_031753.1
RPS27P3	ncRNA	4.749499	23.27722498	4.222494317	RPS27P3
MIR4691	miRNA	9.666626	43.99199914	4.218015814	NR_039840.1
TAGLN	mRNA	5.171676	24.75514403	4.173119776	NM_003186.3
HMGN2P6	ncRNA	1.37632	8.793014904	4.12108363	HMGN2P6
BIN1	mRNA	26.71607	113.1636294	4.119041284	NM_139351.2
IGFBP6	mRNA	0.51035	5.210869464	4.112205847	NM_002178.2
MGST3	mRNA	3.786467	18.6520997	4.105763475	NM_004528.3
RPS27P4	ncRNA	0.476327	5.002434685	4.065790729	RPS27P4
HIST1H2AM	mRNA	21.93352	91.84398178	4.048397206	NM_003514.2
MIR613	miRNA	0	3.027789415	4.027789415	NR_030344.1
HIST1H2BB	mRNA	17.1577	72.07684314	4.024564674	NM_021062.2
LOC1079871	lncRNA	0	3.011937114	4.011937114	
ASNA1	mRNA	11.0411	47.30329595	4.01153518	NM_004317.2
BAD	mRNA	0.805552	6.227499878	4.002930435	NM_032989.2
BTF3	mRNA	38.41884	156.7165862	4.001045635	NM_001207.4
MIR578	Precursor_m	0	2.996249942	3.996249942	NR_030304.1
POLR2I	mRNA	2.449913	12.69970159	3.97102772	NM_006233.4

CRIP1	mRNA	1.369439	8.389499836	3.962752713	NM_001311.4
EEF1G	mRNA	105.2456	418.8359996	3.951563038	NM_001404.4
MIR567	miRNA	0	2.935101984	3.935101984	NR_030292.1
RPL18	mRNA	72.81029	288.6128467	3.92374597	NR_073022.1
NPW	mRNA	1.455703	8.634862036	3.923464512	NM_001099456.2
MAD2L2	mRNA	3.138297	15.2309372	3.922129482	NM_006341.3
BCYRN1P1	ncRNA	0	2.920202989	3.920202989	BCYRN1P1
RPL21P75	ncRNA	3.761135	17.47109121	3.879556613	RPL21P75
RPL19	mRNA	172.0856	670.0069434	3.876734109	NM_000981.3
MIR593	Precursor_m	0	2.876399944	3.876399944	NR_030324.1
RPS25	mRNA	181.0331	703.3148643	3.869157735	NM_001028.2
HIST1H4E	mRNA	24.41015	97.27877794	3.867696922	NM_003545.3
RNU6-1053P	ncRNA	0	2.847920736	3.847920736	RNU6-1053P
PDRG1	mRNA	9.455411	39.07127382	3.832587134	NM_030815.2
HIST1H4B	mRNA	47.4125	184.5085678	3.831831923	NM_003544.2
RAB3IL1	mRNA	3.3933	15.81838149	3.828189101	NM_013401.3
HIST1H2AB	mRNA	17.57016	69.95018731	3.820656185	NM_003513.2
PXMP2	mRNA	5.32321	22.84288357	3.770692868	NM_018663.2
GORASP2	mRNA	30.55068	117.89711	3.768448998	NM_015530.4
RNU6-817P	ncRNA	0	2.765769177	3.765769177	RNU6-817P
SNORD124	snoRNA	0	2.765769177	3.765769177	NR_102369.1
JMJD8	mRNA	7.378828	30.53988715	3.764236108	NM_001005920.2
LOC1019280(	lncRNA	3.107946	14.44999972	3.761003514	NR_120586.1
MIR4724	miRNA	1.846434	9.695730148	3.757589016	NR_039877.1
MZT2B	mRNA	5.725526	23.99930271	3.717077525	NM_025029.3
MIR1825	miRNA	0	2.713584853	3.713584853	NR_031726.1
MIR4657	miRNA	0	2.713584853	3.713584853	NR_039801.1
MIR130B	miRNA	4.008113	17.53902405	3.701797965	NR_029845.1
SNORA94	snoRNA	2.13419	10.58415564	3.696060123	
RNU6-945P	ncRNA	0	2.688224247	3.688224247	RNU6-945P
LMNB2	mRNA	27.49493	104.0867809	3.687911136	NM_032737.3
FBLN5	mRNA	9.173635	36.475368	3.683576944	NM_006329.3
MRPL53	mRNA	7.275797	29.42271698	3.67610733	NM_053050.4
HMGN2P5	ncRNA	1.718961	8.966183925	3.665438655	HMGN2P5
HIST2H4A	mRNA	1.659926	8.716363466	3.652870174	NM_003548.2
HIST1H3I	mRNA	39.61898	147.1366009	3.646979589	NM_003533.2
C9orf16	mRNA	2.765767	12.70779779	3.640107885	NM_024112.3
MT1F	mRNA	2.721866	12.50608671	3.628848351	NM_005949.3
LOC1079859(	lncRNA	2.528195	11.80061515	3.62809214	
PPIAP22	ncRNA	6.662134	26.62613462	3.605540304	PPIAP22
HIST1H1B	mRNA	125.8497	456.0368265	3.602979518	NM_005322.2
MIR122	miRNA	7.733301	30.45599941	3.601845255	NR_029667.1
CTSH	mRNA	4.376168	18.35718972	3.600555276	NM_148979.2
MRPL22	mRNA	10.82431	41.56411517	3.599713516	NM_014180.3
RPL10	mRNA	39.9103	146.1909742	3.597895094	NM_006013
LOC1053780:	lncRNA	4.301902	18.07162268	3.597128593	XR_955078.1

MTFP1	mRNA	4.757675	19.69366159	3.594100617	NM_016498.4
HIST2H3C	mRNA	2.917148	13.04875714	3.586476297	NM_021059.2
TIMM10	mRNA	22.43116	83.00590342	3.58522126	NM_012456.2
RPS7P1	ncRNA	5.097468	20.81884745	3.578345705	RPS7P1
RPS25P6	ncRNA	5.061581	20.67227886	3.575351101	NM_001089589.2
EFNA1	mRNA	4.092576	17.17390771	3.56870653	NM_182685.1
RNU7-103P	ncRNA	0	2.568214236	3.568214236	
EFNA3	mRNA	0.276654	3.551111042	3.564873487	NM_004952.4
LOC10272413	mRNA	0.314613	3.671218818	3.553304452	XR_932558.1
LOC729998	ncRNA	48.51616	174.7826474	3.550005566	LOC729998
RPL12P38	ncRNA	0	2.539357896	3.539357896	RPL12P38
VPS4A	mRNA	13.14057	48.97546779	3.534190384	NM_013245.2
AKR1B1	mRNA	4.895015	19.78799961	3.526369184	NM_001628.2
RASD1	mRNA	4.230532	17.44269989	3.52597045	NM_016084.4
TECRP1	ncRNA	0.578636	4.557675967	3.520555495	TECRP1
TNFRSF12A	mRNA	13.9993	51.51345495	3.501061215	NM_016639.2
C19orf70	mRNA	1.643327	8.236963476	3.494446666	NM_205767
HIST1H1C	mRNA	122.576	430.2811391	3.490007472	NM_005319.3
HIST1H3D	mRNA	25.87213	92.6007148	3.483188912	NM_003530.4
RPL3P4	ncRNA	3.08767	13.21102271	3.476558353	RPL3P4
HIST3H2A	mRNA	4.638422	18.55741899	3.468598213	NM_033445.2
SNORA71D	snoRNA	10.71735	39.60260792	3.465170812	NR_003018.2
BCL2L12	mRNA	6.955879	26.56415533	3.464627108	NR_104200.1
HIST1H3H	mRNA	16.32904	58.98748299	3.461674334	NM_003536.2
CHCHD10	mRNA	8.661083	32.31461795	3.448331686	NR_125755.1
NDUFB1	mRNA	19.95468	71.22514147	3.446731	NM_004545.3
HMGN2P41	ncRNA	28.07694	99.13220912	3.44369888	HMGN2P41
MIR18A	miRNA	6.943633	26.33323892	3.440898966	NR_029488.1
RNA5SP284	ncRNA	0	2.437627071	3.437627071	RNA5SP284
MIR4673	Precursor_m	0	2.437627071	3.437627071	NR_039820.1
HIST1H2BH	mRNA	20.87991	74.10959855	3.43281063	NM_003524.2
MIR6783	Precursor_m	2.567698	11.23593728	3.429645238	NR_106841.1
PNKP	mRNA	5.633717	21.72759981	3.426073348	NM_007254.3
CLDN6	mRNA	11.15543	40.62652475	3.424521499	NM_021195.4
S100A11	mRNA	9.406246	34.62333266	3.423264527	NM_005620.1
HIST1H1D	mRNA	16.28522	58.12030775	3.420281376	NM_005320.2
RBP2	mRNA	6.687957	25.29392392	3.420144649	NM_004164.2
PNKD	mRNA	6.543754	24.75514403	3.414101985	NM_022572.4
MRPS26	mRNA	17.87256	63.25865132	3.404872078	NM_030811.3
CD320	mRNA	8.93255	32.76474756	3.399403693	NM_016579.3
RNU4-39P	ncRNA	1.293958	6.794645537	3.397902648	RNU4-39P
MIR4690	Precursor_m	0	2.396999953	3.396999953	NR_039839.1
KXD1	mRNA	10.79835	38.86667208	3.379003761	NM_024069.3
GSTO1	mRNA	42.75447	146.4052195	3.368917663	NM_004832.2
MIR6815	miRNA	0	2.357704872	3.357704872	NR_106873.1
MIR6850	miRNA	0	2.357704872	3.357704872	NR_106909.1

MIR6890	miRNA	0	2.357704872	3.357704872	NR_106950.1
MIR6733	Precursor_m	0	2.357704872	3.357704872	NR_106791.1
MRPL12	mRNA	7.285684	26.78027534	3.35280411	NM_002949.3
SNORD57	snoRNA	2.282398	9.987499805	3.34740031	NR_002738.1
ID3	mRNA	23.07819	79.51307351	3.343817103	NM_002167.4
MRTO4	mRNA	6.760957	24.93604485	3.341861664	NM_016183.3
COMMD4	mRNA	4.337465	16.83152358	3.340822349	NR_104312.1
HAX1	mRNA	18.32134	63.52559876	3.339602285	NM_006118.3
SNRPB	mRNA	57.29551	192.841037	3.325145117	NM_198216.1
MT1A	mRNA	0.829963	5.084545355	3.324955618	NM_005946.2
MIR6742	miRNA	0	2.319677374	3.319677374	NR_106800.1
RNU7-16P	ncRNA	0	2.319677374	3.319677374	U7.16
MIR6872	Precursor_m	0	2.319677374	3.319677374	NR_106932.1
SNORD59B	snoRNA	2.191102	9.587999813	3.317975988	NR_003046.1
SGTA	mRNA	19.49239	66.901493	3.313497798	NM_003021.3
SNORD19	snoRNA	2.162272	9.461841921	3.308331123	NR_003047.1
YDJC	mRNA	3.228143	12.97823179	3.305997949	NM_001017965.1
HIST1H2BJ	mRNA	37.58127	126.1788749	3.296388909	NM_021058.3
MRPL49	mRNA	6.804827	24.71157496	3.294317284	NR_037567.1
RAD23A	mRNA	35.37529	118.7837868	3.292999026	NR_072976.1
PTMAP2	ncRNA	5.182274	19.33513655	3.289264614	V9HVW6_HUMAN
MIR3131	Precursor_m	0	2.282857098	3.282857098	NR_036081.1
SNORD49A	snoRNA	55.54906	184.3326725	3.277378204	NR_002744.1
TRMT112	mRNA	14.14628	48.59341369	3.274296067	NM_016404.2
HIST1H2BL	mRNA	15.96167	54.28966781	3.259682988	NM_003519.3
SCYL1	mRNA	9.69052	33.82207561	3.257285493	NM_020680.3
RAMP2	mRNA	0.209341	2.931363	3.250830944	NM_005854.2
SNRPG	mRNA	27.62884	92.02187868	3.249237194	NM_003096.2
NFKBID	mRNA	3.38133	13.23595934	3.249232204	NM_139239.1
MIR6513	miRNA	0	2.247187456	3.247187456	NR_106768.1
MIR6738	miRNA	0	2.247187456	3.247187456	NR_106796.1
MIR6877	miRNA	0	2.247187456	3.247187456	NR_106937.1
TCTN3	mRNA	8.27963	29.03704215	3.236879398	NM_015631.5
P3H4	mRNA	3.999752	15.1521068	3.230581877	NM_006455.2
STRA13	mRNA	5.005781	18.39994742	3.230212082	NM_144998.3
ACY3	mRNA	7.200973	25.49066006	3.230185054	NM_080658.1
HIST1H2BE	mRNA	44.57759	146.134342	3.228216633	NM_003523.2
TIGD5	mRNA	0.953838	5.306790941	3.227898705	NM_032862.4
NDUFS6	mRNA	17.75756	59.26648236	3.212917386	NM_004553.4
MIR7109	miRNA	0	2.212615341	3.212615341	NR_106959.1
MIR4658	Precursor_m	0	2.212615341	3.212615341	NR_039802.1
LOC1053718[illegible]	lncRNA	0.270729	3.080164685	3.210884387	XR_934846.1
SNORD14D	snoRNA	7.555524	26.44965466	3.208412975	NR_001454.2
PCBD1	mRNA	16.49013	55.11441069	3.208347718	NM_001289797.1
SERPINC1	mRNA	6.610549	23.38602787	3.204240507	NM_000488.3
RPL35	mRNA	296.156	947.0234163	3.190322149	NM_007209.3

SDHB	mRNA	7.41136	25.82024234	3.188573918	NM_003000.2
HIST1H3F	mRNA	15.86903	52.77514918	3.187802765	NM_021018.2
TMEM109	mRNA	65.16877	209.4664783	3.180752529	NM_024092.2
MIR6754	miRNA	0	2.179090867	3.179090867	NR_106812.1
MIR6860	miRNA	0	2.179090867	3.179090867	NR_106920.1
MIR6852	miRNA	0	2.179090867	3.179090867	NR_106911.1
MIR4641	miRNA	0	2.179090867	3.179090867	NR_039784.2
MIR1249	Precursor_m	0	2.179090867	3.179090867	NR_031651.1
MIR6816	Precursor_m	0	2.179090867	3.179090867	NR_106874.1
HIST2H2AB	mRNA	10.6376	35.9549993	3.175481608	NM_175065.2
APOA1	mRNA	57.45616	184.6100208	3.175200434	NM_000039.1
TAGLN2	mRNA	23.47609	76.70997979	3.174934015	NM_003564.2
RPS27AP16	ncRNA	15.89291	52.57636261	3.171530227	RPS27AP16
SNRPD2	mRNA	32.35805	104.5779726	3.164992677	NM_177542.2
LIPA	mRNA	8.029283	27.48351738	3.154571489	NR_110233.1
MRPS17	mRNA	8.973797	30.43290043	3.151548174	NM_015969.2
CKS1BP3	ncRNA	0.455215	3.585540096	3.151108111	CKS1BP3
MIR6829	miRNA	0	2.146567122	3.146567122	NR_106887.1
RN7SKP80	ncRNA	0	2.146567122	3.146567122	RN7SKP80
MIR611	Precursor_m	0	2.146567122	3.146567122	NR_030342.1
MIR3658	miRNA	5.869023	20.54571388	3.136648867	NR_037431.1
FAM173A	mRNA	0.430754	3.487116577	3.136190138	NM_023933.2
RHBDD2	mRNA	7.612854	25.9611177	3.130334784	NM_020684.2
CADM4	mRNA	5.890601	20.55515585	3.128197002	NM_145296.1
CCND3	mRNA	1.080718	5.507999892	3.127766991	NM_001760.4
AMT	mRNA	3.582183	13.32392345	3.126004158	NR_028435.1
GPX1	mRNA	6.528854	22.49087023	3.12011224	NM_201397.1
NT5C	mRNA	3.334286	12.50608671	3.116104427	NR_045513.1
MIR6804	miRNA	0	2.114999959	3.114999959	NR_106862.1
RNY5P8	ncRNA	0	2.114999959	3.114999959	RNY5P8
MIR6780A	Precursor_m	0	2.114999959	3.114999959	NR_106838.1
MIR6870	miRNA	5.477755	19.17599963	3.114659264	NR_106930.1
TAB1	mRNA	3.36413	12.57978884	3.111682981	NM_153497.2
CDC34	mRNA	20.89453	67.11599869	3.111096726	NM_004359.1
HM13	mRNA	54.77755	171.9341875	3.100426381	NM_178582.2
HIST1H2BM	mRNA	9.948389	32.89156886	3.095575839	NM_003521.2
LOC1079867(	lncRNA	0	2.091927232	3.091927232	
LOC10192741	lncRNA	0	2.084347785	3.084347785	NR_110050.1
MIR4743	miRNA	0	2.084347785	3.084347785	NR_039897.2
MIR4499	Precursor_m	0	2.084347785	3.084347785	NR_039721.1
UFD1L	mRNA	31.15224	97.92343051	3.07672015	NM_005659.6
HMGCL	mRNA	2.350529	9.301790863	3.074675746	NM_001166059.1
ACAA2	mRNA	16.73916	53.54208789	3.074671702	NM_006111.2
RPL24P8	ncRNA	1.79272	7.583236216	3.07343263	RPL24P8
GLTSCR2	mRNA	19.3269	61.42115517	3.070864225	NM_015710.4
SMAGP	mRNA	6.34956	21.56638412	3.070440313	NM_001033873.1

PRPF31	mRNA	11.44231	37.15734238	3.066741005	NM_015629.3
TAX1BP3	mRNA	6.059989	20.59028159	3.058118191	NM_014604.3
H1FX	mRNA	15.37552	49.05340316	3.056599929	NM_006026.3
MIR6830	Precursor_m	0	2.054571388	3.054571388	NR_106888.1
MIR6810	Precursor_m	0	2.054571388	3.054571388	NR_106868.1
SNORD41	snoRNA	0	2.054571388	3.054571388	NR_002751.1
RNU6-103P	ncRNA	1.535819	6.720560616	3.04460221	RNU6-103P
RPS10P19	ncRNA	10.83054	34.99802213	3.042804083	RPS10P19
EMILIN2	mRNA	1.639637	7.031359304	3.042599547	NM_032048.2
ZDHHC12	mRNA	3.743341	13.43193596	3.042567956	NM_032799.4
MLLT1	mRNA	10.03335	32.56623529	3.042252133	NM_005934.3
LOXL4	mRNA	4.432119	15.5155378	3.040348969	NM_032211.6
CAT	mRNA	6.930551	23.07372998	3.035568438	NM_001752.3
MRPL28	mRNA	7.351724	24.34847321	3.035118709	NM_006428.4
BTG2	mRNA	7.392545	24.44727828	3.032128737	NM_006763.2
C10orf35	mRNA	3.556984	12.80771775	3.030012493	NM_145306.2
RPL24P4	ncRNA	15.56037	49.15787433	3.028790132	RPL24P4
MIR6748	miRNA	0	2.025633763	3.025633763	NR_106806.1
MIR20A	miRNA	0	2.025633763	3.025633763	NR_029492.1
MIR503	miRNA	0	2.025633763	3.025633763	NR_030228.1
TRNF	tRNA	0	2.025633763	3.025633763	TRNF
RABAC1	mRNA	3.461778	12.47509269	3.020117076	NM_006423.2
CKS1B	mRNA	12.80514	40.62447973	3.015143274	NR_024163.1
AURKAIP1	mRNA	8.299629	27.02072675	3.013101639	NM_017900.2
GADD45GIP1	mRNA	7.968949	26.01200473	3.01172466	NM_052850.3
CCDC107	mRNA	1.306301	5.944864749	3.011257042	NM_174923.2
RASSF7	mRNA	1.045494	5.157223728	3.010140184	NM_003475.3
HIST1H2AG	mRNA	15.83929	49.67277011	3.009198451	NM_021064.4
PIPOX	mRNA	1.635151	6.916716283	3.004274535	NM_016518.2
RPS25P1	ncRNA	1.022817	5.072489528	3.001996006	RPS25P1
TRA-TGC1-1	tRNA	0	1.997499961	2.997499961	TRNAA19
TRC-GCA3-1	tRNA	0	1.997499961	2.997499961	TRNAC8
RNF126	mRNA	19.02275	58.92261703	2.992726747	NM_194460.2
CTDSP1	mRNA	9.086976	29.1771269	2.991692154	NM_182642.2
RN7SL11P	ncRNA	0	1.990588196	2.990588196	RN7SL11P
AGT	mRNA	33.07004	100.8521033	2.989491925	NM_000029.3
KRT8	mRNA	208.2821	624.1787878	2.987254278	NR_045962.1
HIST1H3B	mRNA	67.89167	203.8465638	2.973459246	NM_003537.3
RPL13	mRNA	19.06428	58.64235759	2.972564688	NM_033251.2
FAM98C	mRNA	1.804437	7.331999857	2.971006313	NM_174905.3
TRK-CTT3-1	tRNA	0	1.970136948	2.970136948	TRNAK15
TRF-GAA1-6	tRNA	0	1.970136948	2.970136948	TRNAF7
TRM-CAT2-1	tRNA	0	1.970136948	2.970136948	TRNAM9
TRR-CCT5-1	tRNA	0	1.970136948	2.970136948	TRR-CCT5-1
TRR-CCG2-1	tRNA	0	1.970136948	2.970136948	TRR4
RPL29P4	ncRNA	12.79537	39.96219769	2.96927213	RPL29P4

NABP2	mRNA	6.298856	20.67227886	2.969270704	NM_024068.3
MTCH1	mRNA	6.770077	22.03762257	2.964915771	NR_130739.1
LOC1002870	ncRNA	3.641048	12.74623313	2.961881028	LOC100287046
S100A4	mRNA	4.079179	14.02499973	2.958155042	NM_019554.2
RTFDC1	mRNA	18.50633	56.60917052	2.953357757	NM_016407.4
PYCR1	mRNA	10.59736	33.1694127	2.946309938	NM_153824.2
MIR4655	miRNA	0	1.943513476	2.943513476	NR_039799.1
RPL8	mRNA	181.1794	534.6672589	2.94032794	NM_033301.1
RPL18AP3	ncRNA	9.217376	29.04057636	2.940145861	NR_001593.1
WDR34	mRNA	7.68332	24.5237619	2.939401371	NM_052844.3
CIAPIN1	mRNA	6.85347	22.08052203	2.938894851	NM_020313
RPL34P35	ncRNA	0	1.936969659	2.936969659	RPL34P35
EGFL7	mRNA	2.06836	8.005580415	2.934981792	NR_045110.1
SCARNA28	ncRNA	3.388302	11.86144307	2.930847258	
TADA3	mRNA	9.352265	29.2825445	2.925209658	NR_103488.1
C9orf116	mRNA	0.227608	2.589556736	2.924026282	NM_144654.2
RPS2P5	ncRNA	7.257593	23.13823299	2.923155923	RPS2P5
MIR1226	miRNA	0	1.917599963	2.917599963	NR_031595.1
MIR6750	miRNA	0	1.917599963	2.917599963	NR_106808.1
MIR4525	miRNA	0	1.917599963	2.917599963	NR_039751.1
MIR4661	miRNA	0	1.917599963	2.917599963	NR_039805.1
NMB	mRNA	1.595463	6.562660066	2.913800416	NM_205858.1
CCDC51	mRNA	1.862606	7.335486719	2.91185257	NM_024661.4
NCAPH2	mRNA	3.735922	12.74301345	2.901866811	NM_152299.3
ARHGDIA	mRNA	27.09895	80.53412541	2.901679066	NR_125441.1
WBP1	mRNA	3.359212	11.64201122	2.900068012	NM_012477.3
FARSA	mRNA	14.98771	45.32697159	2.897662349	NM_004461.2
CD81	mRNA	12.65572	38.51422329	2.893601628	NM_004356.3
PKDCC	mRNA	5.190835	16.90646908	2.892415628	NM_138370.2
MIR4254	Precursor_m	0	1.892368384	2.892368384	NR_036216.1
MIR4732	Precursor_m	0	1.892368384	2.892368384	NR_039885.1
GOLGA2	mRNA	12.85557	39.06466545	2.891592534	NM_004486.4
IL10RB	mRNA	5.028026	16.42818938	2.891193461	NM_000628.4
IHH	mRNA	2.8552	10.13403455	2.888055913	NM_002181.3
CLPP	mRNA	3.283798	11.37065139	2.887776865	NM_006012.2
NOP16	mRNA	12.40246	37.66455776	2.884884251	NM_016391.6
MRPL51	mRNA	35.43954	104.0942373	2.884071654	NM_016497.3
PSMC3	mRNA	20.91849	62.17905842	2.882454746	NM_002804.4
SLC1A7	mRNA	0.857985	4.355147844	2.882234788	NR_109858.1
ISOC2	mRNA	11.37688	34.66430702	2.881527428	NM_024710.2
MST1P2	miscRNA	1.184492	5.292314009	2.880447547	NR_027504.1
UCK1	mRNA	5.189452	16.77899967	2.872467444	NM_031432.2
PRDX5	mRNA	15.07139	45.13246321	2.870470871	NM_181652.2
RPS6KA1	mRNA	3.75001	12.63314136	2.870128705	NM_002953.3
SLC39A5	mRNA	3.386715	11.58643537	2.869216548	NM_173596.2
CAPN1	mRNA	9.338302	28.65127312	2.868098916	NR_040008.1

MIR5704	miRNA	0	1.867792171	2.867792171	NR_049890.1
MIR7703	miRNA	0	1.867792171	2.867792171	NR_106990.1
MIR26B	Precursor_m	0	1.867792171	2.867792171	NR_029500.1
RHOG	mRNA	2.192564	8.155236665	2.867675479	NM_001665.3
SZRD1	mRNA	11.86895	35.89771571	2.867188403	NR_073500.1
SNORD30	snoRNA	9.390437	28.76399944	2.864556998	NR_002561.1
CDIPT	mRNA	6.466395	20.37324199	2.862591943	NM_145752.1
TMEM208	mRNA	4.148903	13.7347308	2.861722157	NM_014187.3
SLC25A1	mRNA	22.0935	65.0227233	2.858930613	NR_046298.2
IL32	mRNA	9.915486	30.19872829	2.85820797	NM_004221.4
APOC3	mRNA	2.466531	8.904427594	2.857157961	NM_000040.1
HSPB1	mRNA	30.96122	90.26829255	2.855594416	NM_001540.3
IER5	mRNA	2.3857	8.658676176	2.852785339	NM_016545.4
CCT7	mRNA	55.72106	160.6650559	2.850176869	NR_029402.1
RPS6KB2	mRNA	24.71038	72.21245916	2.847583988	NM_003952.2
SNORA70	snoRNA	6.086394	19.17599963	2.847146003	NR_000011.1
TMEM160	mRNA	1.898802	7.25142843	2.84649641	NM_017854.1
SNORA65	snoRNA	6.041642	19.03499963	2.845217198	NR_002449.2
RPL37	mRNA	134.0829	383.2757123	2.844740256	NM_000997.4
MIR301B	miRNA	0	1.843846118	2.843846118	NR_030622.1
MIR92A1	miRNA	0	1.843846118	2.843846118	NR_029508.1
MIR1286	Precursor_m	0	1.843846118	2.843846118	NR_031618.1
TMEM223	mRNA	5.787596	18.26529313	2.838308753	NM_001080501.2
HIST1H2AL	mRNA	21.67792	63.34199876	2.837208495	NM_003511.2
RHOD	mRNA	6.38185	19.92916113	2.83521878	NM_014578.3
MIR7-1	Precursor_m	16.43327	48.37581724	2.832275952	NR_029605.1
C14orf80	mRNA	1.729817	6.728420921	2.83111281	NM_173608.2
AES	mRNA	18.34655	53.75090804	2.830009181	NM_198970.1
YY1	mRNA	22.26476	64.73948592	2.825711001	NM_003403.4
MED26	mRNA	4.438633	14.36393188	2.824961989	NM_004831.3
TRIP6	mRNA	9.942501	29.88041038	2.822061587	NM_003302.2
MIR5088	miRNA	0	1.820506294	2.820506294	NR_049811.2
MIR101-2	Precursor_m	0	1.820506294	2.820506294	NR_029836.1
LYPLA2	mRNA	9.408358	28.33857769	2.818751832	NM_007260.2
THOC6	mRNA	4.744816	15.19225322	2.818585201	NM_024339.3
GCHFR	mRNA	5.301053	16.74201931	2.815722812	NM_005258.2
HIST2H2AA3	mRNA	1.846434	7.002471773	2.811402272	NM_003516.2
COX7B	mRNA	17.65855	51.4093411	2.808864281	NM_001866.2
AGPAT2	mRNA	17.62224	51.28465016	2.80764634	NM_006412.3
SYNPR-AS1	lncRNA	0	1.804193513	2.804193513	NR_046677.1
DCTN3	mRNA	2.583847	9.045282842	2.802933185	NM_024348.3
JTB	mRNA	5.846651	18.18308732	2.801820599	NM_006694.3
FABP5	mRNA	11.01546	32.65953425	2.801352561	NM_001444.2
MIR6781	miRNA	2.567698	8.988749825	2.799774753	NR_106839.1
DNAL4	mRNA	0.77359	3.965447132	2.799658724	NM_005740.2
SEPW1	mRNA	15.19514	44.32808133	2.798868975	NM_003009.2

FOXP4	mRNA	12.09913	35.65884953	2.798572169	NM_138457.2
MIR7107	miRNA	0	1.797749965	2.797749965	NR_106957.1
MIR4492	miRNA	0	1.797749965	2.797749965	NR_039713.1
RN7SKP180	ncRNA	0	1.797749965	2.797749965	RN7SKP180
HIST1H3C	mRNA	109.9131	308.9466606	2.794499439	NM_003531.2
HIST2H2BA	miscRNA	0.957278	4.468194088	2.793775478	NR_027337.1
HIST1H2AC	mRNA	38.52487	109.3137341	2.790995472	NM_003512.3
NAT9	mRNA	2.008279	7.394032725	2.790310275	NR_130953.1
CPTP	mRNA	1.639051	6.361508983	2.789453396	NM_001029885.1
CCDC94	mRNA	4.234771	13.58929107	2.78699688	NM_018074.4
SNHG7	lncRNA	10.27079	30.40312441	2.78623971	NR_003672.2
USP5	mRNA	14.57416	42.36627934	2.784502236	NM_003481.2
SLC25A4	mRNA	1.264097	5.303769127	2.784230653	NM_001151.3
SNORD34	snoRNA	2.489889	8.716363466	2.78414714	NR_000019.1
TMSB10	mRNA	219.5648	611.3841789	2.776436762	NM_021103.3
LOC1053770:	lncRNA	0	1.775555521	2.775555521	XR_940727.1
MIR29B1	miRNA	0	1.775555521	2.775555521	NR_029517.1
MIR6785	miRNA	0	1.775555521	2.775555521	NR_106843.1
MIR4502	miRNA	0	1.775555521	2.775555521	NR_039724.1
LOC1079852(	lncRNA	4.956064	15.52342827	2.774219373	
POLR2L	mRNA	2.438727	8.537260107	2.773486054	NM_021128.4
MVB12A	mRNA	4.737111	14.905161	2.772329123	NM_138401.3
SYNGR2	mRNA	4.913915	15.3827024	2.770195888	NM_004710.3
EDF1	mRNA	58.27399	163.0299968	2.767318322	NM_153200.2
TPGS1	mRNA	0.295032	2.582046628	2.765991491	NM_033513.2
CORO1B	mRNA	21.94504	62.45599257	2.765564295	NM_020441.2
TSR3	mRNA	1.910845	7.04765767	2.764715508	NM_001001410.2
CFAP20	mRNA	5.163458	16.01274559	2.760260026	NM_013242.2
TUBB4B	mRNA	179.103	495.7315302	2.758041957	NM_006088.5
RAB1B	mRNA	79.14607	219.9998638	2.757463387	NM_030981.2
SNORD58C	snoRNA	10.95551	31.95999938	2.756887776	NR_003701.1
ATP6V0C	mRNA	33.28432	93.48299818	2.755865793	NM_001694.3
SLC25A10	mRNA	4.616086	14.47626376	2.755702932	NM_012140.4
FTLP3	ncRNA	0.184851	2.264881846	2.755520738	FTLP3
MIR1301	miRNA	0	1.753902405	2.753902405	NR_031570.1
MIR4761	miRNA	0	1.753902405	2.753902405	NR_039918.1
CDCA5	mRNA	12.43978	36.01083004	2.753827594	NM_080668.3
LOC1053702:	lncRNA	0	1.749635002	2.749635002	XR_942013.1
DDOST	mRNA	28.36787	79.68953748	2.747545164	NM_005216.4
TOMM40	mRNA	37.48098	104.7124508	2.747135368	NM_006114.2
IDH3B	mRNA	16.60246	47.29216124	2.74348901	NM_174856.1
RPL10A	mRNA	42.51728	118.2749669	2.740864665	NM_007104.4
GAMT	mRNA	12.44804	35.85337409	2.740428044	NM_138924.2
GRK2	mRNA	30.10857	84.22907501	2.73973009	NM_001619.3
NARF	mRNA	6.479827	19.49208753	2.739647428	NM_031968.2
LOC1079845(	lncRNA	52.6868	146.0157223	2.738395879	

MKNK2	mRNA	14.06705	40.20120397	2.734523881	NM_199054.2
MRPL41	mRNA	3.809035	12.14374148	2.733134956	NM_032477.2
MIR378H	Precursor_m	0	1.732771051	2.732771051	NR_039667.1
MIR762	Precursor_m	0	1.732771051	2.732771051	NR_031576.1
UPF1	mRNA	28.2442	78.88703598	2.731722421	NM_002911.3
NDUFB9	mRNA	19.65592	55.40770406	2.730824803	NM_005005.2
DNAJB2	mRNA	4.259502	13.34649574	2.727728776	NM_006736.5
TRIB3	mRNA	5.616516	17.0016402	2.720712871	NM_021158.4
CHCHD2	mRNA	39.9774	110.3215506	2.716656947	NM_016139.2
RPS3AP6	ncRNA	3.252218	10.54791598	2.715739561	RPS3AP6
MIR501	miRNA	0	1.712142824	2.712142824	NR_030225.1
MIR500A	miRNA	0	1.712142824	2.712142824	NR_030224.1
RNA5SP113	ncRNA	0	1.712142824	2.712142824	RNA5SP113
APEX2	mRNA	4.078901	12.76874438	2.710969187	NM_014481.3
ASRGL1	mRNA	5.162994	15.69594016	2.709063383	NM_025080.3
RPL29	mRNA	73.77236	201.5549313	2.708954813	NM_000992.2
SMOC1	mRNA	3.119684	10.1519998	2.707003694	NM_022137.5
FADD	mRNA	6.740033	19.94697209	2.706315557	NM_003824.3
HIST1H2AD	mRNA	31.25543	86.29199832	2.706273339	NM_021065.3
PTPN9	mRNA	9.580864	27.63385497	2.70619245	NM_002833.3
COX5A	mRNA	34.57388	95.25740074	2.705844702	NM_004255.3
RPL18A	mRNA	33.30736	91.7361532	2.703097696	NM_000980.3
FAM168A	mRNA	5.803946	17.38440212	2.70202068	NM_015159.2
GTF2IRD1	mRNA	5.019862	15.25768391	2.700673779	NM_016328.2
S100P	mRNA	23.1999	64.29599874	2.698192522	NM_005980.2
FIBP	mRNA	4.079983	12.69583423	2.696039365	NM_198897.1
RPL13A	mRNA	62.75531	170.6111906	2.691715895	NR_073024.1
OVCA2	mRNA	2.749059	9.088996105	2.691074097	NM_080822.2
RPSAP58	miscRNA	0.94106	4.220916167	2.68972388	NR_003662.3
C20orf27	mRNA	4.557046	13.91806424	2.684531476	NR_047675.1
GMPR2	mRNA	9.796612	27.97753419	2.683946861	NR_104265.1
MIR5706	miRNA	2.054158	7.19099986	2.681917413	NR_049892.1
NFKBIE	mRNA	0.827712	3.900581094	2.681265606	NM_004556.2
HMG20B	mRNA	13.76338	38.58143502	2.681055655	NM_006339.2
HIST1H2AH	mRNA	23.91535	65.7804561	2.680293237	NM_080596.2
ETNK2	mRNA	1.208328	4.917374904	2.679572091	NM_018208.3
DDX39A	mRNA	14.59402	40.76649555	2.678366372	NR_038336.1
HIST1H2BC	mRNA	68.28434	184.5361608	2.67789449	NM_003526.2
RPL10P16	ncRNA	13.48832	37.77488298	2.676286392	RPL10P16
NUP188	mRNA	17.91933	49.62358112	2.675759934	NM_015354.2
LOC10192928	lncRNA	0.782536	3.766714212	2.67411882	XR_432762
APEH	mRNA	6.48496	19.00052579	2.672095154	NM_001640.3
CPSF4	mRNA	6.56847	19.22067152	2.671698596	NM_006693.2
EIF2B2	mRNA	6.611696	19.3191041	2.669457999	NM_014239.3
RITA1	mRNA	6.883881	20.03532353	2.668143171	NM_032848.2
GRHPR	mRNA	31.97466	86.93968652	2.666887208	NM_012203.1

LOC10013039	ncRNA	6.118769	17.97749965	2.665840113	LOC100130394
PAK4	mRNA	10.61941	29.9468532	2.663376174	NM_005884.3
NDUFA11	mRNA	3.68235	11.47064163	2.663329918	NR_034166.2
AHSG	mRNA	54.60869	147.1007246	2.663265748	NM_001622.2
FBXO6	mRNA	0.105816	1.944764933	2.66297851	NM_018438.5
MCCD1P2	ncRNA	0	1.662658927	2.662658927	MCCD1P2
PSMB10	mRNA	1.470173	5.57552673	2.661970326	NM_002801.3
HIST2H2BF	mRNA	60.86394	163.5430599	2.659757009	NM_001161334.1
RPSAP12	ncRNA	0.317858	2.503636315	2.658583842	RPSAP12
NDUFA7	mRNA	6.811716	19.74730531	2.655921586	NM_005001.3
NDUFB11	mRNA	8.918409	25.33483936	2.655147566	NM_019056.6
MIR4511	miRNA	0	1.653103416	2.653103416	NR_039736.1
NOC4L	mRNA	2.145589	7.340367809	2.651448264	NM_024078
ITPK1	mRNA	4.434837	13.39249084	2.648191698	NM_014216.4
PIGQ	mRNA	1.706637	6.167155659	2.647992705	NM_148920.2
ARRDC1	mRNA	3.134624	9.944634999	2.647069156	NM_152285.2
PPIA	mRNA	59.01189	157.8196534	2.646469858	NM_203431.1
NSUN5P1	miscRNA	2.184113	7.416554879	2.643296674	NR_033322.3
MIR4257	Precursor_m	1.910845	6.689302195	2.641605035	NR_036211.1
TMEM18	mRNA	3.636363	11.24016927	2.640036995	NM_152834.2
NAXE	mRNA	3.341488	10.46031784	2.639721383	
TMEM141	mRNA	28.72948	77.44153695	2.638509845	NM_032928.3
OGFOD2	mRNA	1.329779	5.145178775	2.637666323	NM_024623
OTUB1	mRNA	8.210064	23.28021537	2.636269866	NR_003089.1
FSCN1	mRNA	5.759871	16.82014989	2.636167099	NM_003088.3
C14orf2	mRNA	8.207711	23.26729596	2.635540555	NM_004894.2
ESRRA	mRNA	2.488093	8.191622046	2.635142123	NM_004451.4
MIR425	Precursor_m	1.888881	6.612413664	2.635073432	NR_029948.1
MIR145	Precursor_m	0	1.63431815	2.63431815	NR_029686.1
POLD2	mRNA	11.46154	31.81975919	2.63368444	NM_006230.3
SNHG12	lncRNA	18.45685	50.2104997	2.632004025	NR_024127.1
ZYX	mRNA	34.70464	92.85873775	2.628754524	NM_003461.4
DALRD3	mRNA	1.416661	5.350834741	2.627938023	NM_018114.5
ITPKA	mRNA	2.399666	7.933823534	2.627853674	NM_002220.2
SNORD14C	snoRNA	9.337082	26.1490904	2.626378448	NR_001453.2
RPS19	mRNA	19.35618	52.41187249	2.623865499	NM_001022.3
UQCRC1	mRNA	13.61817	37.35584343	2.623847774	NM_003365.2
EIF3G	mRNA	50.88917	135.1423044	2.623713267	NM_003755.3
FAM64A	mRNA	5.088785	14.97325118	2.62338907	NM_019013.2
POLDIP2	mRNA	21.68402	58.50039144	2.623008581	NM_015584.4
IMPDH1	mRNA	7.184606	20.46197811	2.622237043	NM_183243.2
RPS7	mRNA	71.78267	189.8502985	2.622194298	NM_001011.3
EAPP	mRNA	6.199315	17.87743935	2.6221162	NM_018453.3
ERP29	mRNA	33.04515	88.2265336	2.620829316	NM_006817.3
MAB21L2	mRNA	1.721071	6.128840617	2.619865278	NM_006439.4
LMAN2	mRNA	19.01666	51.42035926	2.618836423	NM_006816.2

SF3B5	mRNA	7.358178	20.88024383	2.617824469	NM_031287.2
RBM38	mRNA	5.274081	15.42335782	2.617651488	NM_183425.2
GALT	mRNA	8.53828	23.95293903	2.616083711	NM_001258332.1
MIR4721	miRNA	0	1.615955025	2.615955025	NR_039872.1
MIR125B2	Precursor_m	0	1.615955025	2.615955025	NR_029694.1
MIR661	Precursor_m	0	1.615955025	2.615955025	NR_030383.1
EBP	mRNA	49.85373	132.0110951	2.615562465	NM_006579.2
COX5B	mRNA	17.24679	46.70590008	2.614481358	NM_001862.2
CBX6	mRNA	6.583968	18.82607263	2.614208267	NM_014292
ARFRP1	mRNA	2.435946	7.98088924	2.61380412	NR_051954.2
HINT2	mRNA	5.040879	14.77904879	2.612045237	NM_032593.2
FAM189B	mRNA	4.842404	14.2467458	2.609669842	NM_198264.1
PRELID3A	mRNA	2.636856	8.48908866	2.609145905	NM_006553.3
MOSPD3	mRNA	3.141854	9.803191829	2.608298253	NM_023948.4
OAZ1	mRNA	90.30643	237.1020614	2.607725109	NM_004152.3
HLA-A	mRNA	2.397155	7.856089232	2.606913228	NM_002116.7
C19orf60	mRNA	6.535958	18.63122691	2.605007587	NM_017967.2
LOC729973	ncRNA	2.827228	8.96941918	2.604866543	LOC729973
NAGLU	mRNA	2.598145	8.371843171	2.604632121	NM_000263.3
RAB34	mRNA	11.6317	31.84236631	2.59999586	NM_031934.5
ACOT8	mRNA	2.15695	7.196898957	2.596461208	NM_183386.1
MEPCE	mRNA	13.17915	35.78887059	2.594574603	NM_019606.5
HMGN2P3	ncRNA	1.623039	5.800148035	2.592469758	HMGN2P3
PGLS	mRNA	2.911208	9.137006541	2.591784319	NM_012088.2
UROD	mRNA	5.748261	16.47320955	2.589290554	NR_036510.1
SH3GL1	mRNA	13.84535	37.4271725	2.588499054	NM_003025.3
SHMT2	mRNA	33.18725	87.43914127	2.586904439	NR_029415.1
ZNF768	mRNA	6.737787	18.99100355	2.583555586	NM_024671.3
AK2P1	ncRNA	0	1.582613449	2.582613449	AK2P1
BCAP31	mRNA	12.88354	34.85111774	2.582275779	NM_005745.7
MIR3960	miRNA	0	1.58043953	2.58043953	NR_039767.1
HNF1B	mRNA	12.30854	33.32181027	2.578931803	NM_006481.1
TXN	mRNA	71.75354	186.5683374	2.578133414	NM_003329.3
PTRHD1	mRNA	1.436474	5.280104792	2.577537967	NM_001013663.1
DNLZ	mRNA	0.876441	3.835199925	2.576793217	NR_073565.1
RN7SL12P	ncRNA	1.699993	5.951172298	2.574514998	RN7SL12P
HIST1H2BD	mRNA	60.30883	156.7809741	2.573543892	NM_138720.2
RN7SKP255	ncRNA	6.847194	19.17599963	2.571110166	RN7SKP255
TRMT2A	mRNA	5.75653	16.3496626	2.567836101	NM_182984.4
HSD17B10	mRNA	18.77112	49.73214856	2.56597202	NM_004493.2
LOC1053784ε	lncRNA	0.197278	2.071836694	2.565683542	XR_959871.1
ATP5I	mRNA	45.02264	117.0261347	2.564523131	NR_033743.1
MCAT	mRNA	1.596237	5.657804649	2.564405092	NR_046423.1
PTGES2	mRNA	5.357907	15.30131244	2.563943172	NR_027812.1
SNORD96A	snoRNA	25.10638	65.91749871	2.56326255	NR_002592.1
HOXC-AS3	lncRNA	0	1.563260839	2.563260839	NR_047506.1

MIR191	miRNA	0	1.563260839	2.563260839	NR_029690.1
RNA5SP82	ncRNA	0	1.563260839	2.563260839	RNA5SP82
MYL9	mRNA	17.16089	45.53699159	2.562484565	NM_181526.2
EIF3I	mRNA	23.29259	61.23029583	2.561698651	NM_003757.2
SNORD126	snoRNA	1.659926	5.810908977	2.560563564	NR_003693.1
HIST1H2BK	mRNA	84.22293	217.1870518	2.560191817	NM_080593.2
CBX8	mRNA	2.444794	7.814281866	2.558725664	NM_020649.2
ETHE1	mRNA	1.226363	4.69561558	2.558260008	NM_014297.3
LDHA	mRNA	189.0004	485.0571185	2.558190365	NR_028500.1
TRIP13	mRNA	10.52004	28.46312078	2.557553701	NM_004237.3
CHID1	mRNA	4.319654	12.60152604	2.556844036	NM_023947.3
JUND	mRNA	25.70052	67.25764515	2.556416186	NM_005354.5
PSMB6	mRNA	26.94905	70.42561789	2.555565418	NM_002798.2
GMIP	mRNA	3.685622	10.96692998	2.553968539	NM_016573.3
SGSM3	mRNA	2.505707	7.949389934	2.552805658	NM_015705.5
IER3	mRNA	2.981842	9.162725628	2.552267143	NM_052815.1
TOMM5	mRNA	46.56394	120.3521255	2.551347358	NM_001134485.1
ALAS1	mRNA	19.30171	50.79450146	2.551237839	NM_199166.2
STX5	mRNA	9.217376	25.04557643	2.549145291	NM_003164.4
UCP2	mRNA	11.88067	31.80466524	2.546813272	NM_003355.2
ATP5G1	mRNA	13.96955	37.12200855	2.546637724	NM_005175.2
MIR222	Precursor_m	2.987866	9.15218164	2.545767765	NR_029636.1
CDT1	mRNA	9.153482	24.83919204	2.544860133	NM_030928.3
PCIF1	mRNA	6.037414	16.90815922	2.544707484	NM_022104.3
TGFB1I1	mRNA	0.364374	2.471407934	2.544322942	NM_015927.4
R3HCC1	mRNA	3.309405	9.956072808	2.542363133	NR_125897.1
SNAI1	mRNA	3.365502	10.09853696	2.542327826	NM_005985.3
TBX2	mRNA	9.723825	26.25353203	2.541400191	NM_005994.3
RPL12P4	ncRNA	1.846434	6.232969381	2.541063202	RPL12P4
CD2BP2	mRNA	4.416029	12.74562582	2.537952819	NM_006110.2
RPL7A	mRNA	221.7568	564.2914946	2.537707521	NM_000972.2
YIPF2	mRNA	9.79617	26.38472222	2.536521956	NM_024029
C19orf48	mRNA	17.9715	47.11804357	2.536332587	NM_199250.2
PLA2G15	mRNA	1.645677	5.709592163	2.536058247	NM_012320.3
RPL31	mRNA	93.44691	238.3412	2.534134786	NM_001099693.1
PTMS	mRNA	54.14104	138.7081449	2.533651019	NM_002824.4
CYB5R2	mRNA	1.83407	6.179765504	2.533376425	NR_126508.1
APOA1-AS	antisense_RI	0	1.532717554	2.532717554	NR_126362.1
F12	mRNA	6.489978	17.95120394	2.530208262	NM_000505.3
GRINA	mRNA	15.94895	41.87441991	2.52962081	NM_001009184.1
TMPRSS6	mRNA	1.389161	5.043155819	2.52940458	NM_153609.3
DNM1P38	ncRNA	0	1.528748948	2.528748948	DNM1P42
LOC388022	miscRNA	0.826385	3.616163723	2.527486033	NM_001013637.1
NR2C2AP	mRNA	8.814778	23.80468919	2.527279657	NM_176880.5
LRG1	mRNA	3.138938	9.453336894	2.525608318	NM_052972.2
TMUB1	mRNA	9.505337	25.51645111	2.524093379	NM_031434.3

SFN	mRNA	3.124195	9.405718448	2.523090877	NM_006142.3
DUS3L	mRNA	5.708638	15.92078709	2.522238925	NM_020175.2
MPP1	mRNA	3.88953	11.32492468	2.520676676	NM_002436.3
ACTG1	mRNA	252.0028	636.7024009	2.520535426	NR_037688.1
URB1-AS1	lncRNA	1.190816	4.516086868	2.517822629	NR_026845.1
ATPIF1	mRNA	26.29322	67.68878829	2.516697488	NM_178191.2
HRAS	mRNA	0.722486	3.333956769	2.516106137	NM_176795.3
PIK3R2	mRNA	25.86756	66.59062884	2.515697014	NR_073517.1
FAM234A	mRNA	9.014027	24.18672647	2.515144599	NR_104317.1
UCK2	mRNA	5.803748	16.10783969	2.514472776	NM_012474.4
TNFRSF1A	mRNA	43.26098	110.2878415	2.514355786	NM_001065.3
RNU6-1337P	ncRNA	0	1.513894707	2.513894707	RNU6-1337P
RFNG	mRNA	1.528258	5.349978014	2.511602377	NM_002917.1
YIPF3	mRNA	17.3988	45.17905671	2.509894626	NM_015388.3
PAQR4	mRNA	0.588373	2.986595001	2.509860317	NM_152341.4
CAMK2G	mRNA	5.439582	15.15872793	2.509281921	NM_172173.2
REXO1	mRNA	8.654302	23.21355935	2.508058937	NM_020695.3
ECHS1	mRNA	11.87053	31.27462693	2.507637748	NM_004092.3
CDK5	mRNA	3.581432	10.48813056	2.507541191	NM_004935.3
RPS15P4	ncRNA	7.887967	21.28535958	2.507362941	RPS15P4
TARS2	mRNA	5.715406	15.83102873	2.506330814	NR_073513.1
GLTPD2	mRNA	3.83004	11.10336468	2.505851897	NM_001014985.2
NEU4	mRNA	3.048001	9.137734628	2.504380461	NM_080741.3
TST	mRNA	5.058613	14.16696542	2.503372625	NM_003312.5
SAP30BP	mRNA	6.497407	17.76363156	2.502682774	NR_126036.1
JMJD7	mRNA	1.502338	5.259240404	2.501357023	NM_001114632.1
KLHDC3	mRNA	5.778909	15.95625526	2.501324943	NR_040101.1
TSTA3	mRNA	14.47551	37.69433861	2.500359283	NM_003313.3
RPS10	mRNA	14.86426	38.66517512	2.500285271	NM_001204091.1
RBM10	mRNA	11.86053	31.14015591	2.499131446	NM_152856.2
GSN	mRNA	23.66519	60.62414819	2.498426338	NM_198252.2
MIR615	miRNA	0	1.498124971	2.498124971	NR_030753.1
MIR621	miRNA	0	1.498124971	2.498124971	NR_030352.1
MIR92B	miRNA	0	1.498124971	2.498124971	NR_030281.1
RPS21P8	ncRNA	0	1.498124971	2.498124971	RPS21P8
LOC441259	ncRNA	0.210144	2.023043439	2.498085603	LOC441259
LOC10798581	lncRNA	1.493933	5.22981808	2.49798917	
SIRT6	mRNA	2.80397	8.501242978	2.49771787	NM_016539.2
NECTIN2	mRNA	20.19634	51.91821327	2.496572606	
NSUN5P2	miscRNA	5.208052	14.48780956	2.49479367	NR_033323.3
RBX1	mRNA	14.87337	38.57423766	2.493120643	NM_014248.3
TMEM150A	mRNA	2.659325	8.115983244	2.491165212	NR_033179.1
SNORD76	snoRNA	50.71995	127.8399975	2.491108136	NR_003942.1
HTRA3	mRNA	17.39688	44.82701211	2.4910203	NM_053044.4
EDC4	mRNA	7.070642	19.10226111	2.490788362	NM_014329.4
LOC1053742[illegible]	lncRNA	0	1.490362665	2.490362665	XR_942619.1

RBM22	mRNA	17.7922	45.79434693	2.490094588	NM_018047.2
TRABD	mRNA	3.110824	9.232888709	2.489254986	NM_025204.2
PHGDH	mRNA	12.7128	33.12637173	2.488651458	NM_006623.3
RARS	mRNA	50.1445	126.279334	2.488622336	NM_002887.3
FBL	mRNA	35.82875	90.61277076	2.487534118	NM_001436.3
AFG3L2	mRNA	29.9066	75.83154729	2.485927162	NM_006796.2
NOA1	mRNA	6.564649	17.80388025	2.485757033	NM_032313.2
DCTPP1	mRNA	4.395438	12.40897301	2.485242915	NM_024096.1
ETV4	mRNA	20.69374	52.91155452	2.485120212	NM_001986.2
MIR634	Precursor_m	0	1.482680383	2.482680383	NR_030364.1
MIR597	Precursor_m	0	1.482680383	2.482680383	NR_030327.1
BCKDHA	mRNA	5.83594	15.9709918	2.482612874	NM_001164783.1
CCDC142	mRNA	3.408605	9.943765651	2.482364568	NM_032779.3
ADAM15	mRNA	2.631006	8.01302578	2.48223913	NR_048577.1
GNB1L	mRNA	1.081848	4.165951202	2.481426214	NM_053004.2
RNASEK-C17	lncRNA	3.443324	10.01993694	2.480110906	NR_037717.1
DACT2	mRNA	2.48654	7.640739595	2.478313797	NR_104425.1
SEMA4B	mRNA	3.834716	10.97315658	2.476496491	NM_198925.2
ZC3H12A	mRNA	3.345343	9.759214095	2.476033197	NM_025079.2
HDHD3	mRNA	7.630071	20.36684812	2.475860117	NM_031219
RRP7A	mRNA	2.982389	8.858573613	2.475542557	NM_015703.4
UNC119	mRNA	11.54609	30.05323987	2.475133739	NM_054035.2
NHP2	mRNA	9.802298	25.73621002	2.47504827	NM_017838.3
PREB	mRNA	11.12196	29.00237512	2.475043009	NM_013388.4
GLMP	mRNA	12.87317	33.33267358	2.474753654	NM_144580.2
RNF157	mRNA	8.002642	21.26770918	2.473463692	NM_052916.2
APRT	mRNA	12.01441	31.18773173	2.473238468	NM_001030018.1
PLEKHG6	mRNA	4.774672	13.27074687	2.47126557	NM_018173.3
CYB5R3	mRNA	8.354139	22.11567371	2.471170775	NM_007326.4
SNORD26	snoRNA	21.91102	55.61039891	2.470880777	NR_002564.1
PTMA	mRNA	262.3962	649.6953765	2.470404952	NM_002823.4
MAZ	mRNA	22.73189	57.61089225	2.46970985	NR_074080.1
CMTM3	mRNA	5.85391	15.92513274	2.469412803	NR_037613.1
RPS11	mRNA	111.9294	277.8442051	2.469191424	NM_001015.4
RPS26P25	ncRNA	3.27211	9.545575035	2.468469755	NM_001093731.2
MIR6789	miRNA	0	1.467550992	2.467550992	NR_106847.1
MIR345	miRNA	0	1.467550992	2.467550992	NR_029906.1
MIR602	Precursor_m	0	1.467550992	2.467550992	NR_030333.1
MRPL38	mRNA	9.286099	24.38091381	2.467496562	NM_032478.3
STARD10	mRNA	7.852918	20.83509014	2.466428491	NM_006645.2
BCKDK	mRNA	3.477548	10.03696514	2.464957303	NM_005881.3
UBE2S	mRNA	41.11766	102.763071	2.463647742	NM_014501.2
SERINC2	mRNA	1.754441	5.783508784	2.462753251	NM_178865.4
RN7SL612P	ncRNA	0	1.462576243	2.462576243	RN7SL612P
DUS1L	mRNA	4.628054	12.8582921	2.462359447	NM_022156.4
MRPS18B	mRNA	3.213807	9.375488735	2.46226024	NM_014046.3

TUFT1	mRNA	3.626446	10.39086437	2.462119801	NM_020127.2
TEN1	mRNA	0.988963	3.894824397	2.460993451	NM_001113324.2
HIST2H2AC	mRNA	30.83587	77.34027309	2.460754526	NM_003517.2
LOC1053722!	lncRNA	0	1.460101494	2.460101494	XR_936275.1
EIF4EBP1	mRNA	18.93939	48.05161722	2.460036525	NM_004095.3
RPL24	mRNA	152.0516	375.416256	2.459408126	NM_000986.3
SNORA80B	snoRNA	2.416657	7.402499856	2.459275483	NR_028374.1
PCYT2	mRNA	5.545155	15.09520105	2.45910171	NM_002861.4
FZR1	mRNA	5.394458	14.71872527	2.458179369	NM_016263.3
APBA3	mRNA	2.787853	8.308720608	2.457518775	NM_004886.3
CDK2AP2	mRNA	17.18729	43.67003963	2.456113288	NR_073484.1
EIF5AL1	mRNA	1.069874	4.082390545	2.455410565	NM_001099692.1
FBRS	mRNA	5.994903	16.16162388	2.453446919	NM_022452.1
RHOF	mRNA	8.435968	22.1488702	2.453258747	NM_019034.2
HIST1H4J	mRNA	1.384826	4.847865074	2.452114244	NM_021968.3
MYL6	mRNA	97.09642	239.462962	2.451291832	NM_079425.1
SNORA81	snoRNA	17.54113	44.43876318	2.450701443	NR_002989.1
MRPS36	mRNA	5.811614	15.69142182	2.450435778	NM_033281.5
CNN1	mRNA	4.392539	12.21113184	2.449890652	NM_001308342.1
AP1M2	mRNA	10.56892	27.33971489	2.449642581	NM_005498.4
GSTP1	mRNA	170.1649	417.9487184	2.447631968	NM_000852.3
RPS8	mRNA	160.37	393.7199923	2.446055317	NM_001012.1
RRP12	mRNA	6.014887	16.14969241	2.444756803	NM_015179.3
DPH1	mRNA	4.716609	12.97328448	2.444330943	NM_001383.3
PGF	mRNA	5.41756	14.67550992	2.442596592	NM_002632.5
SSBP4	mRNA	3.070535	8.940301658	2.442013796	NM_032627.4
S100A6	mRNA	30.79733	76.64784623	2.441960887	NM_014624.3
TARBP2	mRNA	6.58384	17.51653812	2.441578114	NM_134324.2
LAMTOR1	mRNA	7.108537	18.78564849	2.4401009	NM_017907.2
SLC39A13	mRNA	1.552561	5.22601734	2.439125423	NM_152264.4
DENND4B	mRNA	4.920139	13.43468237	2.438233601	NM_014856.2
LOC1053719!	lncRNA	0	1.438199972	2.438199972	XR_935092.1
BCYRN1	lncRNA	0	1.438199972	2.438199972	NR_001568.1
RNU5D-1	snRNA	0	1.438199972	2.438199972	NR_002755.3
HSPBP1	mRNA	2.842231	8.366886629	2.437876778	NM_012267.4
ID1	mRNA	73.30329	180.0957629	2.437250908	NM_181353.2
SOCS1	mRNA	1.35142	4.73092096	2.437217256	NM_003745.1
TBRG4	mRNA	6.859227	18.13547416	2.434777998	NM_199122.2
CIB2	mRNA	2.878934	8.443977108	2.434683794	NR_125435.1
INO80B-WBI	lncRNA	2.489889	7.490624854	2.43292143	NR_037849.1
MVK	mRNA	6.731231	17.78632498	2.429926733	NM_001301182.1
UNC93B1	mRNA	6.652571	17.59038725	2.429299649	NM_030930.3
PEPD	mRNA	9.810904	25.25793981	2.428838388	NM_001166057.1
PET100	mRNA	3.830598	10.72783196	2.42782202	NR_033242.1
RPL39P3	ncRNA	3.423597	9.73781231	2.427393954	RPL39P3
NOL7	mRNA	17.49254	43.88593646	2.427246237	NM_016167.3

HIST1H2BG	mRNA	25.11151	62.37586395	2.427124034	NM_003518.3
CUEDC2	mRNA	2.335832	7.094799193	2.426620776	NM_024040.2
UBAC1	mRNA	9.066022	23.41793205	2.425777751	NM_016172.2
SNRPA	mRNA	26.5313	65.68574457	2.422179326	NM_004596.4
TRAPPC1	mRNA	30.29821	74.80747107	2.422102811	NR_030684.1
CCDC86	mRNA	55.62028	136.0401562	2.42033689	NM_024098.3
FDX1L	mRNA	5.244659	14.10999972	2.41966769	NM_080665.3
POR	mRNA	11.63941	29.58122391	2.419514063	NM_000941.2
SEC11A	mRNA	52.0795	127.4153831	2.419302881	NR_073518.1
LOC1005075(	mRNA	0.974029	3.775122704	2.418972612	XP_011547431.1
GAL	mRNA	2.611644	7.736038969	2.418854123	NM_015973
AKR1B10	mRNA	0.558322	2.768901418	2.418564615	NM_020299.4
RPL7AP62	ncRNA	1.310169	4.586514717	2.418227521	RPL7AP62
PTOV1	mRNA	14.08075	35.46658238	2.418088192	NR_130963.1
LOC1027233	lncRNA	1.755099	5.659021812	2.416981335	XR_433035
TUBA1B	mRNA	66.27677	161.5411149	2.416006401	NM_006082.2
IL27RA	mRNA	2.8895	8.39353519	2.415100717	NM_004843.3
SSR2	mRNA	79.66343	193.7627116	2.41451053	NM_003145.3
CDC20	mRNA	26.29322	64.82632709	2.411819398	NM_001255.2
PPT2	mRNA	3.834278	10.65918178	2.411773062	NM_138934.1
NDUFS8	mRNA	21.8518	54.10741516	2.411512996	NM_002496.3
DCPS	mRNA	2.263535	6.867437882	2.410710344	NM_014026
UBE2L6	mRNA	2.840317	8.256333172	2.410304198	NM_198183.2
RNU6-1238P	ncRNA	0	1.409999972	2.409999972	RNU6-1238P
RNU6-986P	ncRNA	0	1.409999972	2.409999972	RNU6-986P
MYDGF	mRNA	13.44682	33.79975196	2.408817426	NM_019107.3
LOC1001304	lncRNA	0.327356	2.19645414	2.408136504	NR_026874.2
TPI1	mRNA	85.25916	206.7074113	2.407946129	NM_001258026.1
NR1H2	mRNA	5.503964	14.6572533	2.407339962	NM_007121.5
HIST2H2BE	mRNA	27.49966	67.60769099	2.40731606	NM_003528.2
TMEM54	mRNA	1.168588	4.218719918	2.406506242	NM_033504.2
E2F4	mRNA	7.355842	19.10751391	2.406401758	NM_001950.3
PI4KAP2	miscRNA	11.86204	29.94139906	2.405637003	NR_003700.1
NECAP2	mRNA	6.080693	16.03239313	2.405469826	NM_018090.4
FLOT1	mRNA	6.243115	16.42182745	2.405295015	NM_005803.2
COX7C	mRNA	35.12762	85.86795901	2.404475126	NM_001867.2
PSMC4	mRNA	28.67665	70.33203624	2.40364192	NM_153001.2
DAXX	mRNA	2.167231	6.610991843	2.403042823	NM_001350.4
MIR3142HG	lncRNA	0.992518	3.784736768	2.401351646	
TALDO1	mRNA	23.26834	57.27345021	2.401212882	NM_006755.1
USP2-AS1	lncRNA	0.142156	1.741764672	2.400516234	NR_034160.1
CIDEB	mRNA	5.871998	15.48998956	2.399591769	NM_014430.2
SUV39H1	mRNA	12.06061	30.31594445	2.397740411	NM_003173.3
ABCA7	mRNA	1.056802	3.930771627	2.397300551	NM_033308.1
PDK2	mRNA	0.97563	3.735584343	2.396999814	NM_002611.4
PIN1	mRNA	14.1497	35.31130366	2.396833242	NR_038422.2

TSSC1	mRNA	6.099491	16.01438376	2.396564056	NM_003310.2
CDK5RAP3	mRNA	2.869211	8.271742392	2.396287651	NM_176096.2
BSG	mRNA	65.1664	157.5269797	2.39588356	NM_198591.2
NDUFA13	mRNA	23.34615	57.31571844	2.395274617	NM_015965.6
ISYNA1	mRNA	2.172994	6.596702351	2.394175035	NR_045573.1
HMOX1	mRNA	6.40794	16.73377326	2.39388733	NM_002133.2
PPDPF	mRNA	13.95876	34.79814604	2.393122894	NM_024299.2
WDR5	mRNA	20.47743	50.3953894	2.392995438	NM_052821.3
RPL38	mRNA	67.49377	162.8981601	2.392891606	NM_001035258.1
ECSIT	mRNA	4.161545	11.34975243	2.392646323	NM_016581.4
SLC52A2	mRNA	4.802415	12.87156139	2.390652956	NR_045600.1
RPL7AP6	ncRNA	34.32146	83.4188633	2.390016144	RPL7AP6
PLIN3	mRNA	10.04255	25.38361489	2.389268139	NM_005817.4
HIST1H2AE	mRNA	31.75932	77.26499849	2.389090751	NM_021052.2
FTL	mRNA	572.9946	1370.005186	2.388533431	NM_000146.3
SLC2A6	mRNA	4.372153	11.83106399	2.388439758	NM_017585.3
CLTB	mRNA	6.851051	17.74455177	2.387521236	NR_045724.1
LRRC45	mRNA	1.512442	4.989153885	2.383797784	NM_144999
RNU6-230P	ncRNA	0	1.382884588	2.382884588	RNU6-230P
RNU6-23P	ncRNA	0	1.382884588	2.382884588	NR_046488.1
STX18-AS1	lncRNA	1.392088	4.699241538	2.38253861	NR_037888.1
MIR548AT	miRNA	5.666643	14.87793074	2.381698029	NR_049845.1
VPS25	mRNA	7.648611	19.5894452	2.380665053	NM_032353.3
ANKZF1	mRNA	2.775348	7.987404846	2.380549775	NM_018089.2
HMGN2P4	ncRNA	0.272751	2.028995811	2.379880377	HMGN2P4
HEXDC	mRNA	1.500755	4.950600536	2.379521794	NM_173620.2
NELFE	mRNA	3.526678	9.766124089	2.378372169	NM_002904.5
ZNF511	mRNA	3.997281	10.88367546	2.378028418	NR_130127.1
POP7	mRNA	13.04787	32.40579336	2.377996816	NM_005837.2
ZNF319	mRNA	1.284761	4.429424221	2.376363876	NM_020807
ARRDC1-AS1	lncRNA	2.424761	7.137947547	2.376209057	NR_122035.1
SMA4	miscRNA	13.44771	33.30880017	2.374688541	NR_024054.2
SNAPC4	mRNA	4.35952	11.72521491	2.374319744	NM_003086.2
UBXN6	mRNA	6.934754	17.83956487	2.374309954	NM_025241.2
MMP11	mRNA	1.721512	5.461518881	2.374238764	NM_005940.3
BABAM1	mRNA	20.73356	50.59622064	2.37403462	NM_014173.3
NFKB2	mRNA	3.271259	9.136106379	2.373095791	NM_002502.5
ARL2	mRNA	3.6891	10.12610184	2.372758336	NM_001667.3
PSMA7	mRNA	50.00044	119.9893581	2.372319977	NM_152255.1
ISCU	mRNA	4.563447	12.19389636	2.371532551	NM_213595.3
MUL1	mRNA	2.145062	6.456103026	2.370733502	NM_024544.2
ARID5A	mRNA	1.368768	4.615487416	2.370636518	NM_212481.1
UNC45A	mRNA	5.494522	14.39215889	2.370021708	NM_018671.3
SNORD141B	snoRNA	0	1.369714259	2.369714259	
NUBP2	mRNA	3.81344	10.40036677	2.368444863	NR_104318.1
MRPL4	mRNA	4.374912	11.72575684	2.36762122	NM_146388.1

CD9	mRNA	9.759004	24.44449099	2.364948604	NM_001769.3
SNORA5C	snoRNA	1.199508	4.199124006	2.363766385	NR_002991.1
FAM131A	mRNA	2.888537	8.188452267	2.36295855	NM_144635.4
SHF	mRNA	2.760789	7.881335846	2.361562156	NM_138356.2
CAPZB	mRNA	32.68417	78.50865404	2.360415869	NM_004930.4
NR2F2	mRNA	31.93392	76.72276171	2.359960982	NM_021005.3
PLXNB2	mRNA	14.42658	35.39942499	2.359526925	NM_012401.3
SLC2A4RG	mRNA	8.869204	22.27663844	2.358512237	NM_020062.3
TXNDC5	mRNA	19.83588	48.1180492	2.357378012	NM_030810.3
MIR143	miRNA	0	1.356792426	2.356792426	NR_029684.1
RNU6-301P	ncRNA	0	1.356792426	2.356792426	RNU6-301P
MTND4P7	ncRNA	0	1.356792426	2.356792426	MTND4P7
ATP5E	mRNA	26.95205	64.86642242	2.356407414	NM_006886.3
GGA3	mRNA	7.754768	19.62845208	2.356253515	NR_033345.1
SF3A2	mRNA	26.39282	63.54228967	2.356175477	NM_007165.4
TMED9	mRNA	36.9221	88.32312238	2.355437105	NM_017510.4
IER5L	mRNA	3.031968	8.491217546	2.353991456	NM_203434.2
RPS12P22	ncRNA	0.940836	3.568053365	2.353652692	RPS12P22
ZBTB7A	mRNA	6.769025	17.28413727	2.353466256	NM_015898.2
C16orf58	mRNA	6.835023	17.43427623	2.352804317	NM_022744.3
AAMP	mRNA	10.38833	25.78571067	2.352031272	NM_001302545.1
RPS15	mRNA	77.17859	182.876546	2.35200634	NM_001308226.1
TFF1	mRNA	0.668019	2.923170675	2.351994134	NM_003225.2
RPS20	mRNA	69.10731	163.8383778	2.351229406	NM_001146227.1
KEAP1	mRNA	8.933077	22.3521776	2.350950961	NM_203500.1
RNF167	mRNA	15.90056	38.70990101	2.349620981	NM_015528.1
RPL17P6	ncRNA	6.521137	16.66485682	2.348695023	NM_001093734.1
SMG5	mRNA	21.52633	51.86107973	2.346634897	NM_015327.2
LOC1079839	mRNA	0	1.346629187	2.346629187	
CLTA	mRNA	31.51039	75.28441601	2.346462887	NR_132349.1
RNU4-32P	ncRNA	1.16548	4.07999992	2.34590041	RNU4-32P
HIST1H4H	mRNA	26.80292	64.21908966	2.345764126	NM_003543.3
NME3	mRNA	0.929308	3.524335464	2.345055956	NM_002513.2
RPSA	mRNA	57.08689	135.2101997	2.344938749	NM_002295
FKBP11	mRNA	32.3332	77.16103298	2.344840447	NM_016594.2
LSM2	mRNA	1.680675	5.284295351	2.344296026	NM_021177.4
RNU6-768P	ncRNA	0	1.344112123	2.344112123	RNU6-768P
RNU6-481P	ncRNA	0	1.344112123	2.344112123	RNU6-481P
SNORD90	snoRNA	0	1.344112123	2.344112123	NR_003071.1
RPLP0	mRNA	38.61297	91.82178618	2.343216897	NM_053275.3
SULT1A1	mRNA	7.144898	18.07282043	2.341689342	NM_177536.3
ARPC1B	mRNA	9.889417	24.49175368	2.340965918	NM_005720.3
HEIH	lncRNA	1.973966	5.960107992	2.3403457	NR_045680.1
ARL6IP4	mRNA	5.237694	13.58800201	2.33868509	NR_103512.1
BLOC1S5-TX	lncRNA	16.32541	39.51617465	2.338540672	NR_037616.1
LSM10	mRNA	3.545472	9.619028938	2.336177142	NM_032881

CCDC124	mRNA	15.50899	37.56594779	2.336057123	NM_138442.3
MRPL24	mRNA	12.53944	30.61228856	2.334829987	NM_145729.2
ATP6V0E2	mRNA	1.583505	5.030106968	2.334079678	NR_110612.1
EPAS1	mRNA	42.68959	100.8911479	2.332160828	NM_001430.4
CNDP2	mRNA	8.746268	21.71616752	2.330755574	NM_018235.2
LRRC41	mRNA	8.473054	21.07538629	2.330334715	NM_006369.4
RPL13AP5	miscRNA	5.244659	13.55142831	2.330219825	NR_026712.1
CHTF18	mRNA	3.574701	9.65752188	2.329665029	NM_022092.2
GPRIN1	mRNA	1.718307	5.331729934	2.329291548	NM_052899.2
C16orf91	mRNA	1.008176	3.676380296	2.328670813	NM_001272051.1
NARR	mRNA	2.742142	7.713825833	2.32856635	NM_001256281.1
GRN	mRNA	7.697723	19.2481803	2.327986212	NM_002087
LOC283278	mRNA	1.805853	5.531538354	2.327826034	XP_005276455.1
GATA6-AS1	lncRNA	2.02199	6.032718003	2.327181019	NR_102763.1
HIST1H3A	mRNA	30.13349	71.45001993	2.32707655	NM_003529.2
RNH1	mRNA	3.144737	8.644725052	2.326981374	NM_203389.2
DMAP1	mRNA	6.038226	15.36573175	2.32526366	NM_019100.4
HDGFRP2	mRNA	14.89565	35.9549993	2.3248499	NM_032631.3
LOC1001294	miscRNA	0.9499	3.53315022	2.324811986	XR_252901
RPL3	mRNA	131.7855	307.52542	2.323488135	NM_001033853.1
WRAP73	mRNA	2.701887	7.600587051	2.323297869	NM_017818.3
LOC728752	lncRNA	0	1.322248913	2.322248913	NR_036504.1
EPS15L1	mRNA	16.31802	39.21263318	2.322010381	NR_047665.1
RPLP2	mRNA	29.33321	69.43034347	2.321888791	NM_001004.3
RPL6	mRNA	97.23015	227.0211271	2.321294676	NM_001024662.1
SLC2A1	mRNA	10.38833	25.43301858	2.321061669	NM_006516.2
SIN3B	mRNA	10.65976	26.06021701	2.320821065	NM_015260.3
VAX2	mRNA	0.935387	3.489940016	2.319918976	NM_012476.2
SLC17A9	mRNA	23.46725	55.76140811	2.319893504	NM_022082.3
LRP10	mRNA	6.01681	15.27697221	2.319710962	NM_014045.3
MIR3911	miRNA	0	1.319449516	2.319449516	NR_037473.1
VTN	mRNA	211.1449	490.5136138	2.316876545	NM_000638.3
TELO2	mRNA	2.171415	6.347235605	2.316706184	NM_016111.3
FAM43B	mRNA	0.827391	3.230658341	2.315135373	NM_207334.2
ACADVL	mRNA	24.64643	58.33003688	2.313384191	NM_001270448.1
CC2D1A	mRNA	4.334048	11.33965362	2.3133751	NM_017721.4
ARPC4	mRNA	17.00855	40.64319555	2.312411911	NM_005718.4
PSMB7	mRNA	58.44738	136.4518791	2.312160575	NM_002799.3
RPS19BP1	mRNA	3.71282	9.88939077	2.310589061	NR_130151.1
POLR3H	mRNA	4.248404	11.12225073	2.30970242	NM_138338.4
TUT1	mRNA	4.30312	11.24706984	2.309408551	NM_022830.2
COPE	mRNA	19.81772	47.07655351	2.309405358	NM_199444.1
MARS	mRNA	33.54303	78.74329863	2.308520797	NM_004990.3
STRN4	mRNA	15.35321	36.74976056	2.308400293	NM_013403.2
ILK	mRNA	14.98234	35.88598778	2.307921124	NM_004517.3
COPS6	mRNA	21.89565	51.83974653	2.307850436	NM_006833.4

PLEKHO1	mRNA	3.47508	9.32667275	2.307595355	NM_016274
MIR218-1	miRNA	0	1.30745452	2.30745452	NR_029631.1
MIR204	miRNA	0	1.30745452	2.30745452	NR_029621.1
RNY1P11	ncRNA	0	1.30745452	2.30745452	RNY1P11
MIR5006	Precursor_m	0	1.30745452	2.30745452	NR_049803.1
SNORD99	snoRNA	4.108316	10.78649979	2.307315994	NR_003077.1
OSGIN1	mRNA	4.574208	11.86144307	2.307313176	NM_182981.2
PPAN	mRNA	9.034492	22.1389579	2.305942191	NM_020230.5
MAF1	mRNA	7.643379	18.92930196	2.305730408	NM_032272.4
C18orf8	mRNA	4.872397	12.53417765	2.304711092	NR_075075.1
NAA20	mRNA	39.22689	91.71130256	2.304709673	NM_181528.3
SNORD25	snoRNA	44.14907	103.0352219	2.304260564	NR_002565.1
HERPUD1	mRNA	8.892089	21.775036	2.302348406	NM_014685.3
ORAI2	mRNA	0.942431	3.47211352	2.302327821	NM_032831.3
HES4	mRNA	1.223554	4.118556621	2.301971402	NM_021170.3
ATP5B	mRNA	82.30007	190.7195188	2.301552809	NM_001686.3
TUSC2	mRNA	4.781651	12.29814703	2.300060591	NM_007275.2
LOC728715	mRNA	3.994196	10.4868748	2.300044688	XR_247802.1
LENG8	mRNA	15.52888	36.98780062	2.298267728	NM_052925.3
HEXA	mRNA	12.53586	30.10548748	2.298005975	NM_000520.4
GPR157	mRNA	1.651189	5.092191288	2.297908819	NM_024980.4
P2RX5-TAX1	lncRNA	1.2289	4.1189498	2.296626368	NR_037928.1
RPL23AP42	ncRNA	20.65383	48.72590069	2.296402065	NM_001093758.2
RNU6ATAC3	ncRNA	0	1.29567565	2.29567565	RNU6ATAC35P
FBRSL1	mRNA	6.714163	16.70366539	2.294956225	NM_001142641.1
VPS28	mRNA	8.912042	21.74435455	2.2946185	NM_183057.2
BHLHA15	mRNA	5.120847	13.03750683	2.293392694	NM_177455.3
EIF2B4	mRNA	11.05824	26.65389909	2.293361755	NM_172195.3
RPL15P3	ncRNA	6.320487	15.7751279	2.291531827	RPL15P3
FGFR1	mRNA	8.680997	21.17624644	2.290698631	NM_032191.1
CFL1	mRNA	101.3385	233.4221383	2.290655156	NM_005507.2
AMBP	mRNA	309.8713	710.7756765	2.28961506	NM_001633.3
RN7SKP203	ncRNA	126.1475	290.0270483	2.288893742	RN7SKP203
C11orf68	mRNA	1.896146	5.623730659	2.287084541	NM_031450.3
POP5	mRNA	7.050576	17.40396006	2.286042718	NM_198202.1
HLA-E	mRNA	1.790251	5.375691105	2.284988781	NM_005516.5
OAF	mRNA	7.854783	19.23264194	2.284939344	NM_178507.2
OCEL1	mRNA	3.771093	9.901111899	2.284824636	NM_024578
RPL6P19	ncRNA	2.85176	7.799349089	2.28450108	RPL6P19
KCTD17	mRNA	0.998282	3.561897127	2.282909065	NM_024681.3
HIST1H2BF	mRNA	16.43327	38.79795273	2.282874305	NM_003522.3
CXCL10	mRNA	2.70284	7.451200512	2.282356607	NM_001565.3
FKBP8	mRNA	13.7018	32.54832638	2.28191936	NM_012181
RPS27A	mRNA	18.61821	43.76544858	2.281831192	NM_002954.5
LOC643373	ncRNA	93.22718	214.0013901	2.281734401	LOC643373
SREBF1	mRNA	12.58536	29.99485244	2.281488566	NM_004176.4

DHX30	mRNA	12.56277	29.93337105	2.280756151	NR_075079.1
DBP	mRNA	1.179638	3.970734323	2.280532034	NM_001352.4
C11orf31	mRNA	37.73819	87.27281667	2.278702847	NM_170746.2
TRIM47	mRNA	1.707352	5.167558341	2.27807757	NM_033452.2
VPS72	mRNA	10.82242	25.93173862	2.278022725	NM_005997.2
NUDT2	mRNA	2.904484	7.89337659	2.277734134	NM_147173.2
PSMB4	mRNA	38.01858	87.8468091	2.277038467	NM_002796.2
CTSF	mRNA	6.119637	15.2103572	2.276851669	NM_003793.3
ARAF	mRNA	7.118637	17.48192667	2.276481547	NM_001654.4
ARFGAP2	mRNA	8.932139	21.60983452	2.276431563	NM_032389.4
MRPS18A	mRNA	6.547629	16.17974968	2.276178337	NM_018135.3
BRMS1	mRNA	39.45356	91.07598987	2.276091206	NM_015399.3
PI4KB	mRNA	6.381484	15.79640594	2.275478238	NM_002651.2
PLXNC1	mRNA	7.829625	19.08855122	2.275130656	NR_037687.1
ATP6V1F	mRNA	20.71758	48.40942378	2.275088823	NM_004231.3
PUSL1	mRNA	2.33833	6.594118448	2.274825436	NM_153339.1
BAIAP2	mRNA	1.911985	5.620650248	2.273586745	NM_017451.2
ARMC7	mRNA	2.661565	7.320777275	2.272464492	NM_024585
REEP6	mRNA	27.28903	63.26822892	2.271842441	NM_138393.1
SND1	mRNA	60.80495	139.3922436	2.271537049	NM_014390.2
TRIM11	mRNA	7.266612	17.77550527	2.271245417	NM_145214.2
DPCD	mRNA	2.056729	5.939999884	2.270400746	NM_015448.1
RPUSD3	mRNA	8.839409	21.33271352	2.269721018	NM_173659.3
BOLA3	mRNA	18.09439	42.31526993	2.268481559	NM_212552.2
PAN2	mRNA	4.282683	10.97658206	2.26714019	NM_014871.4
RPL23A	mRNA	89.11234	203.2754806	2.266897863	NM_000984.5
ILF3-AS1	lncRNA	2.533223	7.008632384	2.266664628	NR_024333.1
LAD1	mRNA	7.467035	18.18646671	2.266019583	NM_005558.3
CENPT	mRNA	6.259246	15.42473074	2.262594714	NM_025082.3
LOC10537472	lncRNA	0.422449	2.218303299	2.262508788	XR_952823.1
ZNF598	mRNA	6.87768	16.81955444	2.262030777	NM_178167.3
RPS2P46	ncRNA	3.470595	9.112143434	2.261923646	RPS2P46
PPP1R16A	mRNA	2.93649	7.903796204	2.261861546	NM_032902.5
MPG	mRNA	3.442189	9.037563827	2.259598578	NM_002434.3
CD63	mRNA	52.83069	120.5857489	2.258669691	NM_001780.5
APOE	mRNA	64.36468	146.5921637	2.25798046	NM_001302691.1
PIF1	mRNA	2.466424	6.827057458	2.257963277	NM_025049.3
C16orf95	mRNA	0.710781	2.861470532	2.257138537	NM_001256917.1
ADCY3	mRNA	0.937456	3.372917745	2.257040494	NM_004036.3
C12orf65	mRNA	6.738773	16.46660138	2.257024674	NM_152269.4
SBNO2	mRNA	5.271992	13.14091345	2.254612939	NM_014963.2
NFIX	mRNA	0.897292	3.275775287	2.253619856	NM_002501.3
RPL30	mRNA	18.68765	43.35368146	2.25286848	NM_000989.3
GAL3ST1	mRNA	1.754441	5.203451856	2.252163551	NM_004861.1
NME2	mRNA	22.81324	52.62119897	2.25173905	NM_002512.3
MIR6753	miRNA	1.002028	3.50780481	2.251618865	NR_106811.1

EPHB4	mRNA	20.84845	48.19341666	2.251574273	NM_004444.4
DPH2	mRNA	4.843611	12.15524124	2.251217636	NM_201399.1
MPST	mRNA	12.84685	30.16940658	2.251010288	NR_024038.1
AHSA1	mRNA	33.68819	77.04642707	2.24994212	NM_012111.2
AP1M1	mRNA	15.32672	35.72943469	2.249652157	NM_032493.3
FBXO2	mRNA	3.545369	9.224606234	2.249455644	NM_012168.5
MCRIP1	mRNA	2.145664	6.075345504	2.249237236	NM_207368.4
ARHGEF10L	mRNA	5.948916	14.62940884	2.249186717	NM_018125.3
ACAA1	mRNA	14.70879	34.32740674	2.248894691	NR_024024.1
LINC01237	lncRNA	0	1.248437476	2.248437476	NR_110220.1
ARAP1	mRNA	3.00706	8.007109051	2.247809673	NM_015242.4
C19orf43	mRNA	46.7716	106.3635803	2.247435285	NM_024038.2
SHISA5	mRNA	33.90007	77.42762113	2.247205352	NM_016479.4
C2orf72	mRNA	18.26428	42.2905493	2.247192434	NM_001144994.1
NDUFB10	mRNA	12.49588	29.30048723	2.245165228	NM_004548.2
ANKRD13D	mRNA	29.8667	68.28283293	2.244581607	NR_030767.1
RPUSD4	mRNA	6.869	16.66067643	2.244335519	NM_032795.2
TIMM44	mRNA	11.74978	27.61343946	2.244229266	NM_006351.3
FADS2	mRNA	138.934	313.0376294	2.244183794	NM_004265.3
ALDOC	mRNA	7.379359	17.80155406	2.243793815	NM_005165.2
TONSL	mRNA	6.005512	14.71646483	2.24344267	NM_013432.4
UQCC3	mRNA	1.516917	4.646492217	2.243416344	NM_001085372.2
C17orf49	mRNA	2.001837	5.733687596	2.243188672	NM_174893.3
EML1	mRNA	5.781083	14.21022774	2.24303798	NM_004434.2
MTA2	mRNA	26.13044	59.84299003	2.242609432	NM_004739.3
GOT2	mRNA	33.90779	77.28426332	2.242601343	NM_002080.3
BLOC1S1	mRNA	5.471242	13.50984516	2.242204203	NR_037655.1
PMM1	mRNA	1.890239	5.479841733	2.241974387	NM_002676.2
CLPTM1	mRNA	30.10579	68.71217654	2.24113174	NM_001294.3
RPS28P7	ncRNA	74.93569	169.1323167	2.240479019	RPS28P7
GTF2IP13	miscRNA	1.329956	4.21930189	2.240086329	
EMP3	mRNA	11.29911	26.54337705	2.239461239	NM_001425.2
CARS2	mRNA	5.582844	13.73820117	2.238880575	NM_024537
CDC42EP1	mRNA	25.33996	57.95041649	2.238060308	NM_152243.2
C14orf1	mRNA	12.80514	29.88467474	2.237186385	NM_007176.3
TUBB	mRNA	55.43393	125.2462939	2.237063821	NR_120608.1
MUS81	mRNA	5.957059	14.56177472	2.236832502	NM_025128.4
MRPL27	mRNA	6.311496	15.34352744	2.235319266	NM_148571.1
OIP5	mRNA	4.653433	11.6359221	2.235088466	NM_007280.1
PAM16	mRNA	2.532524	6.895479317	2.235081758	NM_016069.9
IMPDH2	mRNA	17.18563	39.64580164	2.235050345	NM_000884.2
RNU6-343P	ncRNA	3.160243	8.29730753	2.234799007	RNU6-343P
ZNF773	mRNA	2.992928	7.918932501	2.233682052	NR_130705.1
AGBL5	mRNA	1.801454	5.255298314	2.232875499	NR_104246.1
GPKOW	mRNA	11.6149	27.15982277	2.232267284	NM_015698.5
TMEM38A	mRNA	0.901826	3.244719449	2.231918442	NM_024074.1

MDK	mRNA	55.75572	125.5979057	2.230575253	NR_073039.1
FAXDC2	mRNA	15.65232	36.13833093	2.230219011	NM_032385.3
PTK2B	mRNA	3.029013	7.983228658	2.229635158	NM_173176.2
ARHGEF5	mRNA	2.904568	7.704642707	2.229348694	NM_005435.3
ERAL1	mRNA	25.52071	58.10146791	2.228502867	NM_005702.2
PMS2P2	miscRNA	0.257172	1.800563345	2.227669917	NR_003613.1
PRKAG1	mRNA	10.81249	25.30931543	2.227245958	NM_212461.1
PLA2G12B	mRNA	4.897676	12.12939735	2.226198601	NM_032562.3
HIST1H2BN	mRNA	18.29985	41.96084551	2.225967883	NM_003520.3
RPS12	mRNA	167.5997	374.275102	2.225834924	NM_001016.3
RAD9A	mRNA	16.48964	37.92851555	2.2258043	NM_004584.2
SNORD79	snoRNA	38.54716	87.00222052	2.225247269	NR_003939.1
SESN2	mRNA	4.984012	12.31112221	2.224447862	NM_031459.4
CYC1	mRNA	12.09107	28.11518742	2.224049414	NM_001916.4
SLC8B1	mRNA	2.366747	6.487532482	2.223966214	NM_024959.2
FBLIM1	mRNA	16.55892	38.01686436	2.222054362	NM_017556.2
GAA	mRNA	6.863381	16.47111079	2.221831914	NM_001079804.1
STMN3	mRNA	0.776987	2.946666609	2.220988485	NR_075070.1
SNORD110	snoRNA	50.39535	113.1383978	2.220792477	NR_003078.1
C16orf59	mRNA	1.917887	5.47717434	2.21981657	NM_025108.2
AMOTL2	mRNA	11.84586	27.51189243	2.21953872	NM_016201.3
COX4I1	mRNA	33.66743	75.93384328	2.219196665	NM_001861.3
GAK	mRNA	15.63327	35.91124068	2.219121338	NM_005255.2
INF2	mRNA	5.211467	12.78266614	2.218906654	NM_032714.2
MIR671	miRNA	0	1.218813536	2.218813536	NR_030407.1
TACC3	mRNA	32.7867	73.95601521	2.218506449	NM_006342.2
TMEM201	mRNA	4.729274	11.71019163	2.218464715	NM_001130924.2
CERCAM	mRNA	2.231354	6.164808186	2.217277483	NM_016174.4
ZNF593	mRNA	2.660061	7.113399142	2.216738848	NM_015871.4
POMGNT2	mRNA	2.22003	6.137850663	2.21670339	NM_032806.5
COX8A	mRNA	11.99272	27.79952608	2.216589783	NM_004074.2
RPL6P10	ncRNA	1.068251	3.583813581	2.216275024	RPL6P10
SPR	mRNA	6.082223	14.68758025	2.215064484	NM_003124.4
NME4	mRNA	23.35188	52.94054318	2.215046295	NM_005009.2
RN7SL381P	ncRNA	8.82323	20.75254993	2.214399028	RN7SL381P
EFHD2	mRNA	5.842335	14.15012788	2.214175175	NM_024329.5
RPP25	mRNA	1.901835	5.424843443	2.214062127	NM_017793.2
ARF1	mRNA	79.17771	176.4249293	2.212895826	NM_001658.3
CHST13	mRNA	8.01398	18.94489873	2.212662824	NM_152889.2
VAT1	mRNA	21.62899	49.06983951	2.21264146	NM_006373.3
PHB2	mRNA	35.70061	80.17772571	2.211890419	NM_001267700.1
RN7SL566P	ncRNA	1.717763	5.011149728	2.211800649	RN7SL566P
FAAP24	mRNA	1.844132	5.290137054	2.211619254	NM_152266.4
LSR	mRNA	12.84074	29.60999942	2.211586491	NM_205835.3
ICAM3	mRNA	1.235038	3.942015838	2.21115557	NM_002162.3
DGAT1	mRNA	6.712863	16.04012331	2.20931236	NM_012079.5

TFR2	mRNA	3.550079	9.050663568	2.208898616	NM_003227.3
UQCC2	mRNA	9.088652	21.28277985	2.208697486	NM_032340.3
LOC10536933	lncRNA	0	1.208571405	2.208571405	XR_950187.1
PLEKHJ1	mRNA	7.426933	17.60725366	2.208069421	NM_018049.1
ITGA5	mRNA	9.782626	22.80815955	2.208011256	NM_002205.2
RAB42	mRNA	0.162545	1.564807091	2.206200916	NM_152304.1
SNX17	mRNA	5.246004	12.7778536	2.205866979	NR_049782.1
GSG2	mRNA	11.69188	26.99517287	2.205754055	NM_031965.2
GLYCTK	mRNA	4.843489	11.88407345	2.204859847	NR_026699.1
PYM1	mRNA	16.48688	37.53866158	2.203861389	
CHDH	mRNA	11.59148	26.74654571	2.203597417	NM_018397.4
BANF1	mRNA	19.40038	43.94499914	2.203144946	NM_003860.3
AP1G2	mRNA	7.253648	17.18397227	2.203143614	NM_080545.1
NKX3-1	mRNA	1.602757	4.734093172	2.20308457	NR_046072.1
CIZ1	mRNA	13.72743	31.44436006	2.202989201	NM_012127.2
TRAF7	mRNA	7.50195	17.71526708	2.201291198	NM_206835.1
F10	mRNA	8.345017	19.56925743	2.201093544	NM_001312675.1
COMT	mRNA	6.062619	14.54429659	2.200925119	NM_007310.2
BRF1	mRNA	3.757034	9.469629445	2.20087355	NM_145696.1
STAT6	mRNA	12.39875	28.48841621	2.200833239	NR_033659.1
PARP1	mRNA	37.23774	83.1521389	2.200761471	NM_001618.3
SNRPC	mRNA	17.55402	39.82525806	2.200345485	NR_029472.1
C19orf25	mRNA	2.165624	5.964627791	2.200080501	NM_152482.2
LINC01515	lncRNA	6.292561	15.03861532	2.199311789	NR_120647.1
SRPRA	mRNA	20.98802	47.34830603	2.198847891	
RNA5SP383	ncRNA	0	1.198499977	2.198499977	RNA5SP383
RHOC	mRNA	13.41011	30.67885409	2.19837715	NM_175744.4
PNMA6A	mRNA	0.144278	1.515223851	2.198088083	NM_032882.5
NAPA	mRNA	12.19242	27.99077365	2.197532244	NR_038456.1
DNAJC11	mRNA	17.26666	39.13231627	2.197025944	NM_018198.3
SH3BP1	mRNA	3.444861	8.765426848	2.197014902	NM_018957.3
MCM2	mRNA	22.02348	49.58037542	2.196903974	NR_073375.1
ENO1	mRNA	99.17959	219.0011957	2.196068091	NM_001428.3
DDX49	mRNA	8.1249	19.03837283	2.196010187	NR_033677.1
BLOC1S4	mRNA	1.700726	4.930439749	2.195868719	NM_018366.2
TMEM258	mRNA	49.17984	109.1772241	2.195646997	NM_014206.3
UBE2M	mRNA	26.20265	58.69818783	2.194572754	NM_003969.3
SCARB1	mRNA	26.01035	58.27279974	2.194447958	NM_005505.4
LINC01194	lncRNA	11.85091	27.19673024	2.194142304	NR_033383.1
DNAJA3	mRNA	20.57875	46.32638346	2.193193535	NM_005147.5
SAMD11	mRNA	3.410192	8.671996698	2.193101032	NM_152486.2
SMPD1	mRNA	1.962571	5.496305625	2.192792889	NR_027400.1
NPM3	mRNA	8.057359	18.8589506	2.192576261	NM_006993.2
CEP131	mRNA	1.235916	3.901643759	2.192231009	NM_014984.2
UBXN1	mRNA	7.894769	18.49569809	2.191816158	NM_015853.4
ORAI1	mRNA	2.521448	6.716077254	2.19116587	NM_032790.3

MRPL55	mRNA	3.257983	8.325820615	2.190196898	NM_181465.2
CTIF	mRNA	3.726051	9.348058266	2.189578197	NM_014772.2
DRG2	mRNA	11.5122	26.39419781	2.189398375	NM_001388.4
ANXA11	mRNA	9.426347	21.82736573	2.189392473	NM_145869.1
TTR	mRNA	37.14732	82.51459473	2.189265291	NM_000371.3
MNX1	mRNA	0.755205	2.842031195	2.188935635	NM_005515.3
MRGBP	mRNA	6.181269	14.71827826	2.188788466	NM_018270.4
SFI1	mRNA	6.046167	14.41685365	2.187977292	NM_014775.3
BOP1	mRNA	7.854383	18.37008617	2.187626884	NM_015201
C20orf24	mRNA	14.7564	33.46016261	2.187057953	NR_026562.3
RRP9	mRNA	4.77832	11.63651052	2.186883209	NM_004704.4
LOC1001309[illegible]	lncRNA	2.31999	6.260399878	2.186873787	NR_024469.1
SDF4	mRNA	12.38251	28.26299831	2.18666064	NM_016547.2
STAG3L1	miscRNA	4.227454	10.42661345	2.185885208	NR_040583.2
MOGS	mRNA	8.344352	19.4243934	2.185747392	NM_006302.2
KLF16	mRNA	6.090611	14.49657748	2.185506691	NM_031918.3
SAA2-SAA4	mRNA	0.386665	2.03039996	2.185387121	NM_001199744.1
NDUFB6	mRNA	4.962426	12.02675935	2.184808508	NM_182739.2
MTX1	mRNA	1.894612	5.323434362	2.18455346	NM_198883.3
C1orf174	mRNA	5.894455	14.05655245	2.183864147	NM_207356.2
G6PD	mRNA	10.28794	23.64986598	2.183735218	NM_001042351.2
YRDC	mRNA	1.969128	5.483333226	2.183581869	NM_024640.3
POLE3	mRNA	23.26107	51.91171422	2.180930369	NR_027261.1
PFKL	mRNA	6.440648	15.22750482	2.180926323	NR_024108.1
GALE	mRNA	4.836277	11.72781093	2.18081008	NM_001127621.1
TOP3B	mRNA	8.505123	19.71212965	2.179049128	NM_003935.4
RBP1	mRNA	1.768206	5.029347582	2.17807037	NM_002899.3
MLF2	mRNA	56.05415	123.2525188	2.177800014	NR_026581.1
CSNK2B	mRNA	15.67146	35.30415825	2.17762347	NM_001320.6
HOMER3	mRNA	5.060218	12.19674888	2.177603034	NR_027297.1
GS1-124K5.4	lncRNA	1.328835	4.070377279	2.17721578	XR_252237.2
POLG	mRNA	10.12301	23.21554811	2.177068012	NM_002693.2
STXBP2	mRNA	5.284556	12.68122382	2.176959429	NR_073560.1
LINC01594	lncRNA	0	1.176923054	2.176923054	NR_131251.1
EMG1	mRNA	16.20558	36.45315332	2.176802307	NM_006331.7
SAMM50	mRNA	6.270095	14.82427078	2.17662491	NM_015380.4
SNORA48	snoRNA	9.738231	22.37199956	2.176522308	NR_002918.1
NRARP	mRNA	2.006351	5.542090697	2.176090439	NM_001004354.2
ISY1	mRNA	2.94978	7.595072693	2.17608918	NM_020701.3
C8orf82	mRNA	1.445991	4.317577555	2.173996986	NM_001001795.1
VPS51	mRNA	3.751071	9.32745634	2.173711055	NR_073519.1
MED9	mRNA	5.029082	12.10366313	2.17340934	NM_018019.2
ATP5D	mRNA	6.104727	14.43975875	2.173167082	NM_001687.4
KLHDC8B	mRNA	1.576332	4.598561061	2.173074063	NM_173546.2
LRSAM1	mRNA	3.052742	7.806875602	2.173066138	NM_138361.5
TP53I13	mRNA	7.293668	17.02198323	2.17298095	NM_138349.2

CLASRP	mRNA	9.616345	22.06860815	2.17293318	NR_103529.1
INTS1	mRNA	8.265999	19.13045093	2.172507377	NM_001080453.2
ATIC	mRNA	39.03546	85.95618153	2.171979019	NM_004044.6
HIST1H3E	mRNA	4.624079	11.20675303	2.170444841	NM_003532.2
MIR6772	miRNA	2.567698	6.741562368	2.169904267	NR_106830.1
KIFC1	mRNA	6.854364	16.03856513	2.169311993	NM_002263.3
S100A3	mRNA	0	1.16926827	2.16926827	NM_002960.1
DHX38	mRNA	9.703522	22.21557209	2.168965701	NM_014003.3
RPS5	mRNA	43.68898	95.87999813	2.167872356	NM_001009.3
SOWAHB	mRNA	0.506331	2.264881846	2.167439364	NM_001029870.1
RNASEK	mRNA	12.30896	27.84064148	2.167008902	NR_037715.1
RCN1	mRNA	9.144106	20.9785131	2.166628862	NM_002901.2
SLC27A4	mRNA	8.12408	18.76499963	2.166245835	NM_005094.3
MZT2A	mRNA	5.610944	13.31804423	2.16580931	NM_001085365.1
TCF25	mRNA	18.30155	40.75773868	2.163439876	NM_014972.2
MAN2C1	mRNA	4.033253	9.883457675	2.162310899	NM_006715.3
CDK10	mRNA	12.69664	28.61430689	2.162158854	NR_027702.1
NFKBIA	mRNA	25.27388	55.80252603	2.161939075	NM_020529.2
NDUFA2	mRNA	5.438906	12.91999975	2.161858	NR_033697.1
SMPD2	mRNA	2.126658	5.752799888	2.159750209	NM_003080.2
F8A1	mRNA	2.124159	5.746039841	2.159313992	NM_012151.3
EIF5AP4	ncRNA	1.850214	5.152200514	2.158504714	EIF5AP4
RPS2	mRNA	105.0194	227.7913331	2.158014267	NM_002952.3
UBXN11	mRNA	3.636071	9.003302577	2.157711484	NM_183008.2
TERC	telomerase_I	80.16227	174.1146751	2.157587248	NR_001566.1
RPL26L1	mRNA	6.145574	14.41427047	2.157177348	NM_016093.2
JOSD1	mRNA	10.64876	24.12340753	2.156746019	NM_014876.5
MUC13	mRNA	4.166823	10.14083342	2.156225092	NM_033049.3
ALG12	mRNA	3.874791	9.507258762	2.155427579	NM_024105.3
MMS19	mRNA	7.31797	16.9262096	2.155118397	NM_022362.4
ABHD15	mRNA	4.541734	10.94143218	2.154818544	NM_198147.2
SCAND1	mRNA	3.116447	7.867614726	2.154191376	NM_033630.2
GSTM2	mRNA	1.000672	3.308443778	2.153497852	NM_001142368.1
IVD	mRNA	5.006114	11.92668477	2.152254259	NM_002225.3
FAM85A	lncRNA	0.261053	1.713502747	2.151775918	XR_956605.1
NME1-NME2	mRNA	11.8015	26.54284118	2.151533119	NR_037149.1
ETFB	mRNA	14.77857	32.9426281	2.151185266	NM_001985.2
RNU6ATAC	snRNA	0	1.150559978	2.150559978	NR_023344.1
S100A16	mRNA	2.841159	7.260622265	2.150554498	NM_080388.1
GRWD1	mRNA	13.24832	29.63066717	2.149774516	NM_031485.3
DBNL	mRNA	5.408604	12.77391319	2.149284524	NM_014063.6
RTEL1	mRNA	1.410856	4.181303333	2.149155113	NM_032957.4
METTL17	mRNA	24.38306	53.54999895	2.149070696	NM_022734.2
COL2A1	mRNA	9.956476	22.5399501	2.148496506	NM_033150.2
STBD1	mRNA	4.956152	11.79411702	2.148050633	NM_003943.4
EPS8L3	mRNA	15.85976	35.1959321	2.146882414	NM_139053.2

RRP7BP	miscRNA	5.35561	12.64283852	2.146582239	NR_002184.2
LOC1053729	lncRNA	0.287798	1.763117304	2.145613934	XR_922599.1
WDR83	mRNA	3.619164	8.908327424	2.145047924	NR_029375.1
C1orf122	mRNA	1.246833	3.819195677	2.144883349	NM_198446.2
SNORD38B	snoRNA	2.381633	6.253043356	2.144834816	NR_001457.1
ARHGEF1	mRNA	5.136829	12.16228277	2.14480209	NM_199002.1
IPO4	mRNA	12.71081	28.40643591	2.144762424	NR_051979.1
MIEN1	mRNA	2.171294	5.800806563	2.14448966	NM_032339.3
ASF1B	mRNA	12.91117	28.83046737	2.144353136	NM_018154.2
PRMT1	mRNA	46.45691	100.7346816	2.143727492	NR_033397.4
PARK7	mRNA	19.09676	42.075779	2.143419029	NM_007262.4
MIR4292	miRNA	73.58178	158.845967	2.143230684	NR_036251.1
SV2A	mRNA	1.153603	3.61504069	2.142939092	NM_014849.4
PRPF19	mRNA	54.12237	117.0956491	2.142426833	NM_014502.4
IRF9	mRNA	3.427677	8.485208416	2.142253707	NM_006084.4
GNPTG	mRNA	3.77926	9.238239312	2.142222714	NM_032520.4
VEGFB	mRNA	3.186506	7.96786688	2.142088789	NM_003377.4
SERPINF2	mRNA	28.20894	61.56781828	2.142077928	NM_001165921.1
C1S	mRNA	3.09841	7.776819485	2.141518181	NM_201442.2
UBL7	mRNA	4.822608	11.46737519	2.14120111	NM_201265.1
MIEF2	mRNA	1.951694	5.319436611	2.140952356	NM_148886.1
SLC9A3R2	mRNA	2.999261	7.561225676	2.140701852	NM_004785.5
PPFIA3	mRNA	2.287211	6.03546593	2.140253782	NR_103842.1
CLCN7	mRNA	4.617801	11.02196913	2.139977787	NM_001287.5
PQLC2	mRNA	1.864692	5.128934897	2.139474228	NR_109848.1
SH3GLB2	mRNA	4.774626	11.35092779	2.13882731	NM_020145.3
FOXA3	mRNA	9.170902	20.75230729	2.138680247	NM_004497.2
H2BFS	ncRNA	0	1.138416864	2.138416864	NM_017445.1
F2	mRNA	27.96637	60.94187228	2.138406403	NM_001311257.1
CUTA	mRNA	6.030556	14.03414485	2.138400565	NM_015921.2
KIF12	mRNA	5.903524	13.76200496	2.138328808	NM_138424.1
LRRC20	mRNA	1.731336	4.840298413	2.138256967	NR_103467.1
PPP2R5B	mRNA	1.92309	5.249490006	2.137973595	NM_006244.3
RPS6	mRNA	263.0512	563.4829323	2.137778504	NM_001010.2
PDLIM1	mRNA	29.01823	63.17156839	2.13775297	NM_020992.3
FGFR4	mRNA	40.54496	87.7903102	2.1372104	NM_213647.2
DMKN	mRNA	6.309456	14.62044474	2.137018773	NR_033746.1
LONP1	mRNA	16.56662	36.53853861	2.136924555	NR_076392.1
KARS	mRNA	53.12898	114.6362768	2.136309995	NM_005548.2
SNRNP25	mRNA	5.296172	12.44850804	2.135981692	NM_024571.3
SNORD86	snoRNA	5.732534	13.37860439	2.135689733	NR_004399.1
SLC25A22	mRNA	1.721633	4.811821467	2.135417006	NM_024698.5
ATRAID	mRNA	6.270244	14.51918891	2.134617281	NM_080592.3
RASSF8-AS1	lncRNA	1.702929	4.769160529	2.134410669	NR_038227.1
ATOX1	mRNA	4.70482	11.1761961	2.134369809	NM_004045.3
PKM	mRNA	178.3832	381.8523788	2.134271114	NM_182471.2

LOC10798623	lncRNA	0.829963	2.905454489	2.134171427	
ECH1	mRNA	35.91117	77.77439278	2.134161191	NM_001398.2
ACD	mRNA	3.579366	8.771209897	2.133747517	NM_022914.2
ERGIC3	mRNA	37.17334	80.43812434	2.133376705	NM_198398.1
HMBS	mRNA	4.077114	9.830733985	2.133246231	NM_001258209.1
PTRF	mRNA	6.557026	15.11811748	2.132865024	NM_012232.5
POU6F2-AS1	lncRNA	0	1.132440923	2.132440923	NR_046711.1
SNORA78	snoRNA	0	1.132440923	2.132440923	NR_003020.1
AP2M1	mRNA	32.3772	70.16131873	2.132033677	NM_004068.3
RTKN	mRNA	8.304355	18.83478967	2.131774917	NM_033046.2
RNF187	mRNA	12.32626	27.40521491	2.131521882	NM_001010858.2
MCRS1	mRNA	3.437096	8.457099098	2.131371114	NM_006337.4
GPX4	mRNA	41.28715	89.11983937	2.131140039	NM_002085.4
ESPL1	mRNA	8.629841	19.52162846	2.131045412	NM_012291.4
ACTR1A	mRNA	14.01375	30.98662661	2.130489048	NM_005736.3
TRP-AGG2-6	tRNA	2.282398	5.992499883	2.130302315	TRP-AGG2-6
TIMP1	mRNA	4.400874	10.50419943	2.130062398	NM_003254.2
SRC	mRNA	9.268705	20.86812786	2.129589585	NM_198291.2
MRPS12	mRNA	3.353728	8.271651044	2.12958917	NM_033363.1
TMEM259	mRNA	17.65225	38.69754298	2.128298095	NM_033420.3
ZNF76	mRNA	3.833317	9.286551543	2.128259062	NM_003427.4
CSRP1	mRNA	21.22561	46.29523623	2.127961252	NM_004078.2
SLC4A2	mRNA	26.12109	56.69882959	2.127452846	NM_003040.3
APOC1	mRNA	45.80537	98.56629118	2.12724099	NM_001645.3
RPL4P4	ncRNA	2.05988	5.508426076	2.127020029	RPL4P4
ZNF414	mRNA	1.638645	4.609615295	2.125945711	NM_032370.2
MIR3610	Precursor_m	2.251132	5.910410844	2.125539792	NR_037404.1
TRR-CCT3-1	tRNA	2.251132	5.910410844	2.125539792	TRR-CCT3-1
SNRNP70	mRNA	49.27503	105.8243576	2.124799323	NM_003089.5
SCRIB	mRNA	6.424657	14.77338415	2.124459621	NM_182706.4
COQ9	mRNA	4.962087	11.66568361	2.124370769	NM_020312.3
ARVCF	mRNA	1.116727	3.496680693	2.124355059	NM_001670.2
RIC8A	mRNA	4.517736	10.7209197	2.124226226	NM_021932.5
SPSB2	mRNA	2.620587	6.689302195	2.123772184	NM_032641.3
LOC10272449	lncRNA	0	1.123593728	2.123593728	XR_432221
IFRD2	mRNA	5.766058	13.36591724	2.123232975	NM_006764.4
TMEM219	mRNA	3.002316	7.496259929	2.122835853	NM_194280.3
PSME1	mRNA	15.30617	33.61076988	2.122556147	NM_176783.2
PTGER4	mRNA	6.157664	14.1883311	2.121967689	NM_000958.2
LOC10537450	lncRNA	0	1.121840852	2.121840852	XR_944693.1
PUF60	mRNA	9.033994	20.28874306	2.12166196	NM_078480.2
MIR4751	Precursor_m	2.220711	5.830540427	2.120817234	NR_039906.1
SLC12A9	mRNA	3.424631	8.383346257	2.120707129	NM_020246.3
ND4L	mRNA	5630.468	11939.96522	2.12040002	YP_003024034.1
RHBDD3	mRNA	24.5697	53.21467509	2.120270312	NM_012265.1
NAT8	mRNA	1.684678	4.691239424	2.119896726	NM_003960.3

KRTAP20-4	miscRNA	16.13981	35.31294574	2.11863128	NR_023342.1
RPS3	mRNA	34.59236	74.39422241	2.118269625	NM_001260507.1
MDH2	mRNA	14.63485	32.11576366	2.118073268	NR_104165.1
ABLIM3	mRNA	2.202757	5.783399887	2.117987819	NM_014945.3
USP19	mRNA	7.762953	17.5583923	2.117823916	NM_006677.2
COG7	mRNA	3.850293	9.266631775	2.11670336	NM_153603.3
EPHX1	mRNA	9.283882	20.76396869	2.116318356	NM_001291163.1
RBM42	mRNA	15.54109	34.00297657	2.116122573	NM_024321.3
TIMM17B	mRNA	15.53691	33.99381752	2.116104393	NM_005834.4
RAB24	mRNA	5.719026	13.2167371	2.115892421	NR_109789.1
AIP	mRNA	22.69861	49.14043523	2.11575398	NM_003977
CCDC189	mRNA	0.215471	1.57145976	2.115607431	
ZNF317	mRNA	13.27587	29.20012736	2.115467161	NR_102435.1
DNPEP	mRNA	4.341628	10.29822202	2.115127063	NM_012100.2
LOC653080	ncRNA	2.184629	5.73580491	2.115098762	Z70705.1
STRIP1	mRNA	8.909018	19.95852053	2.115095584	NR_073071.1
NUP210	mRNA	15.00151	32.84235785	2.114947774	NM_024923.3
MIR877	miRNA	0	1.114883699	2.114883699	NR_030615.1
MIR1272	Precursor_m	0	1.114883699	2.114883699	NR_031674.1
HDAC11	mRNA	0.767397	2.734402882	2.112939114	NM_024827.3
SETD1A	mRNA	6.909019	15.7049489	2.112139065	NM_014712
LOC10537737	lncRNA	0	1.112010288	2.112010288	XR_949978.1
EMD	mRNA	6.136395	14.07051504	2.111782724	NM_000117.2
CTSD	mRNA	50.54603	107.848062	2.111667245	NM_001909.4
MIR3191	miRNA	2.162272	5.677105152	2.111490018	NR_036159.1
KIF20A	mRNA	16.90198	36.79405287	2.111166637	NM_005733.2
TOMM34	mRNA	13.17371	28.92211579	2.111099431	NM_006809.4
ERF	mRNA	24.22433	52.24609098	2.110901685	NM_006494.3
LOC10537285	lncRNA	0	1.110579129	2.110579129	XR_938002.1
UBALD1	mRNA	5.117223	11.90866219	2.110215932	NM_145253.2
LTBR	mRNA	20.36044	44.07182454	2.110060821	NM_002342.2
UBALD2	mRNA	3.205986	7.874348493	2.109932835	NM_182565.3
CLUH	mRNA	12.45707	27.3929742	2.109892861	NM_015229.3
NDUFB8	mRNA	17.10718	37.20264912	2.109806157	NM_005004.3
LOC10798717	lncRNA	3.883483	9.301790863	2.109517083	
RPS9	mRNA	34.50857	73.87475266	2.108638701	NM_001013.3
PHLDA2	mRNA	2.143469	5.627739021	2.108415348	NM_003311.3
UBR4	mRNA	37.38402	79.91090629	2.107932015	NM_020765.2
GNB2	mRNA	9.974518	22.12615341	2.107259179	NM_005273.3
CCAR2	mRNA	17.28951	37.54018965	2.107229521	NR_033902.1
POLR2E	mRNA	18.9791	41.09859655	2.107131539	NM_002695.3
FXR2	mRNA	13.36888	29.27437506	2.10694101	NM_004860.3
TROAP	mRNA	26.22823	56.31934212	2.105143608	NM_005480.3
PANK4	mRNA	2.928806	7.270226689	2.105022847	NM_018216
LHPP	mRNA	2.296899	5.939199376	2.104765826	NM_022126.3
SERGEF	mRNA	2.786986	6.968867217	2.104276809	NR_104040.1

RPL10P9	ncRNA	1.356253	3.956533623	2.103566006	RPL10P9
BOK	mRNA	2.816789	7.028402489	2.103444327	NM_032515.4
AP1B1	mRNA	16.08714	34.93951583	2.10330836	NM_145730.2
GALM	mRNA	6.750677	15.29137304	2.101929112	NM_138801.2
RNU6-513P	ncRNA	4.740365	11.06307671	2.101447714	RNU6-513P
HSD17B7	mRNA	10.16387	22.45811346	2.10125307	NM_016371
SERF2	mRNA	13.03543	28.4787921	2.100313093	NR_037672.1
MYO1C	mRNA	12.51079	27.35832741	2.098939971	NM_033375.4
SNORA11	snoRNA	0	1.097862574	2.097862574	NR_002953.1
MIR4517	miRNA	2.08016	5.461518881	2.097786678	NR_039742.1
RAVER1	mRNA	17.70018	38.22657871	2.097658164	NM_133452.2
RNF185	mRNA	14.29825	31.07090795	2.096377055	NR_024209.1
GUSBP9	miscRNA	13.20049	28.76399944	2.095983742	NR_033968.1
MAP7D1	mRNA	10.49519	23.08371384	2.095111477	NM_018067.4
HDAC7	mRNA	3.508383	8.443744498	2.094707777	NM_016596.3
GPX1P1	ncRNA	1.129434	3.459587561	2.094259563	GPXP1
LRRC8A	mRNA	10.67246	23.43978365	2.093798526	NM_019594.3
AK1	mRNA	2.517436	6.364799876	2.09379763	NM_000476.2
CSF3R	mRNA	1.977642	5.233564368	2.093456478	NM_172313.2
PLK3	mRNA	1.742922	4.739522635	2.09248478	NM_004073
LOC642846	lncRNA	4.978354	11.50888803	2.092363195	NR_024374.1
EXOSC4	mRNA	3.85585	9.159485855	2.092215799	NM_019037.2
TNFAIP3	mRNA	9.503408	20.97501436	2.092179491	NM_006290.3
MICALL1	mRNA	5.04514	11.6463802	2.091991177	NM_033386.3
MFI2-AS1	lncRNA	0.3456	1.814763372	2.091828204	NR_038285.1
CACTIN	mRNA	5.167979	11.9023446	2.091827099	NM_021231.1
FAH	mRNA	3.84083	9.123806166	2.091336901	NM_000137.2
LOC1004217ε	ncRNA	0.527976	2.194843331	2.090898368	LOC100421788
C2CD2L	mRNA	2.304487	5.908465221	2.090631625	NM_014807.4
MIR221	miRNA	4.4818	10.45963616	2.090487998	NR_029635.1
LENG1	mRNA	3.197825	7.774053902	2.090143077	NM_024316.2
JMJD4	mRNA	3.482966	8.3687166	2.089847976	NM_023007.2
SNORA36A	snoRNA	0	1.089545433	2.089545433	NR_002969.1
EXOC7	mRNA	8.154149	18.12788178	2.08953149	NM_015219.4
PHF19	mRNA	4.330241	10.13750968	2.089494539	NR_104601.1
ACY1	mRNA	2.350407	5.999642314	2.089191402	NM_001198898.1
ID2	mRNA	61.20307	128.952974	2.089173026	NM_002166.4
MYRF	mRNA	20.91859	44.77574925	2.08844443	NM_013279
RNASEH2C	mRNA	2.891311	7.126448694	2.088357794	NM_032193.3
STOML2	mRNA	9.876036	21.70867882	2.087955415	NM_013442.2
CLN3	mRNA	5.24009	12.02885497	2.087927503	NM_001286110.1
POLE	mRNA	13.36422	28.97959469	2.087102637	NM_006231.3
LRCH4	mRNA	6.297048	14.22200219	2.086049231	NM_002319.4
EZH1	mRNA	3.995292	9.41623782	2.085211184	NM_001991.3
TUBA1C	mRNA	38.78659	81.95953256	2.085113007	NM_032704
NEURL4	mRNA	5.385878	12.31079213	2.084410569	NM_032442.2

TLN1	mRNA	31.58445	66.91364347	2.084234958	NM_006289.3
RPL37A	mRNA	171.3755	358.1802787	2.083708675	NM_000998.4
DIABLO	mRNA	12.34831	26.81290768	2.083627576	NM_138930.3
CHPF	mRNA	3.2416	7.837733047	2.08358462	NM_024536.5
UBTF	mRNA	9.618677	21.11642816	2.08278567	NR_045058.1
C7orf43	mRNA	6.750754	15.13598562	2.081860008	NM_018275
FASTK	mRNA	10.13952	22.18466241	2.081298144	NM_033015.3
ECI1	mRNA	6.254335	14.09463341	2.080774264	NM_001919.3
CALR	mRNA	225.6457	470.5194257	2.080425483	NM_004343.3
TUBG2	mRNA	5.920456	13.39705913	2.080362864	NM_016437.2
RAB15	mRNA	7.142868	15.93460196	2.07968518	NM_198686.2
IFT22	mRNA	3.859724	9.103256192	2.078977421	NM_022777.3
DDX51	mRNA	1.637057	4.481038488	2.078468024	NM_175066.3
THOP1	mRNA	11.68176	25.35613491	2.078271595	NM_003249.3
IFITM3	mRNA	5.904167	13.34856261	2.078246728	NR_049759.1
SNORD3A	snoRNA	0.757293	2.651059856	2.077661036	NR_002906.1
PIGY	mRNA	3.514489	8.378893642	2.077509547	NM_001042616.2
WBP2	mRNA	12.76452	27.59509205	2.077449018	NM_012478.3
ATG4B	mRNA	7.565877	16.79016684	2.076864613	NM_178326.2
CCDC183	mRNA	10.05532	21.95853104	2.076695391	NM_032874.2
MIR6859-1	miRNA	9.666626	21.14999959	2.076570286	NR_106918.1
ZNF688	mRNA	0.536159	2.189755259	2.076449003	NM_145271.3
ABHD16A	mRNA	3.112909	7.539462408	2.076258417	NR_033488.1
LZTR1	mRNA	8.073817	17.83167984	2.075386702	NM_006767.3
CPQ	mRNA	7.213505	16.045761	2.075333488	NM_016134.3
PAFAH2	mRNA	3.434196	8.200262998	2.074843441	NM_000437.3
PPP2R4	mRNA	25.36201	53.69279895	2.074682771	NM_178003.2
SPAG5	mRNA	49.45515	103.6727658	2.074570439	NM_006461.3
POLR2J	mRNA	12.26184	26.51253789	2.074564724	NM_006234.4
TSFM	mRNA	7.66156	16.96829653	2.074487269	NM_005726.5
PHPT1	mRNA	15.69927	33.63532192	2.07406166	NR_109807.1
RBCK1	mRNA	11.44088	24.79529572	2.073430036	NM_031229.2
MRPL46	mRNA	8.70963	19.12805963	2.072999557	NM_022163.3
SLC2A4	mRNA	1.774313	4.749850654	2.072531709	NM_001042.2
JUNB	mRNA	8.234731	18.13589172	2.072165622	NM_002229.2
TMEM55B	mRNA	9.248158	20.23441519	2.072022662	NM_144568.3
GCDH	mRNA	4.368258	10.12286015	2.071968104	NR_102316.1
ANKRD54	mRNA	3.656113	8.644917864	2.071452783	NR_036556.1
SLC51B	mRNA	5.244659	11.93399977	2.071209921	NM_178859.3
NENF	mRNA	1.385312	3.940273896	2.071122625	NR_026598.1
ADA	mRNA	1.26572	3.692426115	2.071052938	NM_000022.2
SAMD1	mRNA	11.51214	24.91108496	2.070875398	NM_138352.1
ECI2	mRNA	10.77591	23.38057331	2.070376748	NR_028588.1
STK16	mRNA	1.844658	4.889326396	2.070311084	NR_026909.1
MAPKBP1	mRNA	1.182249	3.517899212	2.070294742	NR_049761.1
BET1L	mRNA	8.060972	17.75855852	2.070258886	NM_016526.4

LOC10798445	lncRNA	0.263776	1.615955025	2.069950967	
MICALL2	mRNA	2.477821	6.195799556	2.069053837	NM_182924.3
RN7SK	snRNA	7584.546	15693.7074	2.069028106	NR_001445.2
SNORD44	snoRNA	18.85785	40.08098282	2.068753314	NR_002750.2
CNIH2	mRNA	4.161825	9.678190839	2.068684998	NR_073078.1
YWHAH	mRNA	64.4316	134.354989	2.068648722	NM_003405.3
SNORA103	snoRNA	22.93014	48.49744091	2.068414421	
AHCY	mRNA	26.07264	54.97977879	2.067761845	NM_001161766.1
MYCN	mRNA	15.74088	33.61165175	2.067492658	NM_005378.5
INO80E	mRNA	5.951686	13.36918284	2.067007031	NR_130786.1
MAN2B2	mRNA	9.486544	20.67329766	2.066772121	NM_015274.2
RUVBL2	mRNA	13.91176	29.81549725	2.066523326	NM_006666.1
RPS14	mRNA	27.96078	58.8354534	2.066085953	NM_005617.3
CNP	mRNA	5.003618	11.40204497	2.065761962	NM_033133.4
LINC01473	lncRNA	1.90137	4.992099076	2.065265754	NR_110218.1
YKT6	mRNA	10.55508	22.86421531	2.065257839	NM_006555.3
ZDHHC18	mRNA	5.496951	12.41644449	2.065037138	NM_032283.2
RPL23	mRNA	254.0463	525.6782913	2.065029997	NM_000978.3
CAPNS1	mRNA	62.88239	130.8872041	2.064531411	NM_001749.3
KRT19	mRNA	35.86049	75.08753083	2.064202918	NM_002276.4
PKP2	mRNA	16.65909	35.44471208	2.063793536	NM_004572.3
C19orf53	mRNA	21.85082	46.14883426	2.063331826	NM_014047.2
RPL17P7	ncRNA	12.23188	26.29718395	2.062986042	RPL17P7
ATAD3B	mRNA	7.166764	15.84550849	2.062690644	NM_031921.4
GJB1	mRNA	3.722452	8.740441818	2.062581306	NM_001097642.2
MAD1L1	mRNA	4.340862	10.015595	2.062512393	NM_003550.2
RPS7P11	ncRNA	0.592831	2.282857098	2.061020827	RPS7P11
ELOVL1	mRNA	8.287378	18.13228747	2.060031003	NR_046117.1
SNORA73B	snoRNA	146.6513	303.1142979	2.059679262	NR_004406.1
TKFC	mRNA	2.378142	5.954079386	2.058551288	NM_015533.3
SNORD118	snoRNA	0	1.057499979	2.057499979	NR_033294.1
TLE2	mRNA	2.87672	6.975951724	2.057396717	NM_003260.4
UBB	mRNA	47.88589	99.57667013	2.057376105	NM_018955.3
PSMD8	mRNA	24.02524	50.46315691	2.056449983	NM_002812.4
MYL5	mRNA	0.560862	2.208839547	2.055812082	NM_002477.1
TMEM132A	mRNA	8.15722	17.81940165	2.055143624	NM_178031.2
GPC3	mRNA	62.76021	130.0282135	2.055015464	NM_004484.3
UBE2O	mRNA	12.06027	25.83657092	2.054825547	NM_022066.3
LGALS3	mRNA	5.712996	12.79380343	2.054790946	NR_003225.2
ABCB6	mRNA	8.276528	18.06089428	2.054744339	NM_005689.2
DYNC1H1	mRNA	58.20401	120.6322822	2.054460136	NM_001376.4
STAP2	mRNA	1.684383	4.51452906	2.054300132	NM_017720.2
ALOX12P2	miscRNA	16.56388	35.07171174	2.053744362	NR_002710.2
VAMP8	mRNA	9.711431	20.99805289	2.053698747	NM_003761.4
RN7SL363P	ncRNA	0	1.053626353	2.053626353	RN7SL363P
NEDD8	mRNA	57.31923	118.7377897	2.053144273	NM_006156.2

CHMP2A	mRNA	6.810182	15.02983754	2.052428197	NM_198426.2
NTMT1	mRNA	7.240711	15.91112038	2.052143453	NR_104596.1
DDX12P	miscRNA	5.296985	11.92061374	2.051873075	NR_033399.1
TMA7	mRNA	103.9945	214.3321772	2.050889521	NM_015933.4
GET4	mRNA	6.138863	13.63810318	2.050480929	NM_015949.2
STX10	mRNA	6.488367	14.35102628	2.049983062	NM_003765.2
ITIH2	mRNA	135.4237	278.6653379	2.049976081	NM_002216.2
CDC25B	mRNA	21.56421	45.25504782	2.049929487	NM_212530.1
SNORA49	snoRNA	0	1.049781001	2.049781001	NR_002979.2
FADS3	mRNA	4.644184	10.56764327	2.049480335	NM_021727.4
SLC9A3R1	mRNA	33.8284	70.34053434	2.048343853	NM_004252.4
NAA10	mRNA	3.82169	8.876189451	2.048283984	NM_003491.3
PPP1R7	mRNA	5.621277	12.56069844	2.048048815	NM_002712.2
CDAN1	mRNA	2.814153	6.810925871	2.047879425	NM_138477.2
MXD3	mRNA	2.654509	6.483245553	2.047674473	NM_031300.3
GHDC	mRNA	2.204067	5.559905217	2.047368239	NR_024573.1
SRM	mRNA	13.74419	29.18238488	2.047070375	NM_003132.2
TICAM1	mRNA	2.354509	5.864812523	2.046443349	NM_182919.3
MOCS1	mRNA	3.000068	7.18581778	2.046419862	NR_033233.1
PTMAP4	ncRNA	5.192407	11.66771967	2.045686051	V9HVW7_HUMAN
WDR46	mRNA	1.460426	4.032194166	2.045253554	NM_005452.5
RNF220	mRNA	9.951341	21.38322767	2.043880082	NM_018150.2
LOC440434	miscRNA	5.073223	11.40461237	2.042508802	NR_036750.2
KDM5C	mRNA	35.70679	73.96397116	2.042237272	NM_004187.3
BTBD2	mRNA	9.603856	20.65558401	2.042236679	NM_017797.3
MIR1302-3	miRNA	0	1.042173893	2.042173893	NR_031632.1
CCDC120	mRNA	4.831058	10.90562275	2.041760358	NM_033626.3
CASP6	mRNA	8.013814	17.4038648	2.041740077	NM_032992.2
GSK3A	mRNA	12.70806	26.98669394	2.041623339	NM_019884.2
OFCC1	miscRNA	2.626321	6.40294508	2.041447876	NM_153003.1
TMEM51	mRNA	2.925426	7.0128946	2.041280034	NM_018022.2
USP21	mRNA	3.11501	7.399636219	2.0412191	NM_012475.4
ALYREF	mRNA	20.29449	42.4424691	2.04008035	NM_005782.3
FAM73B	mRNA	2.001754	5.123442787	2.03995509	NM_032809.2
TSEN54	mRNA	4.06802	9.337216072	2.03969527	NM_207346.2
PRRC2A	mRNA	22.86141	47.63998612	2.03843707	NM_080686.2
VCP	mRNA	78.14211	160.2182348	2.03707267	NM_007126.3
TMEM63A	mRNA	11.98905	25.45601028	2.036793528	NM_014698.2
SSR4	mRNA	4.66115	10.53035599	2.036751412	NR_037927.1
RTEL1-TNFR	lncRNA	1.252709	3.588024878	2.036669978	NR_037882.1
PES1	mRNA	10.42811	22.27409147	2.036565003	NM_014303.3
URM1	mRNA	2.48248	6.08988315	2.035871922	NR_049743.1
MAU2	mRNA	16.42311	34.46582665	2.035562768	NM_015329.3
CDX2	mRNA	1.887848	4.87792113	2.035398566	NM_001265.4
FHL3	mRNA	3.371943	7.898382465	2.035338015	NM_004468.4
DDAH2	mRNA	2.090263	5.288478158	2.034933222	NM_013974

MRPL23-AS1	lncRNA	0.345237	1.737321395	2.034825155	NR_024471.1
STX4	mRNA	7.622108	16.54330209	2.034688262	NM_004604.4
RFXANK	mRNA	8.685175	18.70260047	2.034305092	NM_134440.2
MIR331	Precursor_m	1.74822	4.58999991	2.034044057	NR_029895.1
SPSB3	mRNA	1.326792	3.732352868	2.033853106	NM_080861.3
MYD88	mRNA	7.318921	15.91327735	2.033109483	NM_002468.4
SEMA3B	mRNA	1.420901	3.921928756	2.03309802	NR_110697.1
SNORA57	snoRNA	6.617422	14.47852321	2.031989682	NR_004390.1
SETDB1	mRNA	9.30812	19.94536711	2.031928832	NM_012432.3
TMEM97	mRNA	106.2588	216.915174	2.03167662	NM_014573.2
PTBP1	mRNA	77.17929	157.8093794	2.031348484	NM_175847.2
C7orf26	mRNA	2.775291	6.66646771	2.030695893	NM_024067
SNORD36A	snoRNA	31.95357	65.91749871	2.030660017	NR_002448.1
TRIM27	mRNA	7.472182	16.20336445	2.030570739	NM_030950.2
PC	mRNA	35.96579	74.05468276	2.030382433	NM_022172.2
ADHFE1	mRNA	1.474224	4.022406254	2.029891248	NM_144650.2
CSK	mRNA	9.457994	20.22792047	2.029827132	NM_004383.2
PPIB	mRNA	70.97636	145.0791217	2.029543097	NM_000942.4
CD151	mRNA	12.94292	27.2970169	2.029489992	NM_139030.3
DKK1	mRNA	42.97231	88.20428637	2.028647032	NM_012242.2
APOBEC3B	mRNA	0	1.028621566	2.028621566	NM_004900.4
ZFAND2B	mRNA	3.726364	8.587890989	2.028597691	NM_138802.2
RUVBL1	mRNA	16.0004	33.4858413	2.028530712	NM_003707.2
TAF1C	mRNA	5.067893	11.30637491	2.028113486	NM_139353.2
C1QBP	mRNA	34.61866	71.22985244	2.027865632	NM_001212.3
SNORD139	snoRNA	71.70879	146.4349062	2.02774521	
MRPL23	mRNA	1.610387	4.29217809	2.027354011	NM_021134.3
LOC10798617	lncRNA	0	1.027285694	2.027285694	
TMEM41A	mRNA	14.87256	31.17439045	2.027044323	NM_080652.3
NCAPD2	mRNA	26.36298	54.46366935	2.026960259	NM_014865.3
ABHD14A-A(	mRNA	2.09898	5.281309104	2.026895594	NR_037192.1
DNAJB1	mRNA	33.83319	69.60272591	2.0268807	NM_006145.2
ATP5G1P2	ncRNA	0.867954	2.785246425	2.026412934	ATP5GP4
RPL13P12	ncRNA	0.695343	2.434188951	2.025660664	RPL13P12
CCS	mRNA	34.18222	70.21667053	2.02422315	NM_005125.1
HGH1	mRNA	7.466715	16.13784243	2.024143149	NM_016458.3
TPRG1L	mRNA	9.67857	20.61340981	2.023998436	NM_182752.3
BCL7A	mRNA	4.734935	10.60686736	2.023888238	NM_020993
MRPL43	mRNA	6.035462	13.23866083	2.023841544	NM_176794.1
CYBA	mRNA	12.28999	25.89146389	2.023437148	NM_000101.3
C15orf57	mRNA	3.937136	8.988749825	2.023186925	NM_052849.3
PPIF	mRNA	12.09536	25.4837796	2.022378752	NM_005729.3
PCGF1	mRNA	2.138157	5.346468297	2.022355153	NM_032673.2
SKIV2L	mRNA	2.265978	5.604496293	2.022211026	NM_006929.4
PMM2	mRNA	12.66274	26.62186775	2.021693184	NM_000303.2
FDPS	mRNA	33.16609	68.07105336	2.021625647	NM_002004.3

SLC25A11	mRNA	10.60503	22.44956382	2.020637868	NM_003562.4
PGD	mRNA	49.25224	100.5372531	2.020551737	NM_002631
ATP2A1	mRNA	0.139422	1.301538436	2.019916617	NM_173201.3
LLGL1	mRNA	3.885473	8.867899688	2.019845323	NM_004140.3
PYCR2	mRNA	14.01142	29.31621625	2.019543132	NM_013328.3
EIF3FP3	ncRNA	1.305263	3.655472526	2.019497086	EIF3FP3
LINC00571	lncRNA	0.315418	1.656276359	2.01934051	NR_047500.1
RPSAP19	ncRNA	0.315418	1.656276359	2.01934051	RPSAP19
OSGEP	mRNA	18.60751	38.58801155	2.019022648	NM_017807.3
RHOT2	mRNA	9.020371	19.22997851	2.018885249	NM_138769.2
RPS4X	mRNA	127.8907	259.2069615	2.018819035	NM_001007.4
ALS2CL	mRNA	0.745949	2.524303174	2.018559758	NR_033815.1
HP	mRNA	1.021405	3.079019277	2.017913332	NM_005143.3
TECR	mRNA	38.90742	79.52663396	2.017836196	NR_038103.1
MAFF	mRNA	1.865902	4.782335673	2.017632068	NM_152878.1
FBXW5	mRNA	10.7631	22.73334753	2.017610442	NM_178226.1
NUCB1	mRNA	14.97409	31.22884778	2.017570451	NM_006184.5
C8orf58	mRNA	1.673611	4.394112426	2.017538323	NM_173686.2
ZNF503	mRNA	1.034778	3.10495946	2.017398872	NR_120651.1
PDXDC2P	miscRNA	19.36329	40.07985581	2.017348871	NR_003610.1
C1orf43	mRNA	28.48202	58.46464172	2.016979596	NM_138740.3
UCHL1	mRNA	65.79154	133.7116522	2.016896891	NM_004181.4
EME2	mRNA	0.747938	2.52479839	2.016546647	NM_001257370.1
ZNF48	mRNA	1.525243	4.090688543	2.01592014	NM_152652.2
PIAS3	mRNA	4.020544	9.118842	2.01548724	NM_006099.3
NDUFS2	mRNA	7.689743	16.51312793	2.015379337	NM_004550.4
COG4	mRNA	12.72999	26.67084455	2.015357364	NM_015386.2
FKBPL	mRNA	0.611357	2.247187456	2.015188622	NM_022110.3
TRIP10	mRNA	27.21621	55.85808172	2.015085825	NR_110231.1
NSDHL	mRNA	7.098393	15.31810621	2.014980881	NM_015922.2
ALG3	mRNA	20.56716	42.44749606	2.014520754	NR_024533.1
PSMD3	mRNA	32.53591	66.55045138	2.014272142	NM_002809.3
PDLIM7	mRNA	22.43244	46.18487358	2.01365617	NR_103804.1
CHMP1A	mRNA	10.78215	22.70257771	2.011736213	NR_046418.2
ADRM1	mRNA	21.80784	44.88218234	2.011684407	NM_175573.2
SPAG7	mRNA	7.469666	16.03810878	2.011662438	NM_004890.2
EIF5A	mRNA	102.6431	207.4879455	2.011594913	NM_001970.4
CDC45	mRNA	11.9335	25.01217342	2.011224673	NM_003504.4
TOM1	mRNA	9.582673	20.2823073	2.011052124	NR_024194.1
SLC26A1	mRNA	6.817293	14.71956849	2.010871001	NM_213613.3
HSDL2	mRNA	30.91532	63.16843362	2.010584225	NR_036651.1
TBL3	mRNA	3.167553	7.373962848	2.009323656	NM_006453.2
HSPA2	mRNA	5.932587	12.92822357	2.009094771	NM_021979.3
NTAN1	mRNA	4.313901	9.67451991	2.008791802	NM_173474.3
ZFPL1	mRNA	5.335476	11.72558419	2.008623328	NM_006782.3
FLNB	mRNA	24.73714	50.69482724	2.008568855	NM_001457.3

CRAT	mRNA	7.115665	15.2989564	2.008332729	NM_144782.1
SAT2	mRNA	6.38185	13.82347546	2.008097493	NM_133491.3
GCKR	mRNA	3.228097	7.489940466	2.007981561	NM_001486.3
CDIP1	mRNA	6.753397	14.56864427	2.007977293	NM_013399.2
PKN1	mRNA	8.963599	19.00478534	2.007787061	NM_213560.1
GTF2IP18	miscRNA	7.173251	15.40928541	2.007681671	
FAM32A	mRNA	15.57894	32.28253148	2.007519119	NM_014077
MCM7	mRNA	53.96077	109.3218455	2.007283489	NM_182776.2
TAF6L	mRNA	4.81504	10.67095442	2.007029142	NM_006473.3
NECAB3	mRNA	7.092336	15.23546807	2.006277047	NM_031232.3
RAC3	mRNA	2.934512	6.893627685	2.0062535	NM_005052.2
LOC1079857[illegible]	mRNA	0.149122	1.305081644	2.005949994	
MRPS7	mRNA	7.200441	15.44921525	2.005893932	NM_015971.3
CYSTM1	mRNA	5.959316	12.95960414	2.005887415	NM_032412.3
GPT2	mRNA	16.42501	33.95047648	2.005764933	NM_133443
RN7SL449P	ncRNA	0	1.005734246	2.005734246	RN7SL449P
MFSD14C	miscRNA	17.56227	36.22946494	2.005652814	
SHKBP1	mRNA	5.489191	12.01002064	2.004875648	NM_138392.3
XYLT2	mRNA	3.742093	8.506986782	2.00480812	NR_110010.1
POMGNT1	mRNA	4.5337	10.09091261	2.004248806	NM_017739.3
ORMDL2	mRNA	15.64486	32.3433277	2.003220377	NM_014182.4
DHCR24	mRNA	125.2624	251.8695518	2.00272999	NM_014762.3
NSUN5	mRNA	3.492153	7.993265067	2.001994489	NM_148956.3
CIC	mRNA	7.651236	16.31432548	2.001370154	NM_015125
AGPAT1	mRNA	5.660994	12.33015926	2.001226722	NM_032741.4
TNIP2	mRNA	7.076127	15.15428542	2.000251464	NM_024309.3
SCAMP2	mRNA	16.39528	33.7943753	2.00021891	NM_005697.3
ZMYM3	mRNA	6.822572	14.64528576	2.000018221	NM_201599.2

Table S4. RT-qPCR primers, sgRNA sequences and siRNA sequences

RT-qPCR primers

TRIM71-qF	AAGAAAGCCCTGGAGGAACG
TRIM71-qR	TTGTTGAGGTTTTGCCGCAG
CEBPA-qF	ACTAGGAGATTCCGGTGCCT
CEBPA-qR	GAATTCTCCCCTCCTCGCAG
ACTB-qF	TTGTTACAGGAAGTCCCTTGCC
ACTB-qR	ATGCTATCACCTCCCCTGTGTG
PSPH-qF	GAGGACGCGGTGTCAGAAAT
PSPH-qR	GGTTGCTCTGCTATGAGTCTCT
PSAT1-qF	TGCCGCACTCAGTGTTGTTAG
PSAT1-qR	GCAATTCCCGCACAAGATTCT
Psph-qF	AGGAAGCTCTTCTGTTCAGCG
Psph-qR	GAGCCTCTGGACTTGATCCC
Psat1-qF	CAGTGGAGCGCCAGAATAGAA
Psat1-qR	CCTGTGCCCCTTCAAGGAG
CDKN1A-qF	TGTCCGTCAGAACCCATGC
CDKN1A-qR	AAAGTCGAAGTTCCATCGCTC

RIP-qPCR primers

CEBPA-RIP-qPCR-F	ACTAGGAGATTCCGGTGCCT
CEBPA-RIP-qPCR-R	GAATTCTCCCCTCCTCGCAG
m6A-RIP-qF	ATTCCGGTGCCTCCTGAAAG
m6A-RIP-qR	CCCCCTCATCTTAGACGCAC

sgRNA sequences

TRIM71-g1	GCATCCGGTCTCGGGCCAGT
TRIM71-g2	CCCCGACGGAATGATCGTTG
CEBPA-g1	GTAGAAGTCGGCCGACTCCA
CEBPA-g2	GAGCCCCTGTACGAGCGCGT
sgPsph-1	CTCCAGGGATCAAGTCCAG
sgPsph-2	CTGGACATTACGCTCCTGG
sgPsph-3	TGACACGGAGAGCCATGGG

siRNA sequences

siTRIM71-1	GGGCATTGTCTGTCTTTCAAGTGCA
siTRIM71-2	CAGTGGTGGTATATCTATCTCCTTA
siMETTL3-1	CAGGAGATCCTAGAGCTATTAAATA
siMETTL3-2	CCCACCCTGGGATATTCACATGGAA
siMETTL3-3	GATGAGTCTTTAGGTGACTGCTCTT
siMETTL3-4	GCGTGAGAATTGGCTATATCCTGGA
siMETTL14-1	CATATATACCTAAGATGAGATTATA
siMETTL14-2	GGCTGCTTTGTCGGTTGAAAGTTAA
siMETTL14-3	GAGGCCAGGTCAGTGATATGCTATA
siMETTL14-4	CCTGTAATCTTAGGGTTCATGTGTG
siIGF2BP1-1	CCGGGAGCAGACCAGGCAA

siIGF2BP1-2	CCAGGCAAGCCAUCAUGAAGCUGAA
siIGF2BP1-3	GGGAAGAGCUGGAGGCCUA
siIGF2BP1-4	AAGCUGAAUGGCCACCAGUUG
siIGF2BP1-5	GUAUGGUACAGUAGAGAAC
siCEBPA-1	CGGTGGACAAGAACAGCAA
siCEBPA-2	CAGAGAGCTCCTTGGTCAA
siPSPH-1	CAGGTCTTTCTTGAGGAAATCTGTA
siPSPH-2	TATTTGCCAATAGGCTGAAATTCTA
